# Oral microbiota of periodontal health and disease and their changes after nonsurgical periodontal therapy

**DOI:** 10.1038/s41396-017-0037-1

**Published:** 2018-01-16

**Authors:** Casey Chen, Chris Hemme, Joan Beleno, Zhou Jason Shi, Daliang Ning, Yujia Qin, Qichao Tu, Michael Jorgensen, Zhili He, Liyou Wu, Jizhong Zhou

**Affiliations:** 1 Division of Periodontology, Diagnostic Sciences and Dental Hygiene, Herman Ostrow School of Dentistry of the University of Southern California, Los Angeles, CA USA; 2 Institute for Environmental Genomics, and Department of Microbiology and Plant Biology, and School of Civil Engineering and Environmental Sciences, University of Oklahoma, Norman, OK USA; 3 Earth and Environmental Sciences, Lawrence Berkeley National Laboratory, Berkeley, CA USA; 4 State Key Joint Laboratory of Environment Simulation and Pollution Control, School of Environment, Tsinghua University, Beijing, 100084 China; 5 Present Address: Department of Biomedical and Pharmaceutical Sciences, University of Rhode Island, Kingston, RI USA

**Keywords:** Microbial ecology, Infectious diseases

## Abstract

This study examined the microbial diversity and community assembly of oral microbiota in periodontal health and disease and after nonsurgical periodontal treatment. The V4 region of 16S rRNA gene from DNA of 238 saliva and subgingival samples of 21 healthy and 48 diseased subjects was amplified and sequenced. Among 1979 OTUs identified, 28 were overabundant in diseased plaque. Six of these taxa were also overabundant in diseased saliva. Twelve OTUs were overabundant in healthy plaque. There was a trend for disease-associated taxa to decrease and health-associated taxa to increase after treatment with notable variations among individual sites. Network analysis revealed modularity of the microbial communities and identified several health- and disease-specific modules. Ecological drift was a major factor that governed community turnovers in both plaque and saliva. Dispersal limitation and homogeneous selection affected the community assembly in plaque, with the additional contribution of homogenizing dispersal for plaque within individuals. Homogeneous selection and dispersal limitation played important roles, respectively, in healthy saliva and diseased pre-treatment saliva between individuals. Our results revealed distinctions in both taxa and assembly processes of oral microbiota between periodontal health and disease. Furthermore, the community assembly analysis has identified potentially effective approaches for managing periodontitis.

## Introduction

Periodontitis is one of the most common bacterial infections in humans. The disease is a consequence of destructive host immune responses to pathogenic bacterial species resulting from the dysbiosis of oral microbiota [1–3]. Therefore, there has been a continuing interest in assessing the composition and assembly of the subgingival microbiota associated with health and disease.

The assembly of oral microbiota is likely governed by similar ecological processes as observed in other ecological communities [4–10]. While a number of studies have shown that oral microbiota is individualized, specific to each oral niche and distinct in health or disease [11–15], much less is known about the ecological processes that govern the assembly of the oral microbiota, or the perturbation and the reformation of the microbiota after periodontal therapy.

Information about the composition and the assembly processes of oral microbiota could be used to develop effective strategy and monitoring protocols for periodontal therapy. Toward this goal, this study examined the subgingival and salivary microbiota in periodontally healthy subjects, and subjects diagnosed with chronic periodontitis before and after initial therapy. The microbiota of 238 samples was examined via high-throughput sequencing of 16S ribosomal RNA (rRNA) gene. Our results showed that subgingival plaque and saliva were compositionally distinct and their assembly was governed by different ecological processes. A number of taxa, which often formed distinct modules, were found to be differentially abundant in periodontal health and disease. Distinct processes were identified in the community assembly of microbiota of healthy and diseased saliva, as well as of plaque, and were affected by periodontal therapy. The results of this study provide a foundation for hypothesis testing and future studies of microbiome-based periodontal diagnosis, risk assessment, and treatment strategies.

## Materials and methods

### Subjects and microbial sampling

A total of 238 samples were obtained from 21 periodontally healthy adults and 48 adults diagnosed with chronic periodontitis recruited from the Herman Ostrow School of Dentistry of USC. Two calibrated examiners (CC and JB) were involved in the examination and microbial sampling of the study subjects. At baseline, two contralateral maxillary posterior teeth were sampled with sterile paper points as described previously [16, 17]. An unstimulated whole saliva sample was obtained from each subject. A subset (*N* = 19) of the diseased subjects were examined and sampled again at the appointment for periodontal re-evaluation at least 4 weeks after the completion of the conventional nonsurgical periodontal treatment.

### DNA extraction, amplification of 16S rDNA, and MiSeq sequencing

DNA from subgingival samples was extracted using the QIAamp DNA Mini Kit (Qiagen Inc.). DNA extraction from the saliva samples followed the protocol described previously [15, 18]. The V4 region of the 16S rDNA of sample DNA was PCR amplified with barcoded primers targeting base positions 515–806 as described previously [19] (see list of primers in Supplementary Table S1). PCR products were sequenced with the Illumina MiSeq platform at the Institute for Environmental Genomics, University of Oklahoma.

### Sequence processing and statistical analysis

A total of 10,412,986 reads (250 bp) from both ends were merged into longer reads and checked for chimeras (deposited in the NCBI Sequence Read Archive accession: SRP075100). Operational taxonomic units (OTUs) were generated at a similarity level of 97%. Representative sequences of each OTU were annotated by the Ribosomal Database Project (RDP) naive Bayesian 16S classifier [20]. Community analysis and differential abundance of OTUs were performed using STAMP 2.0.8 [21, 22], R v3.1.3 (http://www.r-project.org/), vegan (R package), and metagenomeSeq (R/Bioconductor package) [23]. A heat map of the log_2_ transformed counts of the 200 taxa with the largest overall variance was created in metagenomeSeq using the MRheatmap function.

### Network construction and analysis

Networks of subgingival and saliva bacteria were constructed and analyzed based on a random matrix theory (RMT)-based approach as described previously [24–27] and graphed using Cytoscape 3.4.0 [28].

### Mechanisms underlying community assembly

The relative roles of community assembly processes were determined as described previously by Stegen et al. [7, 8, 29].

Details of materials and methods are provided in the Supplementary Text online.

## Results

### Sample groups and clinical responses to treatment

Six different groups were identified among 238 samples based on sample sites, and health, disease, and treatment states (see study subject demographics in Supplementary Table S2 and sample site information in Table 1). The sample groups are designated as HP (subgingival plaque, periodontally healthy), D1P (subgingival plaque, periodontally diseased/pre-treatment), D2P (subgingival plaque, periodontally diseased/post-treatment), HS (saliva, periodontally healthy), D1S (saliva, periodontally diseased/pre-treatment), and D2S (saliva, periodontally diseased/post-treatment). The D2P and D2S samples were obtained at periodontal re-evaluation phase. The D2P samples were each obtained from one of the two initial sampling sites for D1P of each subject. There were statistically significant differences in periodontal probing depth (PPD), clinical attachment loss (CAL), and % bleeding on probing (BOP) of the sample sites between healthy subjects and subjects diagnosed with periodontitis, and in the PPD and % BOP of the sample sites before vs. after treatment (Table 1).

**Table 1:** Clinical characteristics of the subgingival sampling sites

Subject group	Baseline			Post-treatment		
	PPD (mm)	CAL (mm)	% BOP	PPD (mm)	CAL (mm)	% BOP
Healthy^a, b^	3.1 ± 0.42^c^	0.5 ± 1.13^c^	4.8^d^	N/A	N/A	N/A
Diseased; all sites^a, e^	5.7 ± 1.36^c^	5.4 ± 2.16^c^	86^d^	N/A	N/A	N/A
Diseased; pre- and post-treatment sites^f^	5.8 ± 1.3^g^	5.5 ± 1.6	89^h^	4.9 ± 1.41^g^	5.1 ± 1.59	21^h^

*PPD*periodontal probing depth, *CAL* clinical attachment loss, *BOP* bleeding on probing

^a^Single-site samples from two contralateral teeth of each subject

^b^From mesiopalatal site of the first molars (*N* = 42) of 21 subjects

^c^*p *<  0.0001 by Student’s *t*-test between non-diseased and diseased

^d^*p* <  0.0001 by chi-square test between non-diseased and diseased

^e^From palatal sites of maxillary molars (*N* = 90), buccal sites of maxillary molars (*N* = 4) and palatal sites of maxillary premolars (*N* = 2) of 48 subjects

^f^Among 48 subjects with periodontitis, 19 subjects were sampled again (from a site sampled before the treatment) at least 4 weeks after nonsurgical periodontal therapy

^g^*p* <  0.0005 by paired Student’s *t*-test before and after treatment

^h^*p *<  0.0001 by chi-square test before and after treatment

### Taxa abundance and diversity of the samples

A total of 1979 OTUs (>6.89 million occurrences in 238 samples) were defined with RDP annotations, including 1225 OTUs belonging to 394 genera, and 747 OTUs of unclassified genera. Most (86%) of the unclassified OTUs had fewer than 100 total occurrences each in the samples. Rarefaction curves showed that most samples leveled out between 100–300 taxa (Supplementary Figure S1).

As expected, plaque samples were more similar within each subject than between subjects (Supplementary Figure S2). The sample group HS showed less species-richness and less evenness than either D1S or D2S (Supplementary Figure S3). Welch’s *t-*test showed no significant differences among HP, D1P, and D2P, but significant differences between HS and either D1S or D2S (Supplementary Table S3). Beta diversity was greater between sample groups than within each of the sample groups (data not shown). Principal component analysis and classical multidimensional scaling analysis (Supplementary Figure S4 and Supplementary Figure S5), showed a clear separation between plaque and saliva samples without obvious separation among HS, D1S, and D2S, or between D1P and D2P. Dissimilarity analysis also showed significant differences between subgingival plaque and saliva samples and, in addition, showed differences between HP and either D1P or D2P (Supplementary Table S4).

### Differential abundance of taxa in health and disease

The distribution pattern of the top six phylotypes (comprising 98.9–99.5% of the total counts) in each sample group is shown in Supplementary Figure S6. Plaque samples showed higher abundances of *Fusobacteria* than saliva samples, whereas saliva showed higher abundances of *Firmicutes* and *Proteobacteria* than plaque. The abundance of *Bacteriodetes* and *Spirochaetes* was higher in HP than in D1P. In contrast, the abundance of *Actinobacteria* was higher in HP than in D1P.

We next identified taxa that were differentially abundant in periodontal disease and health. The taxa with a log_2_ (abundance ratio) of 2 or more between D1P and HP and between D1S and HS are shown in Table 2 (see Supplementary Table S5 for the list of taxa before filtering). Twenty-eight taxa were found to be overabundant in D1P. These included well-recognized pathogenic bacteria such as *Porphyromonas, Tannerella, Prevotella*, or *Filifactor*, and also bacteria that were not generally known to be associated with periodontitis, such as *Mycoplasma, Phocoaeicola*, *Johnsonella*, *Desulfobulbus*, and *Mogibacterium*. Twenty-four of the 28 taxa showed a decrease after treatment.

**Table 2:** Differential abundance of OTUs in the different sample groups

OTU	Taxon	Log_2_ (abundance ratio)		Adjusted *p-*value
		Pre-treatment/healthy	Post-treatment/healthy	
*Overabundant in subgingival plaque of subjects with chronic periodontitis*^a^				
OTU_13	*Filifactor*	3.51	1.96	1.66E-09
OTU_73	*Desulfobulbus*	3.45	1.93	6.29E-13
OTU_75	*Eubacterium*	3.37	1.52	5.76E-16
OTU_137	*Hallella*	3.34	1.06	1.44E-13
OTU_4	*Porphyromonas*	3.16	1.33	6.57E-06
OTU_63	*Phocaeicola*	3.10	1.22	8.52E-08
OTU_12	*Tannerella*	3.10	1.53	6.79E-09
OTU_111	Unclassified (*Bacteroidetes*)	3.07	1.26	6.69E-11
OTU_55	*Alloprevotella*	3.05	3.46	1.62E-08
OTU_8	*Porphyromonas*	3.02	2.17	9.20E-08
OTU_25	Unclassified (*Firmicutes*)	2.96	1.39	2.12E-08
OTU_58	Unclassified (*Firmicutes*)	2.93	2.20	2.11E-11
OTU_66	Unclassified (*Firmicutes*)	2.92	1.33	2.18E-10
OTU_110	*Johnsonella*	2.89	1.70	1.16E-11
OTU_109	Unclassified (*Firmicutes*)	2.73	2.25	1.75E-15
OTU_1773	*Treponema*	2.38	0.39	1.06E-06
OTU_74	*Eubacterium*	2.35	0.86	2.08E-10
OTU_129	*Treponema*	2.24	0.73	9.98E-06
OTU_147	*Treponema*	2.23	2.00	1.47E-07
OTU_606	*Treponema*	2.19	1.32	3.19E-06
OTU_131	*Prevotella*	2.19	3.19	1.32E-08
OTU_161	*Eubacterium*	2.15	0.14	1.74E-11
OTU_62	*Mycoplasma*	2.14	0.75	3.24E-09
OTU_72	*Leptotrichia*	2.14	2.19	2.67E-06
OTU_101	*Treponema*	2.12	1.18	5.40E-09
OTU_615	*Treponema*	2.11	0.86	1.18E-08
OTU_97	*Mogibacterium*	2.06	0.90	4.84E-09
OTU_33	*Treponema*	2.06	2.03	2.38E-05
*Overabundant in subgingival plaque of periodontally healthy subjects*^a^				
OTU	Taxon	Log_2_ (abundance ratio)		Adjusted *p-*value
		Healthy/pre-treatment	Healthy/post-treatment	
OTU_98	*Exiguobacterium*	2.90	2.71	2.15E-16
OTU_335	*Actinomyces*	2.73	4.33	1.12E-07
OTU_7	*Veillonella*	2.52	2.31	2.86E-08
OTU_159	*Paludibacter*	2.45	1.02	2.22E-16
OTU_550	*Capnocytophaga*	2.44	1.38	5.77E-09
OTU_15	*Actinomyces*	2.43	1.93	5.43E-09
OTU_5	*Corynebacterium*	2.38	1.65	1.79E-06
OTU_91	*Prevotella*	2.28	1.67	4.69E-07
OTU_68	*Leptotrichia*	2.17	2.05	9.26E-07
OTU_1327	*Veillonella*	2.15	2.02	1.98E-07
OTU_1274	*Leptotrichia*	2.07	2.44	2.93E-07
OTU_206	*Opitutus*	2.04	2.68	1.91E-15
*Overabundant in saliva of subjects with chronic periodontitis*^a^				
OTU	Taxon	Log_2_ (abundance ratio)		Adjusted *p-*value
		Pre-treatment/healthy	Post-treatment/healthy	
OTU_4	*Porphyromonas*	2.70	1.79	0.02179
OTU_12	*Tannerella*	2.17	1.63	0.001173
OTU_73	*Desulfobulbus*	2.13	1.20	0.000271
OTU_74	*Eubacterium*	2.09	1.86	0.013454
OTU_63	*Phocaeicola*	2.08	0.40	0.001862
OTU_97	*Mogibacterium*	2.05	2.42	0.01061
*Overabundant in Saliva of Periodontally Healthy Subjects*^a^				
OTU	Taxon	Log_2_ (abundance ratio)		Adjusted *p-*value
		Healthy/pre-treatment	Healthy/post-treatment	
OTU_57	*Prevotella*	2.33	2.35	0.00377

^a^ Analysis was conducted in metagenomeSeq after removing OTUs that contained <15 total occurrences across all samples (i.e., low-abundance taxa). The linear model used for the zero-inflated Gaussian ZIG) fit was ~Treatment + normFactor. This table shows only those OTUs with >50% effective sample size and adjusted *p*-values < 0.05. The coefficients for overabundant taxa in healthy vs. diseased samples are negative in the linear model fit

Twelve OTUs were overabundant in HP. These included initial colonizers that were compatible with periodontal health [30], such as *Actinomyces, Veillonella*, and *Capnocytophaga*, but also bacteria such as *Leptotrichia* not previously reported to be health associated. The abundances of 9 of these 12 taxa increased after treatment. Notably, some disease- and health-associated taxa belong to the same genera (e.g., *Prevotella* and *Leptotrichia*). Finally, six taxa were overabundant in D1S (Table 2). These taxa were part of the disease-associated taxa identified in D1P. Five of these taxa showed decreased abundance after treatment.

### Diversity in the composition of subgingival plaque

**Fig. 1:**
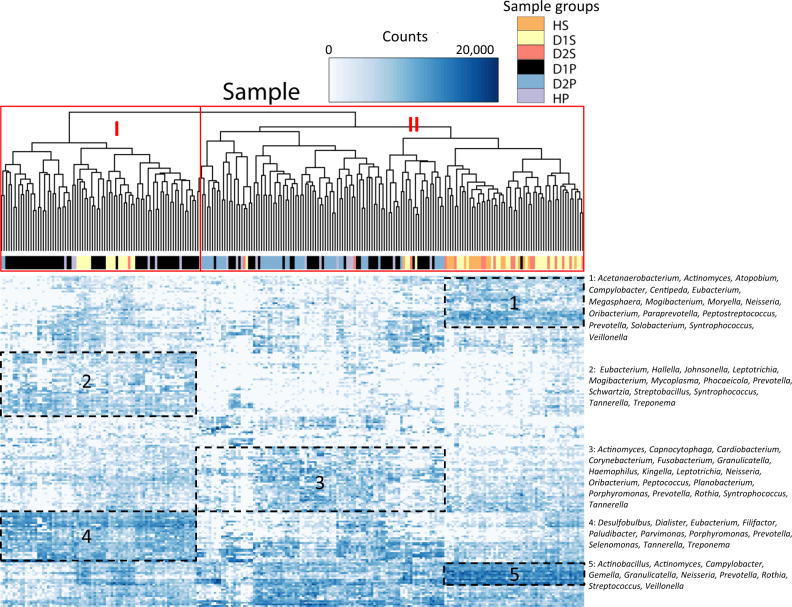
Heat map of OTU levels in the samples. A heat map of the log_2_ transformed counts of the 200 OTUs with the highest variance are displayed for the six sample groups. The genera of selected taxa are shown to the right of the map. The sample groups (identified by different colors) are: D1P (diseased/pre-treatment plaque), D2P (diseased/post-treatment plaque), HP (healthy plaque), D1S (diseased/pre-treatment saliva), D2S (diseased/post-treatment saliva), and HS (healthy saliva). Each row represents an OTU and each column represents an individual sample. The density of the color in each cell represents the count of the taxon in that sample. Two major clusters were identified. The genera of the taxa marked by boxes are provided at the right of the heat map. Cluster I was characterized by high levels of disease-associated taxa (Boxes 2 and 4; see Supplementary Table S6 for the complete list of taxa). Cluster II could be further distinguished based on sample compositions as a subgroup with predominantly subgingival plaque samples and a subgroup with predominantly saliva samples. The plaque-dominated subgroup was characterized by health-associated taxa and other bacteria considered to have low pathogenicity (Box 3; Supplementary Table S6). The saliva-dominated subgroup was characterized by high levels of *Streptococcus*, *Neisseria*, *Veillonella, Actinomyces*, *Rothia*, and *Prevotella* (Boxes 1 and 5; Supplementary Table S6)

A heat map generated by clustering analysis divided the samples into two major clusters (Cluster I and II; Fig. 1). The smaller Cluster I comprised predominantly D1P samples. This cluster was characterized by high levels of periodontal pathogens, including 27 of the 28 disease-associated taxa identified in Table 2 (Fig. 1: Box 2 (14 of the 27 taxa), Box 4 (13 of the 27 taxa); see Supplementary Table S6 for complete list of taxa), which were at lower levels in Cluster II. Cluster II could be further distinguished as two subgroups based on the sample compositions: one subgroup included predominantly subgingival plaque samples (including a majority of the D2P), and the other one predominantly saliva samples. The plaque-dominated subgroup showed higher levels of several health-associated taxa and bacteria of low pathogenicity, such as *Capnocytophaga, Neisseria, Haemophilus, Kingella*, and *Cardiobacterium* (Fig. 1: Box 3; Supplementary Table S6). The saliva-dominated subgroup was characterized by high levels of bacteria that are not generally associated with periodontitis. Presumably some of these genera, such as *Streptococcus*, *Neisseria*, *Veillonella, Actinomyces*, *Rothia*, and *Prevotella*, were saliva-enriched bacteria (Fig. 1: Boxes 1 and 5; Supplementary Table S6).

### Shift in the levels of disease- and health-associated taxa after treatment

**Fig. 2:**
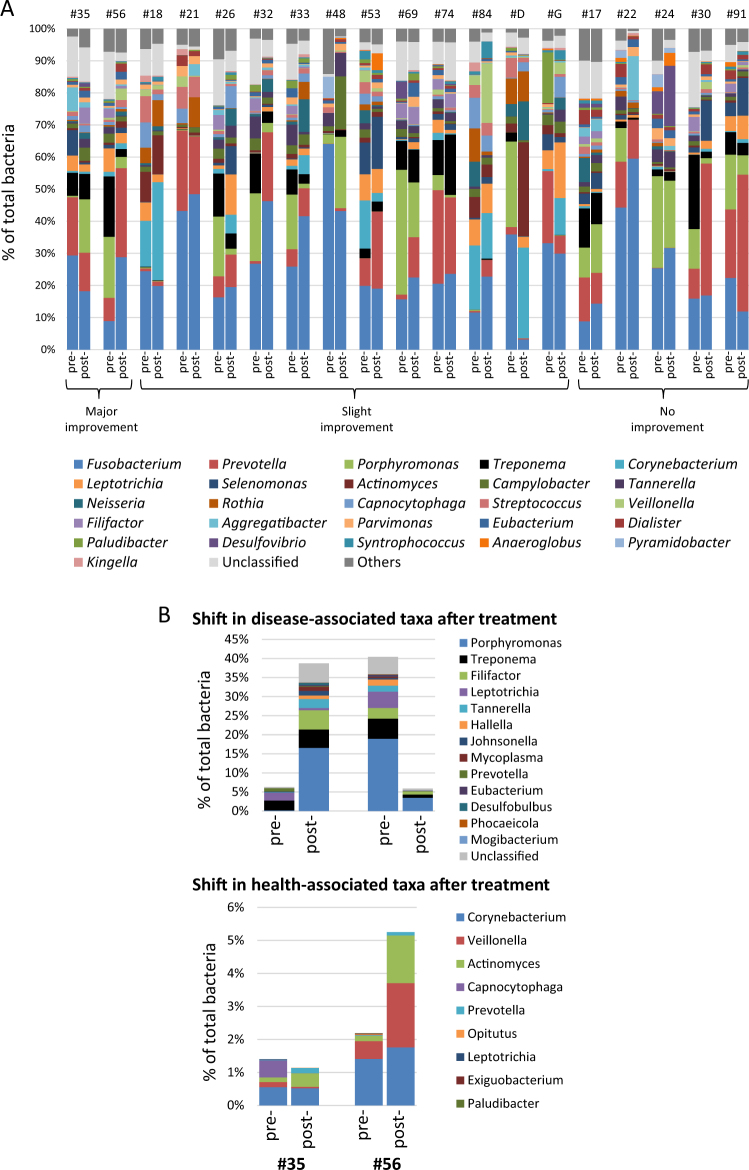
Site-specific microbiome before and after nonsurgical periodontal treatment. **a** The compositions of the pre- and post-treatment microbiome are shown side-by-side for each site (each from a different individual). The subject identification is provided at the top of the figure. The sites were defined by their treatment outcomes as effective (major improvement (MI): reduction in PPD of 2 mm or more, gain in CAL of 2 mm or more and a shift from BOP to no BOP), somewhat effective (slight improvement (SI): reduction in PPD by 1 mm and/or gain in CAL by 1 mm and a shift from BOP to no BOP), and ineffective (no improvement (NI): persistent BOP). The top 26 genera and unclassified genera that constituted approximately 95% of the microbiota are shown. Significant variations in microbiome were noted among individual sites irrespective of the treatment outcomes. **b** Examples of contrasting changes of microbiome between sites with comparable treatment outcomes. The levels of selected disease- and health-associated taxa (from Table 2) are shown

The changes in microbiome composition in individual subgingival sites after treatment are shown in Fig. 2. The treatment was considered effective in two sites (major improvement (MI)), somewhat effective in 12 sites (slight improvement (SI)), and ineffective in 5 sites (no improvement (NI); see Supplementary Text online). There were remarkable variations of microbiome compositions among these sites. For example, the levels of the pathogenic *Treponema* in pre-treatment diseased sites were as high as 23.2% in subject #30 and as low as 0.1% in subject #21 (Fig. 2a). Moreover, the shifts in the levels of disease- and health-associated genera also varied and may or may not correlate with treatment outcomes. Figure 2b shows examples of two sites with MI. In subject #56, the levels of the disease-associated taxa decreased (e.g., *Porphyromonas*, *Treponema*, and *Filifactor*) and the levels of health-associated taxa increased, as expected for a site with clinical improvement after treatment. Unexpectedly, an opposite trend occurred in a site with MI in subject #35. Here the levels of the same three pathogenic taxa increased, whereas the health-associated taxa decreased slightly. Collectively, these results revealed that while there was an overall trend for disease-associated taxa to decrease and health-associated taxa to increase after treatment, the taxa composition and the pattern of shift may differ in individual sites irrespective of the outcomes of treatment.

Similarly, the saliva microbiome exhibited significant variations before and after nonsurgical periodontal treatment among the 18 individuals examined (Supplementary Figure S7). As an example, *Neisseria* comprised up to 55.2% of the total saliva microbiota in subject D, but comprised <0.1% of the total microbiota in subject #91 before treatment. As another example, the levels of *Streptococcus* increased from 3.7 to 41.1% in subject #84, but decreased from 35 to 7.3% in subject #53 after treatment.

### Potential interactions and niche-sharing among oral taxa

Network analysis was first performed at the OTU level to provide details of the interactions among the taxa. A summary of the network parameters is provided in Supplementary Table S7. The entire set of the figures generated by the network analysis is available in Supplementary Figure S8. For each module, both the original network and the network with the OTU nodes (excluding unclassified OTUs) merged into the single genus nodes are provided.

Each network contained 136–197 nodes and 453–681 links. Among 3472 links identified, there was a predominance of positive correlations with only three that were negative (identified in HS); these are OTU_93 *Phenobacterium*/OTU_117 *Actinomyces*, OTU_29 *Eubacterium*/OTU_1329 *Veillonella*, and OTU_258 *Centipeda*/OTU_2 *Neisseria*. The topology of the post-treatment samples (D2P and D2S) was noticeably different in comparison with the corresponding pre-treatment samples. Specifically, fewer links and a lower level of centralization were found in D2P, whereas more links and a higher level of centralization were found in D2S than in other sample groups.

**Fig. 3:**
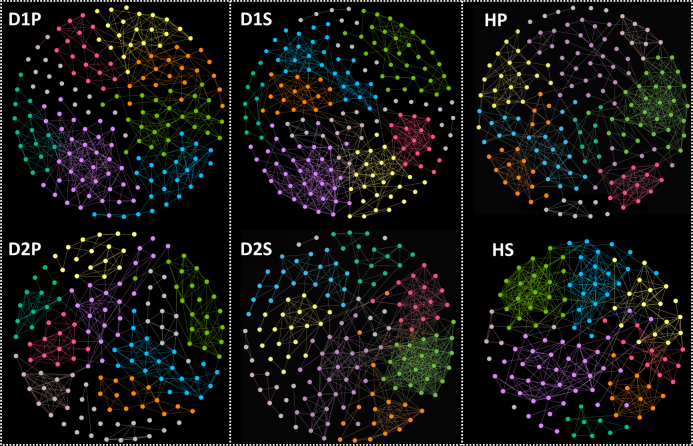
Overview of modular structures of sample groups. The network analysis was performed at the level of OTU. The nodes of the same modules are color coded in each sample group. Seven to 10 modules were identified in each of the sample groups of D1P, D2P, D1S, D2S, HP, and HS. With the exception of three interactions (not shown here; see text for details), the interactions were all positive. Within each sample group, the numbers of interactions varied among modules (See Fig. 5 for examples and Supplementary Figure S8 for all data)

Figure 3 provides an overview of the modular organization of each of the six sample groups.

**Fig. 4:**
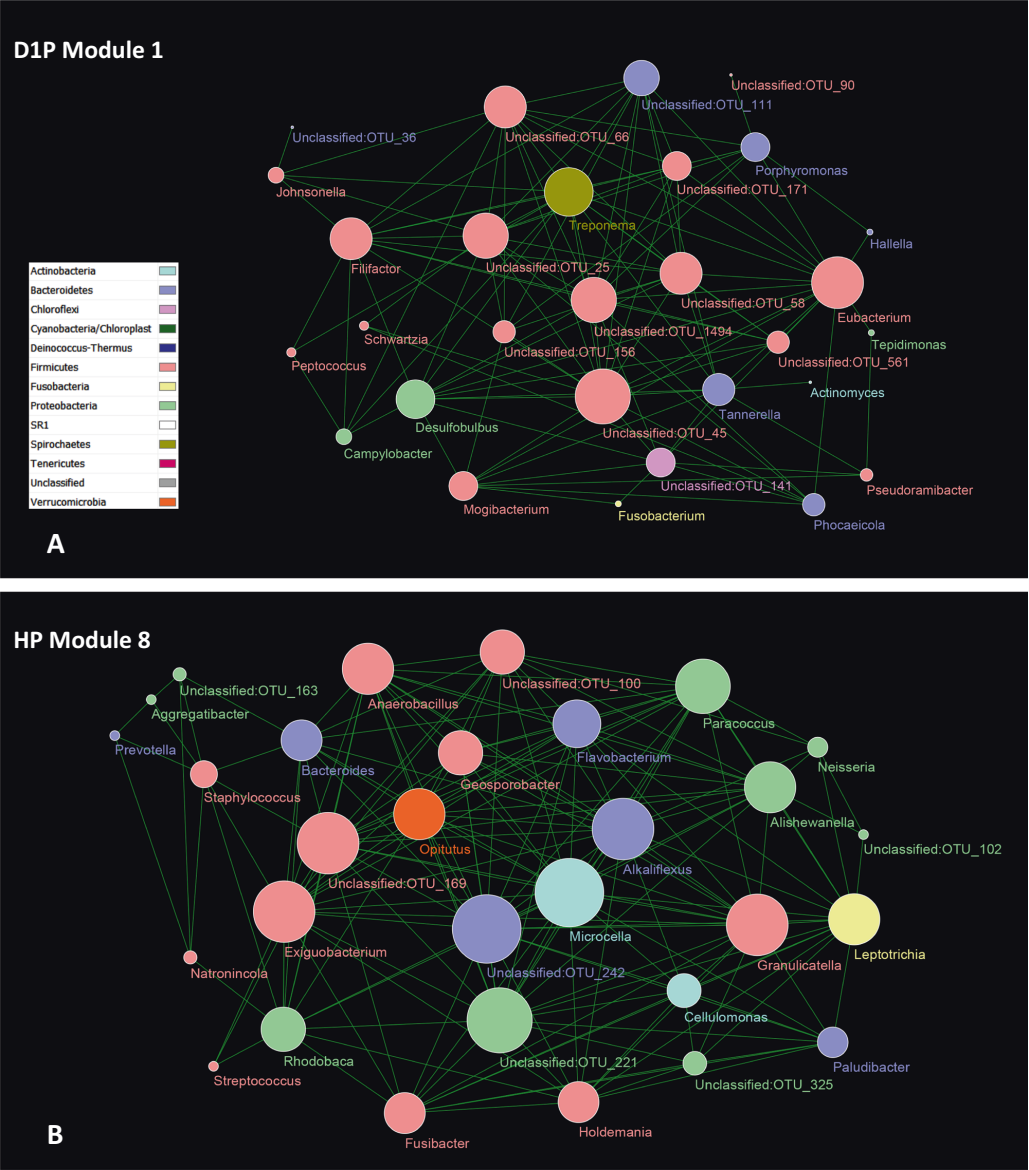
Two examples of network modules. The network analysis was performed at the level of OTU. The OTU nodes under each genus are merged into the single genus nodes, which are color coded by phyla. The size of the node correlates with the number of links of the node. **a** D1P Module 1 was one of the seven modules identified in D1P. This module included 18 of the 23 disease-associated taxa identified in Table 2, and was dominated by *Firmicutes*. **b** HP Module 8. One of the nine modules identified in HP and it contained four of the eight health-associated taxa. *Firmicutes*, *Proteobacteria*, and *Actinobacteria* were the dominant phyla and accounted for the bulk of the links in the module

Seven to 10 modules were found in each sample group. Several features of these modules were noted. The disease- or health-associated taxa were concentrated to a few modules within D1P or HP. For example, among seven modules of D1P were two modules (D1P Module 1 and D1P Module 4) that contained 23 of the 28 overabundant taxa. Included 18 of the 23 disease-associated taxa (*Porphyromonas*, *Filifactor, Treponema, Tannerella, Eubacterium, Desulfobulbus*, *Phocaeicola*, *Mogibacterium*, and unclassified genera) were found in D1P Module 1 (Fig. 4a). One of the nine modules identified in HP (Fig. 4b) contained four of the eight health-associated taxa (*Exiguobacterium, Paludibacter*, *Leptotrichia,* and *Opitutus*; Fig. 4b). Some of the modules were specific to D1P or HP, as evidenced by removing the nodes shared with HP from modules in D1P or vice versa without significantly affecting the modules (see Supplementary Figure S8; D1P-specific vs. HP and HP-specific vs. D1P). There were shared nodes and links in the modules among plaque sample groups; the highest number of shared nodes and links were found between D1P and D2P, and the lowest between D1P and HP (Supplementary Figure S8; D1P–HP intersection, D1P–D2P intersection and HP–D2P intersection).

Nine modules were identified in D1S, including three modules that collectively contained all six of the disease-associated taxa (Supplementary Figure S8; D1S Module 1, D1S Module 6 and D1S Module 7). In general, these three large modules were D1S specific (Supplementary Figure S8; D1S specific vs. HS). In contrast, few taxa and no large modules were found to be specific to either HS or D2S (data not shown).

Among 1007 nodes with connectivity to at least one other node, 989 nodes were identified as peripherals (i.e., nodes with most of their links inside their modules). For the remaining 18 nodes, three taxa (OTU_6 *Terrahaemophilus*, OTU_52 *Prevotella*, and OTU_143 *Campylobacter*) were identified as module hubs (highly connected nodes within modules), and the other 15 taxa as connectors (nodes that connect modules) (Supplementary Table S8). No taxa were identified as network hubs (i.e., highly connected nodes within entire network).

**Fig. 5:**
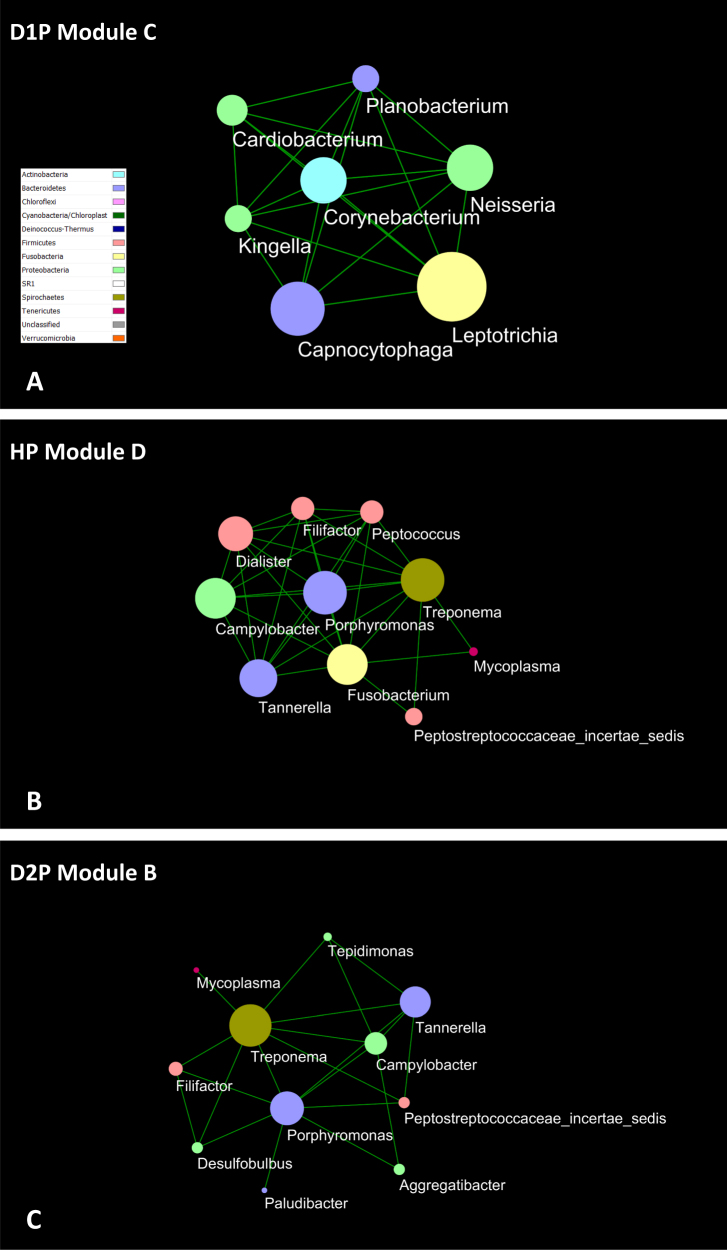
Composition and interaction among genera in network modules. The network analysis was performed at the level of genus, which led to fewer nodes, links, and modules (see Supplementary Figure S9) but accentuated the compositions of the modules and the interactions of different taxa within the modules. Modules dominated by disease- or health-associated taxa may occur in both diseased and healthy plaque, such as Module C **a** and Module D **b**. Recurrent patterns of interactions among taxa, such as *Mycoplasma, Treponema, Tannerella, Porphyromonas*, and *Filifactor*, were easily recognized **b**, **c**

Network analysis was also performed at the genus level (see Supplementary Figure S9). The results confirmed the modular organization of the microbiota, and further accentuated initial findings from the analysis at the level of OTUs. The concentration of either disease- or health-associated taxa in individual modules was evident in both D1P and HP samples. The modules dominated by disease-associated taxa may be found in non-diseased samples and vice versa. For example, Module C of D1P comprised genera that were associated with periodontal health, whereas Module D of HP comprised several well-known periodontal pathogens (Figs. 5a, b). The analysis at the genus level also readily identified the interactions among specific taxa across the sample groups, such *Mycoplasma, Treponema, Tannerella, Porphyromonas*, and *Filifactor* (Figs. 5b, c).

### Community assembly processes

**Fig. 6:**
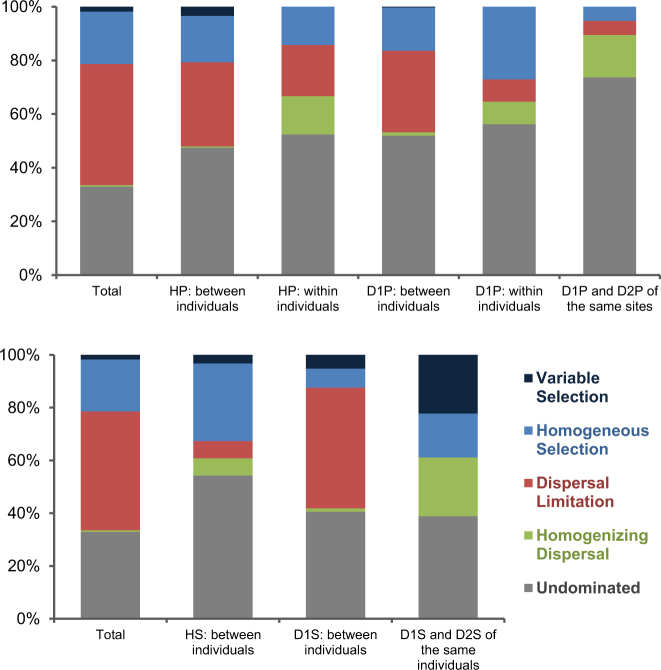
Relative roles of ecological processes in shaping microbial communities in subgingival plaque (upper) and saliva (lower). Each column displays the percentages of ecological processes for the pairwise turnovers of the indicated sample types. “Total” counts all pairwise turnovers among all samples. The permutation test was used to show whether the value of a certain group was differentiable from a random pattern. HP healthy plaque, D1P diseased/pre-treatment plaque, D2P diseased/post-treatment plaque, HS healthy saliva, D1S diseased/pre-treatment saliva, D2S diseased/post-treatment saliva. The analysis was performed for samples from different individuals, samples from the same individuals, and paired samples before and after treatment. The label “between individuals” applies to analysis of the samples (HP, HS, D1P, and D1S) from different individuals. The label “within individuals” applies to the pairs of samples from the same individuals (HP and D1P). “D1P and D2P of the same sites” is the analysis of the same diseased sites before and after treatment. “D1S and D2S of the same individuals” is the analysis of the saliva samples from the same individuals before and after treatment

Figure 6 shows a summary of the results of the analysis of community assembly processes. Five community assembly processes were evaluated. These are variable selection, homogeneous selection, dispersal limitation, homogenizing dispersal, and undominated [7, 8, 31]. “Selection” is defined as a major niche-based process, which shapes community structure due to fitness differences among different microorganisms, including effects of abiotic environmental filtering (e.g., oral temperature, oxidation-reduction status, surface texture, morphology of tooth, or dental restorations) and biotic interactions (e.g., host response and defense mechanisms, and microbial competition, commensalism, and mutualism). “Variable selection” or “homogeneous selection” are selection processes under heterogeneous or homogeneous abiotic and biotic environmental conditions, respectively, which drive communities toward more dissimilarity or similarity, respectively. “Dispersal limitation” means that the movement to and/or establishment (colonization) of taxa in a new location is restricted, which leads to communities that are more dissimilar (e.g., oral communities of non-cohabitant individuals). “Homogenizing dispersal” means a very high rate of dispersal among communities, which homogenizes the communities to become very similar (e.g., among different oral sites within individuals). “Undominated” is a turnover not differentiable from either phylogenetic or taxonomic null patterns, which mainly includes various stochastic processes, for example, drift. “Drift” means random changes of community structure due to the inherent stochastic processes of birth, death, and reproduction.

The undominated was a major factor that governed the community turnovers in plaque and saliva. In HP:between individuals (HP from different individuals), dispersal limitation and homogeneous selection were major processes of bacterial community assembly (Fig. 6; upper). HP:within individuals (HP from the same individuals) showed a greater contribution of homogenizing dispersal for community assembly, whereas dispersal limitation and homogeneous selection continued to be significant factors. These findings were similar for diseased plaque, that is, the community beta diversity was largely shaped by dispersal limitation and homogeneous selection between individuals (Fig. 6; D1P:between individuals, i.e., D1P from different individuals), and an increased contribution from homogenizing dispersal within individuals (Fig. 6; D1P:within individuals, i.e., D1P from the same individuals). In diseased sites before and after periodontal treatment (Fig. 6; D1P and D2P of the same sites), the trend for greater contribution from homogenizing dispersal was also noted. However, the more notable trends were the low contributions of dispersal limitation and homogeneous selection, and the high proportion of undominated.

The community assembly processes were different among saliva samples (Fig. 6; lower). Homogeneous selection was a major assembly process in HS:between individuals, whereas dispersal limitation was a major process for D1S:between individuals. In D1S and D2S of the same individuals, homogenizing dispersal, homogeneous selection, and variable selection were the three dominant processes.

## Discussion

The findings of different distribution patterns of phyla between plaque and saliva, or between periodontally healthy and diseased sites in this study were similar to the results in other studies [11, 12, 15, 32–36]. The subgingival microbiota from two sites of the same individual were more similar than subgingival microbiota from different individuals, similar to the results of the study by Shi et al. [37]. Our results of differential abundances of specific genera in periodontal health and disease were also in general agreement with other studies [11, 12, 33, 37]. Taken together, the following genera were found to be associated with subgingival plaque of periodontitis: *Filifactor*, *Treponema, Porphyromonas*, *Tannerella*, *Eubacterium*, *Peptostreptococcaceae*, *Desulfobulbus, Lachnospiraceae*, *Mogibacterium, Alloprevotella, Hallella*, *Phocaeicola*, *Johnsonella*, and *Mycoplasma*. The genera that were found to associated with subgingival plaque in health included *Capnocytophaga, Corynebacterium, Streptococcus, Actinomyces*, and *Veillonella*, *Exiguobacterium, Paludibacter*, and *Opitutus*. We also noted that *Leptotrichia* and *Prevotella* were associated with both disease and health, suggesting distinct pathogenic potentials of bacteria of the same genera.

Several studies have examined the changes in the subgingival microbiome in response to periodontal treatment [37–39]. The decrease in periodontal pathogenic species after treatment was observed in our previous study [38]. In a longitudinal study, Shi et al. [37] examined the subgingival microbiome at the single-site level in 12 subjects with chronic periodontitis by shot-gun sequencing and full-length sequencing of the 16S rDNA. The subjects were followed up once or twice after completion of the initial treatment. In addition to identifying several disease-associated genera, they used the microbiome profile to distinguish diseased or healthy/resolved states after treatment. In contrast to their study, this study included both periodontally healthy individuals and subjects with a wide range of periodontal disease severity, and limited the follow-up to one visit after treatment. Also, we evaluated the changes in the subgingival microbiome in sites with varying degrees of improvement, while in the study by Shi et al. [37] only clinically resolved sites were followed up. We noted that while the overall trends for disease- and health-associated taxa were to decrease and increase after treatment, respectively, the variations were significant at the level of individual sites. Moreover, the saliva microbiome in this study was likely affected by conditions other than periodontal disease status. In consideration of these factors, we are currently testing the potential use of the microbiome profile as biomarkers with a different clinical study design, in which a clinically more homogeneous group of patients will be followed up with three or more visits after initial therapy.

In this study, the findings in the diversity of the individual subgingival microbiome and the lack of concordance in their changes after periodontal treatment were similar to the results by Schwarzberg et al. [39]. In their study, the subgingival microbiota pooled from two periodontal pockets of 36 individuals (periodontally healthy, gingivitis, or periodontitis) were examined before and after initial periodontal treatment. No clear differences in microbiome composition between pre- and post-treatment subgingival plaque samples were noted. They further noted highly variable changes of specific taxa among individuals after treatment. The results in their study and this study have emphasized the importance of accounting for person-to-person variations in the microbiota when using the microbiome as biomarkers.

Correlations among oral taxa may indicate synergic or antagonistic interactions between oral bacteria, as well as their preference for ecological niches. Even though correlations are by no means the empirically validated microbial interactions, network analysis based on correlations still provides useful insights into the microbial interactomes and generates hypotheses for improving our understanding. We characterized the organization of the complex interactions by inferring the microbial ecological networks with an RMT-based approach. The RMT-based approach has been powerful in objectively selecting critical thresholds for the network inference, and its applicability has been demonstrated in characterizing interactomes in different biological systems, such as protein [40], metabolic [41], and microbial ecological networks [26]. Oral microbial communities are expected to display a range of such relationships. Our network analysis showed that each sample group comprised multiple modules with essentially all links identified to be positive correlations. Individual modules may comprise predominantly health- or disease-associated taxa, and may be found in both diseased and healthy samples. The results suggest that antagonism between oral bacteria is not a major driving force in the formation of the oral microbial community. Healthy individuals nevertheless harbor ecological niches that support disease-associated bacteria. The shift of health-compatible to disease-inducing microbiome was due to the proportional increases of pathogenic bacteria, and not due to de novo colonization of disease-associated bacteria in previously healthy individuals.

The network topology for D2P and D2S was different from that in other sample groups. This lack of stability of the microbiome in D2S is in contrast with the reported stability of the salivary microbiome in response to antibiotics [42]. Presumably standard periodontal care (plaque control, scaling and root planing, and subgingival irrigation with iodine) has a greater impact on the microbiota than a single use of antibiotics alone.

The assessment of ecological processes that governed community turnovers of oral microbiota has revealed several interesting findings. First, the between-individuals and within-individuals subgingival plaque community turnover, either in periodontal health or disease, was affected differently by two distinct processes of dispersal. In the within-individuals subgingival plaque, dispersal limitation and homogenizing dispersal contributed approximately equally to the community turnover. In contrast, in the between-individuals plaque homogenizing dispersal played essentially no role. Homogeneous selection (other than the undominated process) accounted for the other major ecological process in both groups. The findings suggested that subgingival bacteria were not freely disseminated between individuals and were selected for by local environmental factors associated with gingival crevices. This conclusion is in agreement with studies that showed limited oral transmission of oral bacteria even between long-term cohabitants such as spouses. On the contrary, bacteria can freely disseminate to subgingival and other oral sites via saliva [43–45], which explains the contribution by homogenizing dispersal for the within-individuals plaque community. Not surprisingly, homogenizing dispersal also showed relatively high contributions for both gingival and saliva communities within diseased individuals before and after treatment (Fig. 6; D1P and D2P of the same sites, and D1S and D2S of the same individuals). Conventional periodontal therapy usually treats one area of the mouth at a time. It is not uncommon for two periodontal procedures to have an interval of several weeks or longer. In this scenario, homogenizing dispersal may contribute to resistance of the pathogenic microbial community to treatment due to recolonization of bacteria from the untreated diseased sites. Our findings suggest a rationale for whole mouth treatment of periodontitis in a single visit to prevent recolonization of pathogenic bacteria in the treated sites.

Second, the assembly mechanisms of saliva for healthy and diseased individuals were noticeably different. In saliva of healthy individuals, homogeneous selection is a major process, and in a proportion greater than variable selection, dispersal limitation, and homogenizing dispersal combined. This may suggest that the oral environment in health is high. Interestingly, the influence of dispersal limitation increased and homogeneous selection decreased in saliva of individuals with periodontitis. Here the role of dispersal limitation in the assembly processes is as expected due to the limited person-to-person transmission of oral bacteria. The lesser role of homogeneous selection, however, could be due to highly individualized oral environments associated with periodontitis. It is also conceivable that diseased subjects may have greater variations in other factors such as caries experiences, oral hygiene practice, and host immune responses. These variables could explain the lower contribution of homogeneous selection (and the relatively greater contribution of dispersal limitation) in the community assembly processes of the saliva microbiome of diseased subjects.

The assembly processes of the saliva before and after treatment were also of interest (D1S and D2S of the same individuals). Here variable selection became a major factor (which had negligible contributions to community turnover in the other groups). This appears to suggest that the completion of initial periodontal treatment changed and led to highly diversified oral environments of diseased subjects. Taken together with the community assembly processes of healthy individuals, and pre- and post-treatment individuals, the findings may be interpreted as healthy oral environments are relatively homogeneous (all healthy mouths look alike), while diseased and non-diseased (post-treatment) oral environments are unique for each patient.

Third, undominated (mainly includes various stochastic processes) is the foremost process of community assembly. This finding may be the basis for the observed overall diversity of oral microbiomes among individuals. Under stochastic assembly the colonization and development of specific bacteria (e.g., pathogens) in the oral cavity is largely by chance, but with an expected probability related to the relative abundance of the bacteria in the local environment (e.g., a gingival site in the oral cavity) and meta-community (e.g., source community in the environment) [31, 46]. A major implication of this finding is that diversity of the oral microbiome is expected even between highly similar oral environments. Although it has stochasticity, we can lower the probability of infection by effectively reducing the relative abundance of pathogens in our oral and surrounding environment. A clean environment, food hygiene, and healthy habits can reduce the probability of pathogens disseminating from the environment, and daily oral hygiene and regular dental cleaning can effectively control the relative abundance of pathogens in the oral environment and gingival crevices. These practices are even more critical for patients after treatment, as the succession of gingival bacteria in a diseased individual after treatment (D1P and D2P of the same site) was shown to be mostly stochastic (>70% undominated). Therefore, the finding strongly suggests the importance of post-treatment follow-up to reduce the chance for reformation of the pathogenic microbial community.

In conclusion, microbiota were distinct in saliva and subgingival plaque and in periodontal health and disease [47–49], and exhibited changes following nonsurgical periodontal therapy. The subgingival microbiota was markedly heterogeneous. These variations were accounted for by evaluating the microbial assembly mechanisms, which were affected by the periodontal disease status, as well as by nonsurgical periodontal therapy. The results from this study can be used to design large-scale prospective studies to investigate the use of microbiome profile and community assembly processes for diagnosis and risk assessment of periodontitis.

## Electronic supplementary material

Supplementary Text online

Supplementary Table S1

Supplementary Table S2

Supplementary Table S3

Supplementary Table S4

Supplementary Table S5

Supplementary Table S6

Supplementary Table S7

Supplementary Table S8

Supplementary Figure S1

Supplementary Figure S2

Supplementary Figure S3

Supplementary Figure S4

Supplementary Figure S5

Supplementary Figure S6

Supplementary Figure S7

Supplementary Figure S8

Supplementary Figure S9
