# Supplementary material for: Oral microbiota of periodontal health and disease and their changes after nonsurgical periodontal therapy
Source: ISME J. 2018 Jan 16;12(5):1210–24. doi: 10.1038/s41396-017-0037-1 (PMC5932080; doi:10.1038/s41396-017-0037-1)
Supplement: Supplementary file 1 — Supplementary Text online [file 41396_2017_37_MOESM1_ESM.pdf]

Supplementary Text online

Materials and Methods

Calibration of examiners. Two calibrated examiners (CC & JB) were involved in the examination and sampling of the study subjects. The assessment of intra- and inter-examiner consistency was performed by a modification of a protocol described previously (Drucker et al 2012). Briefly, the examiners measured the periodontal probing depth (PPD) and clinical attachment loss (CAL) at 6 sites on each of two maxillary teeth in 9 subjects who were not involved in the main study. Each site was measured twice by the same examiner within an interval of 30 to 60 minutes. The results showed 99.1% and 98.1% intra-examiner agreement of 1 (i.e., difference of 1 mm or less) for PPD and CAL, respectively. The inter-examiner agreement of 1 was 100% for both PPD and CAL, excluding the 3 sites with an intra-examiner difference of 2 mm.

Subjects and microbial sampling. This investigation was approved by the Health Sciences Institutional Review Board of the University of Southern California (USC) (HS-11-00026). A total of 238 samples were obtained from 69 adult subjects (age 19 and over) recruited from the Herman Ostrow School of Dentistry of USC. The exclusion criteria included subjects who had periodontal treatment or antibiotic therapy during the previous six months, subjects with a medical condition that may affect their immune status, subjects who required antibiotic premedication prior to treatment, pregnant or lactating women and subjects who were taking medications (e.g., cyclosporine) that may affect the characteristics of the subgingival bacteria. Inclusion criteria for the diseased group consisted of being an adult patient with CAL and PPD >3 mm in at least 30% of the teeth. The periodontally healthy group consisted of adult patients with no CAL, no bleeding on probing (BOP), and PPDs ≤3mm in all teeth with the exception of the 3rd molars

For each study subject at baseline, two contralateral maxillary posterior teeth were sampled with sterile paper points as described previously (Ashimoto et al 1996, Chen et al 1997). After subgingival sampling, the sample sites were examined by the two designated examiners. The periodontal diagnosis, following the American Academy of Periodontology disease classification system (Armitage 2004), was verified by the examiners. The clinical measurements (i.e., PPD, CAL, BOP) were limited to the sampling sites.

An unstimulated whole saliva sample was obtained from each subject. A 50-ml sterile, disposable centrifuge tube was provided to each subject for saliva collection. During sample collection, the subjects were instructed to lower their heads and let the saliva run passively to the front bottom of their mouths and spit into the tubes provided. Each saliva donor provided 3-5 ml of whole saliva in 2 to 10 minutes. After collection, the saliva samples were immediately transferred to the laboratory and either processed immediately or kept frozen at -80°C until use.

After baseline examination and sampling, the subjects who were diagnosed with chronic periodontitis received conventional nonsurgical periodontal treatment that included oral hygiene instruction, scaling and root planing, and subgingival irrigation with 10% povidone iodine. No antibiotics were prescribed for the subjects. A subset (N=19) of the diseased subjects were resampled at the appointment for periodontal re-evaluation at least 4 weeks after the completion of the treatment for the whole mouth.

Among 19 subgingival sites, the treatment was considered effective in two sites (major improvement [MI]: reduction in PPD of 2 mm or more, gain in CAL of 2 mm or more and a shift from BOP to no BOP), and somewhat effective in 12 sites (slight improvement [SI]: reduction in PPD by 1 mm and/or gain in CAL by 1 mm and a shift from BOP to no BOP), and ineffective in 5 sites (no improvement [NI]: persistent BOP),

Extraction of DNA. Subgingival plaque samples collected by sterile paper points were immersed in PBS and vortexed to dislodge the bacteria. After removing the paper points, the samples were centrifuged at 20,000 rpm for 5 min to pellet the bacteria. The DNA was then extracted with the QIAamp DNA Mini Kit (Qiagen Inc.) following the manufacturer's protocol. DNA extraction from the saliva samples followed the protocol described previously (Quinque et al 2006, Yang et al 2012). Briefly, 500 µl of saliva was mixed with an equal volume of lysis buffer (50 mM Tris, pH 8.0, 50 mM EDTA, 50 mM sucrose, 100 mM NaCl, 1% SDS). To this mixture, 75 µl of 10% SDS and 15 µl of proteinase K (20 mg/ml) were added, and the sample was incubated at 53°C in a shaking water bath overnight. After addition of 200 µl of 5M NaCl, the mixture was incubated on ice for 10 min and then centrifuged at 13,000 rpm at room temperature. The DNA in the supernatant was precipitated with the addition of 400 µl of isopropanol and pelleted by centrifugation. The pellet was washed with 70% ethanol, dried in air, and dissolved in water until use. The amount and the quality of the DNA were determined by OD_{260} and OD_{280} measurements.

Amplification of 16S rDNA and MiSeq sequencing. Barcoded forward and reverse primers targeting 16S rDNA base positions 515-806 that included the V4 region (in reference to *Escherichia coli*) were used (Baker et al 2003). Each forward primer was comprised of an adapter, a sequencing primer, a spacer, and a 19-base primer for the 16S rDNA target. Each reverse primer also included a 12-bp barcode sequence allowing for pooling of up to 2,167 multiple samples in each lane. The list of primers is provided in Supplementary Table S1.

In a typical PCR reaction, the PCR mixture contained 2.5 µl of buffer, 1 µl each of the forward and reverse primers, 0.1 µl of *Accu*Taq, 2.5~5.0 ng of template DNA, and enough water to achieve a final volume of 25 µl. The PCR profile was as follows: 94°C/1 min; 30 cycles of 94°C/20s, 53°C/25s, 68°C/45s; and a final extension at 68°C for 10 min. After amplification, 2 µl

of the PCR product was analyzed directly by agarose gel (1%) and another 5 µl of the PCR product was analyzed by Picogreen. Based on the Picogreen result, part of the purified PCR product (about 5 µl to 10 µl containing 100 ng to 200 ng of DNA) was added to a multi-sample pool. The pooled PCR products were gel purified (1% agarose gel) and re-quantified with Picogreen, Qubit, and Bioanalyzer. Then the pooled PCR products were serially diluted to 2 nM for sequencing. PCR products were then processed for sequencing by the Illumina MiSeq platform according to the method described previously (Caporaso et al 2010, Caporaso et al 2011, Caporaso et al 2012).

Sequencing data pre-processing. After assigning each sequence to its sample according to its tag/barcode and allowing no mismatches, a total of 10,412,986 reads (250 bp) from both ends were obtained from the samples. Pair-end reads were merged into longer reads by FLASH (Magoc and Salzberg 2011) followed by the recommended UPARSE pipeline (Edgar 2013). The setting for maximum expected error was 0.5 and chimeras were checked against the Greengenes 16S core reference sequences (DeSantis et al 2006). Operational taxonomic units (OTUs) were generated at a similarity level of 97% and 10,000 sequences from each sample were resampled without replacement. Taxonomic annotations were assigned to the representative sequence of each OTU by the Ribosomal Database Project (RDP) naïve Bayesian 16S classifier (Wang et al 2007). The sequences are accessible at the NCBI Sequence Read Archive (accession: SRP075100).

16S rDNA sequence analysis. The final 16S data set was analyzed for differential abundance of OTUs or genera using a variety of tools including STAMP 2.0.8 (Parks and Beiko 2010, Parks et al 2014), R v3.1.3 (http://www.r-project.org/), vegan (R package), and metagenomeSeq (R/Bioconductor package) (Paulson et al 2013). For STAMP and R analyses, a metadata file was constructed describing the experimental metadata for each sample including subject (patient) number, treatment status (healthy, diseased pre-treatment, diseased post-treatment), subgingival

plaque vs. saliva, healthy vs. diseased, recession, PPD, CAL, gender, tobacco use, alcohol use, age, and whether the samples were paired (i.e., pre- and post-treatment samples from the same subject and site). These metadata were used to group the samples into subsets and for analysis using different factors in two-group and multi-group analyses in STAMP, and for subset-grouping and constructing linear models in metagenomeSeq.

STAMP allows for statistical analyses of two samples, two groups (multiple samples in each group), and multiple groups. The statistical parameters used for these analyses were as follows and were fully described previously (Parks and Beiko 2010, Parks et al 2014). For multiple group analysis (multiple groups of samples), the statistical test used was ANOVA with Storey's false discovery rate (FDR) approach used for multiple test correction of *p*-values. The Tukey-Kramer method was used for the post-hoc test (0.95) and the eta-squared method was used as the measure of effect size.

Abundance profiling of metagenomics data was analyzed using metagenomeSeq to test for differential abundance between metagenomic samples by fitting normalized abundance profiles to a zero-inflated Gaussian (ZIG) distribution (Paulson et al 2013). The fit was made using a user-defined linear model that included covariates of interest such as sample site (saliva, subgingival plaque) or treatment state (periodontally healthy, periodontally diseased/pre-treatment, periodontally diseased/post-treatment). Rare features (OTUs consisting of cumulatively <15 occurrences across all samples) were removed and normalization factors were determined for each trimmed data set using the *cumNorm* function of metagenomeSeq (0.75). The *fitZig* output was examined both with and without the effective sample size cutoff (eff = 0.5) to identify those samples most likely to be differentially abundant, with *p*-values adjusted to account for multiple corrections using the FDR approach.

Pearson correlation coefficients and Bray-Curtis dissimilarity values were calculated in R for all pairs of samples. A heat map of the $\log_2$ transformed counts of the 200 OTUs with the largest overall variance was created in metagenomeSeq using the MRheatmap function. The heatmap was clustered both by OTU (column) and sample (row). Figures were reformatted in Adobe Illustrator without modification of the underlying data.

Principal component analysis (PCA) plots were constructed in STAMP using different factors defined in the metadata file. Alpha and beta diversity, hierarchical clustering and detrended correspondence analysis (DCA) were conducted using the Institute for Environmental Genomics (IEG) pipeline (http://ieg.ou.edu) or R. Classical multidimensional scaling (CMDS) plots were constructed in metagenomeSeq. All other analyses were conducted in R using custom R scripts.

Correlation network construction and analysis. Microbial interactions in this study were characterized by pairwise correlations (i.e., Pearson correlation coefficient) among the abundances at the level of OTU or genus. To construct the correlation network, we first assembled the correlation matrix for each sample group based on all pairwise taxa abundance correlations. A threshold value was assigned for each correlation matrix to remove the correlations with strengths lower than the threshold for the network inference. The threshold identification is a crucial step, because using inappropriate critical thresholds can cause inaccurate structures of the inferred network. In this study, we used a Random Matrix Theory (RMT)-based approach for assigning the critical thresholds. The details of the RMT approach were documented previously (Deng et al 2012, Shi et al 2016). In brief, a system (i.e., correlation matrix) falling within the applicable range of the RMT obeys two universal laws in which if the system was system-specific and non-random, its nearest neighbor spacing distribution (NNSD) of matrix eigenvalues follows Poisson statistics; while if the system was inflated with randomness and noise, its NNSD follows the Wigner-Dyson

distribution. When the threshold value is attempted from low to high, weak and random correlations are removed from the system increasingly, so the NNSD of the system will transition from following the GOE distributions to following Poisson distributions. Therefore, such a transition point is mathematically defined and can serve as a reference point to distinguish system-specific and non-random properties of the system from random noise. Compared with methods using arbitrary thresholds or relying on empirical p-values, the RMT-based approach has the advantages of being theoretically sound, identifying the thresholds automatically and objectively, and having been demonstrated to work compatibly with network inference in biological systems, such as inferring metabolic (Luo et al 2007 #12103), protein (Luo et al 2007 #12104), and microbial ecological networks (Zhou, 2011 #12105). The RMT-based approach used in this study has the following steps for the identification of the threshold.

1. Start from the taxonomic profiling data C, which is an $m \times n$ matrix where m is the number of samples and n is the number of OTUs detected, and every quantity c_{kj} represents the count the OTU j in sample k. Meanwhile, OTUs with presence in less than 60% of the samples were excluded from further analysis.

2. Calculate the co-occurrence matrix P, which is an $n \times n$ symmetric matrix, and every quantity p_{ij} from P is the co-occurrence strength (i.e., Pearson correlation) between the OTU i and j.

3. Generate a threshold series, $s = [s_0, s_1, \ldots, s_k, \ldots, s_{max}]$, where s_0 = 0.3, $s_{max} = 1$ and $s_k - s_{k-1} = 0.01$.

4. For each s_k in every s, the following algorithm was performed for transitioning point evaluation.

i. Reduce the P to P_{s_k} in such a way that any $p_{ij} \in [-s_k, s_k]$ was set to 0.

ii. Calculate eigenvalues λ of the P_{s_k}, where λ is the eigenvalue, and transform λ to e, where $e_i = N_{av}(\lambda_i)$, and N_{av} is the unfolding function of eigenvalues.

iii. Calculate the nearest neighbor spacings (NNS) of unfolded eigenvalues, d, where $d_i = |e_{i+1} - e_i|$ for every $i = 1, 2, \dots, n-1$.

iv. Approximate the probability density of NNSD, $P(d)$, which can be treated as a standard quantity describing the level fluctuations of the system. Assess the goodness of fit of $P(d)$ for both Poisson and Wigner-Dyson statistics, which are expressed as $P(d) \approx e^{-d}$ and $P_{GOE}(d) \approx \frac{\pi}{2} \cdot d \cdot e^{(-\pi \cdot d^2/4)}$, respectively. Terminate the process and yield the threshold s_k, if $P(d)$ fits the Poisson distribution better than the Wigner-Dyson distribution, and s_k is chosen for the final threshold.

With the final threshold s_k, each correlation matrix was transformed into the corresponding adjacency matrix by: 1) setting every value in the correlation matrix to 0 if its absolute form was less than s_k, and 2) setting the value to 1 if its absolute form was greater than s_k. To this end, adjacency matrices were obtained and used for constructing all correlation networks.

Each correlation network was visualized and analyzed in Cytoscape 3.4.0. We computed the following topological indices for the analysis: node number, link/edge number, average shortest path length, clustering coefficient (Watts and Strogatz 1998), diameter (i.e., the longest length of all shortest paths), average node degree (i.e., average number of node neighbors), density (i.e., edge number divided by the number of all possible edges), centralization and heterogeneity (Dong and Horvath 2007). Putative key nodes/taxa were inferred based on: 1) whether the nodes had the most degree in the module where they were located, 2) whether the nodes had most of their edges connected to different modules, and 3) both situations. Accordingly, all taxa were assigned to one of the following roles in the network: peripherals, connectors, module hubs or network hubs

(Deng et al 2012, Olesen et al 2007, Shi et al 2016). Unique or shared taxa in each module between sample groups were identified using “Difference” or “Intersection” in the “Advanced Network Merge” interface available from “Tools” in Cytoscape 3.4.0. For the inferred networks at the level of OTU, the OTU nodes under each genus were also merged together into the single genus nodes using the “Group” then “Collapse” features provided in the menu in the Cytoscape graphics panel.

Mechanisms underlying community assembly. Five community assembly processes were evaluated. These are variable selection, homogeneous selection, dispersal limitation, homogenizing dispersal and undominated as described previously (Stegen et al 2013a, Stegen et al 2015, Webb et al 2008). “Selection” is defined as a major niche-based process which shapes community structure due to fitness differences (e.g., survival, growth, reproduction) among different organisms, including effects of abiotic conditions (environmental filtering) and biotic interactions (e.g., competition, facilitation, mutualism, predation, host filtering). “Variable selection” or “homogeneous selection” are selection processes under heterogeneous or homogeneous abiotic and biotic environmental conditions, respectively, which drive communities toward more dissimilarity or similarity, respectively. “Dispersal limitation” means that the movement to and/or establishment (colonization) of individuals in a new location is restricted, which leads to communities that are more dissimilar; while “homogenizing dispersal” means a very high rate of dispersal among communities, which homogenizes the communities to become very similar. “Undominated” is a turnover not differentiable from either phylogenetic or taxonomic null patterns, which mainly includes various stochastic processes, e.g., drift. "Drift" means random changes of community structure due to the stochastic processes of birth, death and offspring reproduction.

The relative roles of community assembly processes were quantified using a framework proposed by Stegen *et al* (Stegen et al 2013b, Stegen et al 2015). The influence of selection was estimated based on the beta nearest taxon index (*βNTI*) between communities. The pairwise turnovers between communities were first analyzed for phylogenetic dissimilarity using the beta mean nearest taxon distance (*βMNTD*, Eq. S1).

$$\beta MNTD_{ij} = \frac{1}{2}\left[\sum_{k=1}^{A} p_{ik}\min_{m}(\delta_{km}) + \sum_{m=1}^{B} p_{jm}\min_{k}(\delta_{km})\right] \qquad \text{Eq. S1}$$

where $\beta MNTD_{ij}$ is *βMNTD* between community *i* and *j*; δ_{km} is the phylogenetic distance between OTU *k* and OTU *m*; p_{ik} is the relative abundance (proportion) of OTU *k* in community *i*; p_{jm} is the relative abundance (proportion) of OTU *m* in community *j*. Then, we performed null model analysis for phylogenetic dissimilarity, where the OTU labels were shuffled across the tips of the phylogenetic tree to randomize phylogenetic relationships among OTUs, while the relative abundance of each OTU in each community was fixed as observed. This null model algorithm is called "taxa shuffle" (Kembel 2009) and was performed for 1000 times to get 1000 sets of null *βMNTD* values. Then, *βNTI* between community *i* and *j* is calculated as follows (Fine and Kembel 2011, Stegen et al 2012).

$$\beta NTI_{ij} = \frac{\beta MNTD_{obs[i,j]} - \overline{\beta MNTD_{null[i,j]}}}{sd(\beta MNTD_{null[i,j]})} \qquad \text{Eq. S2}$$

where $\beta MNTD_{obs[i,j]}$ is observed *βMNTD* between community *i* and *j*; $\overline{\beta MNTD_{null[i,j]}}$ and $sd(\beta MNTD_{null[i,j]})$ are the mean and standard deviation of the 1000 null *βMNTD* between community *i* and *j* from the null model. If the community turnover is due to neutral processes, the observed *βMNTD* should not be differentiable from the null *βMNTD* values. In contrast, if the observed *βMNTD* of a turnover was significantly higher (*βNTI*>2) or lower (*βNTI*<-2) than null

expectation, the turnover was considered to be governed by “variable selection” or “homogeneous selection” (Stegen et al 2015, Webb et al 2008).

Then, the turnovers not governed by selection (i.e., -2<*βNTI*<2) were analyzed using the modified Raup-Crick metric (RC) based on the Bray-Curtis dissimilarity index (BC) (Stegen et al 2013b) (Stegen et al 2015). The observed BC of each turnover was compared with 1000 null BC values from null model analysis. In the null model, the richness of each sample was fixed as observed, and the presence of OTUs in each null community was simulated as a random draw of OTUs from a regional species pool with probabilities proportional to their observed occurrence frequencies (Chase et al 2011). Then, the abundance of the present OTUs in each null community was simulated as a random draw of individuals into the present OTUs with probabilities proportional to their regional relative abundances (Stegen et al 2013b). After 1000-time randomization, an α value for each turnover was calculated as the percentage of null BC values lower than observed BC plus a half of percentage of null BC values equal to observed BC. Then, the RC value of each turnover was calculated by subtracting 0.5 from the α value and multiplying by 2 to make the index range from -1 to +1 (Eq. S3 and S4).

$$RC_{ij} = 2(\alpha - 0.5) = 2\frac{\sum_{t=1}^{N_{null}} \delta_{[i,j,t]}}{N_{null}} - 1 \quad \text{Eq. S3}$$

$$\delta_{[i,j,t]} = \begin{cases} 0 & BC_{null[i,j,t]} > BC_{obs[i,j]} \\ 0.5 & BC_{null[i,j,t]} = BC_{obs[i,j]} \\ 1 & BC_{null[i,j,t]} < BC_{obs[i,j]} \end{cases} \quad \text{Eq. S4}$$

where RC_{ij} is RC value between community *i* and *j*; N_{null} is the randomization times; $\delta_{[i,j,t]}$ is an operator for RC at the tth randomization; $BC_{null[i,j,t]}$ is the null BC dissimilarity between community *i* and *j* at the tth randomization; $BC_{obs[i,j]}$ is the observed BC dissimilarity between community *i* and *j*. If selection has little effect on the turnover, the major cause of the non-random

turnover pattern should be abnormal dispersal conditions. Thus, if an observed BC was significantly higher (RC>0.95) or lower (RC<-0.95) than null expectation when |*βNTI*|<2, the turnover was considered to be governed by “dispersal limitation” or “homogenizing dispersal” (Stegen et al 2013b). The remaining turnover (i.e., |*βNTI*|<2 and |RC|<0.95) that was not differentiable from null patterns was named “undominated” (Stegen et al 2015) and included other stochastic processes (e.g., drift).

After the steps described above, each pairwise turnover was assigned a governing process. Then, the relative influence of a process was estimated as the percentage of turnovers governed by this process (Stegen et al 2013b, Stegen et al 2015), shown in Eq. S5 to S9.

$$P_{VS} = \frac{\sum w_{VS[i,j]}}{n} \times 100\% \qquad w_{VS[i,j]} = \begin{cases} 1 & \beta NTI_{ij} > 2 \\ 0 & else \end{cases} \qquad \text{Eq. S5}$$

$$P_{HS} = \frac{\sum w_{HS[i,j]}}{n} \times 100\% \qquad w_{HS[i,j]} = \begin{cases} 1 & \beta NTI_{ij} < -2 \\ 0 & else \end{cases} \qquad \text{Eq. S6}$$

$$P_{DL} = \frac{\sum w_{DL[i,j]}}{n} \times 100\% \qquad w_{DL[i,j]} = \begin{cases} 1 & \left|\beta NTI_{ij}\right| < 2 \,\&\, RC_{ij} > 0.95 \\ 0 & else \end{cases} \qquad \text{Eq. S7}$$

$$P_{HD} = \frac{\sum w_{HD[i,j]}}{n} \times 100\% \qquad w_{DL[i,j]} = \begin{cases} 1 & \left|\beta NTI_{ij}\right| < 2 \,\&\, RC_{ij} < -0.95 \\ 0 & else \end{cases} \qquad \text{Eq. S8}$$

$$P_{Un} = \frac{\sum w_{Un[i,j]}}{n} \times 100\% \qquad w_{Un[i,j]} = \begin{cases} 1 & \left|\beta NTI_{ij}\right| < 2 \,\&\, \left|RC_{ij}\right| < 0.95 \\ 0 & else \end{cases} \qquad \text{Eq. S9}$$

where P_{VS}, P_{HS}, P_{DL}, P_{HD}, and P_{Un} are the process ratios to estimate the relative influence of “variable selection”, “homogeneous selection”, “dispersal limitation”, “homogenizing dispersal”, and “undominated”, respectively; $w_{VS[i,j]}$, $w_{HS[i,j]}$, $w_{DL[i,j]}$, $w_{HD[i,j]}$, and $w_{Un[i,j]}$ are the operators of the corresponding processes for the turnover between community *i* and *j*; *n* is the number of pairwise turnovers in a certain comparison within a group or between groups (treatments) of communities. The significance of such a percentage was analyzed by a permutation test in which null results were calculated by randomizing the sample names for 1000 times.

References

Ashimoto A, Chen C, Bakker I, Slots J (1996). Polymerase chain reaction detection of 8 putative periodontal pathogens in subgingival plaque of gingivitis and advanced periodontitis lesions. *Oral Microbiol Immunol* **11:** 266-273.

Armitage GC (2004). Periodontal diagnoses and classification of periodontal diseases. *Periodontology 2000* **34:** 9-21.

Ashimoto A, Chen C, Bakker I, Slots J (1996). Polymerase chain reaction detection of 8 putative periodontal pathogens in subgingival plaque of gingivitis and advanced periodontitis lesions. *Oral Microbiol Immunol* **11:** 266-273.

Baker GC, Smith JJ, Cowan DA (2003). Review and re-analysis of domain-specific 16S primers. *J Microbiol Methods* **55:** 541-555.

Caporaso JG, Kuczynski J, Stombaugh J, Bittinger K, Bushman FD, Costello EK *et al* (2010). QIIME allows analysis of high-throughput community sequencing data. *Nat Methods* **7:** 335-336.

Caporaso JG, Lauber CL, Walters WA, Berg-Lyons D, Lozupone CA, Turnbaugh PJ *et al* (2011). Global patterns of 16S rRNA diversity at a depth of millions of sequences per sample. *Proc Natl Acad Sci U S A* **108 Suppl 1:** 4516-4522.

Caporaso JG, Lauber CL, Walters WA, Berg-Lyons D, Huntley J, Fierer N *et al* (2012). Ultra-high-throughput microbial community analysis on the Illumina HiSeq and MiSeq platforms. *ISME J* **6:** 1621-1624.

Chase JM, Kraft NJB, Smith KG, Vellend M, Inouye BD (2011). Using null models to disentangle variation in community dissimilarity from variation in alpha-diversity. *Ecosphere* **2**.

Chen C, Ashimoto A, Sangsurasak S, Flynn MJ, Slots J (1997). Oral food consumption and subgingival microorganisms: Subgingival microbiota of gastrostomy tube-fed children and healthy controls. *J Periodontol* **68:** 1163-1168.

Deng Y, Jiang YH, Yang Y, He Z, Luo F, Zhou J (2012). Molecular ecological network analyses. *BMC Bioinformatics* **13:** 113.

DeSantis TZ, Hugenholtz P, Larsen N, Rojas M, Brodie EL, Keller K *et al* (2006). Greengenes, a chimera-checked 16S rRNA gene database and workbench compatible with ARB. *Appl Environ Microbiol* **72:** 5069-5072.

Dong J, Horvath S (2007). Understanding network concepts in modules. *BMC systems biology* **1:** 24.

Drucker SD, Prieto LE, Kao DW (2012). Periodontal probing calibration in an academic setting. *Journal of dental education* **76:** 1466-1473.

Edgar RC (2013). UPARSE: highly accurate OTU sequences from microbial amplicon reads. *Nat Methods* **10:** 996-998.

Fine PVA, Kembel SW (2011). Phylogenetic community structure and phylogenetic turnover across space and edaphic gradients in western Amazonian tree communities. *Ecography* **34:** 552-565.

Kembel SW (2009). Disentangling niche and neutral influences on community assembly: assessing the performance of community phylogenetic structure tests. *Ecology Letters* **12:** 949-960.

Magoc T, Salzberg SL (2011). FLASH: fast length adjustment of short reads to improve genome assemblies. *Bioinformatics* **27:** 2957-2963.

Olesen JM, Bascompte J, Dupont YL, Jordano P (2007). The modularity of pollination networks. *Proc Natl Acad Sci U S A* **104:** 19891-19896.

Parks DH, Beiko RG (2010). Identifying biologically relevant differences between metagenomic communities. *Bioinformatics* **26:** 715-721.

Parks DH, Tyson GW, Hugenholtz P, Beiko RG (2014). STAMP: statistical analysis of taxonomic and functional profiles. *Bioinformatics* **30:** 3123-3124.

Paulson JN, Stine OC, Bravo HC, Pop M (2013). Differential abundance analysis for microbial marker-gene surveys. *Nat Methods* **10:** 1200-1202.

Quinque D, Kittler R, Kayser M, Stoneking M, Nasidze I (2006). Evaluation of saliva as a source of human DNA for population and association studies. *Anal Biochem* **353:** 272-277.

Shi S, Nuccio EE, Shi ZJ, He Z, Zhou J, Firestone MK (2016). The interconnected rhizosphere: High network complexity dominates rhizosphere assemblages. *Ecol Lett* **19:** 926-936.

Stegen JC, Lin X, Konopka AE, Fredrickson JK (2012). Stochastic and deterministic assembly processes in subsurface microbial communities. *Isme Journal* **6:** 1653–1664.

Stegen JC, Lin X, Fredrickson JK, Chen X, Kennedy DW, Murray CJ *et al* (2013a). Quantifying community assembly processes and identifying features that impose them. *ISME J* **7:** 2069-2079.

Stegen JC, Lin X, Fredrickson JK, Chen X, Kennedy DW, Murray CJ *et al* (2013b). Quantifying community assembly processes and identifying features that impose them. *Isme Journal* **7:** 2069-2079.

Stegen JC, Lin X, Fredrickson JK, Konopka AE (2015). Estimating and mapping ecological processes influencing microbial community assembly. *Front Microbiol* **6:** 370.

Wang Q, Garrity GM, Tiedje JM, Cole JR (2007). Naive Bayesian classifier for rapid assignment of rRNA sequences into the new bacterial taxonomy. *Appl Environ Microbiol* **73:** 5261-5267.

Watts DJ, Strogatz SH (1998). Collective dynamics of 'small-world' networks. *Nature* **393:** 440-442.

Webb CO, Ackerly DD, Kembel SW (2008). Phylocom: software for the analysis of phylogenetic community structure and trait evolution. *Bioinformatics* **24:** 2098-2100.

Yang F, Zeng X, Ning K, Liu KL, Lo CC, Wang W *et al* (2012). Saliva microbiomes distinguish caries-active from healthy human populations. *ISME J* **6:** 1-10.
