# Supplementary material for: Oral microbiota of periodontal health and disease and their changes after nonsurgical periodontal therapy
Source: ISME J. 2018 Jan 16;12(5):1210–24. doi: 10.1038/s41396-017-0037-1 (PMC5932080; doi:10.1038/s41396-017-0037-1)
Supplement: Supplementary file 2 — Supplementary Table S1 [file 41396_2017_37_MOESM2_ESM.docx]

| **Reverse Primers** | **Sequences** |
| --- | --- |
| 806R MSP1 | CAAGCAGAAGACGGCATACGAGATTTACCGACGAGTGTGACTGGAGTTCAGACGTGTGCTCTTCCGATCTGGACTACHVGGGTWTCTAAT |
| 806R MSP2 | CAAGCAGAAGACGGCATACGAGATATTGGACACGCTGTGACTGGAGTTCAGACGTGTGCTCTTCCGATCTTGGACTACHVGGGTWTCTAAT |
| 806R MSP3 | CAAGCAGAAGACGGCATACGAGATTCGCATGGATACGTGACTGGAGTTCAGACGTGTGCTCTTCCGATCTTCGGACTACHVGGGTWTCTAAT |
| 806R MSP4 | CAAGCAGAAGACGGCATACGAGATAGCGAACCTGTTGTGACTGGAGTTCAGACGTGTGCTCTTCCGATCTCTAGGACTACHVGGGTWTCTAAT |
| 806R MSP5 | CAAGCAGAAGACGGCATACGAGATAGCTTCGACAGTGTGACTGGAGTTCAGACGTGTGCTCTTCCGATCTAGCTGGACTACHVGGGTWTCTAAT |
| 806R MSP6 | CAAGCAGAAGACGGCATACGAGATGTCAGCCGTTAAGTGACTGGAGTTCAGACGTGTGCTCTTCCGATCTACTGTGGACTACHVGGGTWTCTAAT |
| 806R MSP7 | CAAGCAGAAGACGGCATACGAGATTCCAGATAGCGTGTGACTGGAGTTCAGACGTGTGCTCTTCCGATCTCTGACGGGACTACHVGGGTWTCTAAT |
| 806R MSP8 | CAAGCAGAAGACGGCATACGAGATGAGAGTCCACTTGTGACTGGAGTTCAGACGTGTGCTCTTCCGATCTGATCGTGGGACTACHVGGGTWTCTAAT |
| 806R MSP9 | CAAGCAGAAGACGGCATACGAGATGCTCACAATGTGGTGACTGGAGTTCAGACGTGTGCTCTTCCGATCTGGACTACHVGGGTWTCTAAT |
| 806R MSP10 | CAAGCAGAAGACGGCATACGAGATTTGACGACATCGGTGACTGGAGTTCAGACGTGTGCTCTTCCGATCTTGGACTACHVGGGTWTCTAAT |
| 806R MSP11 | CAAGCAGAAGACGGCATACGAGATCTTAGAACGTGCGTGACTGGAGTTCAGACGTGTGCTCTTCCGATCTTCGGACTACHVGGGTWTCTAAT |
| 806R MSP12 | CAAGCAGAAGACGGCATACGAGATCGGTTCACATAGGTGACTGGAGTTCAGACGTGTGCTCTTCCGATCTCTAGGACTACHVGGGTWTCTAAT |
| 806R MSP13 | CAAGCAGAAGACGGCATACGAGATCGATAGGCCTTAGTGACTGGAGTTCAGACGTGTGCTCTTCCGATCTAGCTGGACTACHVGGGTWTCTAAT |
| 806R MSP14 | CAAGCAGAAGACGGCATACGAGATGCTATATCCAGGGTGACTGGAGTTCAGACGTGTGCTCTTCCGATCTACTGTGGACTACHVGGGTWTCTAAT |
| 806R MSP15 | CAAGCAGAAGACGGCATACGAGATGTCTTCAGCAAGGTGACTGGAGTTCAGACGTGTGCTCTTCCGATCTCTGACGGGACTACHVGGGTWTCTAAT |
| 806R MSP16 | CAAGCAGAAGACGGCATACGAGATTAGACACCGTGTGTGACTGGAGTTCAGACGTGTGCTCTTCCGATCTGATCGTGGGACTACHVGGGTWTCTAAT |
| 806R MSP17 | CAAGCAGAAGACGGCATACGAGATTCAGCTGACTAGGTGACTGGAGTTCAGACGTGTGCTCTTCCGATCTGGACTACHVGGGTWTCTAAT |
| 806R MSP18 | CAAGCAGAAGACGGCATACGAGATTAAGTCGGCCTAGTGACTGGAGTTCAGACGTGTGCTCTTCCGATCTTGGACTACHVGGGTWTCTAAT |
| 806R MSP19 | CAAGCAGAAGACGGCATACGAGATGCTCCTTAGAAGGTGACTGGAGTTCAGACGTGTGCTCTTCCGATCTTCGGACTACHVGGGTWTCTAAT |
| 806R MSP20 | CAAGCAGAAGACGGCATACGAGATATGGCCTGACTAGTGACTGGAGTTCAGACGTGTGCTCTTCCGATCTCTAGGACTACHVGGGTWTCTAAT |
| 806R MSP21 | CAAGCAGAAGACGGCATACGAGATTTGCAAGTACCGGTGACTGGAGTTCAGACGTGTGCTCTTCCGATCTAGCTGGACTACHVGGGTWTCTAAT |
| 806R MSP22 | CAAGCAGAAGACGGCATACGAGATCCTAGTAAGCTGGTGACTGGAGTTCAGACGTGTGCTCTTCCGATCTACTGTGGACTACHVGGGTWTCTAAT |
| 806R MSP23 | CAAGCAGAAGACGGCATACGAGATCTAGGATCACTGGTGACTGGAGTTCAGACGTGTGCTCTTCCGATCTCTGACGGGACTACHVGGGTWTCTAAT |
| 806R MSP24 | CAAGCAGAAGACGGCATACGAGATTATGAACGTCCGGTGACTGGAGTTCAGACGTGTGCTCTTCCGATCTGATCGTGGGACTACHVGGGTWTCTAAT |
| 806R MSP25 | CAAGCAGAAGACGGCATACGAGATCTTGTGCGACAAGTGACTGGAGTTCAGACGTGTGCTCTTCCGATCTGGACTACHVGGGTWTCTAAT |
| 806R MSP26 | CAAGCAGAAGACGGCATACGAGATCACGATGGTCATGTGACTGGAGTTCAGACGTGTGCTCTTCCGATCTTGGACTACHVGGGTWTCTAAT |
| 806R MSP27 | CAAGCAGAAGACGGCATACGAGATACGTGCCTTAGAGTGACTGGAGTTCAGACGTGTGCTCTTCCGATCTTCGGACTACHVGGGTWTCTAAT |
| 806R MSP28 | CAAGCAGAAGACGGCATACGAGATTGAACTAGCGTCGTGACTGGAGTTCAGACGTGTGCTCTTCCGATCTCTAGGACTACHVGGGTWTCTAAT |
| 806R MSP29 | CAAGCAGAAGACGGCATACGAGATTATTCAGCGGACGTGACTGGAGTTCAGACGTGTGCTCTTCCGATCTAGCTGGACTACHVGGGTWTCTAAT |
| 806R MSP30 | CAAGCAGAAGACGGCATACGAGATTAATCGGTGCCAGTGACTGGAGTTCAGACGTGTGCTCTTCCGATCTACTGTGGACTACHVGGGTWTCTAAT |
| 806R MSP31 | CAAGCAGAAGACGGCATACGAGATGCGTCCATGAATGTGACTGGAGTTCAGACGTGTGCTCTTCCGATCTCTGACGGGACTACHVGGGTWTCTAAT |
| 806R MSP32 | CAAGCAGAAGACGGCATACGAGATCGTAAGATGCCTGTGACTGGAGTTCAGACGTGTGCTCTTCCGATCTGATCGTGGGACTACHVGGGTWTCTAAT |
| 806R MSP33 | CAAGCAGAAGACGGCATACGAGATCTGTTACAGCGAGTGACTGGAGTTCAGACGTGTGCTCTTCCGATCTGGACTACHVGGGTWTCTAAT |
| 806R MSP34 | CAAGCAGAAGACGGCATACGAGATACGATCATCTGGGTGACTGGAGTTCAGACGTGTGCTCTTCCGATCTTGGACTACHVGGGTWTCTAAT |
| 806R MSP35 | CAAGCAGAAGACGGCATACGAGATGTAACGGCTCTAGTGACTGGAGTTCAGACGTGTGCTCTTCCGATCTTCGGACTACHVGGGTWTCTAAT |
| 806R MSP36 | CAAGCAGAAGACGGCATACGAGATCCATGCTTAGAGGTGACTGGAGTTCAGACGTGTGCTCTTCCGATCTCTAGGACTACHVGGGTWTCTAAT |
| 806R MSP37 | CAAGCAGAAGACGGCATACGAGATGTACGCACAGTTGTGACTGGAGTTCAGACGTGTGCTCTTCCGATCTAGCTGGACTACHVGGGTWTCTAAT |
| 806R MSP38 | CAAGCAGAAGACGGCATACGAGATTTAGAGCCATGCGTGACTGGAGTTCAGACGTGTGCTCTTCCGATCTACTGTGGACTACHVGGGTWTCTAAT |
| 806R MSP39 | CAAGCAGAAGACGGCATACGAGATATAAGGTCGCCTGTGACTGGAGTTCAGACGTGTGCTCTTCCGATCTCTGACGGGACTACHVGGGTWTCTAAT |
| 806R MSP40 | CAAGCAGAAGACGGCATACGAGATAGTGGCACTATCGTGACTGGAGTTCAGACGTGTGCTCTTCCGATCTGATCGTGGGACTACHVGGGTWTCTAAT |
| 806R MSP41 | CAAGCAGAAGACGGCATACGAGATCCAGAAGTGTTCGTGACTGGAGTTCAGACGTGTGCTCTTCCGATCTGGACTACHVGGGTWTCTAAT |
| 806R MSP42 | CAAGCAGAAGACGGCATACGAGATCTACTAGCGGTAGTGACTGGAGTTCAGACGTGTGCTCTTCCGATCTTGGACTACHVGGGTWTCTAAT |
| 806R MSP43 | CAAGCAGAAGACGGCATACGAGATTAGCGTTCCAGAGTGACTGGAGTTCAGACGTGTGCTCTTCCGATCTTCGGACTACHVGGGTWTCTAAT |
| 806R MSP44 | CAAGCAGAAGACGGCATACGAGATGTGAGTCATACCGTGACTGGAGTTCAGACGTGTGCTCTTCCGATCTCTAGGACTACHVGGGTWTCTAAT |
| 806R MSP45 | CAAGCAGAAGACGGCATACGAGATTGGTCCTACAAGGTGACTGGAGTTCAGACGTGTGCTCTTCCGATCTAGCTGGACTACHVGGGTWTCTAAT |
| 806R MSP46 | CAAGCAGAAGACGGCATACGAGATTACGCGTACAGTGTGACTGGAGTTCAGACGTGTGCTCTTCCGATCTACTGTGGACTACHVGGGTWTCTAAT |
| 806R MSP47 | CAAGCAGAAGACGGCATACGAGATGAGCCATCTGTAGTGACTGGAGTTCAGACGTGTGCTCTTCCGATCTCTGACGGGACTACHVGGGTWTCTAAT |
| 806R MSP48 | CAAGCAGAAGACGGCATACGAGATCGTCCGTATGAAGTGACTGGAGTTCAGACGTGTGCTCTTCCGATCTGATCGTGGGACTACHVGGGTWTCTAAT |
| 806R MSP49 | CAAGCAGAAGACGGCATACGAGATGATACGTTCGCAGTGACTGGAGTTCAGACGTGTGCTCTTCCGATCTGGACTACHVGGGTWTCTAAT |
| 806R MSP50 | CAAGCAGAAGACGGCATACGAGATCAGCTGGTTCAAGTGACTGGAGTTCAGACGTGTGCTCTTCCGATCTTGGACTACHVGGGTWTCTAAT |
| 806R MSP51 | CAAGCAGAAGACGGCATACGAGATTTAAGCGCCTGAGTGACTGGAGTTCAGACGTGTGCTCTTCCGATCTTCGGACTACHVGGGTWTCTAAT |
| 806R MSP52 | CAAGCAGAAGACGGCATACGAGATCCTGCGAAGTATGTGACTGGAGTTCAGACGTGTGCTCTTCCGATCTCTAGGACTACHVGGGTWTCTAAT |
| 806R MSP53 | CAAGCAGAAGACGGCATACGAGATTTGTAGCCGACAGTGACTGGAGTTCAGACGTGTGCTCTTCCGATCTAGCTGGACTACHVGGGTWTCTAAT |
| 806R MSP54 | CAAGCAGAAGACGGCATACGAGATTCTGTAGAGCCAGTGACTGGAGTTCAGACGTGTGCTCTTCCGATCTACTGTGGACTACHVGGGTWTCTAAT |
| 806R MSP55 | CAAGCAGAAGACGGCATACGAGATCTATTAAGCGGCGTGACTGGAGTTCAGACGTGTGCTCTTCCGATCTCTGACGGGACTACHVGGGTWTCTAAT |
| 806R MSP56 | CAAGCAGAAGACGGCATACGAGATCTCTGAGGTAACGTGACTGGAGTTCAGACGTGTGCTCTTCCGATCTGATCGTGGGACTACHVGGGTWTCTAAT |
| 806R MSP57 | CAAGCAGAAGACGGCATACGAGATCAGGATTCGTACGTGACTGGAGTTCAGACGTGTGCTCTTCCGATCTGGACTACHVGGGTWTCTAAT |
| 806R MSP58 | CAAGCAGAAGACGGCATACGAGATTCACTGCTAGGAGTGACTGGAGTTCAGACGTGTGCTCTTCCGATCTTGGACTACHVGGGTWTCTAAT |
| 806R MSP59 | CAAGCAGAAGACGGCATACGAGATACATGTCACGTGGTGACTGGAGTTCAGACGTGTGCTCTTCCGATCTTCGGACTACHVGGGTWTCTAAT |
| 806R MSP60 | CAAGCAGAAGACGGCATACGAGATATTCTGCCGAAGGTGACTGGAGTTCAGACGTGTGCTCTTCCGATCTCTAGGACTACHVGGGTWTCTAAT |
| 806R MSP61 | CAAGCAGAAGACGGCATACGAGATTACACGCTGATGGTGACTGGAGTTCAGACGTGTGCTCTTCCGATCTAGCTGGACTACHVGGGTWTCTAAT |
| 806R MSP62 | CAAGCAGAAGACGGCATACGAGATTGCATACACTGGGTGACTGGAGTTCAGACGTGTGCTCTTCCGATCTACTGTGGACTACHVGGGTWTCTAAT |
| 806R MSP63 | CAAGCAGAAGACGGCATACGAGATACGCAATGTCTGGTGACTGGAGTTCAGACGTGTGCTCTTCCGATCTCTGACGGGACTACHVGGGTWTCTAAT |
| 806R MSP64 | CAAGCAGAAGACGGCATACGAGATGCTCGAAGATTCGTGACTGGAGTTCAGACGTGTGCTCTTCCGATCTGATCGTGGGACTACHVGGGTWTCTAAT |
| 806R MSP65 | CAAGCAGAAGACGGCATACGAGATAGACGTTGCTACGTGACTGGAGTTCAGACGTGTGCTCTTCCGATCTGGACTACHVGGGTWTCTAAT |
| 806R MSP66 | CAAGCAGAAGACGGCATACGAGATTAGAGCTGCCATGTGACTGGAGTTCAGACGTGTGCTCTTCCGATCTTGGACTACHVGGGTWTCTAAT |
| 806R MSP67 | CAAGCAGAAGACGGCATACGAGATGGTAACCTCTGAGTGACTGGAGTTCAGACGTGTGCTCTTCCGATCTTCGGACTACHVGGGTWTCTAAT |
| 806R MSP68 | CAAGCAGAAGACGGCATACGAGATGACTTCATGCGAGTGACTGGAGTTCAGACGTGTGCTCTTCCGATCTCTAGGACTACHVGGGTWTCTAAT |
| 806R MSP69 | CAAGCAGAAGACGGCATACGAGATCTGCATACTGAGGTGACTGGAGTTCAGACGTGTGCTCTTCCGATCTAGCTGGACTACHVGGGTWTCTAAT |
| 806R MSP70 | CAAGCAGAAGACGGCATACGAGATTAAGGCATCGCTGTGACTGGAGTTCAGACGTGTGCTCTTCCGATCTACTGTGGACTACHVGGGTWTCTAAT |
| 806R MSP71 | CAAGCAGAAGACGGCATACGAGATAGTATTCGCGCAGTGACTGGAGTTCAGACGTGTGCTCTTCCGATCTCTGACGGGACTACHVGGGTWTCTAAT |
| 806R MSP72 | CAAGCAGAAGACGGCATACGAGATTTCGCAGATACGGTGACTGGAGTTCAGACGTGTGCTCTTCCGATCTGATCGTGGGACTACHVGGGTWTCTAAT |
| 806R MSP73 | CAAGCAGAAGACGGCATACGAGATGCACCTGTTGAAGTGACTGGAGTTCAGACGTGTGCTCTTCCGATCTGGACTACHVGGGTWTCTAAT |
| 806R MSP74 | CAAGCAGAAGACGGCATACGAGATCTCATGGTAGCAGTGACTGGAGTTCAGACGTGTGCTCTTCCGATCTTGGACTACHVGGGTWTCTAAT |
| 806R MSP75 | CAAGCAGAAGACGGCATACGAGATACTAGTTGGACCGTGACTGGAGTTCAGACGTGTGCTCTTCCGATCTTCGGACTACHVGGGTWTCTAAT |
| 806R MSP76 | CAAGCAGAAGACGGCATACGAGATGCGGACTATTCAGTGACTGGAGTTCAGACGTGTGCTCTTCCGATCTCTAGGACTACHVGGGTWTCTAAT |
| 806R MSP77 | CAAGCAGAAGACGGCATACGAGATATCGCTTAAGGCGTGACTGGAGTTCAGACGTGTGCTCTTCCGATCTAGCTGGACTACHVGGGTWTCTAAT |
| 806R MSP78 | CAAGCAGAAGACGGCATACGAGATTCAGGACGTATCGTGACTGGAGTTCAGACGTGTGCTCTTCCGATCTACTGTGGACTACHVGGGTWTCTAAT |
| 806R MSP79 | CAAGCAGAAGACGGCATACGAGATGCATTACTGGACGTGACTGGAGTTCAGACGTGTGCTCTTCCGATCTCTGACGGGACTACHVGGGTWTCTAAT |
| 806R MSP80 | CAAGCAGAAGACGGCATACGAGATGCTATGGAACTCGTGACTGGAGTTCAGACGTGTGCTCTTCCGATCTGATCGTGGGACTACHVGGGTWTCTAAT |
| 806R MSP81 | CAAGCAGAAGACGGCATACGAGATGATTGTGCAACCGTGACTGGAGTTCAGACGTGTGCTCTTCCGATCTGGACTACHVGGGTWTCTAAT |
| 806R MSP82 | CAAGCAGAAGACGGCATACGAGATAGCCTCATGATGGTGACTGGAGTTCAGACGTGTGCTCTTCCGATCTTGGACTACHVGGGTWTCTAAT |
| 806R MSP83 | CAAGCAGAAGACGGCATACGAGATAACTCCTGTGGAGTGACTGGAGTTCAGACGTGTGCTCTTCCGATCTTCGGACTACHVGGGTWTCTAAT |
| 806R MSP84 | CAAGCAGAAGACGGCATACGAGATTAGAAGGCTCCTGTGACTGGAGTTCAGACGTGTGCTCTTCCGATCTCTAGGACTACHVGGGTWTCTAAT |
| 806R MSP85 | CAAGCAGAAGACGGCATACGAGATGACTAGTCAGCTGTGACTGGAGTTCAGACGTGTGCTCTTCCGATCTAGCTGGACTACHVGGGTWTCTAAT |
| 806R MSP86 | CAAGCAGAAGACGGCATACGAGATGGATACTCGCATGTGACTGGAGTTCAGACGTGTGCTCTTCCGATCTACTGTGGACTACHVGGGTWTCTAAT |
| 806R MSP87 | CAAGCAGAAGACGGCATACGAGATCCGACATTGTAGGTGACTGGAGTTCAGACGTGTGCTCTTCCGATCTCTGACGGGACTACHVGGGTWTCTAAT |
| 806R MSP88 | CAAGCAGAAGACGGCATACGAGATTCGTGACGCTAAGTGACTGGAGTTCAGACGTGTGCTCTTCCGATCTGATCGTGGGACTACHVGGGTWTCTAAT |
| 806R MSP89 | CAAGCAGAAGACGGCATACGAGATGGCCTATAAGTCGTGACTGGAGTTCAGACGTGTGCTCTTCCGATCTGGACTACHVGGGTWTCTAAT |
| 806R MSP90 | CAAGCAGAAGACGGCATACGAGATGTAGCACTCATGGTGACTGGAGTTCAGACGTGTGCTCTTCCGATCTTGGACTACHVGGGTWTCTAAT |
| 806R MSP91 | CAAGCAGAAGACGGCATACGAGATCTAAGACGTCGTGTGACTGGAGTTCAGACGTGTGCTCTTCCGATCTTCGGACTACHVGGGTWTCTAAT |
| 806R MSP92 | CAAGCAGAAGACGGCATACGAGATCGTGCACAATTGGTGACTGGAGTTCAGACGTGTGCTCTTCCGATCTCTAGGACTACHVGGGTWTCTAAT |
| 806R MSP93 | CAAGCAGAAGACGGCATACGAGATTGTAACGCCGATGTGACTGGAGTTCAGACGTGTGCTCTTCCGATCTAGCTGGACTACHVGGGTWTCTAAT |
| 806R MSP94 | CAAGCAGAAGACGGCATACGAGATATGCGAGACTTCGTGACTGGAGTTCAGACGTGTGCTCTTCCGATCTACTGTGGACTACHVGGGTWTCTAAT |
| 806R MSP95 | CAAGCAGAAGACGGCATACGAGATCCGTCAAGATGTGTGACTGGAGTTCAGACGTGTGCTCTTCCGATCTCTGACGGGACTACHVGGGTWTCTAAT |
| 806R MSP96 | CAAGCAGAAGACGGCATACGAGATTAGTAGCACCTGGTGACTGGAGTTCAGACGTGTGCTCTTCCGATCTGATCGTGGGACTACHVGGGTWTCTAAT |
|  |  |
| **Forward primers** | **Sequences** |
| 515F SP1 | AATGATACGGCGACCACCGAGATCTACACTCTTTCCCTACACGACGCTCTTCCGATCTAGACATAGTGCCAGCMGCCGCGGTAA |
| 515F SP2 | AATGATACGGCGACCACCGAGATCTACACTCTTTCCCTACACGACGCTCTTCCGATCTTACATAGTGCCAGCMGCCGCGGTAA |
| 515F SP3 | AATGATACGGCGACCACCGAGATCTACACTCTTTCCCTACACGACGCTCTTCCGATCTCTATAGTGCCAGCMGCCGCGGTAA |
| 515F SP4 | AATGATACGGCGACCACCGAGATCTACACTCTTTCCCTACACGACGCTCTTCCGATCTGCTAGTGCCAGCMGCCGCGGTAA |
| 515F SP5 | AATGATACGGCGACCACCGAGATCTACACTCTTTCCCTACACGACGCTCTTCCGATCTCACGTGCCAGCMGCCGCGGTAA |
| 515F SP6 | AATGATACGGCGACCACCGAGATCTACACTCTTTCCCTACACGACGCTCTTCCGATCTACGTGCCAGCMGCCGCGGTAA |
| 515F SP7 | AATGATACGGCGACCACCGAGATCTACACTCTTTCCCTACACGACGCTCTTCCGATCTTGTGCCAGCMGCCGCGGTAA |
| 515F SP8 | AATGATACGGCGACCACCGAGATCTACACTCTTTCCCTACACGACGCTCTTCCGATCTGTGCCAGCMGCCGCGGTAA |
