# Supplementary material for: Oral microbiota of periodontal health and disease and their changes after nonsurgical periodontal therapy
Source: ISME J. 2018 Jan 16;12(5):1210–24. doi: 10.1038/s41396-017-0037-1 (PMC5932080; doi:10.1038/s41396-017-0037-1)
Supplement: Supplementary file 3 — Supplementary Table S2 [file 41396_2017_37_MOESM3_ESM.docx]

Supplementary Table S2. Characteristics of the study subjects and the samples

| Subject Group | No. of Subjects (male, female) | Mean Age in Yr. (range) | Disease Classification (no. of subjects) | Baseline (sample designation) | | Post-Treatment (sample designation) | |
| --- | --- | --- | --- | --- | --- | --- | --- |
|  |  |  |  | plaque samples | saliva samples | plaque samples | saliva samples |
| Healthy | 21 (15, 6) | 31.9 (19-79) | healthy (21) | 42 (HP) | 18 (HS) | N/A | N/A |
| Diseased | 48 (23, 25) | 52.0 (25-79) | generalized mild (3); generalized mild and localized moderate (6); generalized moderate (1); generalized moderate and localized severe (27); generalized severe (11) | 96 (D1P) | 45 (D1S) | 19 (D2P) | 18 (D2S) |

HP, healthy plaque; HS, healthy saliva; D1P, diseased/pre-treatment plaque; D1S, diseased/pre-treatment saliva; D2P, diseased/post-treatment plaque; D2S, diseased/post-treatment saliva.
