# Supplementary material for: Oral microbiota of periodontal health and disease and their changes after nonsurgical periodontal therapy
Source: ISME J. 2018 Jan 16;12(5):1210–24. doi: 10.1038/s41396-017-0037-1 (PMC5932080; doi:10.1038/s41396-017-0037-1)
Supplement: Supplementary file 4 — Supplementary Table S3 [file 41396_2017_37_MOESM4_ESM.docx]

Supplementary Table S3. Alpha diversity of saliva and subgingival plaque samples^a^

|  | D1P | D2P | HP |
| --- | --- | --- | --- |
| D1P | - | - | - |
| D2P | 0.1096 | - | - |
| HP | 0.7614 | 0.1023 | - |
|  |  |  |  |
|  | D1S | D2S | HS |
| D1S | - | - | - |
| D2S | 0.07785 | - | - |
| HS | 2.572E-5^b^ | 0.03078^b^ | - |

^a^D1P, diseased/pre-treatment plaque; D2P, diseased/post-treatment plaque; HP, healthy plaque; D1S, diseased/pre-treatment saliva; D2S, diseased/post-treatment saliva; HS, healthy saliva. ^b^Statistically significant difference between mean values by Welch's two-sample *t*-test at *p*<0.01.
