# Supplementary material for: Oral microbiota of periodontal health and disease and their changes after nonsurgical periodontal therapy
Source: ISME J. 2018 Jan 16;12(5):1210–24. doi: 10.1038/s41396-017-0037-1 (PMC5932080; doi:10.1038/s41396-017-0037-1)
Supplement: Supplementary file 5 — Supplementary Table S4 [file 41396_2017_37_MOESM5_ESM.docx]

Supplementary Table S4. Dissimilarity analysis of plaque vs. saliva and between plaque treatment states

| Group 1^a^ | Group 2^a^ | MRPP (Delta) | *p* | Anosim (R) | *p*-Value | Adonis (F) | *p* |
| --- | --- | --- | --- | --- | --- | --- | --- |
| Subgingival Plaque vs. Saliva | | | | | | | |
| D1P | D1S | 0.640 | 0.001 | 0.581 | 0.001 | 29.922 | 0.001 |
| D2P | D2S | 0.630 | 0.001 | 0.754 | 0.001 | 13.241 | 0.001 |
| HP | HS | 0.597 | 0.001 | 0.805 | 0.001 | 22.695 | 0.001 |
| Subgingival Plaque Only | | | | | | | |
| D1P | D2P | 0.649 | 0.105 | 0.041 | 0.275 | 1.575 | 0.083 |
| D1P | HP | 0.640 | 0.001 | 0.215 | 0.001 | 11.937 | 0.001 |
| D2P | HP | 0.634 | 0.002 | 0.198 | 0.002 | 3.748 | 0.001 |

MRPP, multi-response permutation procedure.

^a^D1P, diseased/pre-treatment plaque; D1S, diseased/pre-treatment saliva; D2P, diseased/post-treatment plaque; D2S, diseased/post-treatment saliva; HP, healthy plaque; HS, healthy saliva.
