# Supplementary material for: Oral microbiota of periodontal health and disease and their changes after nonsurgical periodontal therapy
Source: ISME J. 2018 Jan 16;12(5):1210–24. doi: 10.1038/s41396-017-0037-1 (PMC5932080; doi:10.1038/s41396-017-0037-1)
Supplement: Supplementary file 6 — Supplementary Table S5 [file 41396_2017_37_MOESM6_ESM.docx]

Supplementary Table S5. Unfiltered differential abundance of OTUs in different sample groups

| Overabundant in Subgingival Plaque of Subjects with Chronic Periodontitis | | | | | | | |
| --- | --- | --- | --- | --- | --- | --- | --- |
| OTU^a^ | | Taxon | Abundance Ratio | | | Adjusted *p-*Value^b^ | |
|  |  |  | Pre-Treatment/Healthy | | Post-Treatment/Healthy |  |  |
| OTU_150 | | *Bacteroidetes* | 4.68 | | -0.43 | 1.29E-38 | |
| OTU_90 | | *Peptostreptococcaceae* | 4.29 | | 1.08 | 1.72E-13 | |
| OTU_13* | | *Filifactor* | 3.51 | | 1.96 | 1.66E-09 | |
| OTU_73* | | *Desulfobulbus* | 3.45 | | 1.93 | 6.29E-13 | |
| OTU_94 | | *Pyramidobacter* | 3.44 | | 1.83 | 2.61E-08 | |
| OTU_119 | | *Desulfomicrobium* | 3.39 | | 0.50 | 5.55E-18 | |
| OTU_75* | | *Eubacterium* | 3.37 | | 1.52 | 5.76E-16 | |
| OTU_137* | | *Hallella* | 3.34 | | 1.06 | 1.44E-13 | |
| OTU_142 | | *Clostridia* | 3.17 | | 0.53 | 2.39E-13 | |
| OTU_4* | | *Porphyromonas* | 3.16 | | 1.33 | 6.57E-06 | |
| OTU_63* | | *Phocaeicola* | 3.10 | | 1.22 | 8.52E-08 | |
| OTU_12* | | *Tannerella* | 3.10 | | 1.53 | 6.79E-09 | |
| OTU_111* | | Unclassified (*Bacteroidetes*) | 3.07 | | 1.26 | 6.69E-11 | |
| OTU_55* | | *Alloprevotella* | 3.05 | | 3.46 | 1.62E-08 | |
| OTU_194 | | Unclassified Bacteria sp. | 3.02 | | 0.00 | 2.68E-20 | |
| OTU_8* | | *Porphyromonas* | 3.02 | | 2.17 | 9.20E-08 | |
| OTU_25* | | Unclassified (*Firmicutes*) | 2.96 | | 1.39 | 2.12E-08 | |
| OTU_58* | | Unclassified (*Firmicutes*) | 2.93 | | 2.20 | 2.11E-11 | |
| OTU_66* | | Unclassified (*Firmicutes*) | 2.92 | | 1.33 | 2.18E-10 | |
| OTU_39 | | *Leptotrichiaceae* | 2.90 | | -0.14 | 4.15E-12 | |
| OTU_110* | | *Johnsonella* | 2.89 | | 1.70 | 1.16E-11 | |
| OTU_106 | | *Mollicutes* | 2.83 | | 2.61 | 1.02E-07 | |
| OTU_172 | | *Lachnospiraceae* | 2.80 | | 0.22 | 2.46E-14 | |
| OTU_109* | | Unclassified (*Firmicutes*) | 2.73 | | 2.25 | 1.75E-15 | |
| OTU_211 | | *Wolinella* | 2.50 | | -0.49 | 1.41E-21 | |
| OTU_144 | | *Lachnospiraceae* | 2.44 | | -0.35 | 2.68E-08 | |
| OTU_1773* | | *Treponema* | 2.38 | | 0.39 | 1.06E-06 | |
| OTU_74* | | *Eubacterium* | 2.35 | | 0.86 | 2.08E-10 | |
| OTU_85 | | *Prevotella* | 2.35 | | 0.47 | 1.01E-06 | |
| OTU_129* | | *Treponema* | 2.24 | | 0.73 | 9.98E-06 | |
| OTU_135 | | *Treponema* | 2.23 | | 1.78 | 1.65E-08 | |
| OTU_147* | | *Treponema* | 2.23 | | 2.00 | 1.47E-07 | |
| OTU_171 | | *Stomatobaculum* | 2.22 | | 1.03 | 3.46E-08 | |
| OTU_178 | | *Peptostreptococcaceae* | 2.21 | | 0.62 | 5.34E-06 | |
| OTU_606* | | *Treponema* | 2.19 | | 1.32 | 3.19E-06 | |
| OTU_131* | | *Prevotella* | 2.19 | | 3.19 | 1.32E-08 | |
| OTU_114 | | *Bacteroidetes* | 2.17 | | 1.20 | 3.06E-06 | |
| OTU_161* | | *Peptostreptococcaceae* | 2.15 | | 0.14 | 1.74E-11 | |
| OTU_62* | | *Mycoplasma* | 2.14 | | 0.75 | 3.24E-09 | |
| OTU_72* | | *Leptotrichia* | 2.14 | | 2.19 | 2.67E-06 | |
| OTU_208 | | *Erysipelothrichaceae* | 2.13 | | 0.20 | 2.06E-10 | |
| OTU_101* | | *Treponema* | 2.12 | | 1.18 | 5.40E-09 | |
| OTU_615* | | *Treponema* | 2.11 | | 0.86 | 1.18E-08 | |
| OTU_207 | | *Clostridia* | 2.06 | | 0.98 | 1.61E-07 | |
| OTU_97* | | *Mogibacterium* | 2.06 | | 0.90 | 4.84E-09 | |
| OTU_33* | | *Treponema* | 2.06 | | 2.03 | 2.38E-05 | |
| OTU_249 | | *Treponema* | 2.01 | | 0.56 | 2.04E-11 | |
| Overabundant in Subgingival Plaque of Periodontally Healthy Subjects | | | | | | | |
| OTU^a^ | Taxon | | Abundance Ratio | | | | Adjusted *p-*Value^b^ |
|  |  |  | Healthy/Pre-treatment | Healthy/Post-treatment | | |  |
| OTU_180 | *Lachnoanaerobaculum* | | 3.80 | 4.35 | | | 6.93E-15 |
| OTU_185 | *Capnocytophaga* | | 3.59 | 4.36 | | | 3.24E-11 |
| OTU_209 | *Actinomycetales* | | 3.36 | 2.49 | | | 4.24E-29 |
| OTU_98* | *Exiguobacterium* | | 2.90 | 2.71 | | | 2.15E-16 |
| OTU_146 | *Ottowia* | | 2.86 | 2.41 | | | 0.000459 |
| OTU_335* | *Actinomyces* | | 2.73 | 4.33 | | | 1.12E-07 |
| OTU_216 | *Dysgonomonas* | | 2.57 | 2.13 | | | 9.62E-23 |
| OTU_7* | *Veillonella* | | 2.52 | 2.31 | | | 2.86E-08 |
| OTU_304 | *Skermanella* | | 2.50 | 3.14 | | | 2.12E-31 |
| OTU_159* | *Paludibacter* | | 2.45 | 1.02 | | | 2.22E-16 |
| OTU_550* | *Capnocytophaga* | | 2.44 | 1.38 | | | 5.77E-09 |
| OTU_15* | *Actinomyces* | | 2.43 | 1.93 | | | 5.43E-09 |
| OTU_5* | *Corynebacterium* | | 2.38 | 1.65 | | | 1.79E-06 |
| OTU_221 | *Rhizobiales* | | 2.37 | 1.84 | | | 7.91E-26 |
| OTU_362 | *Rhizobiales* | | 2.35 | 2.54 | | | 1.52E-44 |
| OTU_262 | *Desulfovibrio* | | 2.35 | 2.40 | | | 5.33E-14 |
| OTU_217 | *Opitutus* | | 2.33 | 2.30 | | | 5.44E-20 |
| OTU_363 | *Spartobacteria* | | 2.31 | 2.86 | | | 7.10E-29 |
| OTU_341 | *Solirubtobacter* | | 2.30 | 3.01 | | | 7.82E-27 |
| OTU_91* | *Prevotella* | | 2.28 | 1.67 | | | 4.69E-07 |
| OTU_165 | *Clostridium* | | 2.28 | 1.16 | | | 3.28E-14 |
| OTU_204 | *Clostridia* | | 2.17 | 1.16 | | | 2.30E-16 |
| OTU_68* | *Leptotrichia* | | 2.17 | 2.05 | | | 9.26E-07 |
| OTU_1327* | *Veillonella* | | 2.15 | 2.02 | | | 1.98E-07 |
| OTU_248 | *Lachnospiraceae* | | 2.10 | 2.75 | | | 4.94E-11 |
| OTU_700 | *Spartobacteria* | | 2.09 | 2.71 | | | 2.25E-28 |
| OTU_291 | *Acidobacteria* | | 2.08 | 3.06 | | | 3.83E-16 |
| OTU_1763 | *Acidobacteria* | | 2.08 | 2.63 | | | 1.12E-27 |
| OTU_236 | *Acidobacteria* | | 2.07 | 3.22 | | | 9.81E-15 |
| OTU_1274* | *Leptotrichia* | | 2.07 | 2.44 | | | 2.93E-07 |
| OTU_883 | *Acidobacteria* | | 2.06 | 2.33 | | | 2.89E-26 |
| OTU_206* | *Opitutus* | | 2.04 | 2.68 | | | 1.91E-15 |
| OTU_894 | *Rhizobiales* | | 2.03 | 2.47 | | | 1.25E-22 |
| OTU_474 | *Conexibacter* | | 2.00 | 2.45 | | | 1.21E-31 |
| Overabundant in Saliva of Subjects with Chronic Periodontitis | | | | | | | |
| **OTU**^a^ | Taxon | | Abundance Ratio | | | | Adjusted *p-*Value^b^ |
|  |  |  | Pre-treatment/Healthy | Post-treatment/Healthy | | |  |
| OTU_75 | *Eubacterium* | | 3.32 | 2.55 | | | 0.000271 |
| OTU_85 | *Prevotella* | | 2.73 | 2.10 | | | 0.012844 |
| OTU_4* | *Porphyromonas* | | 2.70 | 1.79 | | | 0.02179 |
| OTU_148 | *Pseudoramibacter* | | 2.51 | 2.23 | | | 0.000478 |
| OTU_12* | *Tannerella* | | 2.17 | 1.63 | | | 0.001173 |
| OTU_73* | *Desulfobulbus* | | 2.13 | 1.20 | | | 0.000271 |
| OTU_74* | *Eubacterium* | | 2.09 | 1.86 | | | 0.013454 |
| OTU_63* | *Bacteroidaceae* | | 2.08 | 0.40 | | | 0.001862 |
| OTU_97* | *Mogibacterium* | | 2.05 | 2.42 | | | 0.01061 |
| Overabundant in Saliva of Periodontally Healthy Subjects | | | | | | | |
| OTU^a^ | Taxon | | Abundance Ratio | | | | Adjusted *p-*Value^b^ |
|  |  |  | Healthy/Pre-treatment | Healthy/Post-treatment | | |  |
| OTU_219 | *Peptostreptococcaceae* | | 2.48 | 2.58 | | | 1.09E-06 |
| OTU_57* | *Prevotella* | | 2.33 | 2.35 | | | 0.00377 |

^a, *^Indicates the OTU contains at least half the effective sample size of all OTUs.

^b^All with *p*<0.05.

Analysis was conducted in metagenomeSeq after removing OTUs that contained <15 total occurrences across all samples. The linear model used for the zero-inflated Gaussian (ZIG) fit was ~Treatment + normFactor.
