# Supplementary material for: Oral microbiota of periodontal health and disease and their changes after nonsurgical periodontal therapy
Source: ISME J. 2018 Jan 16;12(5):1210–24. doi: 10.1038/s41396-017-0037-1 (PMC5932080; doi:10.1038/s41396-017-0037-1)
Supplement: Supplementary file 7 — Supplementary Table S6 [file 41396_2017_37_MOESM7_ESM.docx]

Supplementary Table S6. Dominant taxa identified by cluster analysis

| \|  \|  \| **Genus** \| **Health- or disease-associated** \| \| --- \| --- \| --- \| --- \| \| **Box 1** \|  \|  \|  \| \|  \| OTU_1051 \| *Oribacterium* \|  \| \|  \| OTU_1095 \| *Mogibacterium* \|  \| \|  \| OTU_113 \| Unclassified (*Leptotrichiaceae*) \|  \| \|  \| OTU_117 \| *Actinomyces* \|  \| \|  \| OTU_118 \| Unclassified (*Lachnospiraceae*) \|  \| \|  \| OTU_122 \| SR1_genera \|  \| \|  \| OTU_123 \| *Moryella* \|  \| \|  \| OTU_1318 \| *Prevotella* \|  \| \|  \| OTU_132 \| *Oribacterium* \|  \| \|  \| OTU_1359 \| Unclassified (*Pasteurellaceae*) \|  \| \|  \| OTU_136 \| *Prevotella* \|  \| \|  \| OTU_143 \| *Campylobacter* \|  \| \|  \| OTU_1441 \| *Neisseria* \|  \| \|  \| OTU_153 \| SR1_genera \|  \| \|  \| OTU_258 \| *Centipeda* \|  \| \|  \| OTU_43 \| Unclassified (*Prevotellaceae*) \|  \| \|  \| OTU_44 \| *Megasphaera* \|  \| \|  \| OTU_47 \| *Oribacterium* \|  \| \|  \| OTU_484 \| *Actinomyces* \|  \| \|  \| OTU_52 \| *Prevotella* \|  \| \|  \| OTU_54 \| *Peptostreptococcus* \|  \| \|  \| OTU_57 \| *Prevotella* \|  \| \|  \| OTU_60 \| *Paraprevotella* \|  \| \|  \| OTU_647 \| *Acetanaerobacterium* \|  \| \|  \| OTU_76 \| *Solobacterium* \|  \| \|  \| OTU_774 \| *Veillonella* \|  \| \|  \| OTU_81 \| *Eubacterium* \|  \| \|  \| OTU_84 \| Unclassified (*Clostridiales*) \|  \| \|  \| OTU_86 \| *Atopobium* \|  \| \|  \| OTU_96 \| *Syntrophococcus* \|  \| \| **Box 2** \|  \|  \|  \| \|  \| OTU_107 \| *Schwartzia* \|  \| \|  \| OTU_109 \| Unclassified (*Firmicutes*) \| disease-associated taxon \| \|  \| OTU_110 \| *Johnsonella* \| disease-associated taxon \| \|  \| OTU_111 \| Unclassified (*Bacteroidetes*) \| disease-associated taxon \| \|  \| OTU_112 \| *Johnsonella* \|  \| \|  \| OTU_125 \| *Prevotella* \|  \| \|  \| OTU_1265 \| *Prevotella* \|  \| \|  \| OTU_127 \| *Leptotrichia* \|  \| \|  \| OTU_129 \| *Treponema* \| disease-associated taxon \| \|  \| OTU_130 \| *Prevotella* \|  \| \|  \| OTU_131 \| *Prevotella* \| disease-associated taxon \| \|  \| OTU_135 \| *Treponema* \|  \| \|  \| OTU_137 \| *Hallella* \| disease-associated taxon \| \|  \| OTU_140 \| *Syntrophococcus* \|  \| \|  \| OTU_142 \| Unclassified (*Clostridiales*) \|  \| \|  \| OTU_144 \| Unclassified (*Lachnospiraceae*) \|  \| \|  \| OTU_147 \| *Treponema* \| disease-associated taxon \| \|  \| OTU_149 \| Unclassified (*Bacteroidales*) \|  \| \|  \| OTU_152 \| Unclassified (*Peptostreptococcaceae*) \|  \| \|  \| OTU_156 \| Unclassified (*Clostridiales*) \|  \| \|  \| OTU_1661 \| *Treponema* \|  \| \|  \| OTU_170 \| *Tannerella* \|  \| \|  \| OTU_276 \| *Treponema* \|  \| \|  \| OTU_39 \| *Streptobacillus* \|  \| \|  \| OTU_392 \| *Treponema* \|  \| \|  \| OTU_536 \| *Treponema* \|  \| \|  \| OTU_615 \| *Treponema* \| disease-associated taxon \| \|  \| OTU_62 \| *Mycoplasma* \| disease-associated taxon \| \|  \| OTU_63 \| *Phocaeicola* \| disease-associated taxon \| \|  \| OTU_685 \| *Prevotella* \|  \| \|  \| OTU_71 \| *Leptotrichia* \|  \| \|  \| OTU_72 \| *Leptotrichia* \| disease-associated taxon \| \|  \| OTU_726 \| *Prevotella* \|  \| \|  \| OTU_74 \| *Eubacterium* \| disease-associated taxon \| \|  \| OTU_75 \| *Eubacterium* \| disease-associated taxon \| \|  \| OTU_85 \| *Prevotella* \|  \| \|  \| OTU_90 \| Unclassified (*Clostridiales*) \|  \| \|  \| OTU_95 \| *Treponema* \|  \| \|  \| OTU_97 \| *Mogibacterium* \| disease-associated taxon \| \| **Box 3** \|  \|  \|  \| \|  \| OTU_102 \| Unclassified (*Pasteurellaceae*) \|  \| \|  \| OTU_103 \| unclassified (*Peptostreptococcaceae*) \|  \| \|  \| OTU_108 \| *Leptotrichia* \|  \| \|  \| OTU_1170 \| *Leptotrichia* \|  \| \|  \| OTU_1207 \| *Capnocytophaga* \|  \| \|  \| OTU_1274 \| *Leptotrichia* \| health-associated taxon \| \|  \| OTU_128 \| *Actinomyces* \|  \| \|  \| OTU_1324 \| *Rothia* \|  \| \|  \| OTU_1325 \| *Tannerella* \|  \| \|  \| OTU_134 \| *Capnocytophaga* \|  \| \|  \| OTU_1354 \| *Capnocytophaga* \|  \| \|  \| OTU_1456 \| *Porphyromonas* \|  \| \|  \| OTU_151 \| *Prevotella* \|  \| \|  \| OTU_1608 \| *Fusobacterium* \|  \| \|  \| OTU_1652 \| *Tannerella* \|  \| \|  \| OTU_175 \| Unclassified (*Flavobacteriaceae*) \|  \| \|  \| OTU_1808 \| *Haemophilus* \|  \| \|  \| OTU_309 \| *Leptotrichia* \|  \| \|  \| OTU_37 \| Unclassified (*Burkholderiales*) \|  \| \|  \| OTU_385 \| *Cardiobacterium* \|  \| \|  \| OTU_40 \| *Corynebacterium* \|  \| \|  \| OTU_41 \| Unclassified (*Actinomycetaceae*) \|  \| \|  \| OTU_438 \| *Capnocytophaga* \|  \| \|  \| OTU_48 \| *Kingella* \|  \| \|  \| OTU_50 \| *Oribacterium* \|  \| \|  \| OTU_51 \| *Leptotrichia* \|  \| \|  \| OTU_550 \| *Capnocytophaga* \| health-associated taxon \| \|  \| OTU_56 \| *Neisseria* \|  \| \|  \| OTU_586 \| *Granulicatella* \|  \| \|  \| OTU_59 \| *Syntrophococcus* \|  \| \|  \| OTU_64 \| Unclassified (*Actinomycetales*) \|  \| \|  \| OTU_669 \| Unclassified (*Neisseriaceae*) \|  \| \|  \| OTU_69 \| *Peptococcus* \|  \| \|  \| OTU_760 \| *Leptotrichia* \|  \| \|  \| OTU_79 \| *Porphyromonas* \|  \| \|  \| OTU_80 \| *Cardiobacterium* \|  \| \|  \| OTU_808 \| *Leptotrichia* \|  \| \|  \| OTU_88 \| *Actinomyces* \|  \| \|  \| OTU_91 \| *Prevotella* \| health-associated taxon \| \|  \| OTU_93 \| *Planobacterium* \|  \| \| **Box 4** \|  \|  \|  \| \|  \| OTU_101 \| *Treponema* \| disease-associated taxon \| \|  \| OTU_11 \| *Prevotella* \|  \| \|  \| OTU_12 \| *Tannerella* \| disease-associated taxon \| \|  \| OTU_13 \| *Filifactor* \| disease-associated taxon \| \|  \| OTU_16 \| *Dialister* \|  \| \|  \| OTU_1773 \| *Treponema* \| disease-associated taxon \| \|  \| OTU_19 \| *Paludibacter* \|  \| \|  \| OTU_24 \| *Selenomonas* \|  \| \|  \| OTU_25 \| Unclassified (*Firmicutes*) \| disease-associated taxon \| \|  \| OTU_28 \| *Prevotella* \|  \| \|  \| OTU_29 \| *Eubacterium* \|  \| \|  \| OTU_30 \| *Treponema* \|  \| \|  \| OTU_31 \| *Parvimonas* \|  \| \|  \| OTU_33 \| *Treponema* \| disease-associated taxon \| \|  \| OTU_35 \| *Prevotella* \|  \| \|  \| OTU_36 \| Unclassified (*Bacteroidetes*) \|  \| \|  \| OTU_4 \| *Porphyromonas* \| disease-associated taxon \| \|  \| OTU_45 \| Unclassified (*Firmicutes*) \|  \| \|  \| OTU_46 \| *Selenomonas* \|  \| \|  \| OTU_49 \| *Treponema* \|  \| \|  \| OTU_55 \| *Prevotella* \| disease-associated taxon \| \|  \| OTU_561 \| Unclassified (*Firmicutes*) \|  \| \|  \| OTU_58 \| Unclassified (*Firmicutes*) \| disease-associated taxon \| \|  \| OTU_606 \| *Treponema* \| disease-associated taxon \| \|  \| OTU_66 \| Unclassified (*Firmicutes*) \| disease-associated taxon \| \|  \| OTU_73 \| *Desulfobulbus* \| disease-associated taxon \| \|  \| OTU_8 \| *Porphyromonas* \| disease-associated taxon \| \|  \| OTU_9 \| *Prevotella* \|  \| \|  \| OTU_92 \| *Prevotella* \|  \| \|  \| OTU_992 \| *Treponema* \|  \| \| **Box 5** \|  \|  \|  \| \|  \| OTU_10 \| *Prevotella* \|  \| \|  \| OTU_1327 \| *Veillonella* \| health-associated taxon \| \|  \| OTU_18 \| *Gemella* \|  \| \|  \| OTU_2 \| *Neisseria* \|  \| \|  \| OTU_20 \| *Streptococcus* \|  \| \|  \| OTU_27 \| *Rothia* \|  \| \|  \| OTU_3 \| *Streptococcus* \|  \| \|  \| OTU_32 \| *Granulicatella* \|  \| \|  \| OTU_361 \| *Actinomyces* \|  \| \|  \| OTU_38 \| *Campylobacter* \|  \| \|  \| OTU_437 \| *Prevotella* \|  \| \|  \| OTU_6 \| *Actinobacillus* \|  \| \|  \| OTU_7 \| *Veillonella* \| health-associated taxon \| \|  \| OTU_860 \| *Streptococcus* \|  \| \|  \|  \|  \|  \| \|  \|  \|  \|  \| \|  \|  \|  \|  \| \|  \|  \|  \|  \| \|  \|  \|  \|  \| \|  \|  \|  \|  \| \|  \|  \|  \|  \| \|  \|  \|  \|  \| \|  \|  \|  \|  \| \|  \|  \|  \|  \| \|  \|  \|  \|  \| \|  \|  \|  \|  \| \|  \|  \|  \|  \| \|  \|  \|  \|  \| |  |  |
| --- | --- | --- | --- | --- | --- | --- | --- | --- | --- | --- | --- | --- | --- | --- | --- | --- | --- | --- | --- | --- | --- | --- | --- | --- | --- | --- | --- | --- | --- | --- | --- | --- | --- | --- | --- | --- | --- | --- | --- | --- | --- | --- | --- | --- | --- | --- | --- | --- | --- | --- | --- | --- | --- | --- | --- | --- | --- | --- | --- | --- | --- | --- | --- | --- | --- | --- | --- | --- | --- | --- | --- | --- | --- | --- | --- | --- | --- | --- | --- | --- | --- | --- | --- | --- | --- | --- | --- | --- | --- | --- | --- | --- | --- | --- | --- | --- | --- | --- | --- | --- | --- | --- | --- | --- | --- | --- | --- | --- | --- | --- | --- | --- | --- | --- | --- | --- | --- | --- | --- | --- | --- | --- | --- | --- | --- | --- | --- | --- | --- | --- | --- | --- | --- | --- | --- | --- | --- | --- | --- | --- | --- | --- | --- | --- | --- | --- | --- | --- | --- | --- | --- | --- | --- | --- | --- | --- | --- | --- | --- | --- | --- | --- | --- | --- | --- | --- | --- | --- | --- | --- | --- | --- | --- | --- | --- | --- | --- | --- | --- | --- | --- | --- | --- | --- | --- | --- | --- | --- | --- | --- | --- | --- | --- | --- | --- | --- | --- | --- | --- | --- | --- | --- | --- | --- | --- | --- | --- | --- | --- | --- | --- | --- | --- | --- | --- | --- | --- | --- | --- | --- | --- | --- | --- | --- | --- | --- | --- | --- | --- | --- | --- | --- | --- | --- | --- | --- | --- | --- | --- | --- | --- | --- | --- | --- | --- | --- | --- | --- | --- | --- | --- | --- | --- | --- | --- | --- | --- | --- | --- | --- | --- | --- | --- | --- | --- | --- | --- | --- | --- | --- | --- | --- | --- | --- | --- | --- | --- | --- | --- | --- | --- | --- | --- | --- | --- | --- | --- | --- | --- | --- | --- | --- | --- | --- | --- | --- | --- | --- | --- | --- | --- | --- | --- | --- | --- | --- | --- | --- | --- | --- | --- | --- | --- | --- | --- | --- | --- | --- | --- | --- | --- | --- | --- | --- | --- | --- | --- | --- | --- | --- | --- | --- | --- | --- | --- | --- | --- | --- | --- | --- | --- | --- | --- | --- | --- | --- | --- | --- | --- | --- | --- | --- | --- | --- | --- | --- | --- | --- | --- | --- | --- | --- | --- | --- | --- | --- | --- | --- | --- | --- | --- | --- | --- | --- | --- | --- | --- | --- | --- | --- | --- | --- | --- | --- | --- | --- | --- | --- | --- | --- | --- | --- | --- | --- | --- | --- | --- | --- | --- | --- | --- | --- | --- | --- | --- | --- | --- | --- | --- | --- | --- | --- | --- | --- | --- | --- | --- | --- | --- | --- | --- | --- | --- | --- | --- | --- | --- | --- | --- | --- | --- | --- | --- | --- | --- | --- | --- | --- | --- | --- | --- | --- | --- | --- | --- | --- | --- | --- | --- | --- | --- | --- | --- | --- | --- | --- | --- | --- | --- | --- | --- | --- | --- | --- | --- | --- | --- | --- | --- | --- | --- | --- | --- | --- | --- | --- | --- | --- | --- | --- | --- | --- | --- | --- | --- | --- | --- | --- | --- | --- | --- | --- | --- | --- | --- | --- | --- | --- | --- | --- | --- | --- | --- | --- | --- | --- | --- | --- | --- | --- | --- | --- | --- | --- | --- | --- | --- | --- | --- | --- | --- | --- | --- | --- | --- | --- | --- | --- | --- | --- | --- | --- | --- | --- | --- | --- | --- | --- | --- | --- | --- | --- | --- | --- | --- | --- | --- | --- | --- | --- | --- | --- | --- | --- | --- | --- | --- | --- | --- | --- | --- | --- | --- | --- | --- | --- | --- | --- | --- | --- | --- | --- | --- | --- | --- | --- | --- | --- | --- | --- | --- | --- | --- | --- | --- | --- | --- | --- | --- | --- | --- | --- | --- | --- | --- | --- | --- | --- | --- | --- | --- | --- | --- | --- | --- | --- | --- | --- | --- | --- | --- | --- | --- | --- | --- | --- | --- | --- | --- | --- | --- | --- | --- | --- | --- | --- | --- | --- | --- | --- | --- | --- | --- | --- | --- | --- | --- | --- | --- | --- | --- | --- | --- | --- | --- | --- | --- | --- | --- | --- | --- | --- | --- | --- | --- | --- | --- | --- | --- | --- | --- | --- | --- | --- | --- | --- | --- | --- | --- | --- | --- | --- | --- | --- | --- | --- | --- | --- | --- | --- | --- | --- | --- | --- | --- | --- | --- | --- | --- | --- | --- | --- | --- | --- |
