# Supplementary material for: Oral microbiota of periodontal health and disease and their changes after nonsurgical periodontal therapy
Source: ISME J. 2018 Jan 16;12(5):1210–24. doi: 10.1038/s41396-017-0037-1 (PMC5932080; doi:10.1038/s41396-017-0037-1)
Supplement: Supplementary file 8 — Supplementary Table S7 [file 41396_2017_37_MOESM8_ESM.docx]

Supplementary Table S7. Parameters of the networks of the 6 sample groups

| Sample^a^ | No. of nodes | No. of links | Clustering coefficient | Average shortest path length | Diameter | Centralization | Average number of neighbors | Density | Heterogeneity |
| --- | --- | --- | --- | --- | --- | --- | --- | --- | --- |
| HP | 174 | 603 | 0.505 | 4.785 | 11 | 0.082 | 6.931 | 0.040 | 0.696 |
| HS | 136 | 506 | 0.501 | 4.045 | 11 | 0.079 | 7.441 | 0.055 | 0.576 |
| D1P | 179 | 568 | 0.426 | 5.711 | 14 | 0.083 | 6.346 | 0.036 | 0.691 |
| D2P | 160 | 453 | 0.485 | 4.938 | 14 | 0.047 | 5.663 | 0.036 | 0.593 |
| D1S | 197 | 681 | 0.442 | 3.825 | 9 | 0.083 | 6.914 | 0.035 | 0.758 |
| D2S | 161 | 661 | 0.561 | 4.484 | 15 | 0.125 | 8.211 | 0.051 | 0.811 |

^a^Subgingival plaque samples: D1P, diseased/pre-treatment plaque; HP, healthy plaque; D2P, diseased/post-treatment plaque. Saliva samples: D1S, diseased/pre-treatment saliva; HS, healthy saliva; D2S, diseased/post-treatment saliva.
