# Supplementary material for: Oral microbiota of periodontal health and disease and their changes after nonsurgical periodontal therapy
Source: ISME J. 2018 Jan 16;12(5):1210–24. doi: 10.1038/s41396-017-0037-1 (PMC5932080; doi:10.1038/s41396-017-0037-1)
Supplement: Supplementary file 9 — Supplementary Table S8 [file 41396_2017_37_MOESM9_ESM.docx]

Supplementary Table S8. Identification of connectors and module hubs in the sample groups

| **OTU** | **Sample^a^ Group** | **Taxon** | **Among-module connectivity (Pi)** | **Within-module connectivity (Zi)** | **Role** |
| --- | --- | --- | --- | --- | --- |
| OTU_7 | D1P | *Veillonella* | 0.71875 | -0.683506743 | connector |
| OTU_837 | D1P | *Capnocytophaga* | 0.64 | -1.304876509 | connector |
| OTU_1354 | D2P | *Capnocytophaga* | 0.691358025 | -0.388107736 | connector |
| OTU_572 | HP | *Atopobium* | 0.666666667 | -1.714762432 | connector |
| OTU_222 | HP | *Propionivibrio* | 0.666666667 | -1.538967528 | connector |
| OTU_6 | HP | *Terrahaemophilus* | 0 | 3.19958775 | module hub |
| OTU_171 | D1S | *Stomatobaculum* | 0.6484375 | -0.059976014 | connector |
| OTU_95 | D1S | *Treponema* | 0.625 | 0 | connector |
| OTU_141 | D1S | *Anaerolineae* | 0.625 | -0.444444444 | connector |
| OTU_52 | D1S | *Prevotella* | 0 | 2.898206753 | module hub |
| OTU_13 | D2S | *Filifactor* | 0.72 | -0.267261242 | connector |
| OTU_58 | D2S | *Peptostreptococcaceae* | 0.625 | -0.848477933 | connector |
| OTU_1652 | D2S | *Tannerella* | 0.666666667 | -0.922705473 | connector |
| OTU_103 | D2S | *Eubacterium* | 0.775510204 | -1.020641491 | connector |
| OTU_42 | D2S | *Capnocytophaga* | 0.666666667 | -0.733586072 | connector |
| OTU_24 | HS | *Selenomonas* | 0.625 | -2.258735662 | connector |
| OTU_617 | HS | *Veillonella* | 0.64 | -1.048078123 | connector |
| OTU_143 | HS | *Campylobacter* | 0 | 2.558543653 | module hub |

^a^Subgingival plaque samples: D1P, diseased/pre-treatment plaque; D2P, diseased/post-treatment plaque; HP, healthy plaque. Saliva samples: D1S, diseased/pre-treatment saliva; D2S, diseased/post-treatment saliva; HS, healthy saliva.
