# Supplementary figures and images for: Oral microbiota of periodontal health and disease and their changes after nonsurgical periodontal therapy
Source: ISME J. 2018 Jan 16;12(5):1210–24. doi: 10.1038/s41396-017-0037-1 (PMC5932080; doi:10.1038/s41396-017-0037-1)

## Slide 1
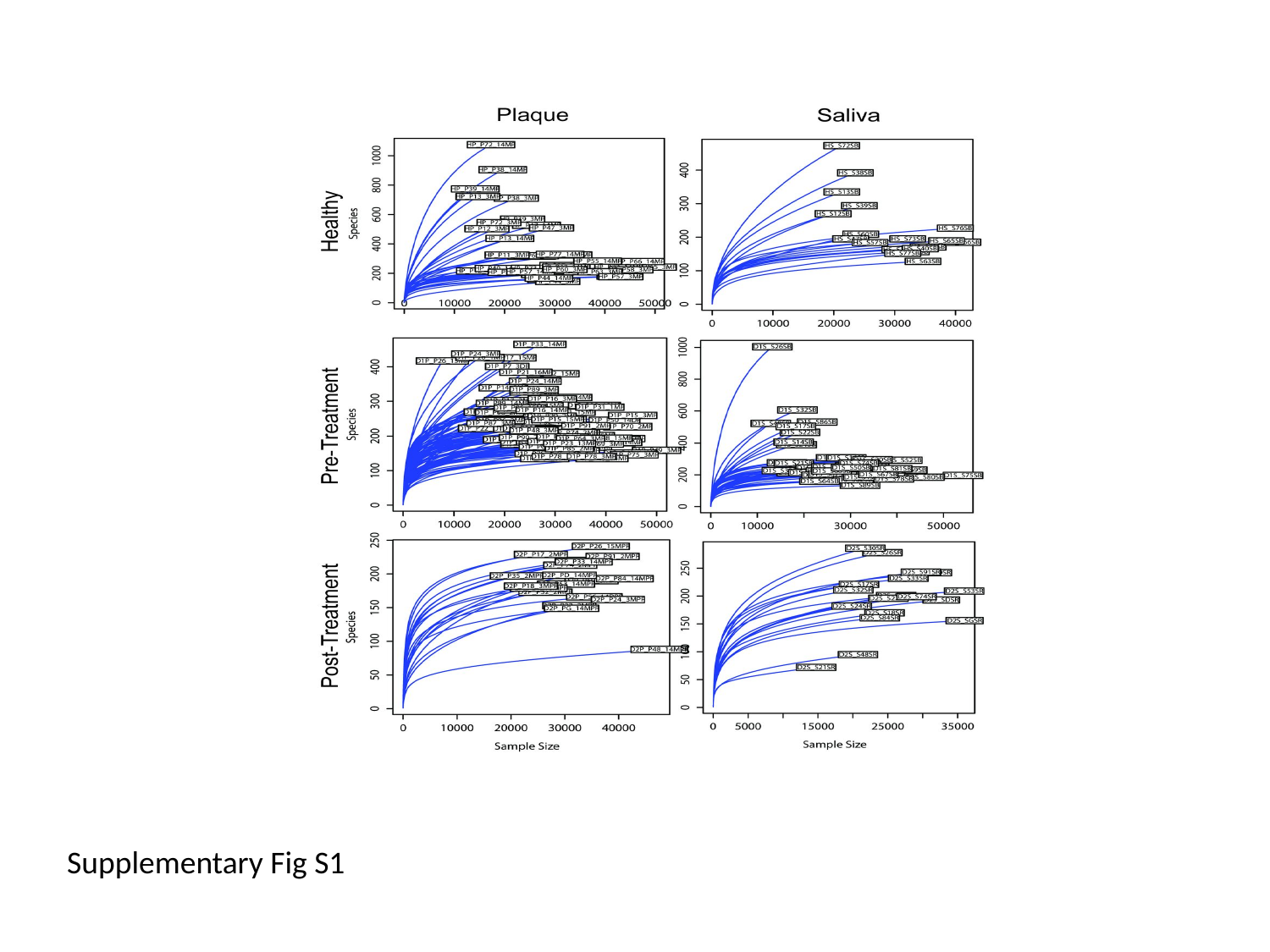

Supplementary Fig S1

Supplement: Supplementary file 10 — Supplementary Figure S1 [file 41396_2017_37_MOESM10_ESM.pptx]

## Slide 1
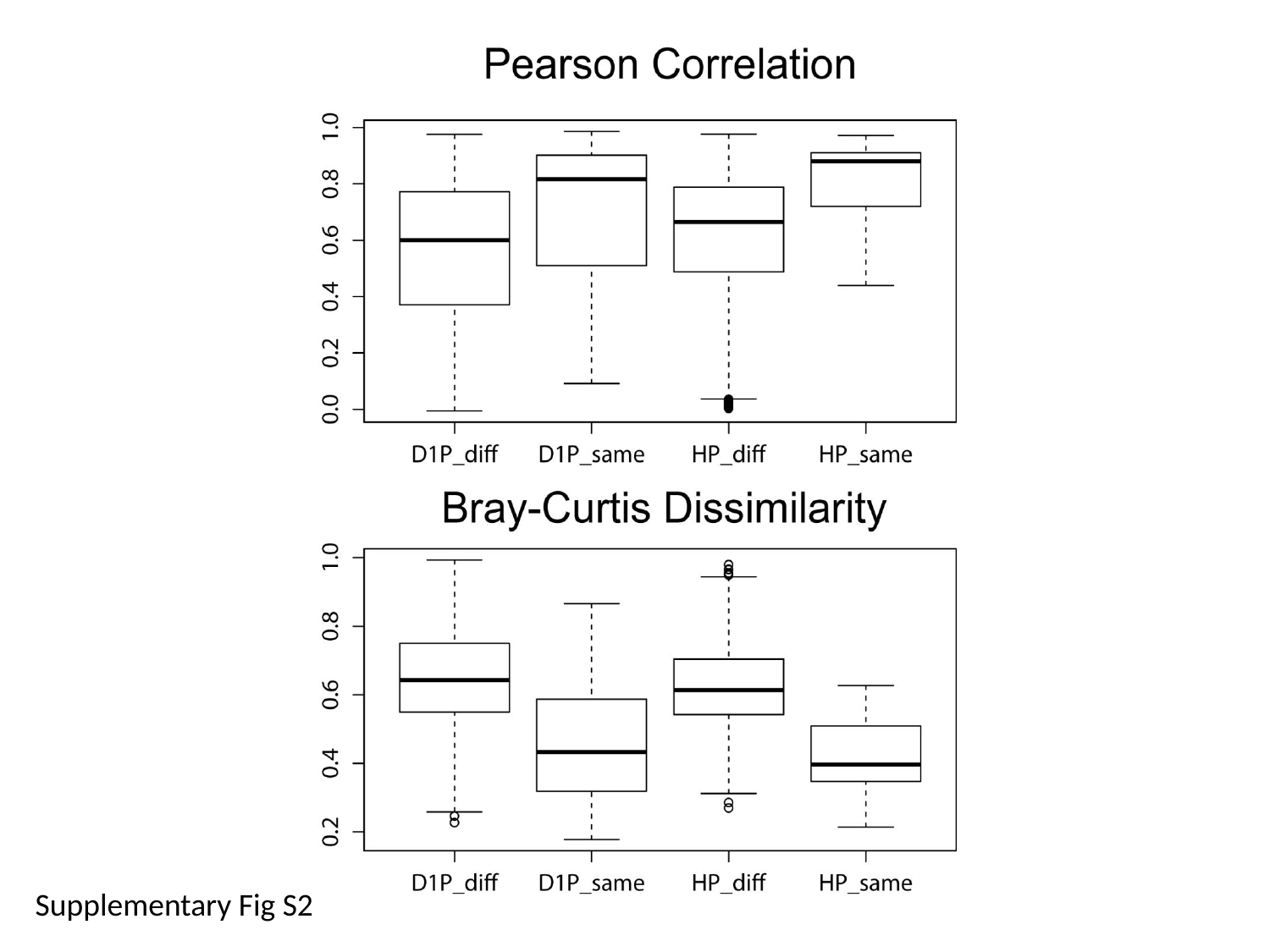

Supplementary Fig S2

Supplement: Supplementary file 11 — Supplementary Figure S2 [file 41396_2017_37_MOESM11_ESM.pptx]

## Slide 1
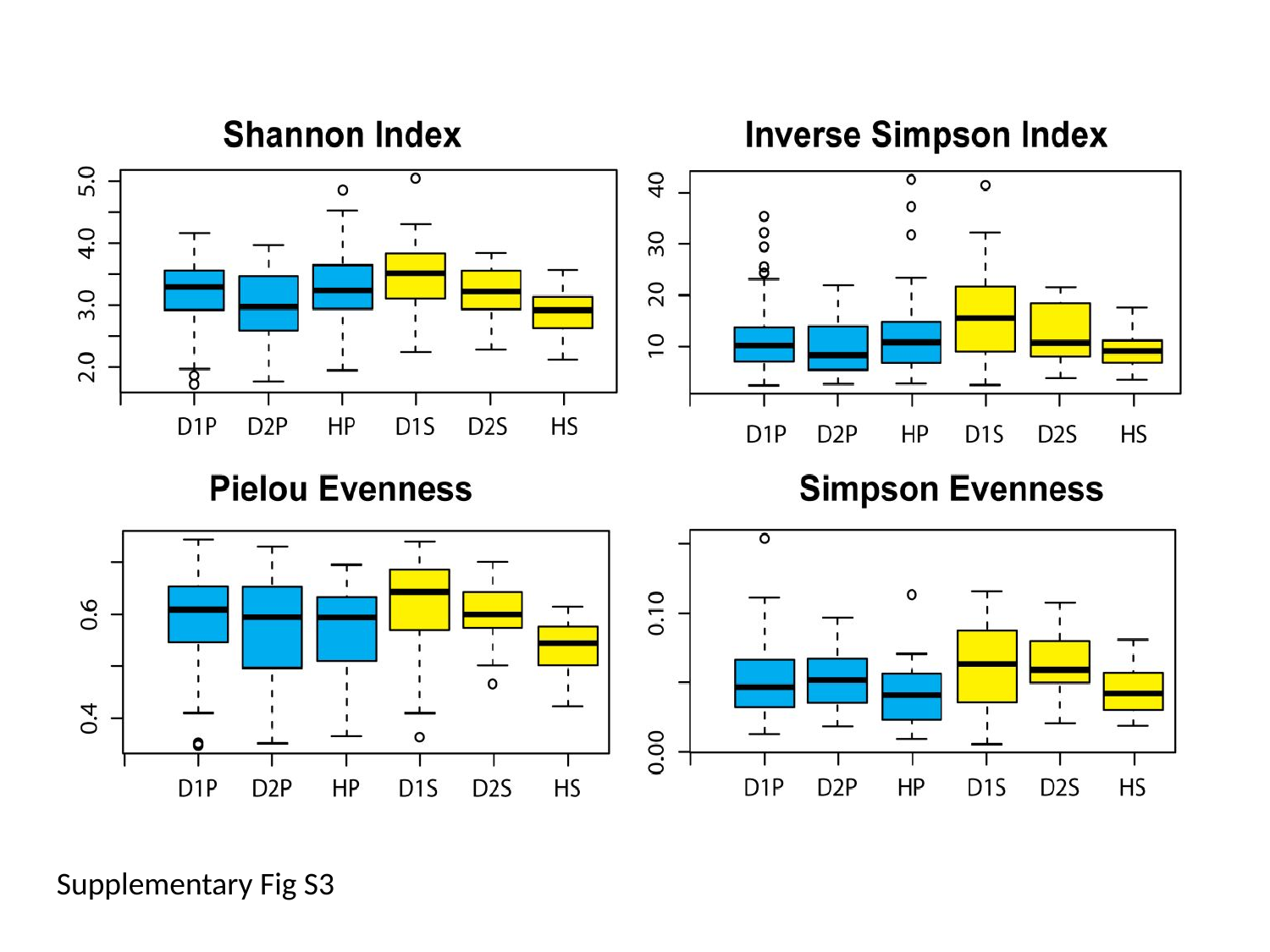

Supplementary Fig S3

Supplement: Supplementary file 12 — Supplementary Figure S3 [file 41396_2017_37_MOESM12_ESM.pptx]

## Slide 1
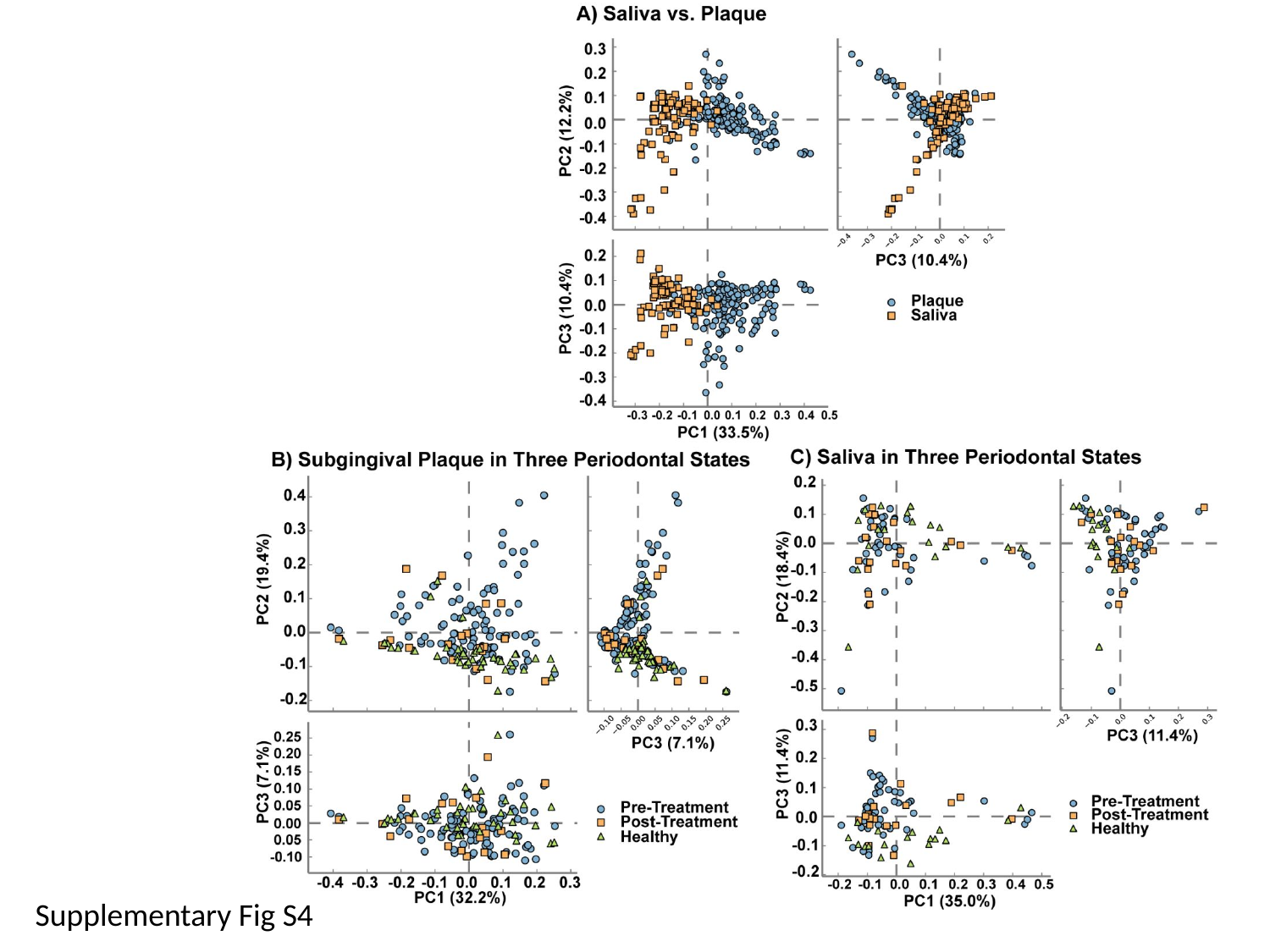

Supplementary Fig S4

Supplement: Supplementary file 13 — Supplementary Figure S4 [file 41396_2017_37_MOESM13_ESM.pptx]

## Slide 1
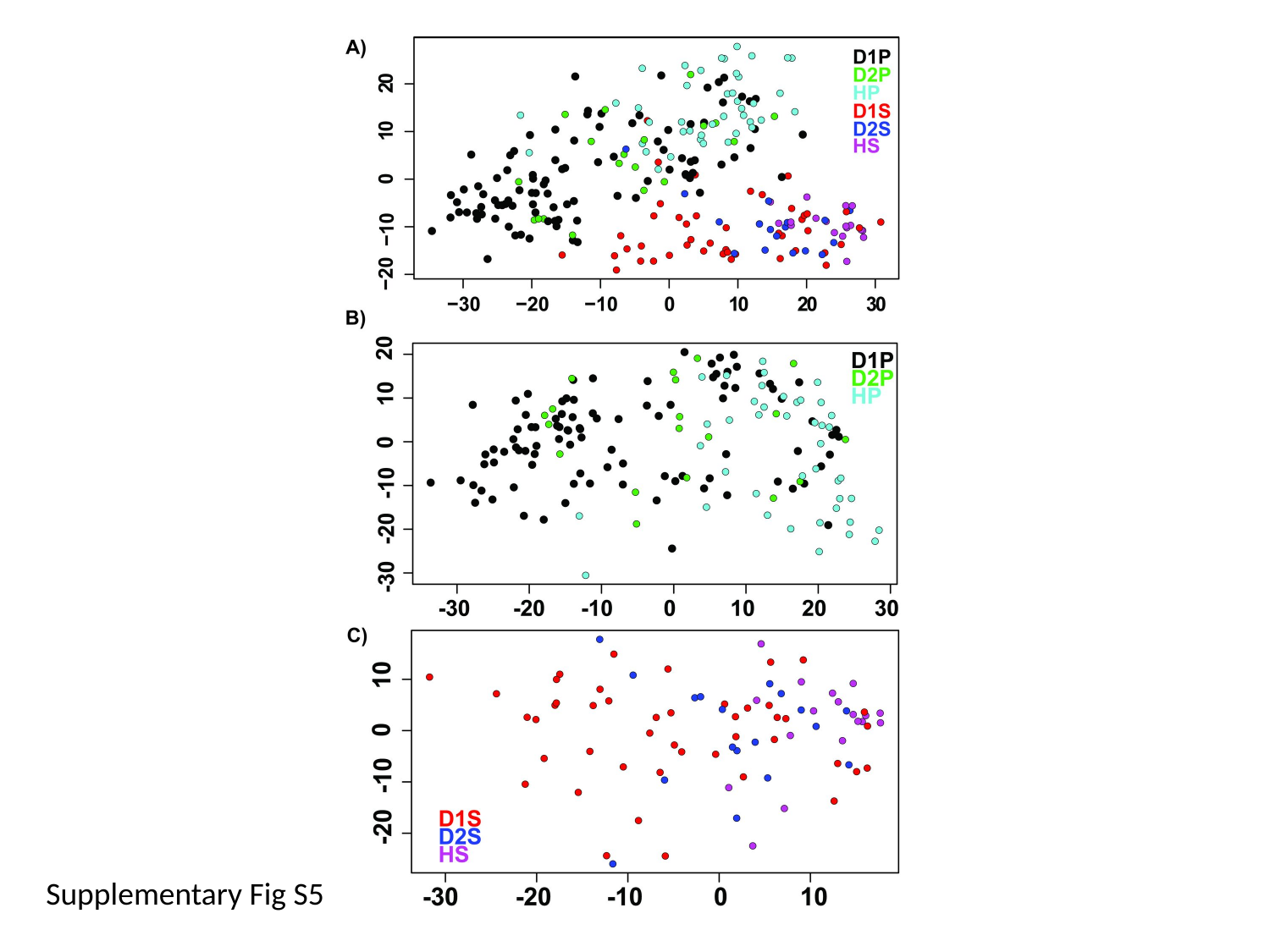

Supplementary Fig S5

Supplement: Supplementary file 14 — Supplementary Figure S5 [file 41396_2017_37_MOESM14_ESM.pptx]

## Slide 1
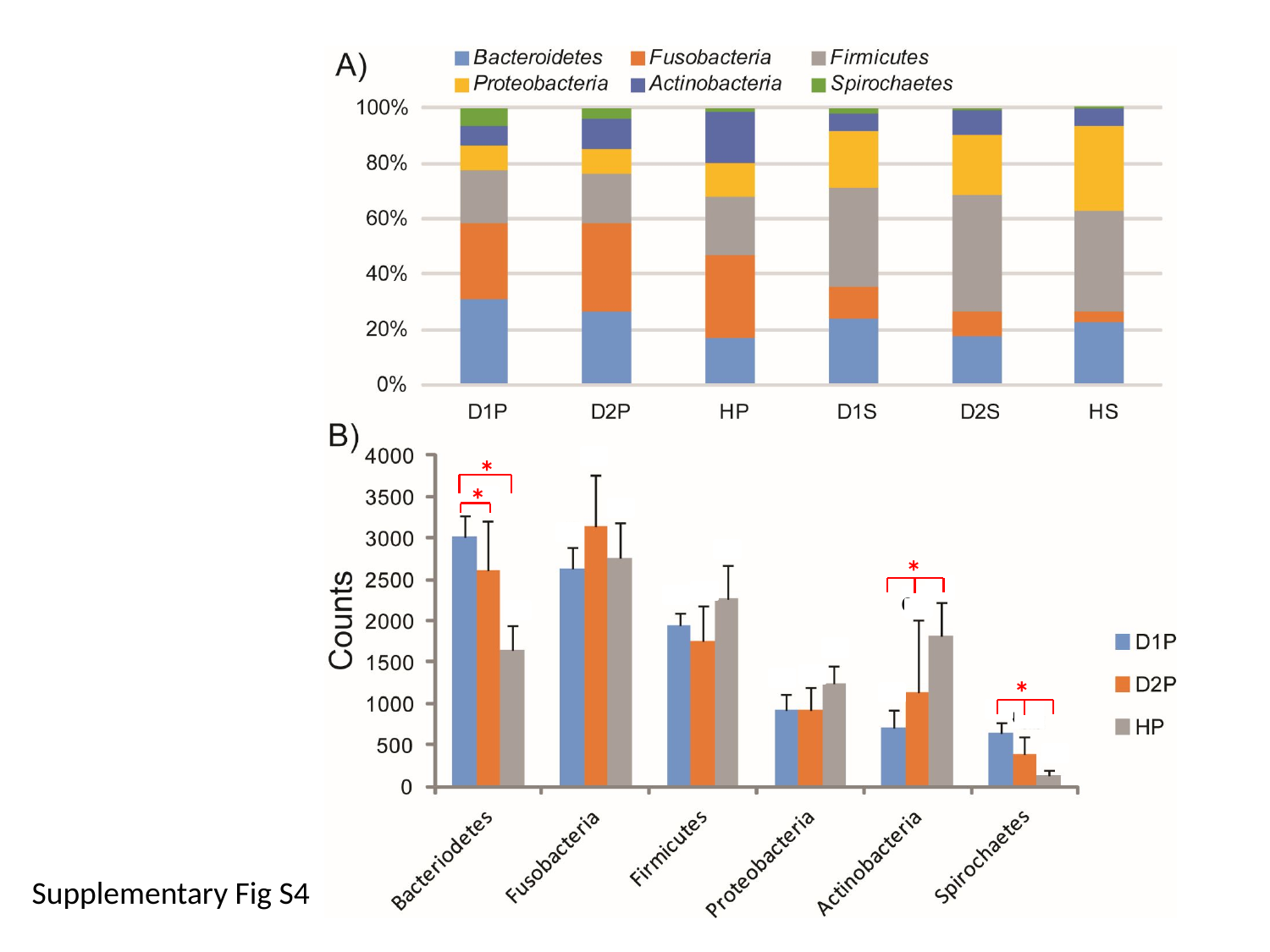

*
*
*
c
*
c
Supplementary Fig S4

Supplement: Supplementary file 15 — Supplementary Figure S6 [file 41396_2017_37_MOESM15_ESM.pptx]
