# Supplementary material for: Oral microbiota of periodontal health and disease and their changes after nonsurgical periodontal therapy
Source: ISME J. 2018 Jan 16;12(5):1210–24. doi: 10.1038/s41396-017-0037-1 (PMC5932080; doi:10.1038/s41396-017-0037-1)
Supplement: Supplementary file 16 — Supplementary Figure S7 [file 41396_2017_37_MOESM16_ESM.pptx]

## Slide 1
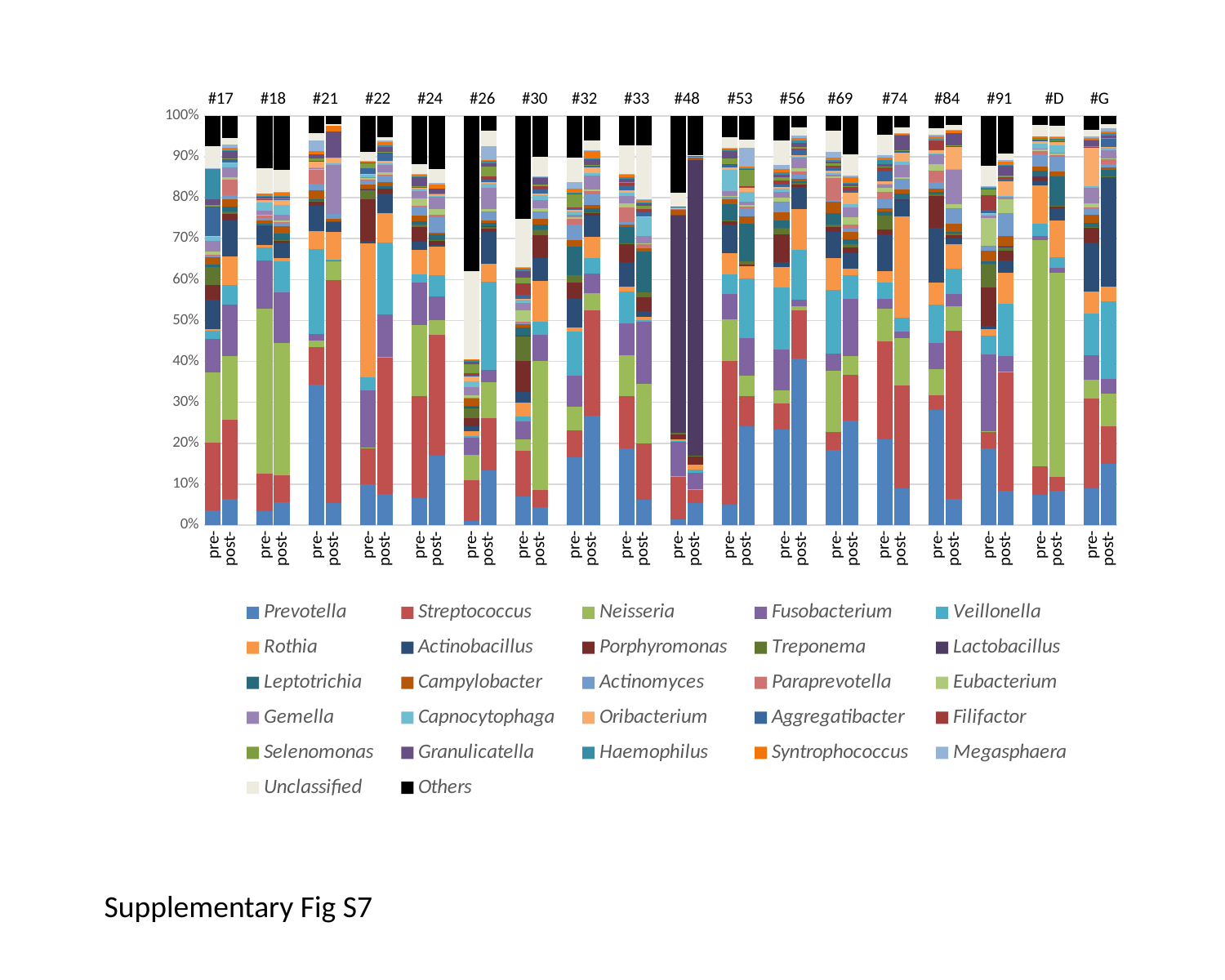

#17
#18
#21
#22
#24
#26
#30
#32
#33
#48
#53
#56
#69
#74
#84
#91
#D
#G
### Chart
| Category | Prevotella | Streptococcus | Neisseria | Fusobacterium | Veillonella | Rothia | Actinobacillus | Porphyromonas | Treponema | Lactobacillus | Leptotrichia | Campylobacter | Actinomyces | Paraprevotella | Eubacterium | Gemella | Capnocytophaga | Oribacterium | Aggregatibacter | Filifactor | Selenomonas | Granulicatella | Haemophilus | Syntrophococcus | Megasphaera | Unclassified | Others |
|---|---|---|---|---|---|---|---|---|---|---|---|---|---|---|---|---|---|---|---|---|---|---|---|---|---|---|---|pre-
post-
pre-
post-
pre-
post-
pre-
post-
pre-
post-
pre-
post-
pre-
post-
pre-
post-
pre-
post-
pre-
post-
pre-
post-
pre-
post-
pre-
post-
pre-
post-
pre-
post-
pre-
post-
pre-
post-
pre-
post-
Supplementary Fig S7
