# Supplementary material for: Oral microbiota of periodontal health and disease and their changes after nonsurgical periodontal therapy
Source: ISME J. 2018 Jan 16;12(5):1210–24. doi: 10.1038/s41396-017-0037-1 (PMC5932080; doi:10.1038/s41396-017-0037-1)
Supplement: Supplementary file 17 — Supplementary Figure S8 [file 41396_2017_37_MOESM17_ESM.pptx]

## Slide 1
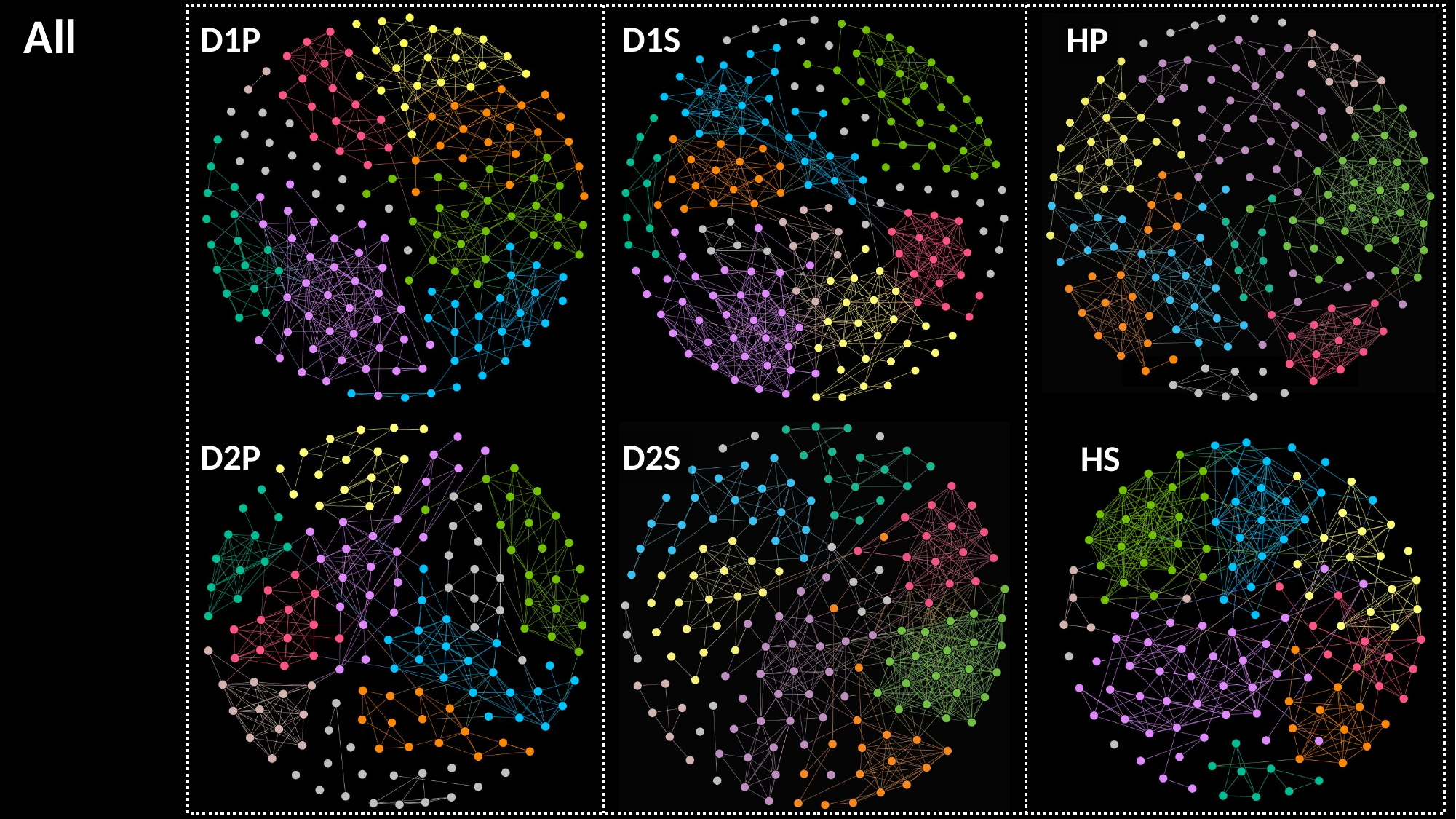

All
D1S
D1P
HP
D2P
D2S
HS

## Slide 2
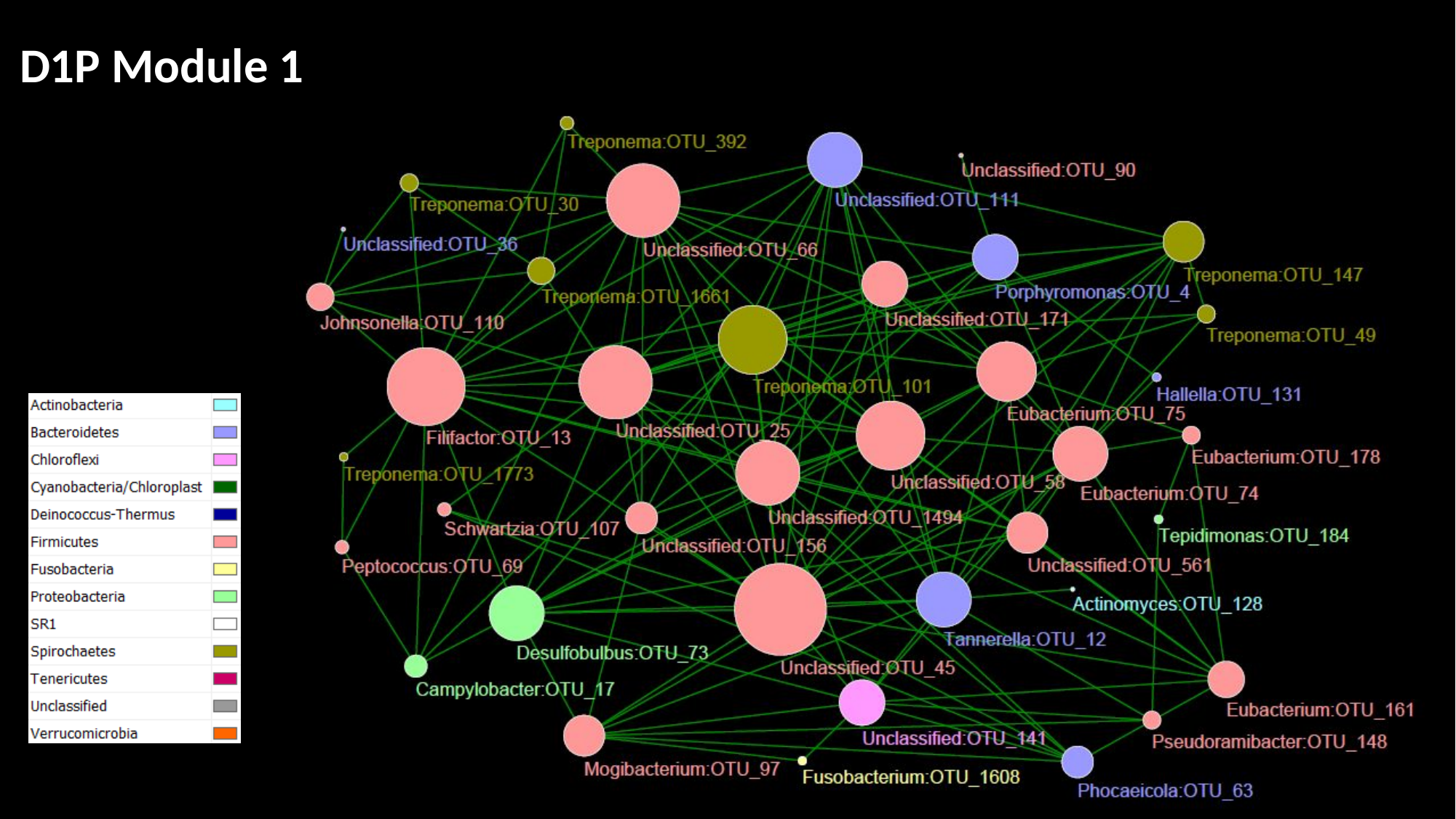

D1P Module 1

## Slide 3
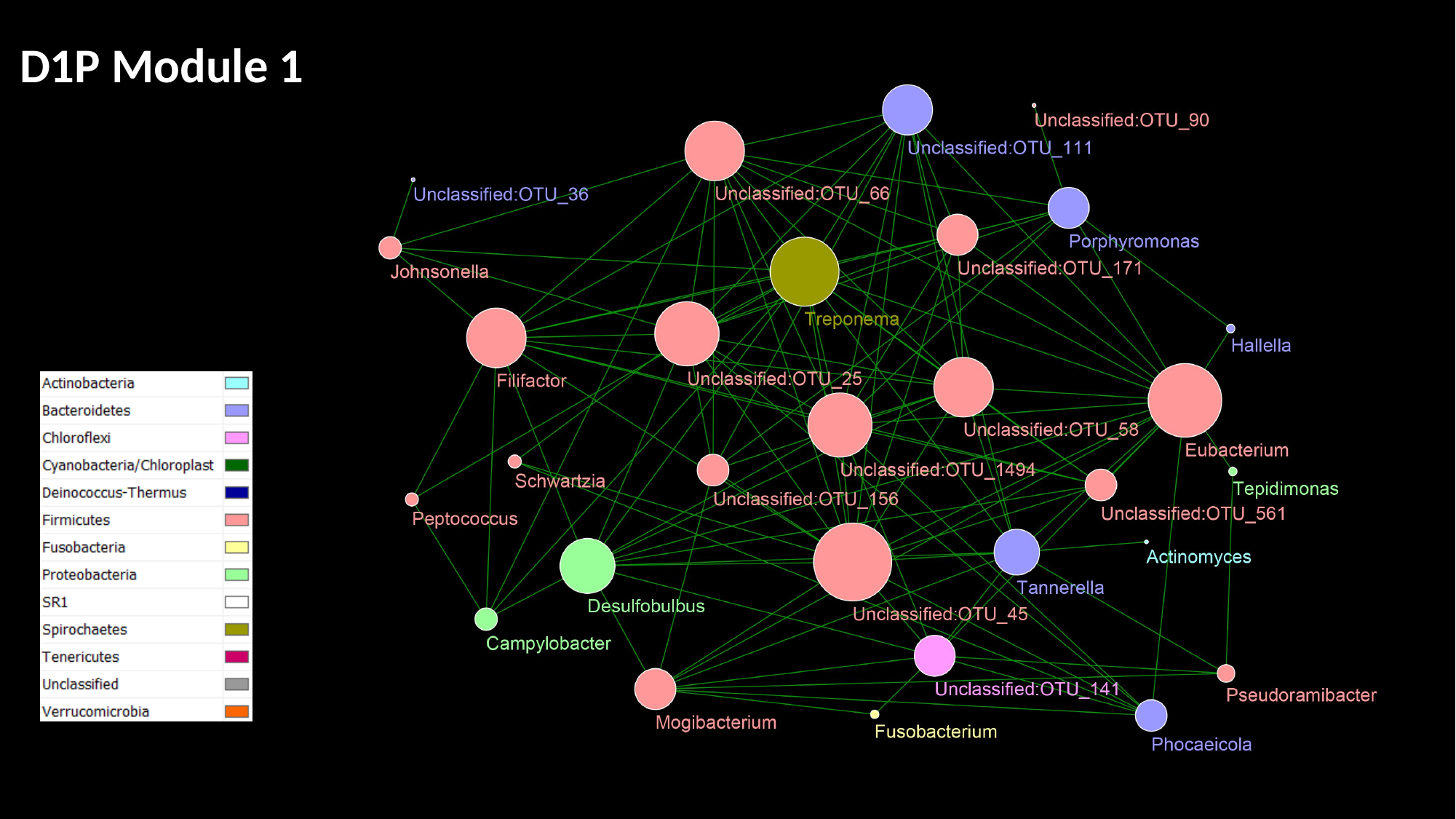

D1P Module 1

## Slide 4
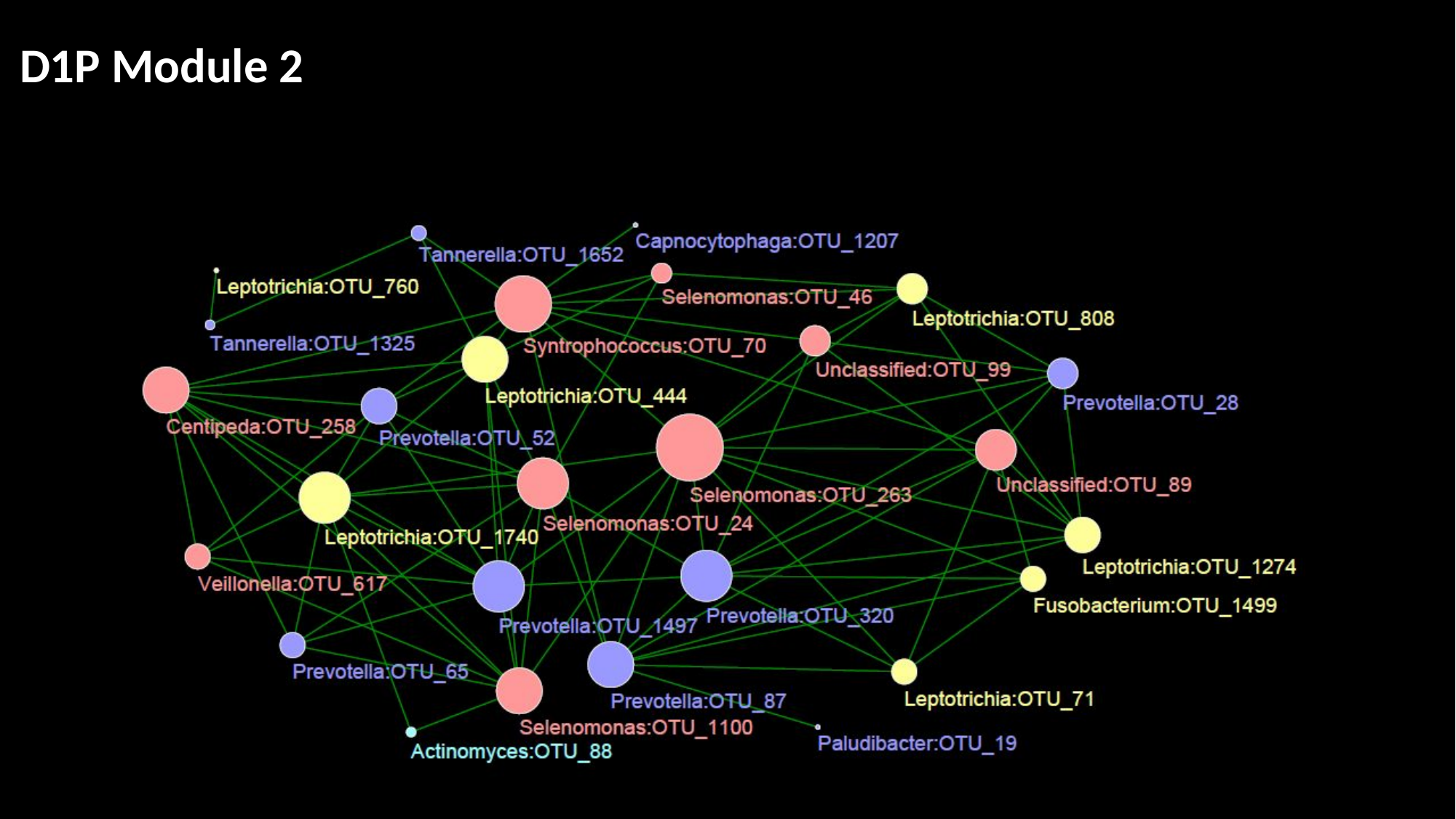

D1P Module 2

## Slide 5
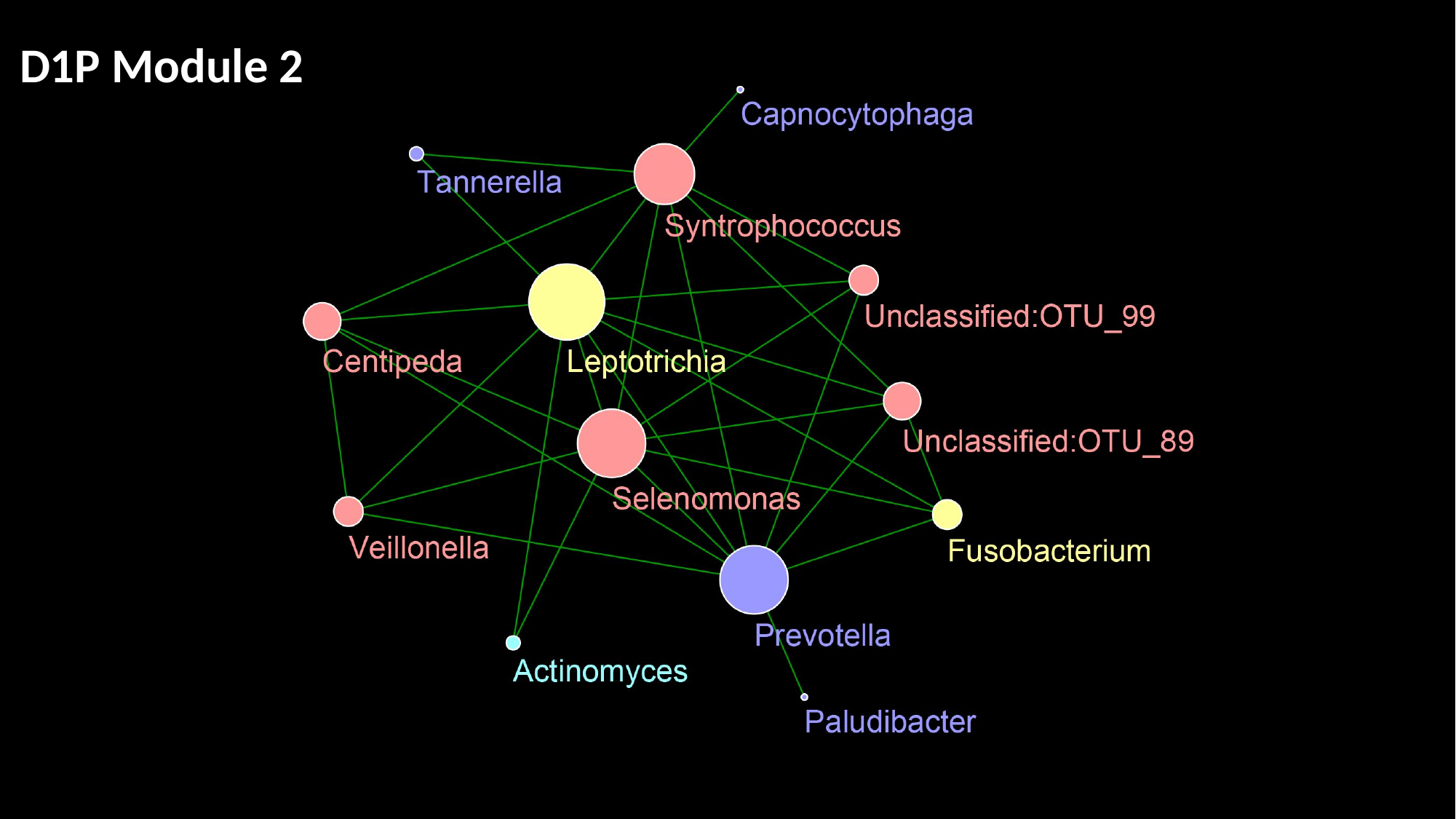

D1P Module 2

## Slide 6
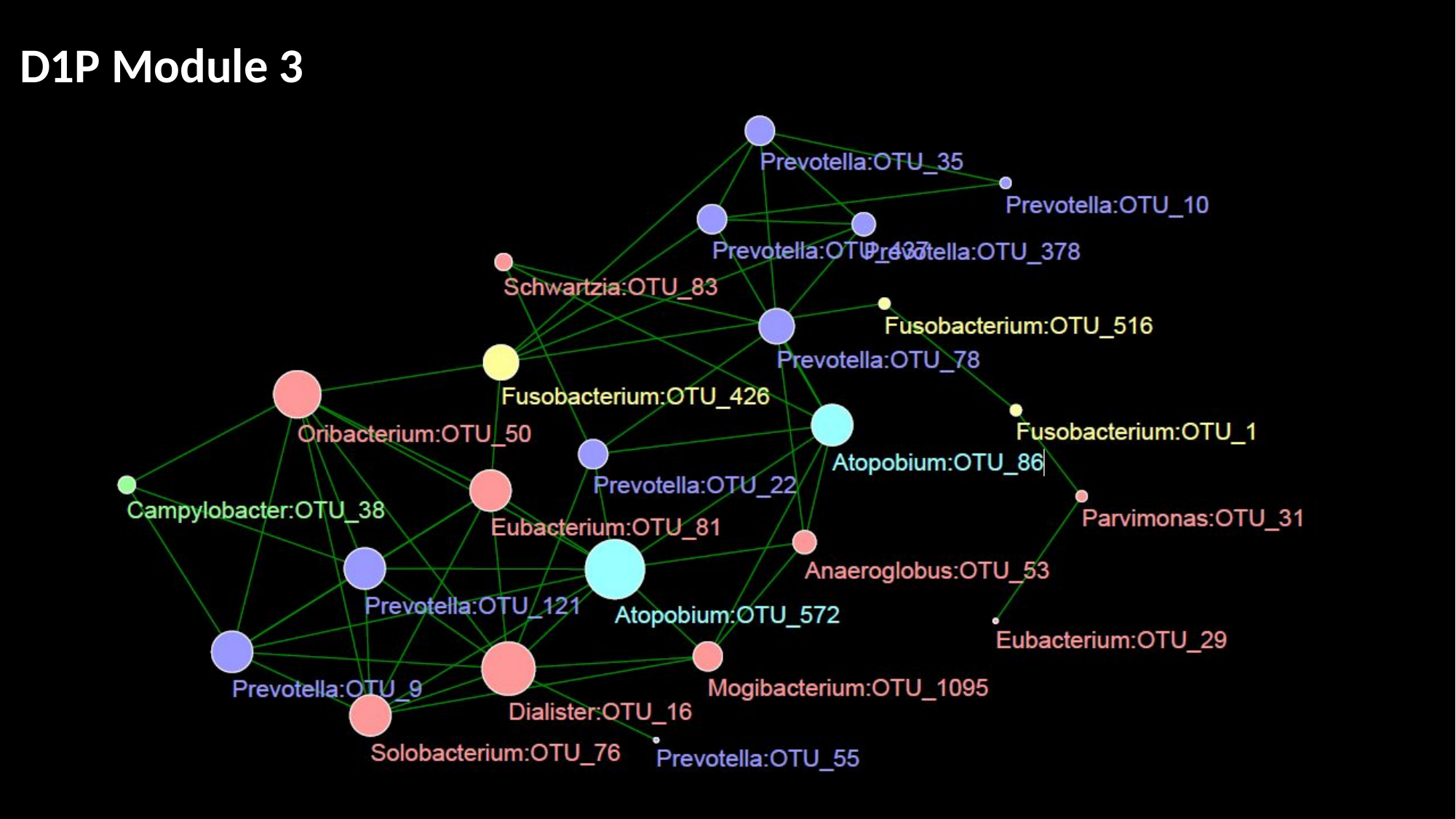

D1P Module 3

## Slide 7
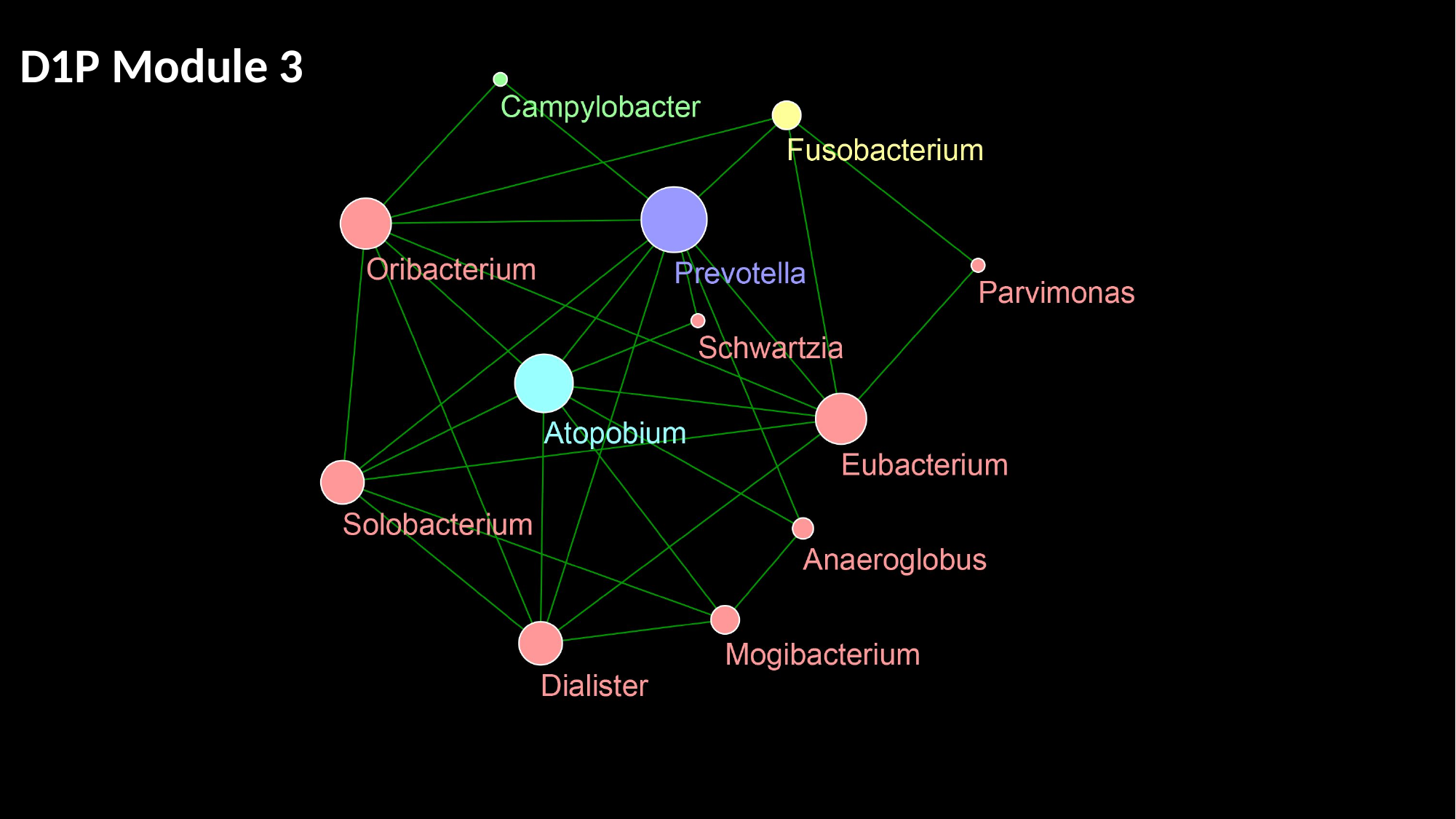

D1P Module 3

## Slide 8
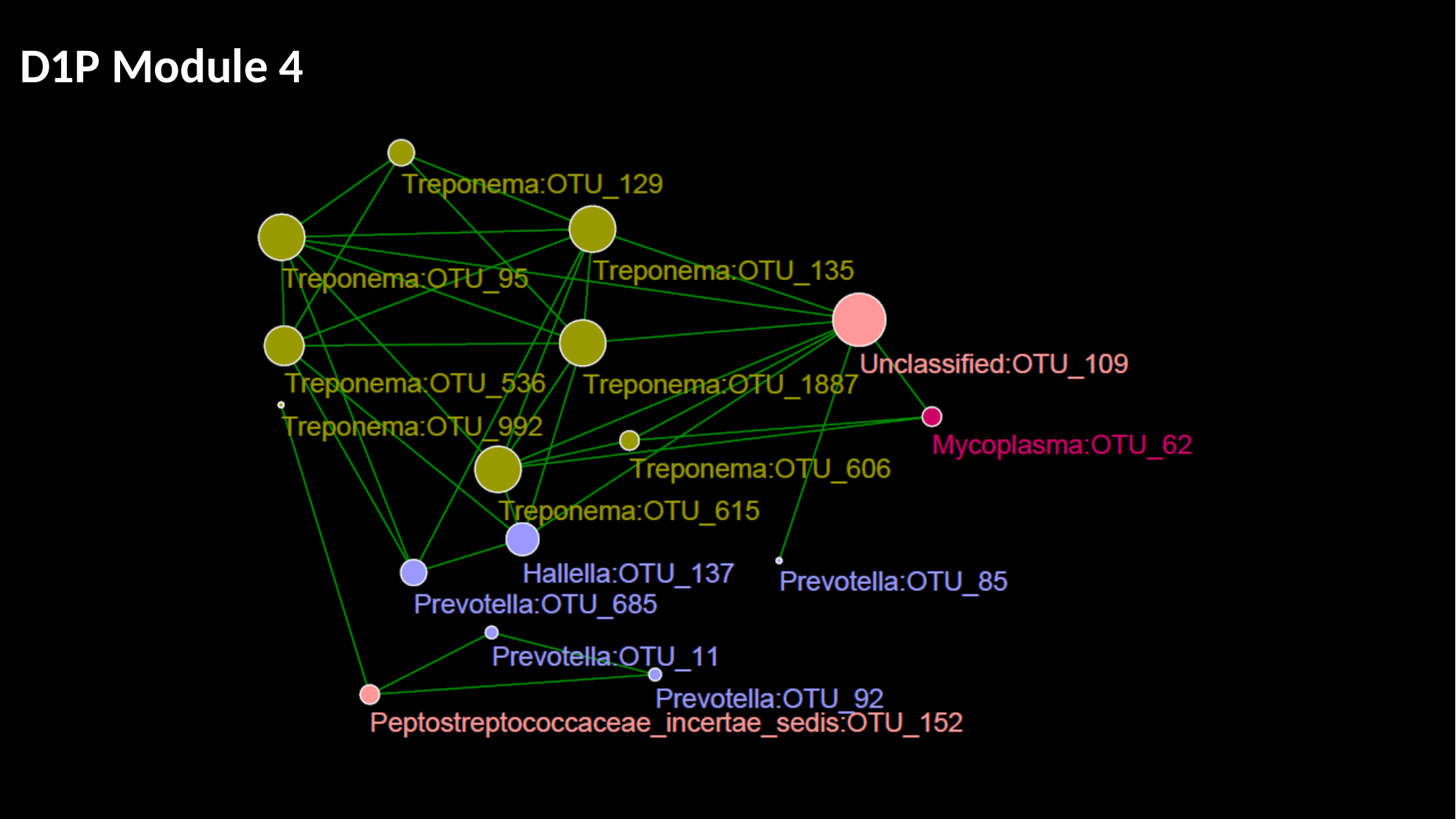

D1P Module 4

## Slide 9
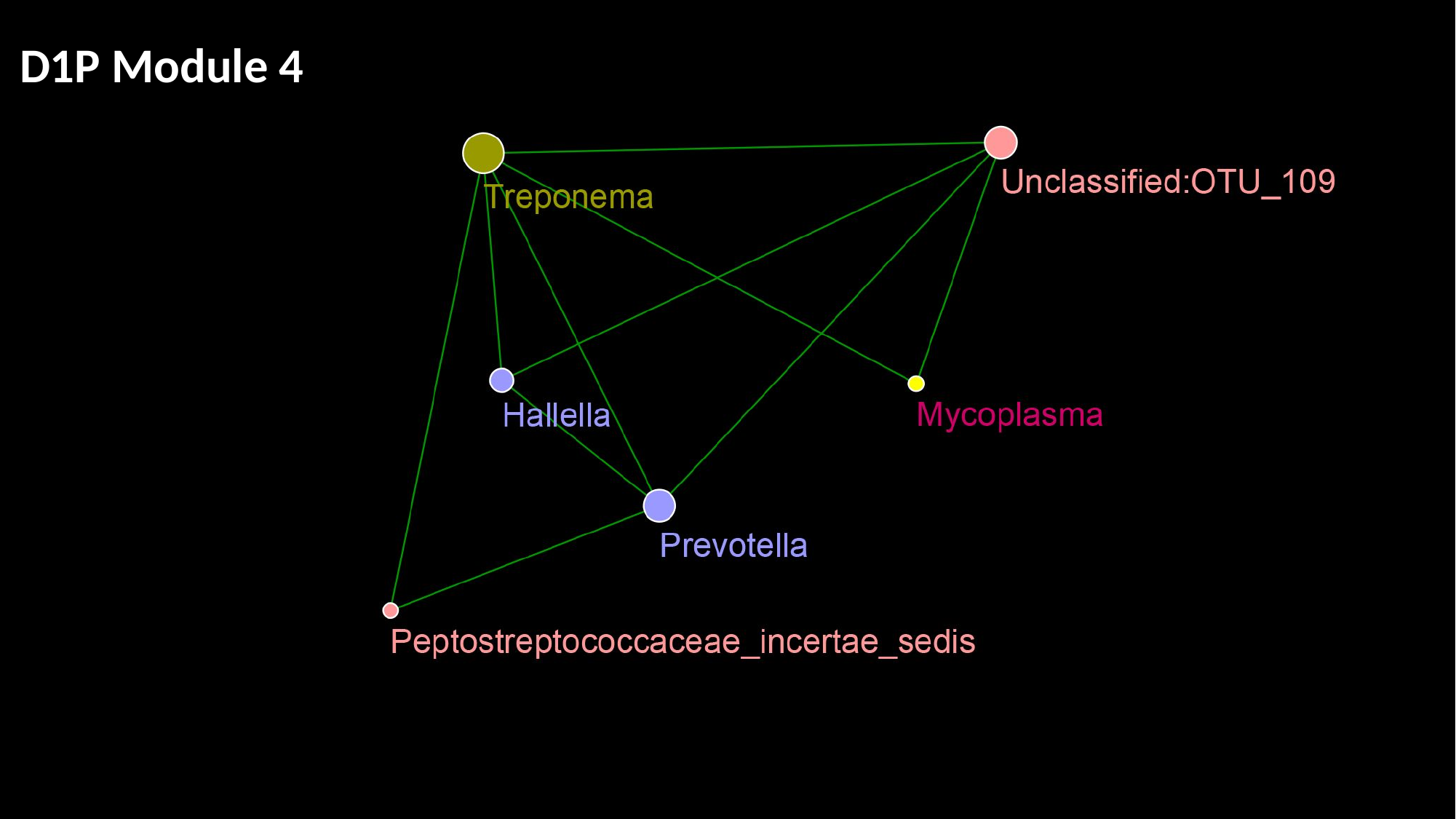

D1P Module 4

## Slide 10
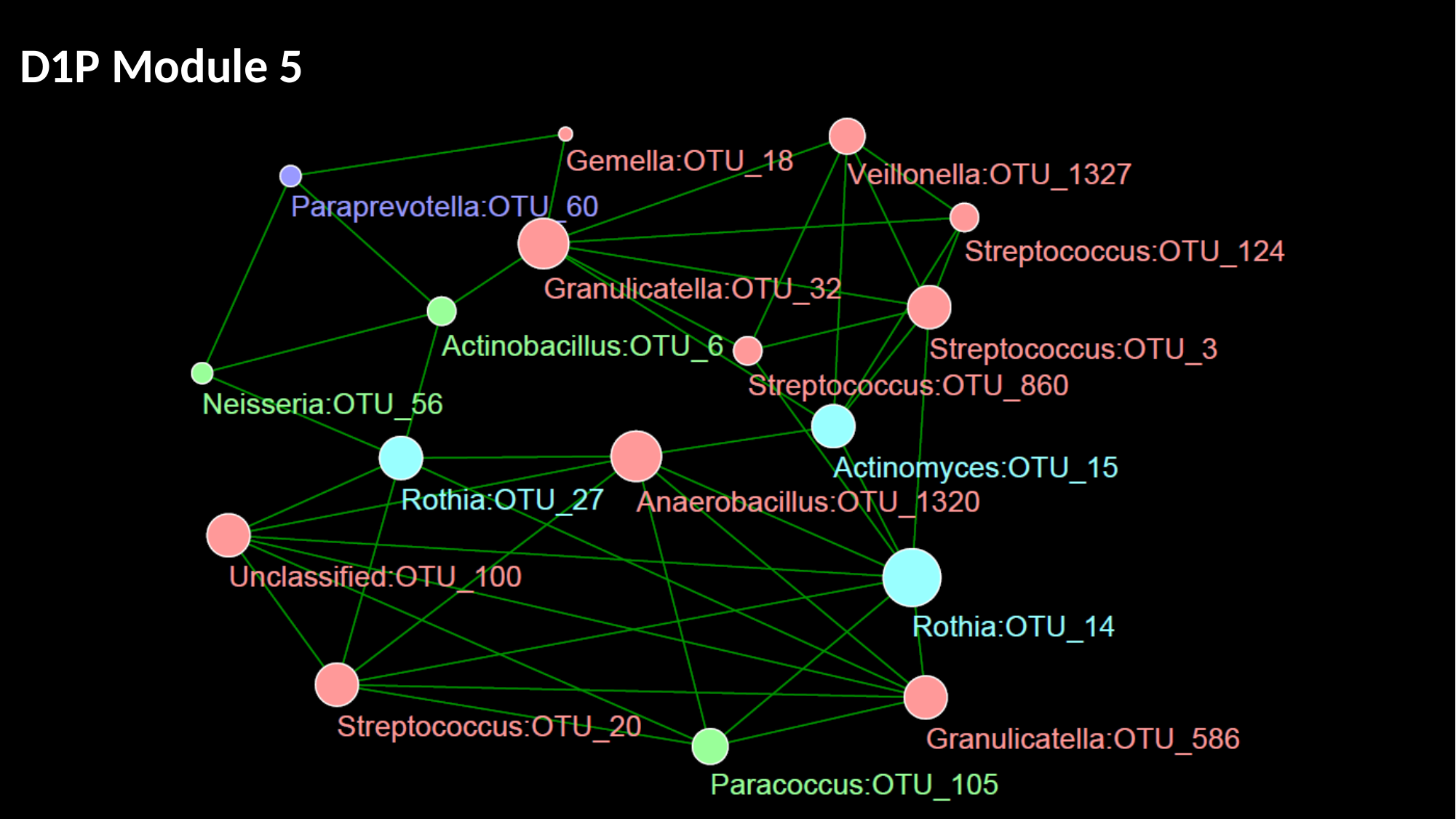

D1P Module 5

## Slide 11
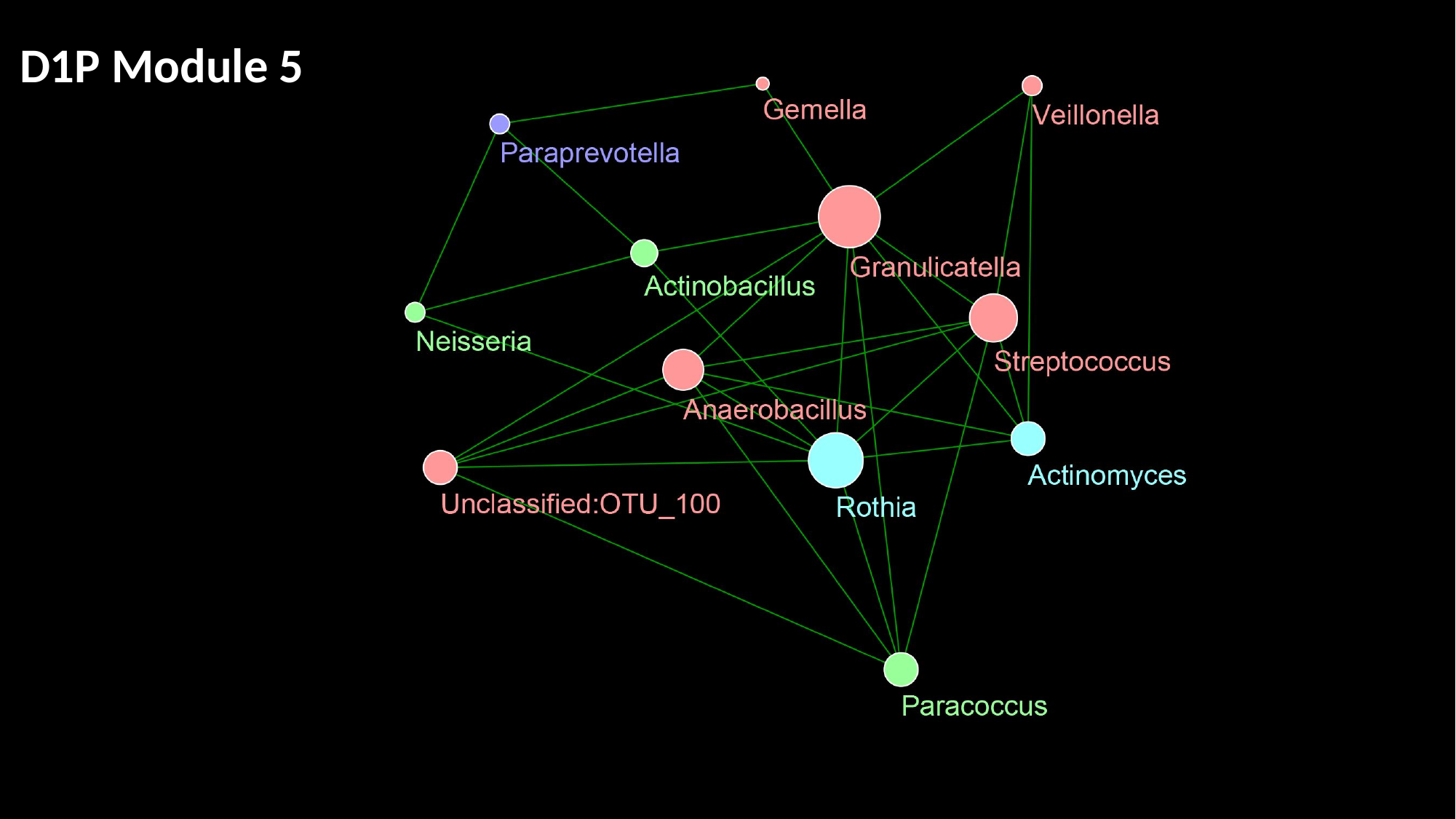

D1P Module 5

## Slide 12
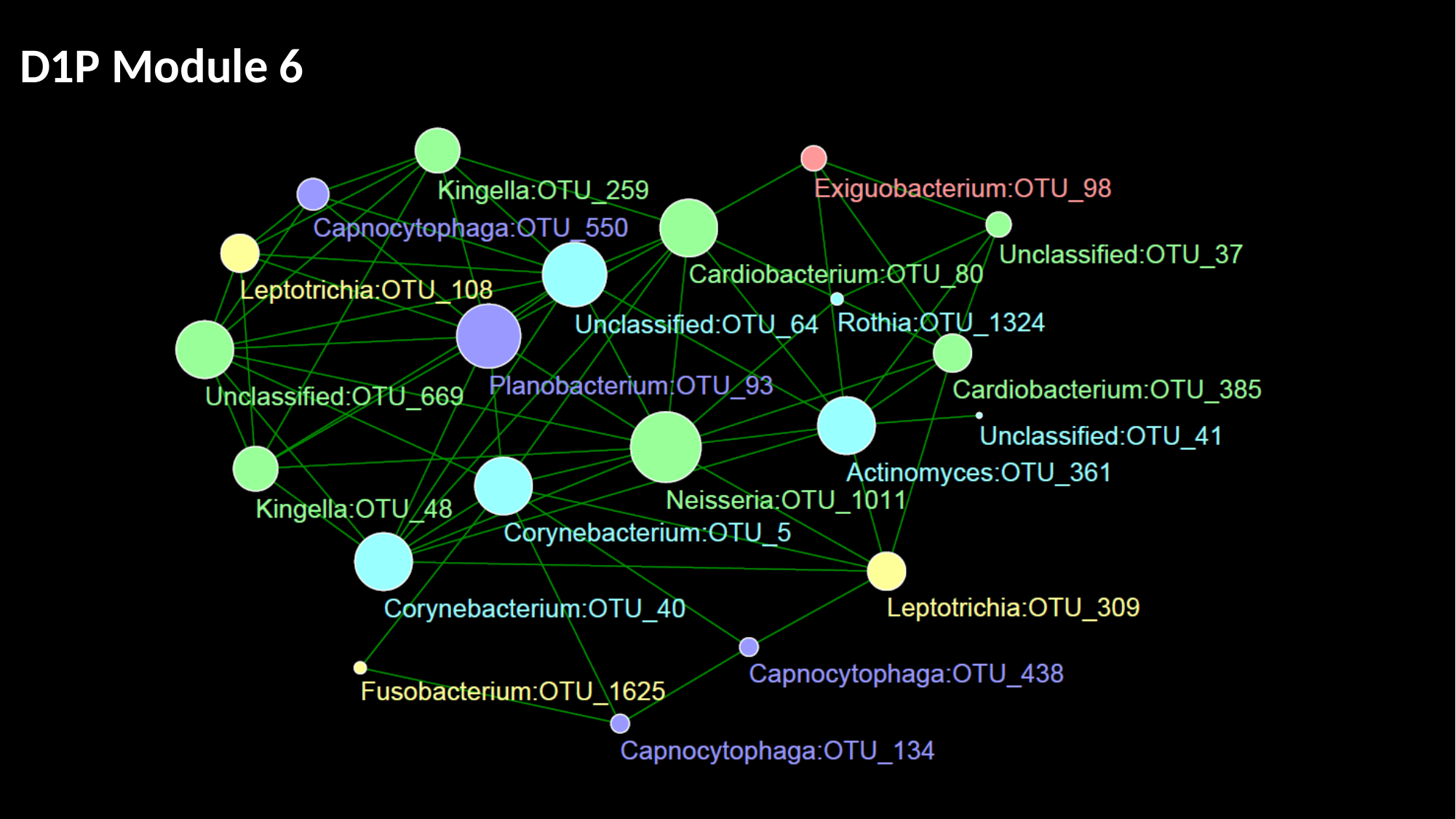

D1P Module 6

## Slide 13
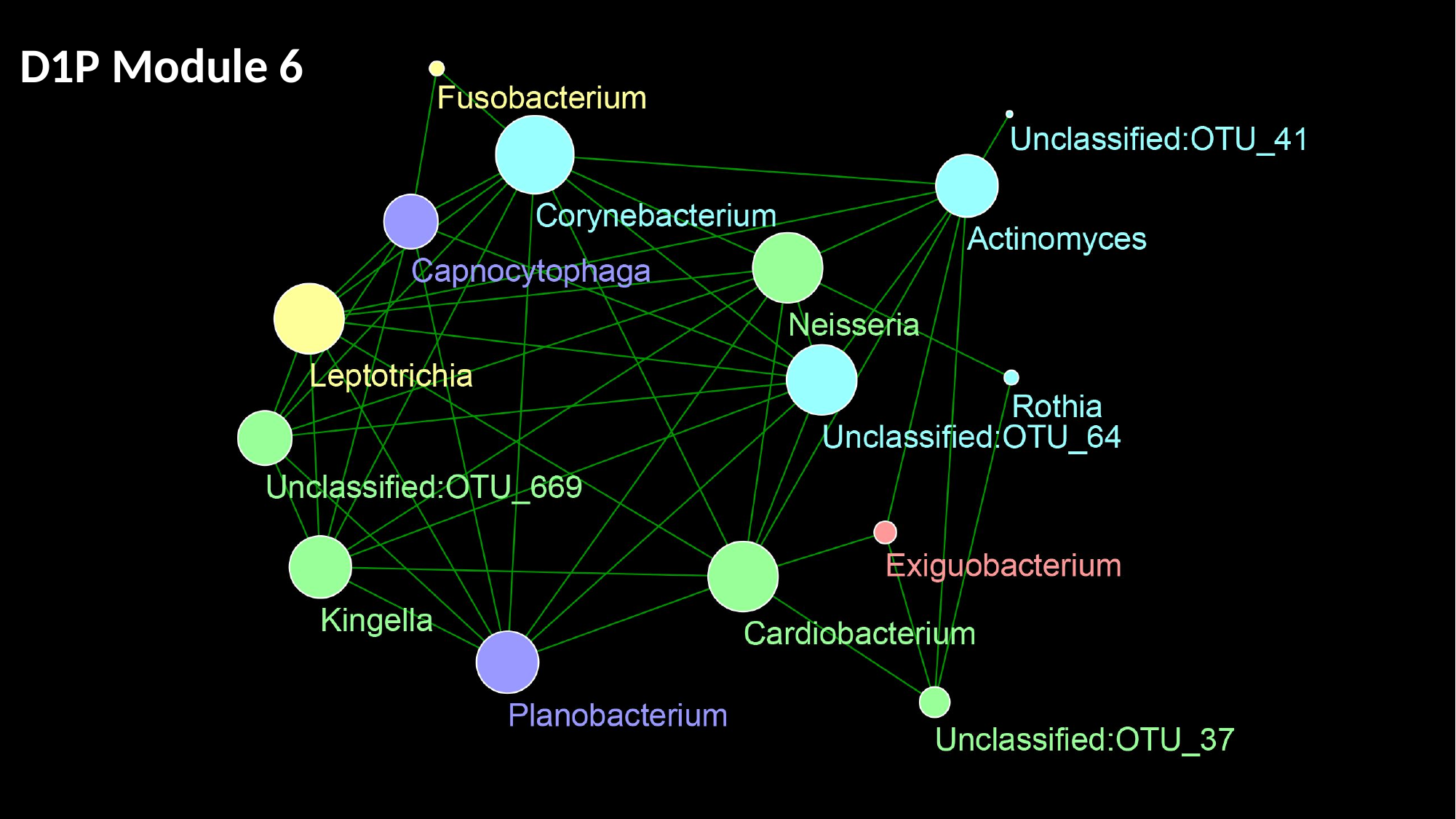

D1P Module 6

## Slide 14
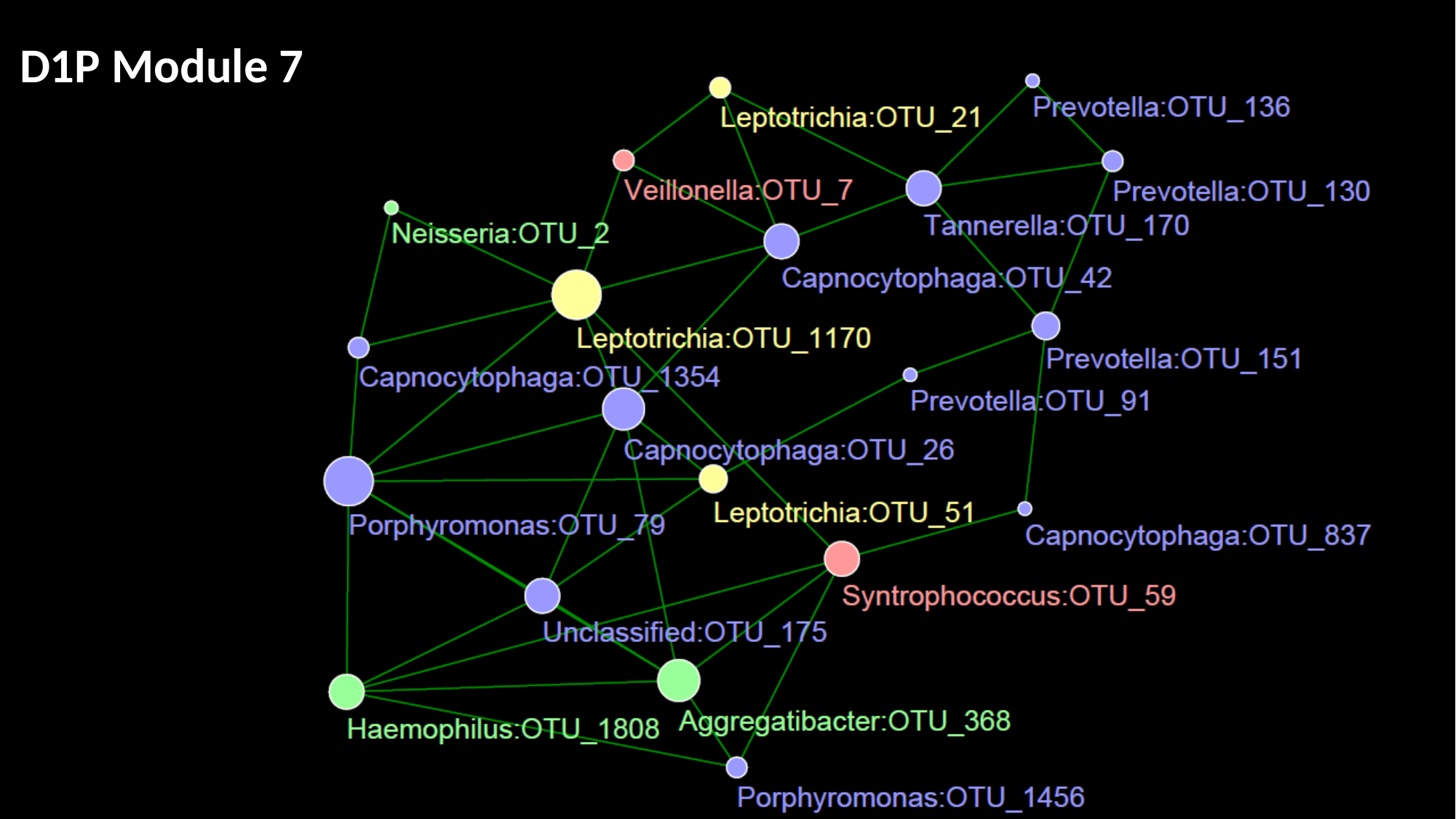

D1P Module 7

## Slide 15
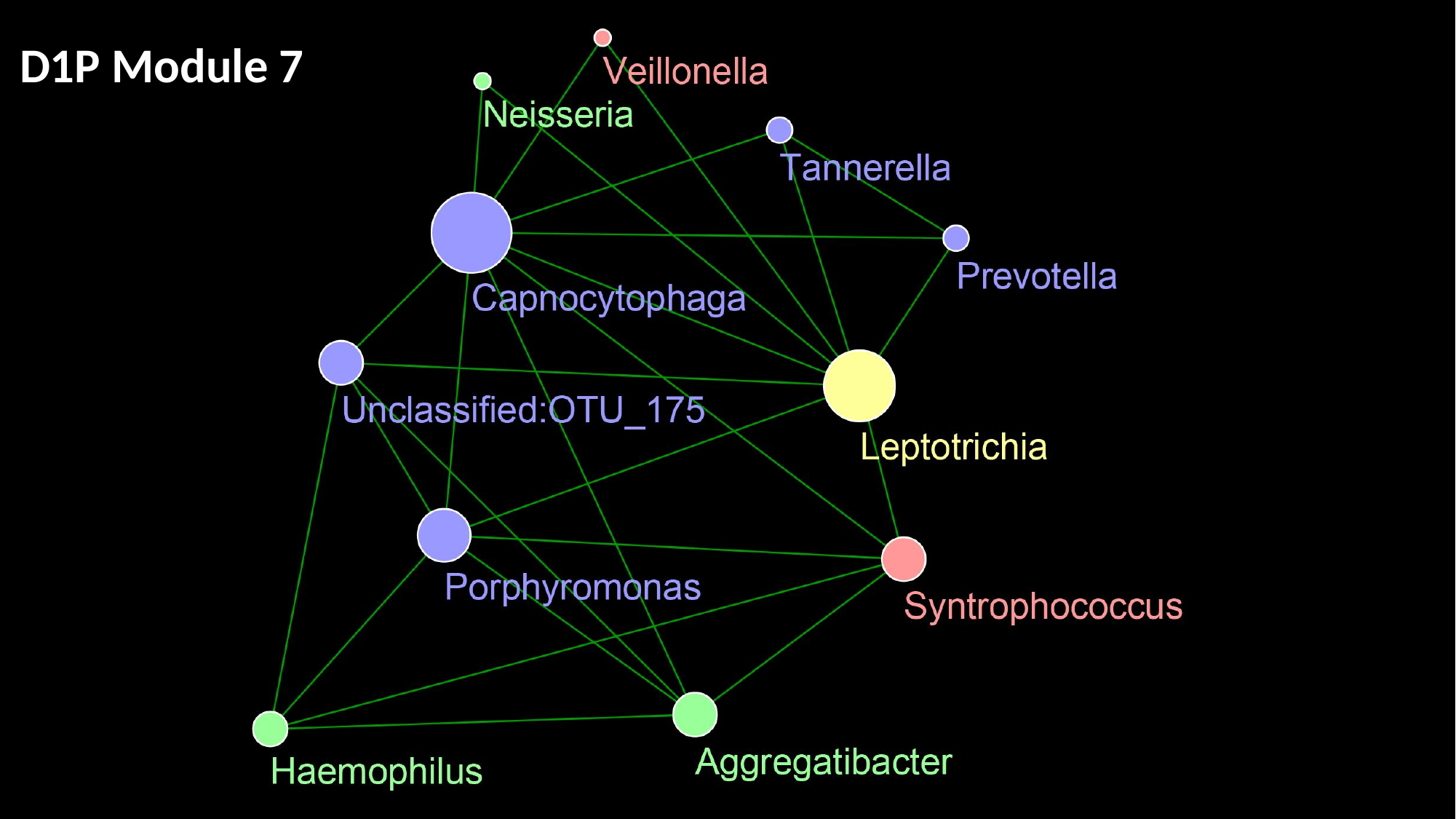

D1P Module 7

## Slide 16
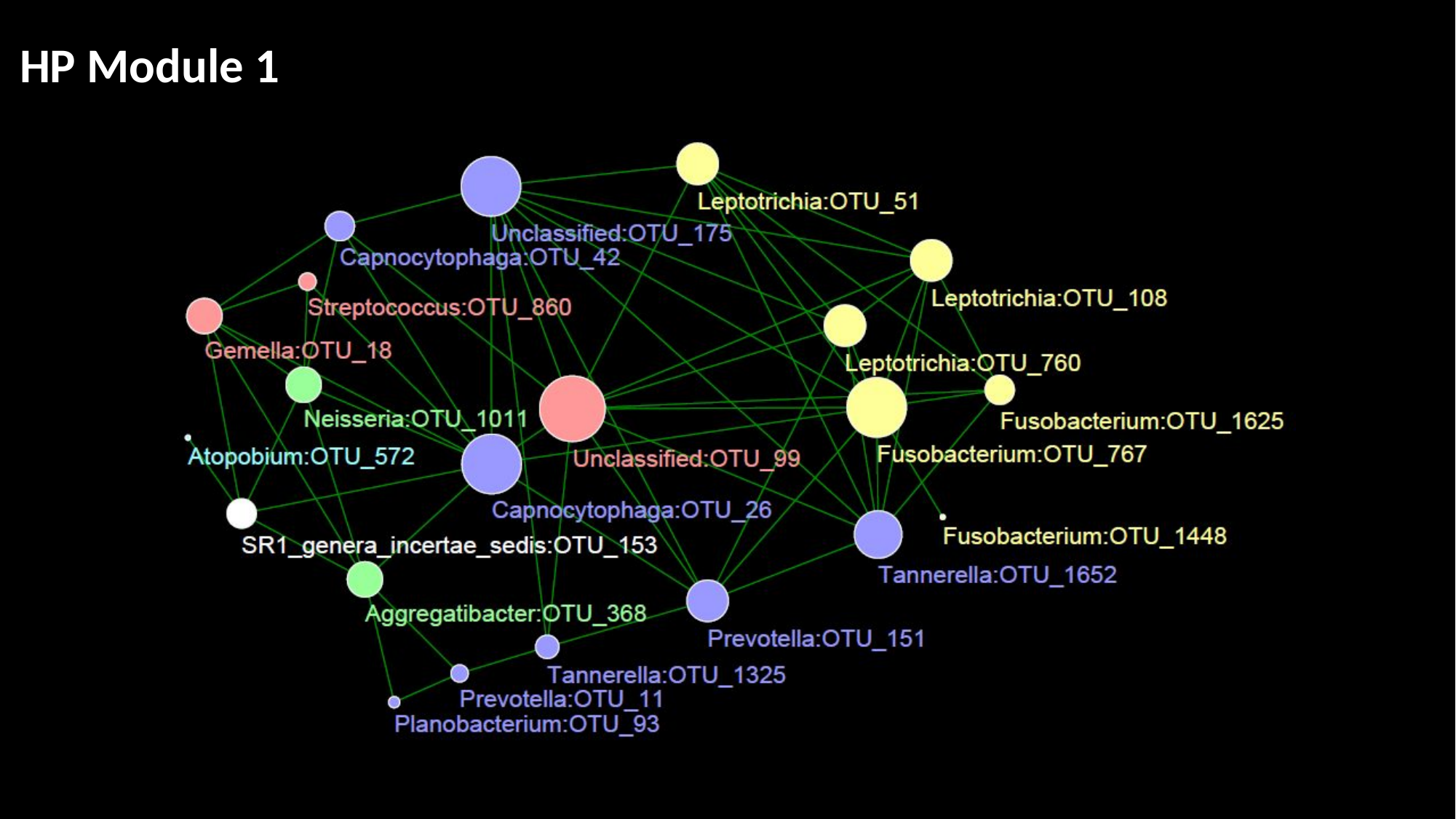

HP Module 1

## Slide 17
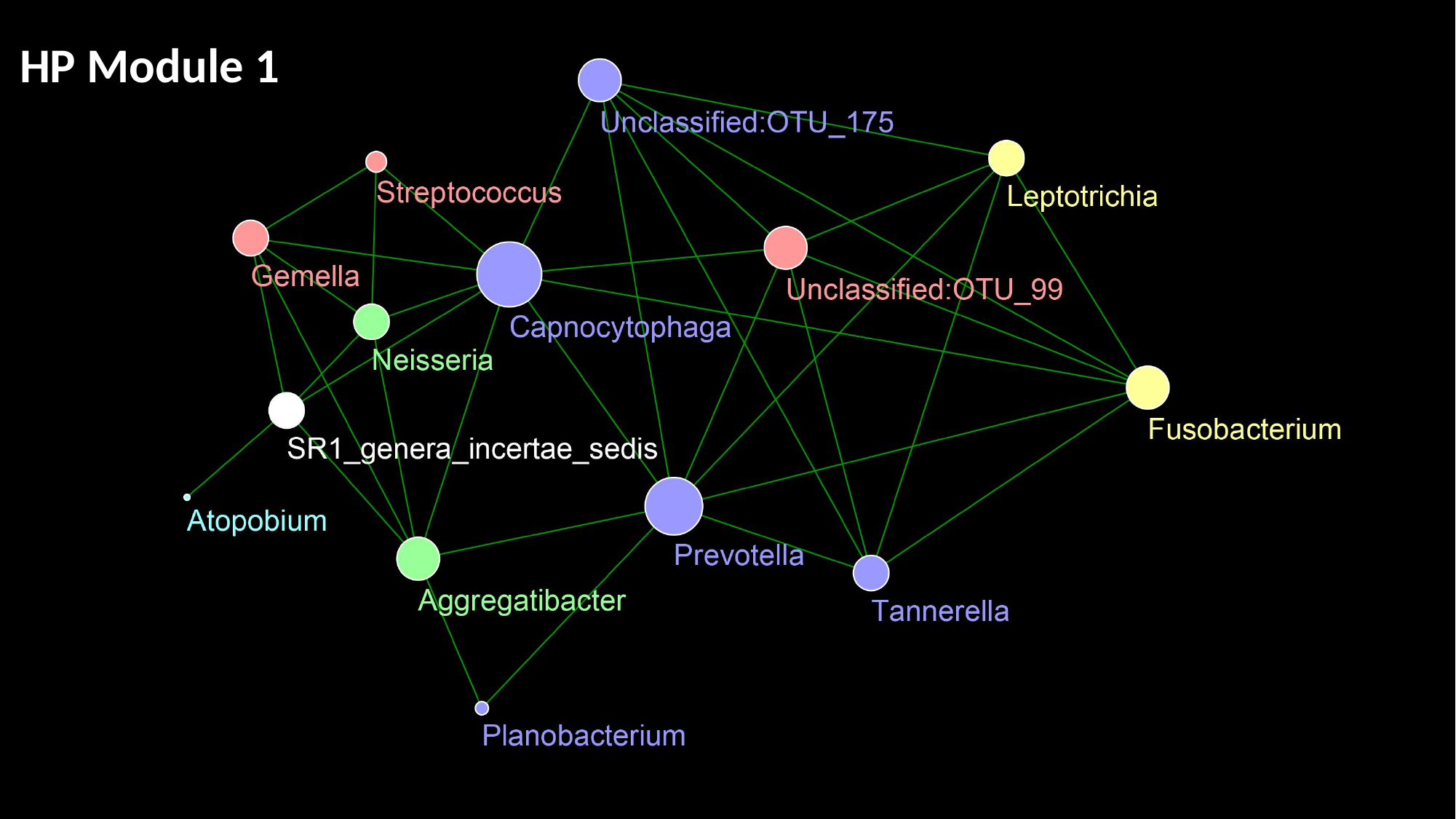

HP Module 1

## Slide 18
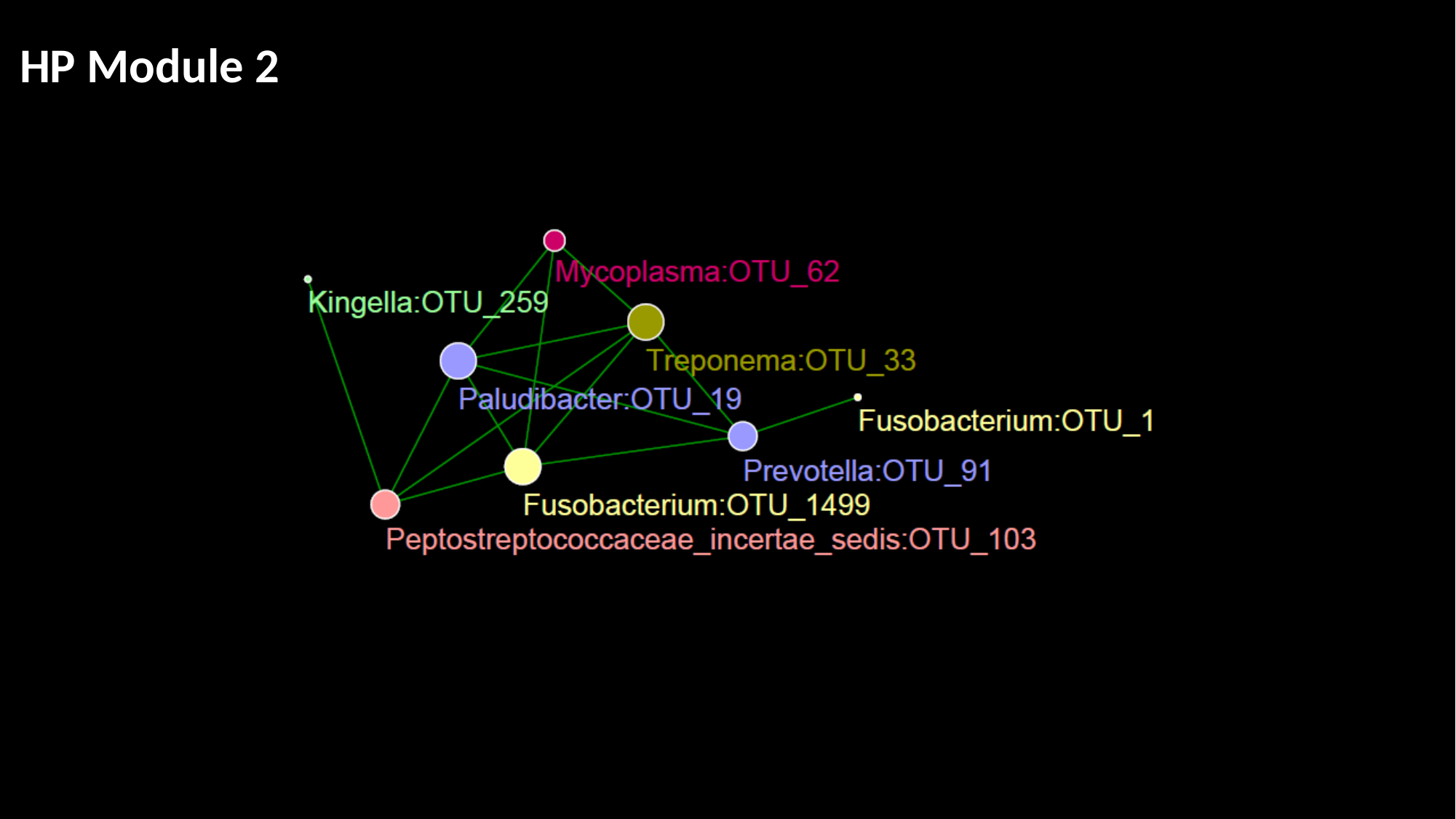

HP Module 2

## Slide 19
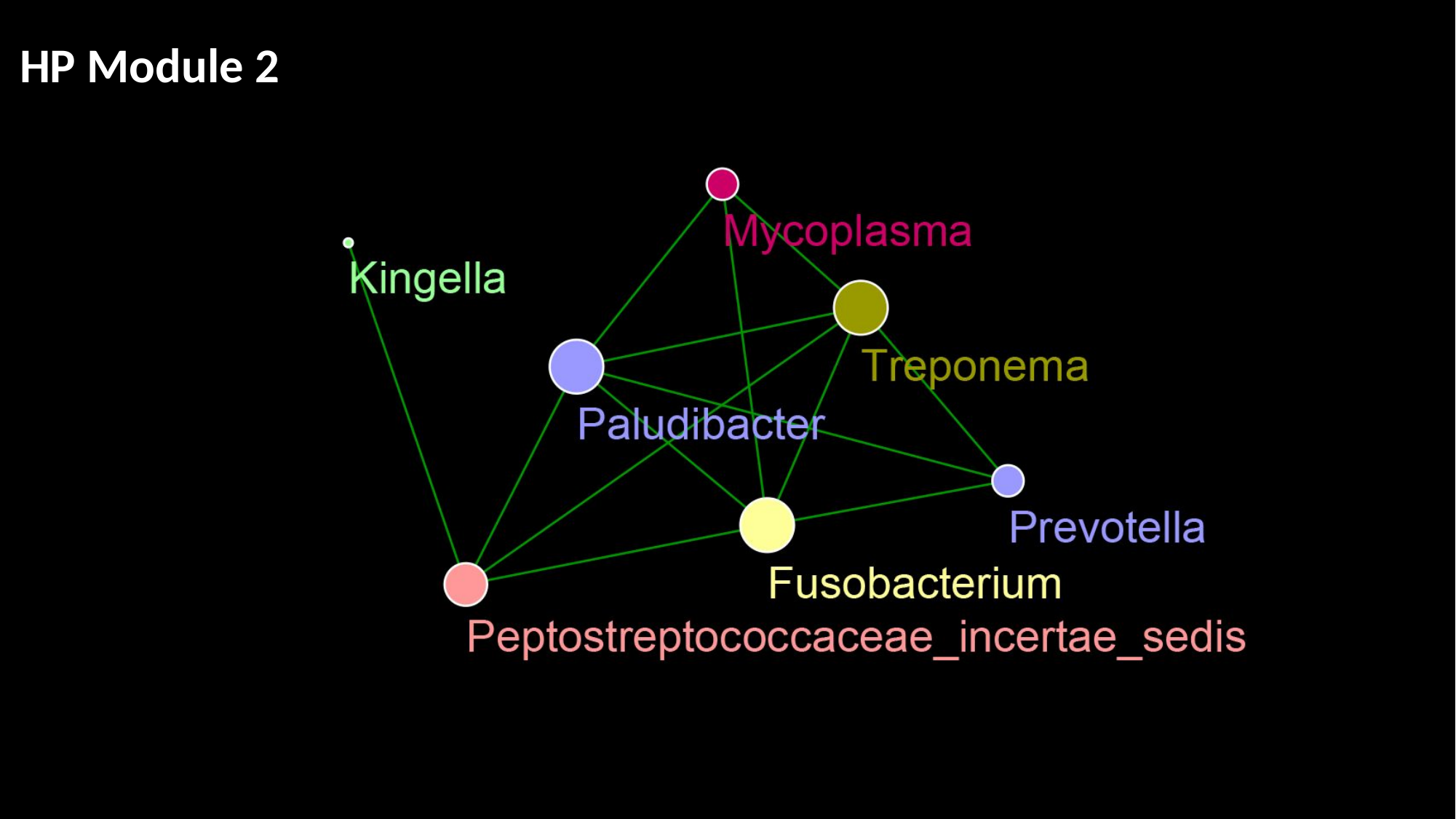

HP Module 2

## Slide 20
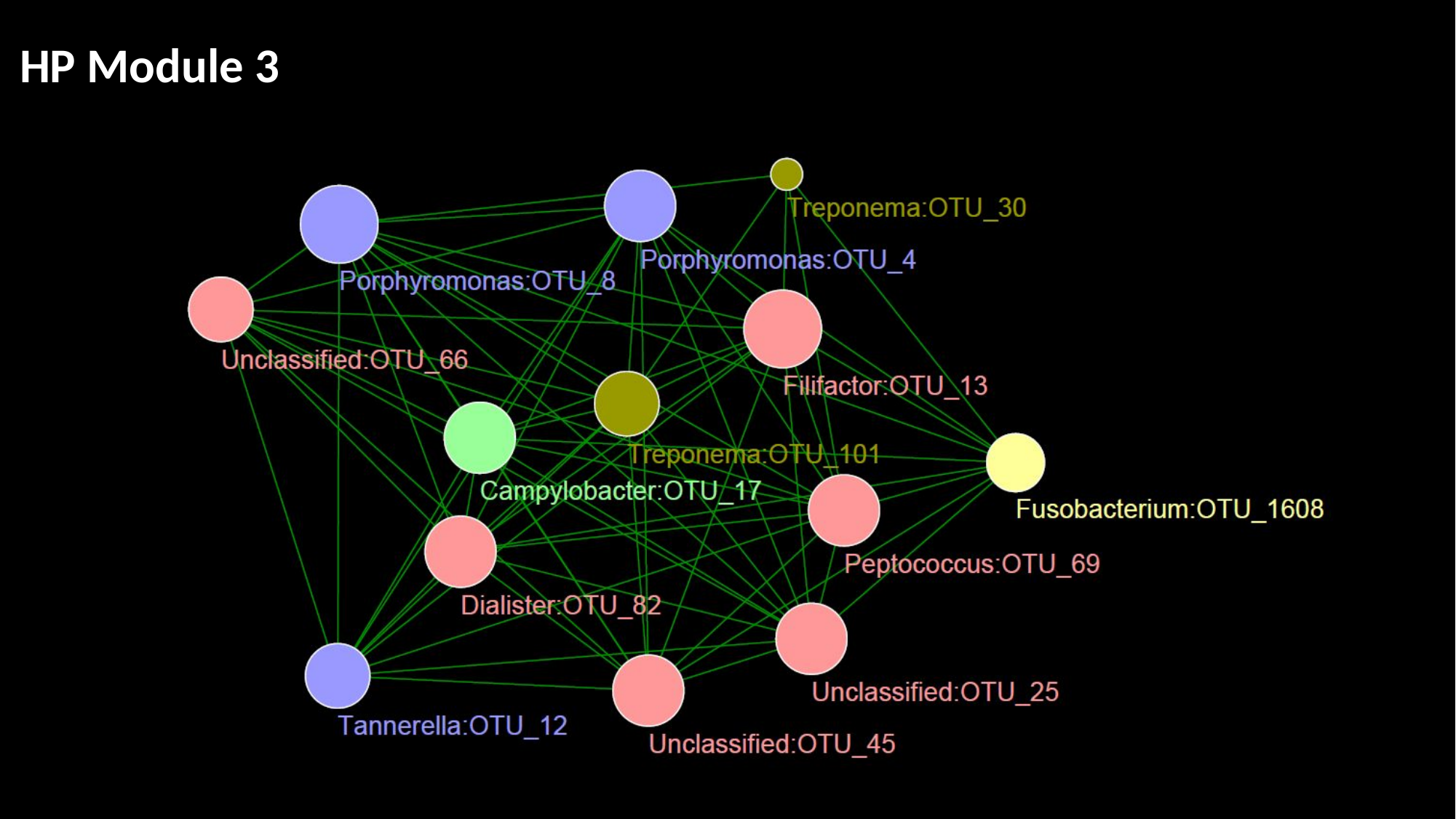

HP Module 3

## Slide 21
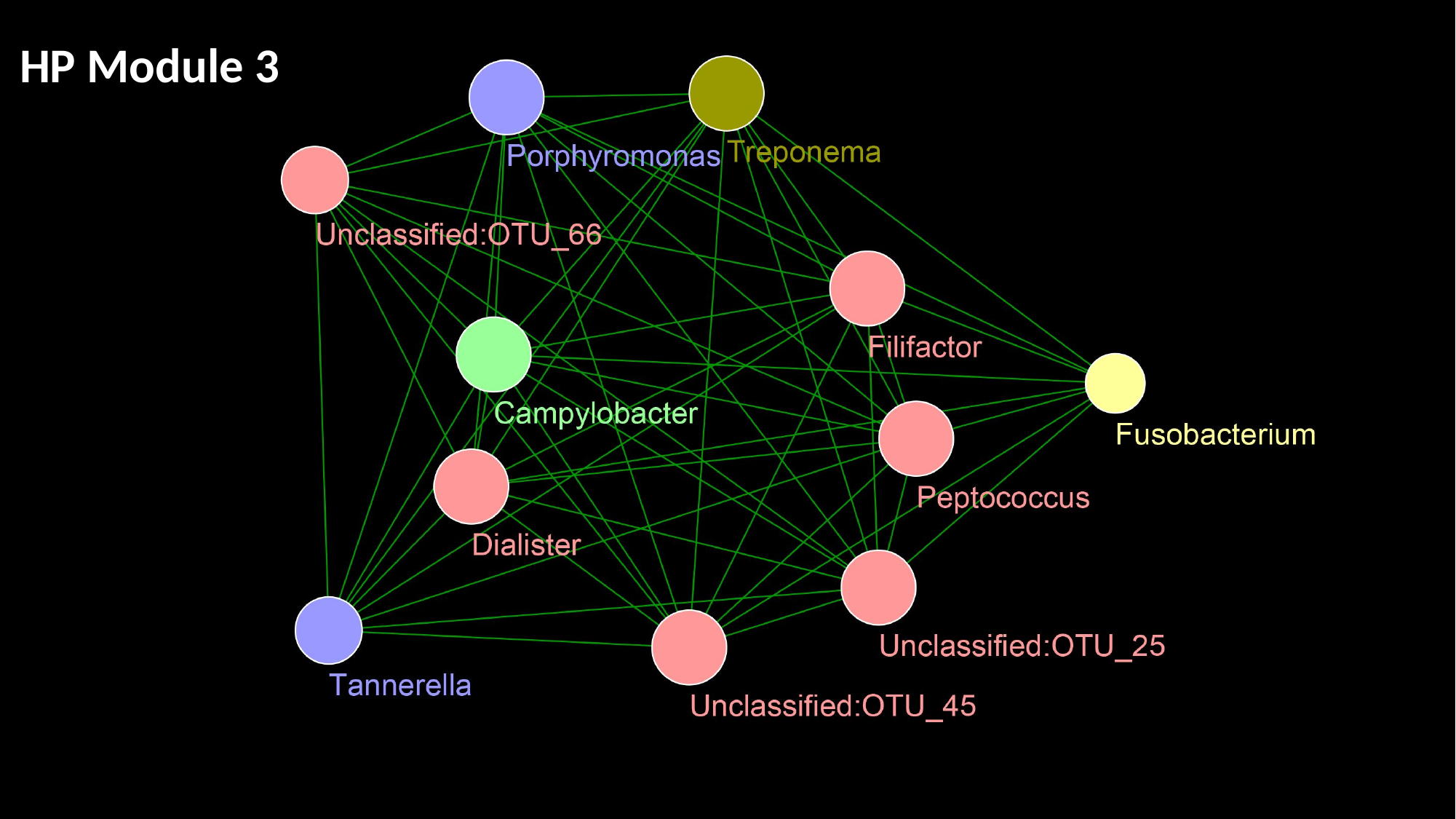

HP Module 3

## Slide 22
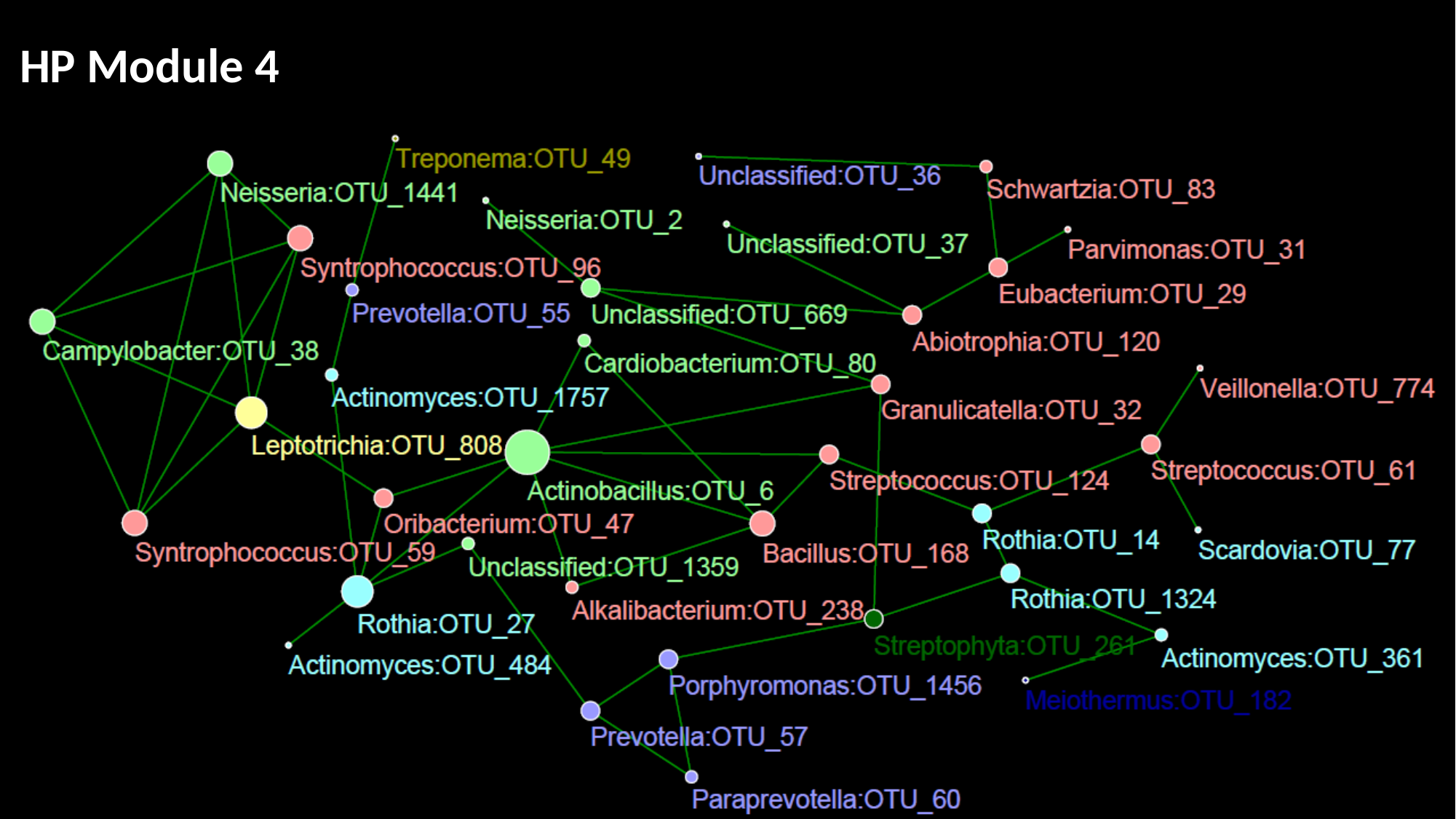

HP Module 4

## Slide 23
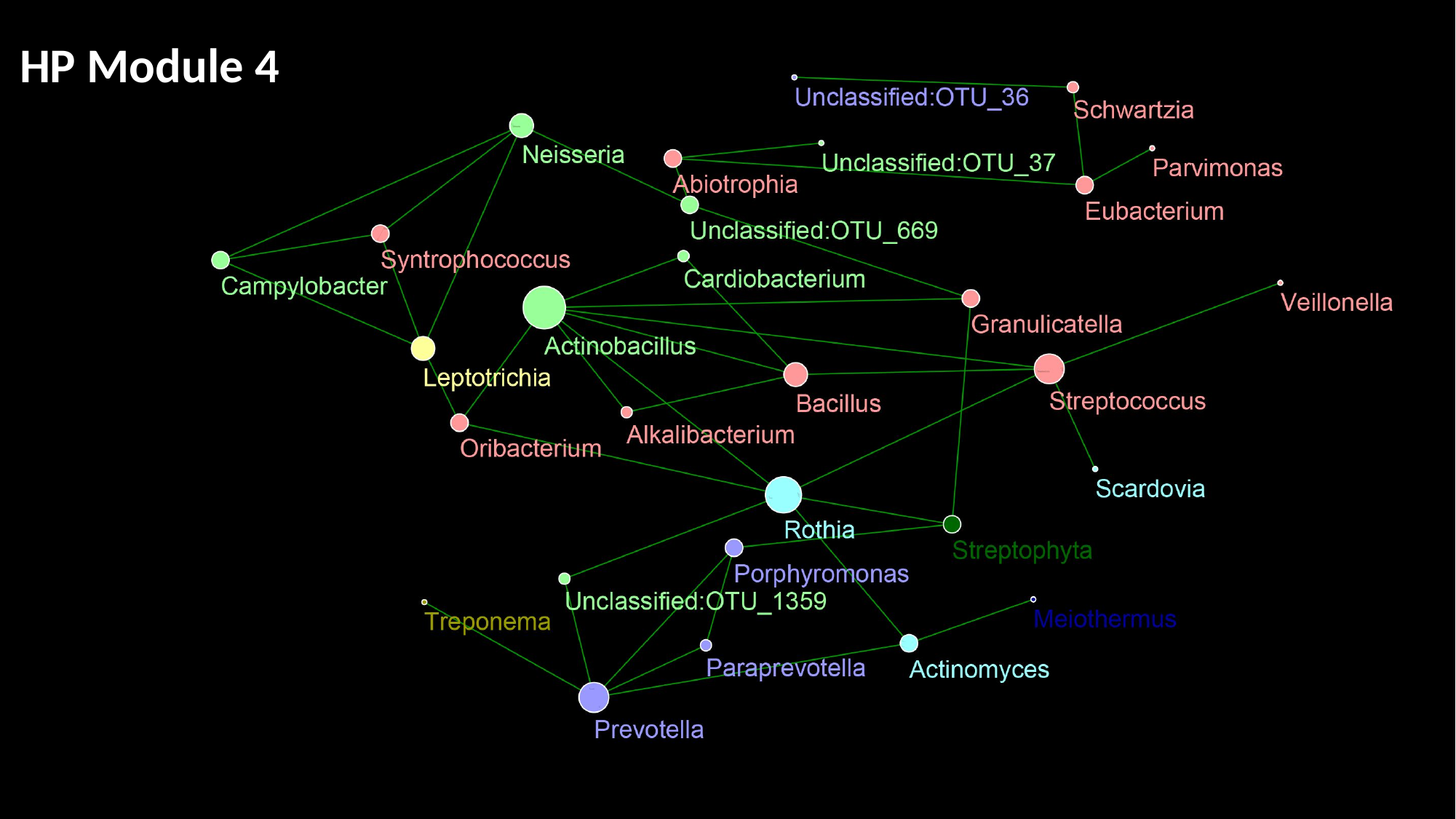

HP Module 4

## Slide 24
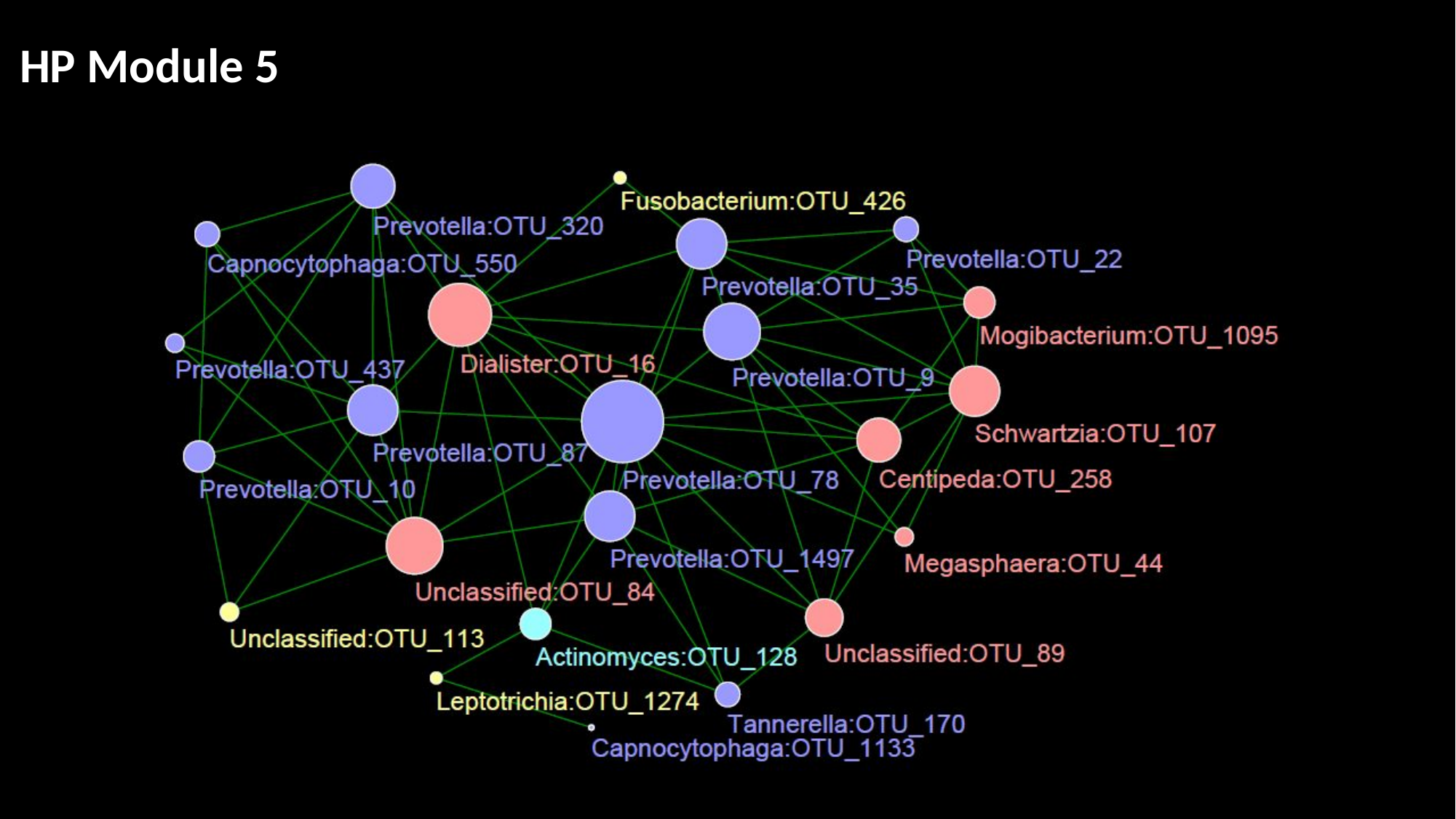

HP Module 5

## Slide 25
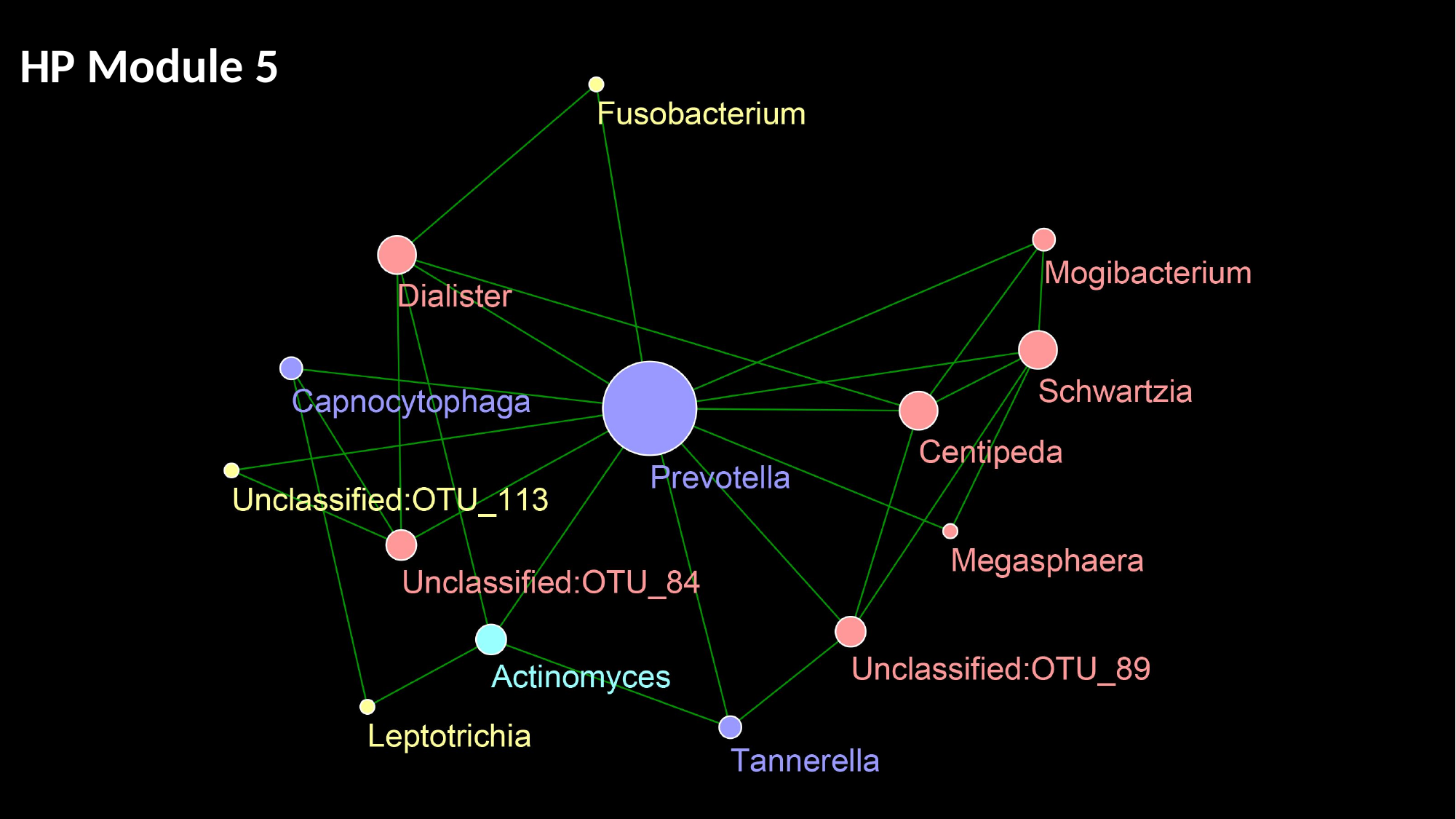

HP Module 5

## Slide 26
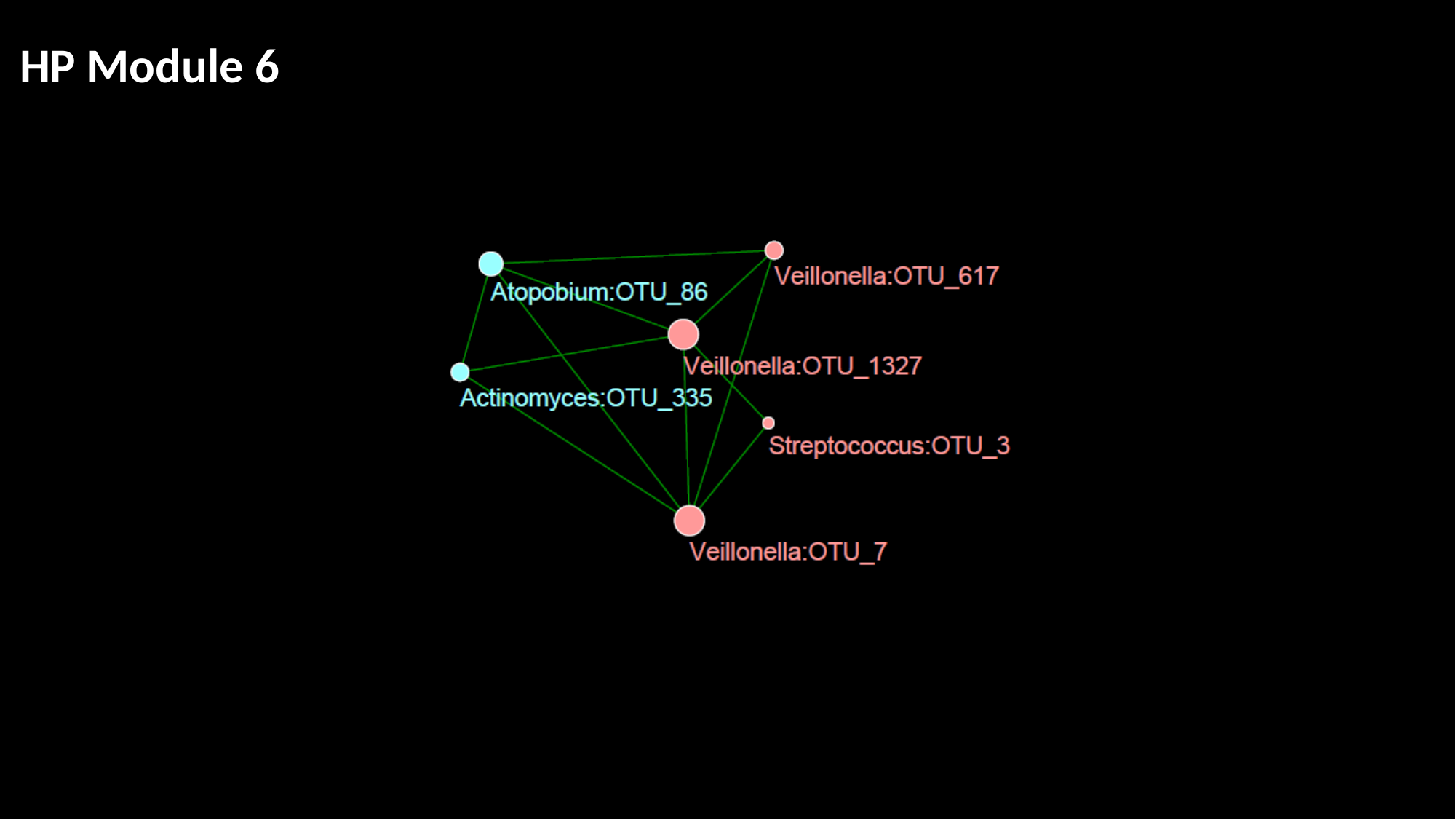

HP Module 6

## Slide 27
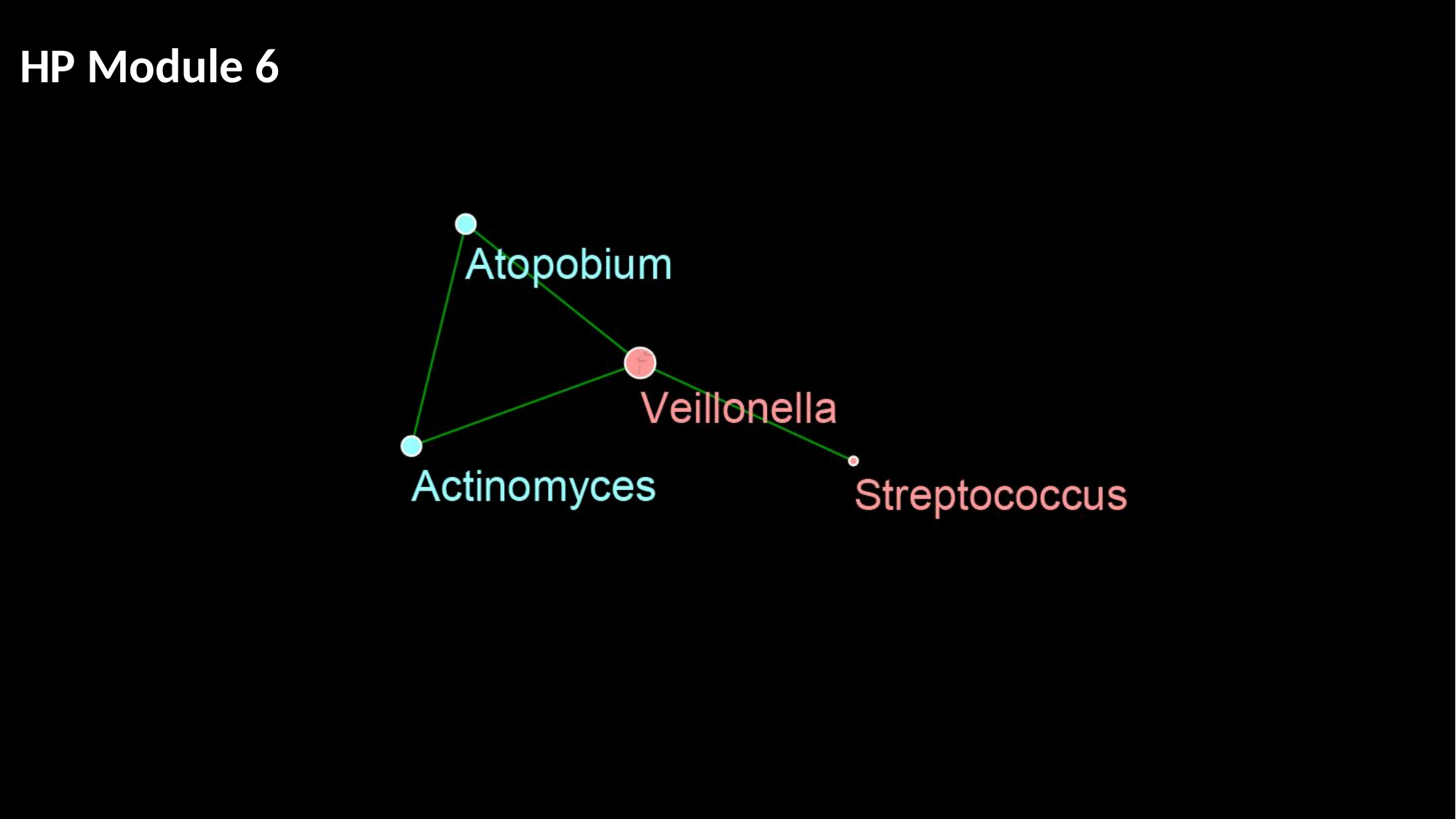

HP Module 6

## Slide 28
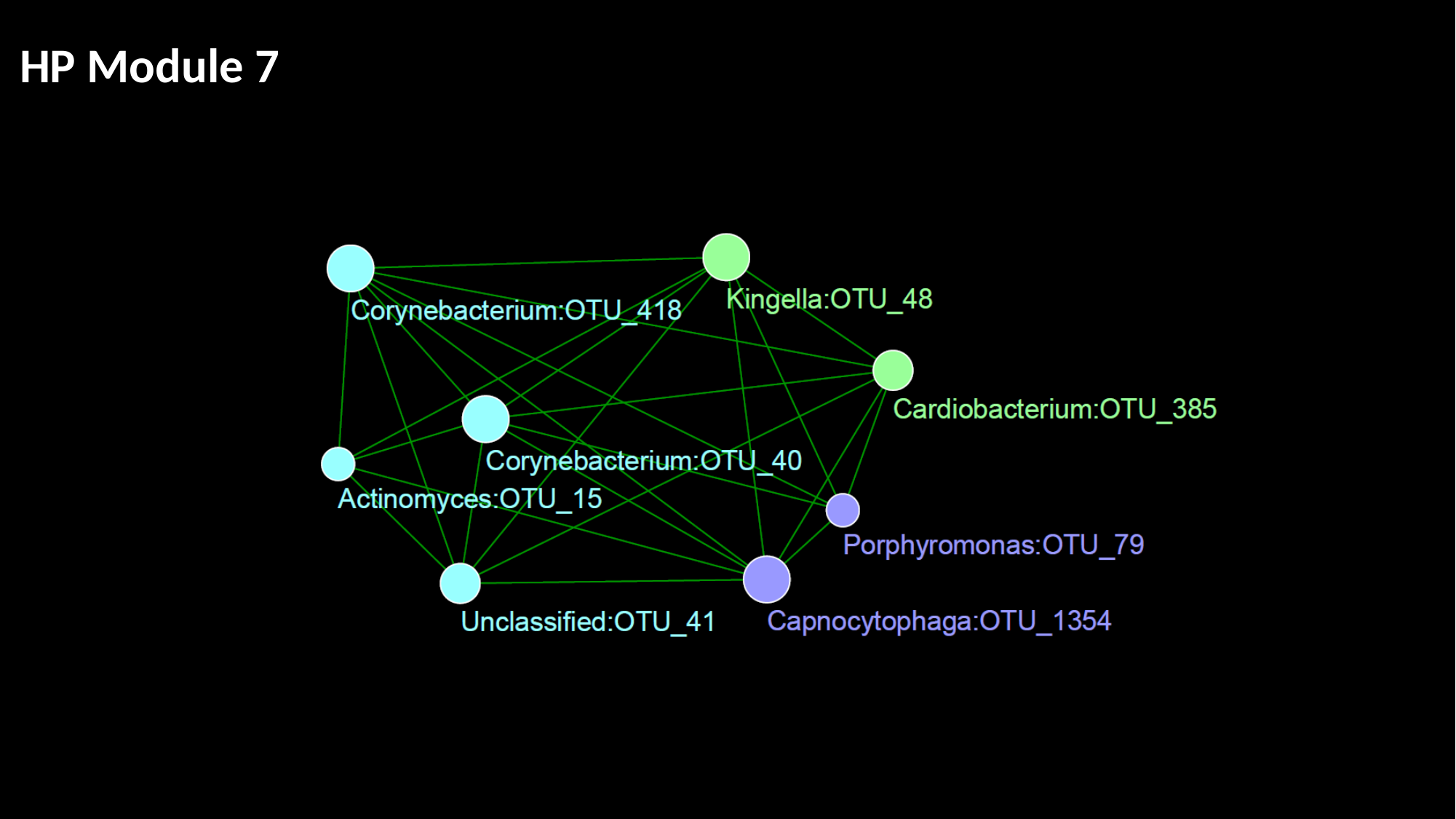

HP Module 7

## Slide 29
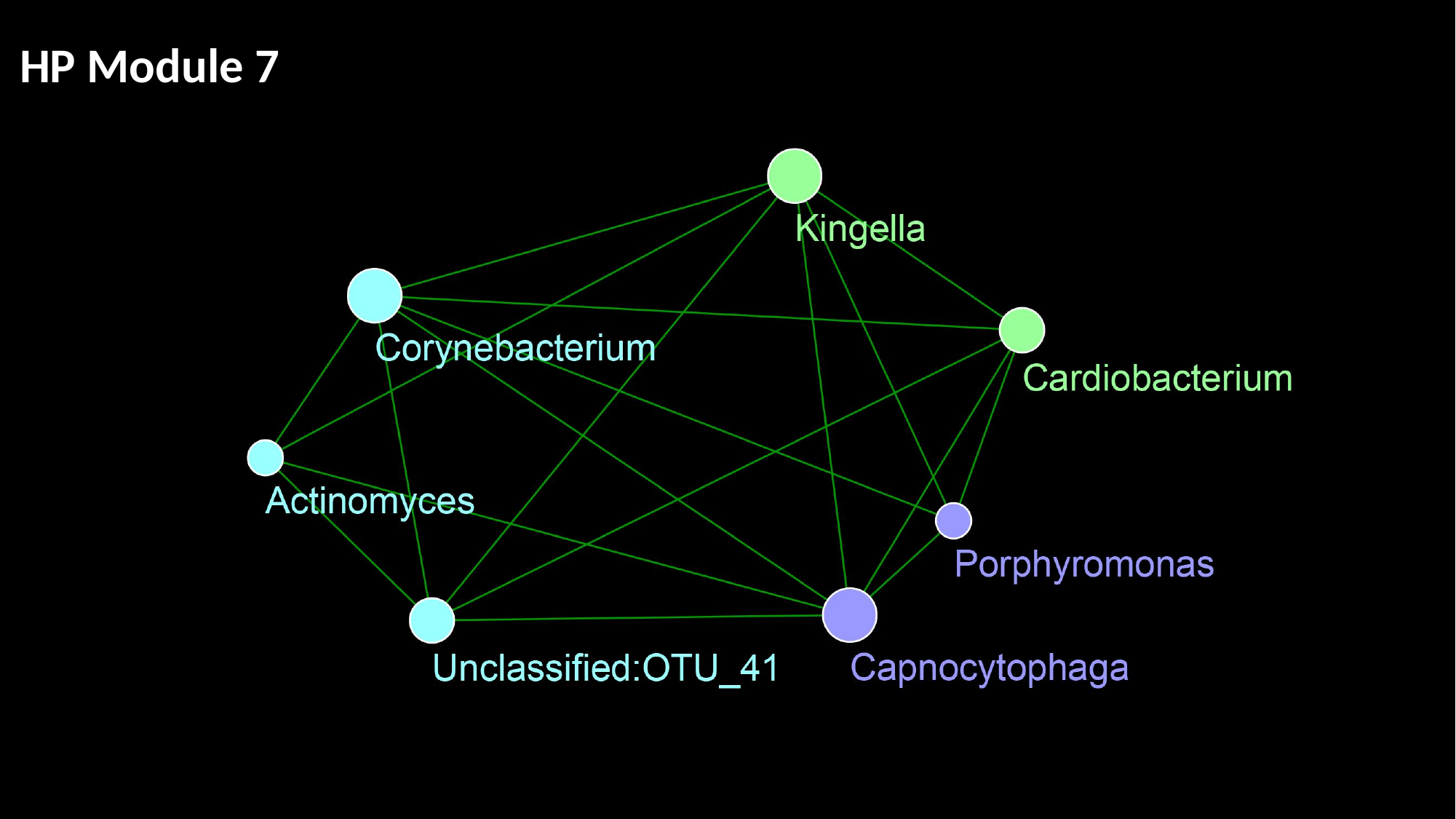

HP Module 7

## Slide 30
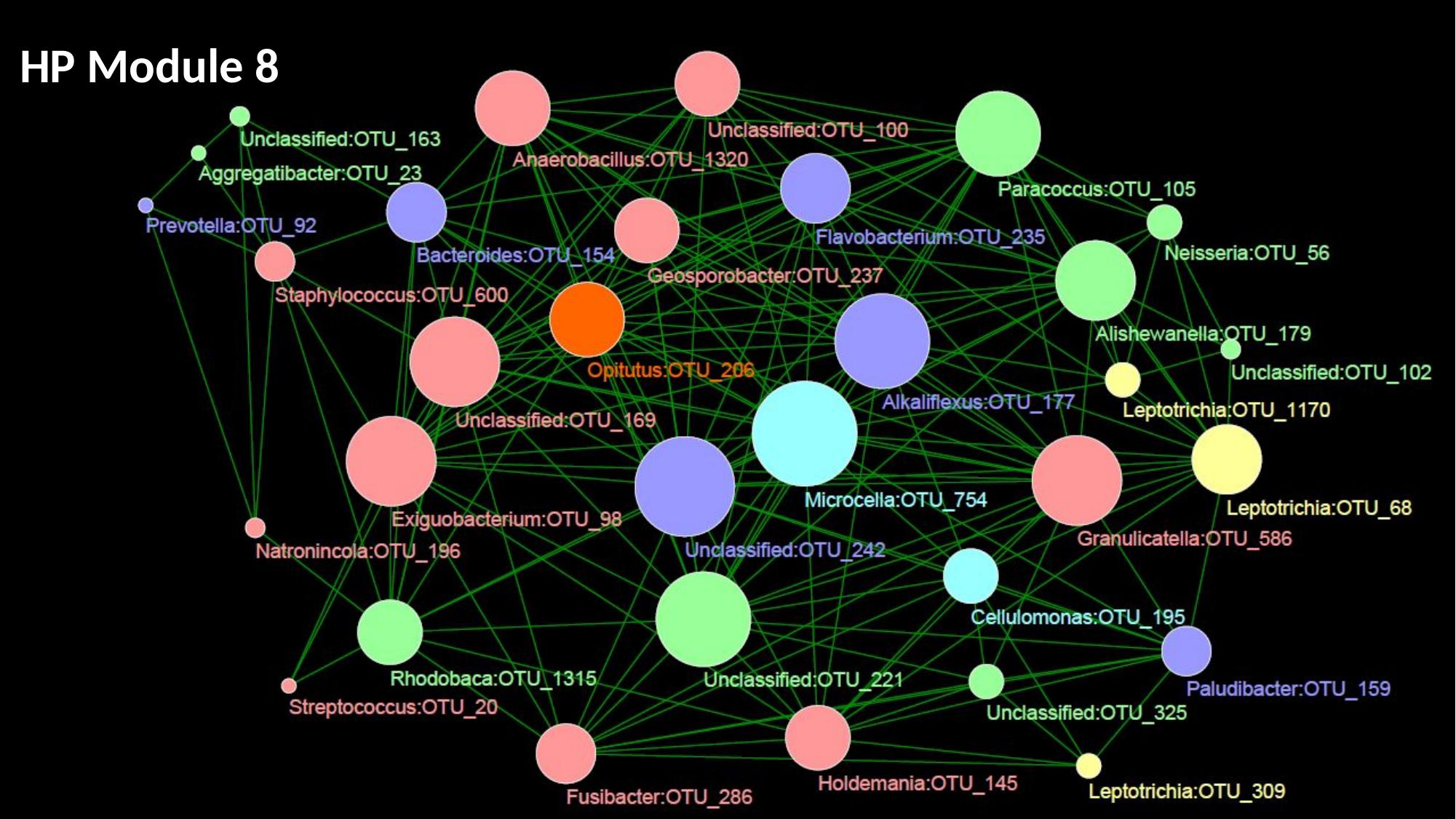

HP Module 8

## Slide 31
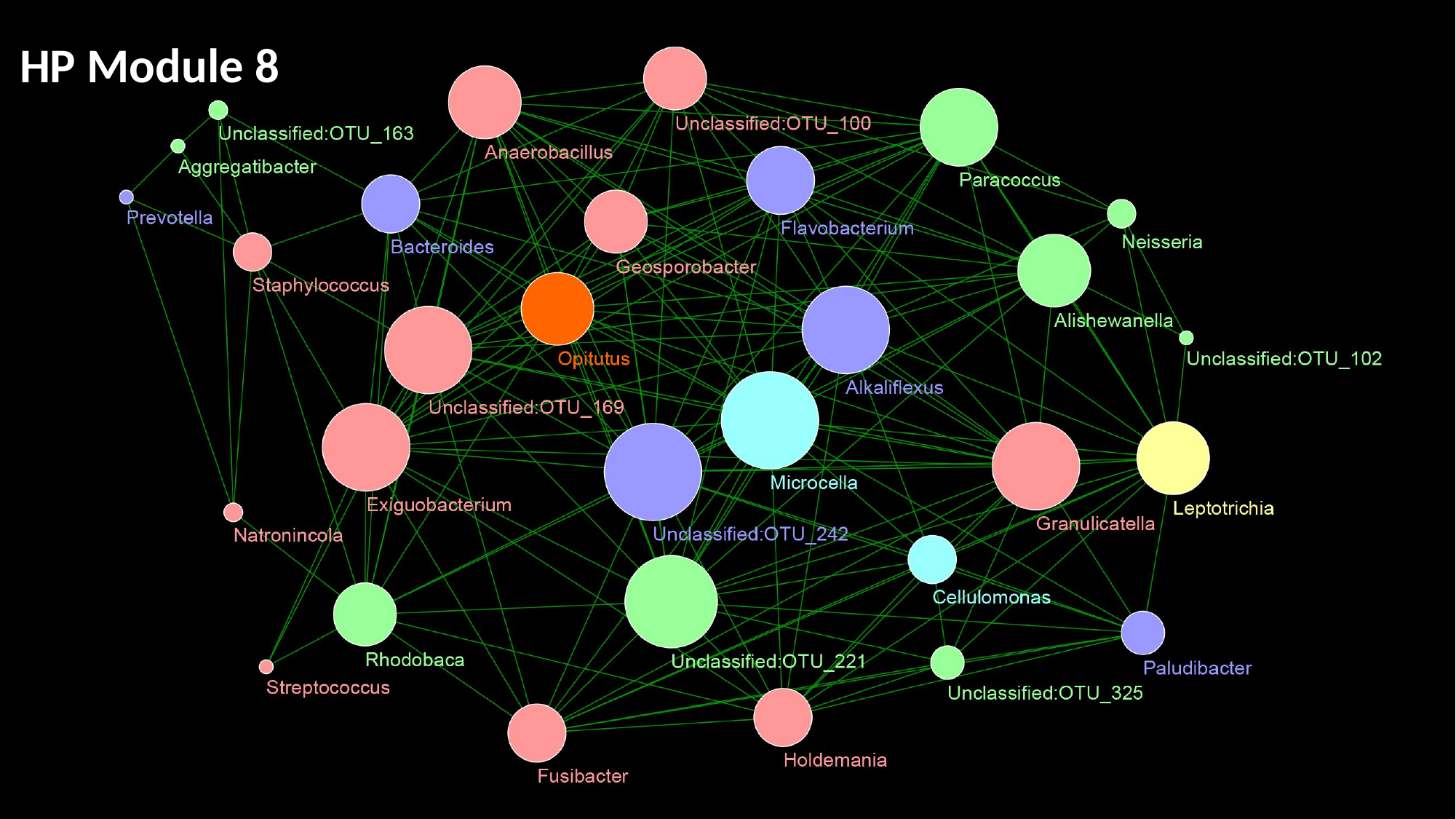

HP Module 8

## Slide 32
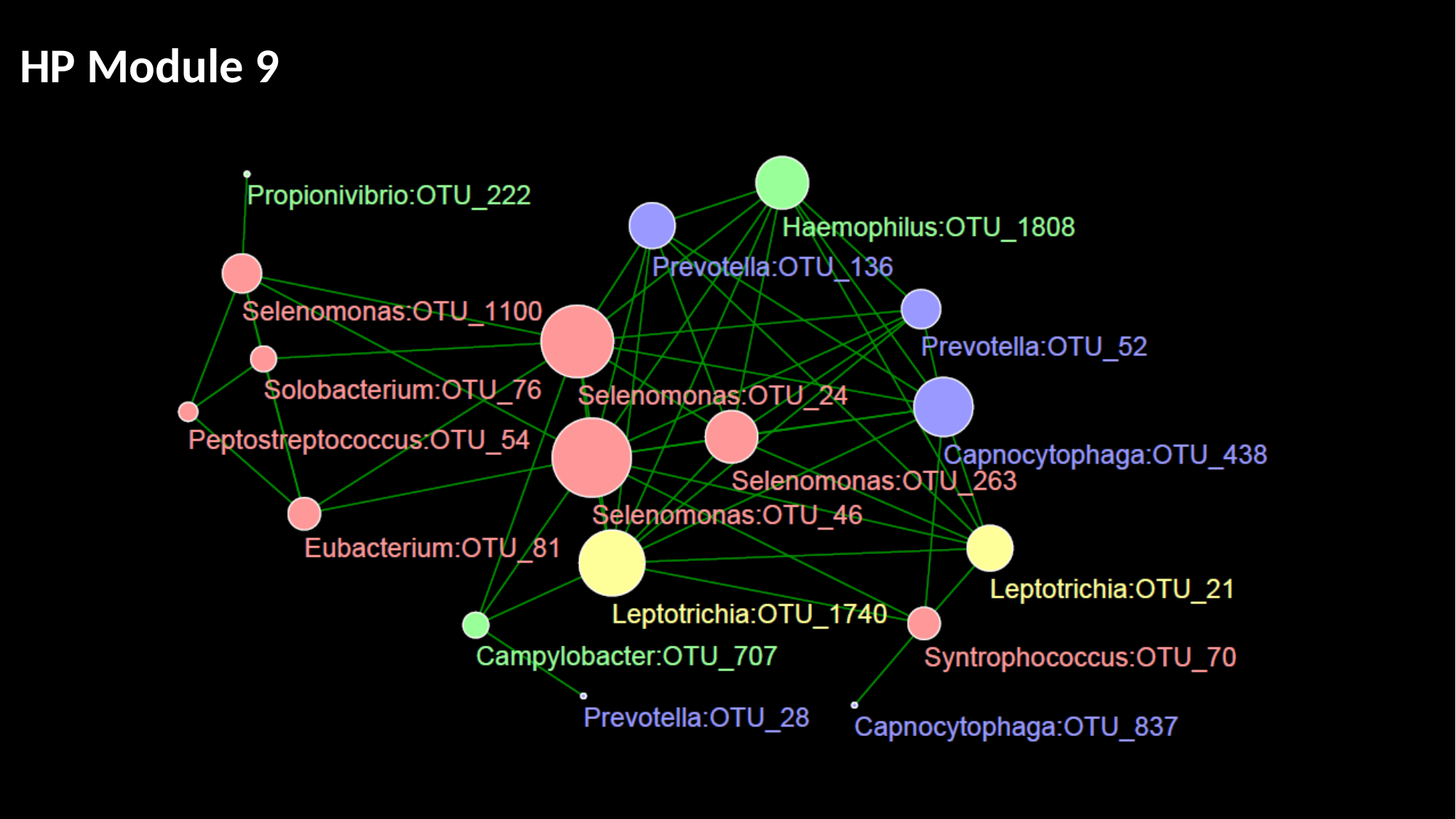

HP Module 9

## Slide 33
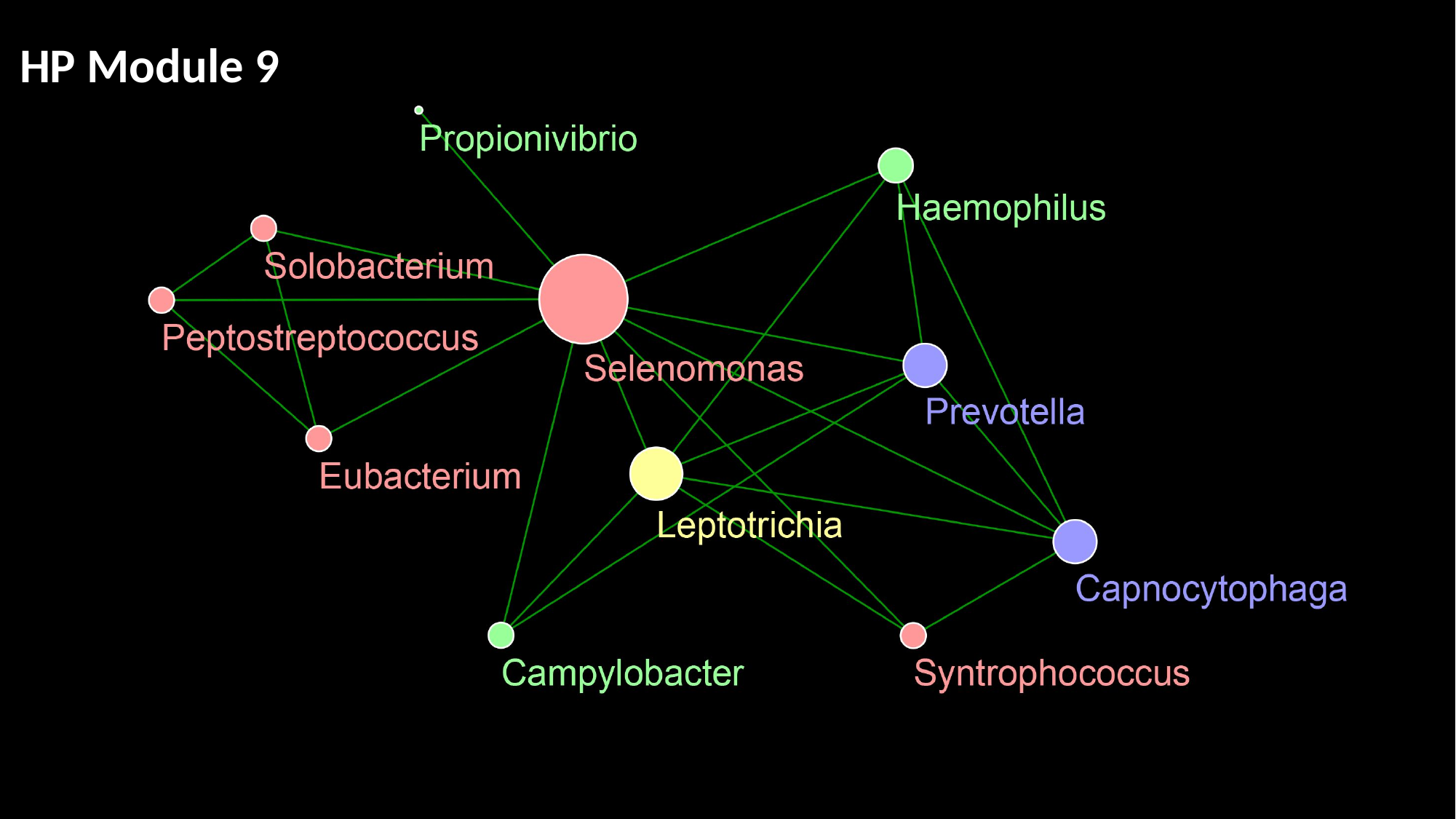

HP Module 9

## Slide 34
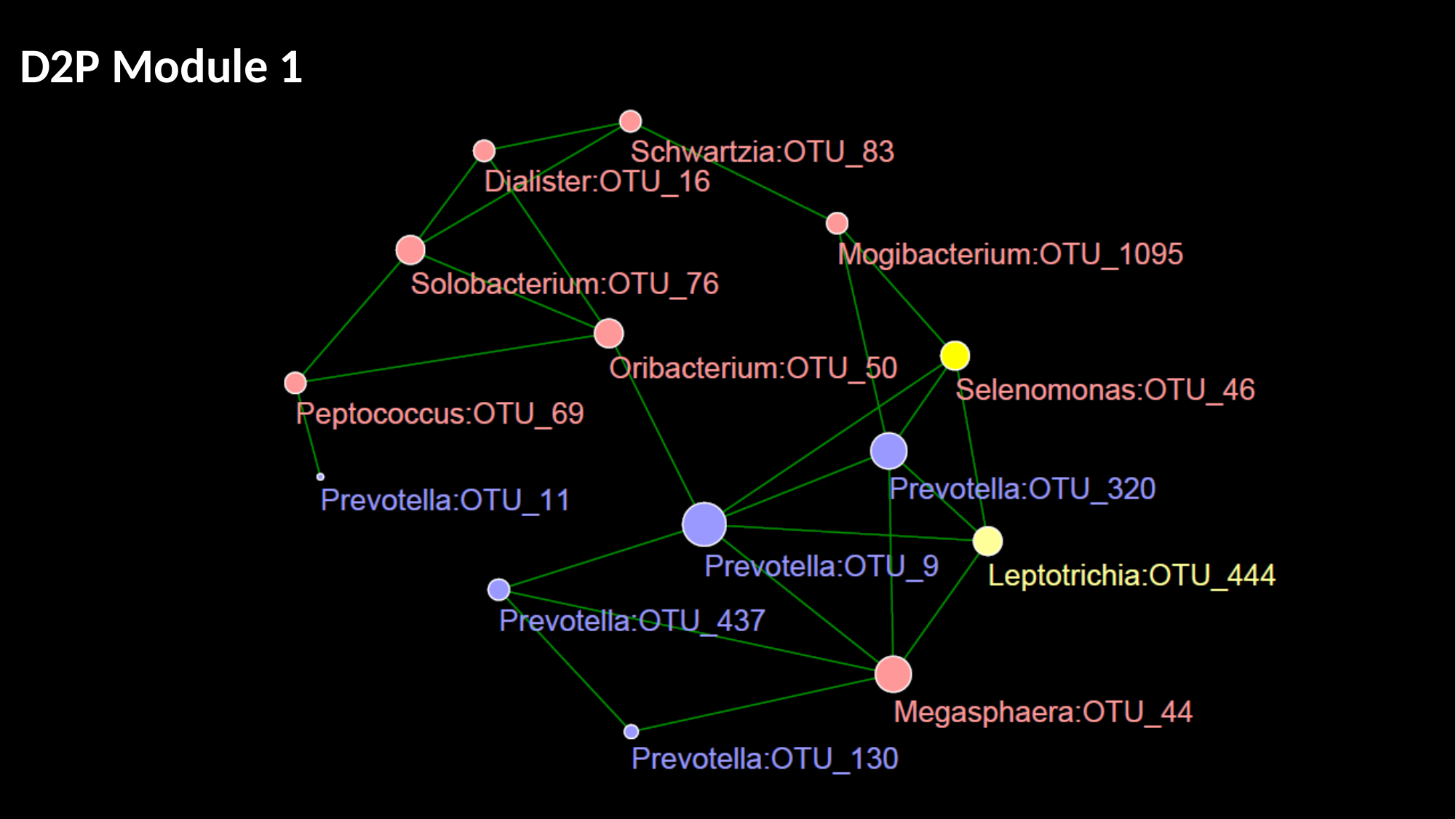

D2P Module 1

## Slide 35
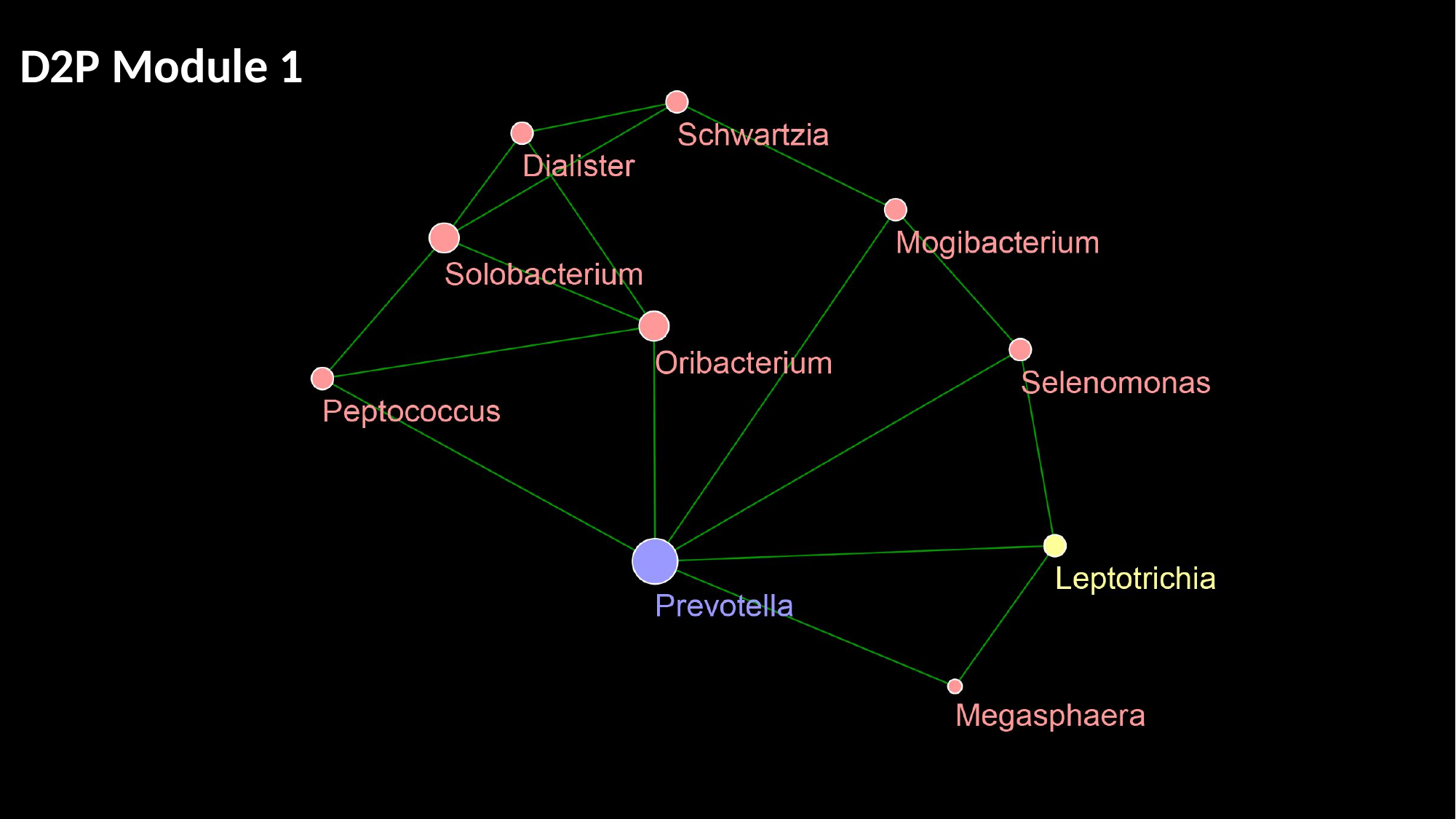

D2P Module 1

## Slide 36
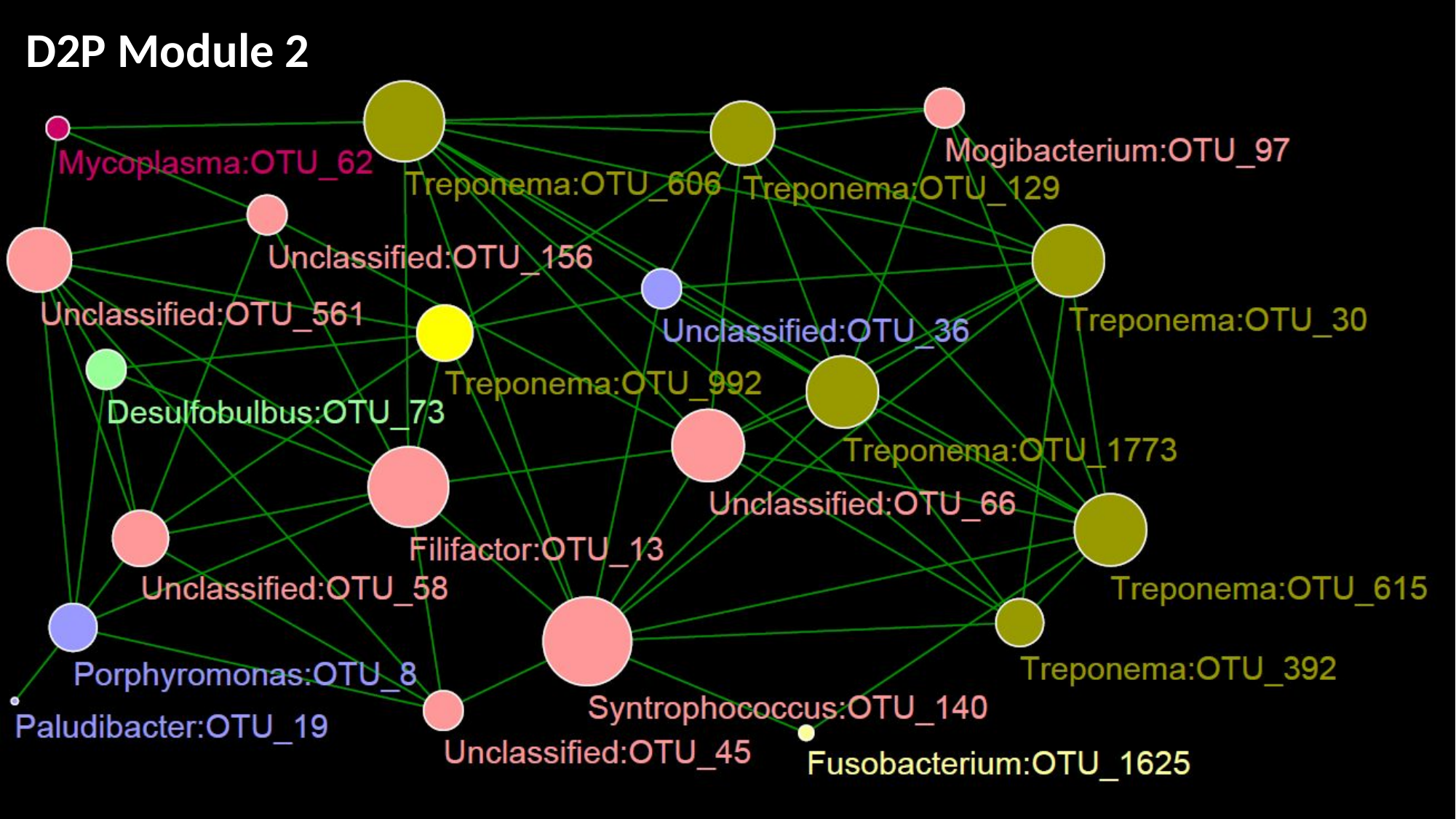

D2P Module 2

## Slide 37
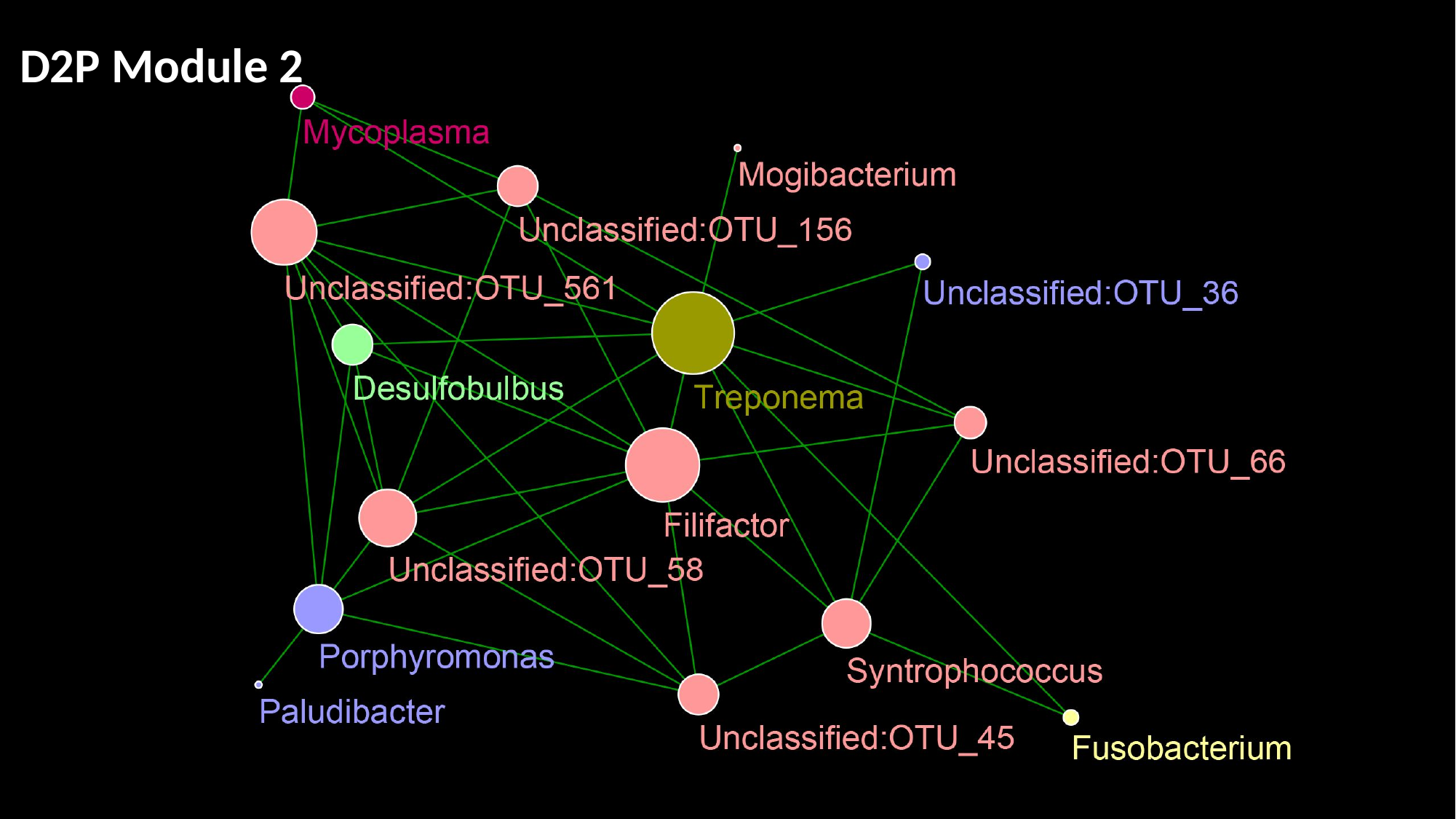

D2P Module 2

## Slide 38
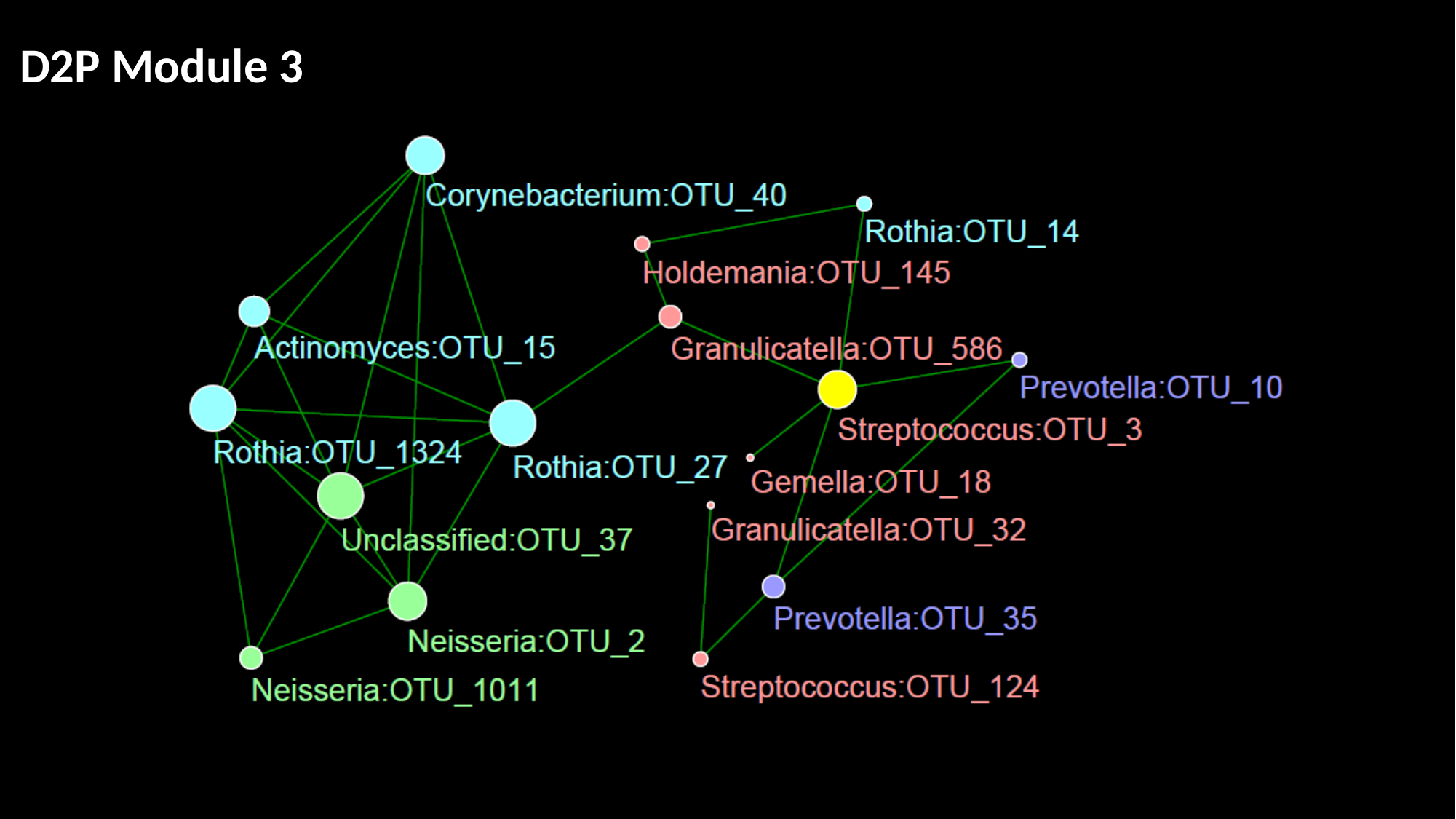

D2P Module 3

## Slide 39
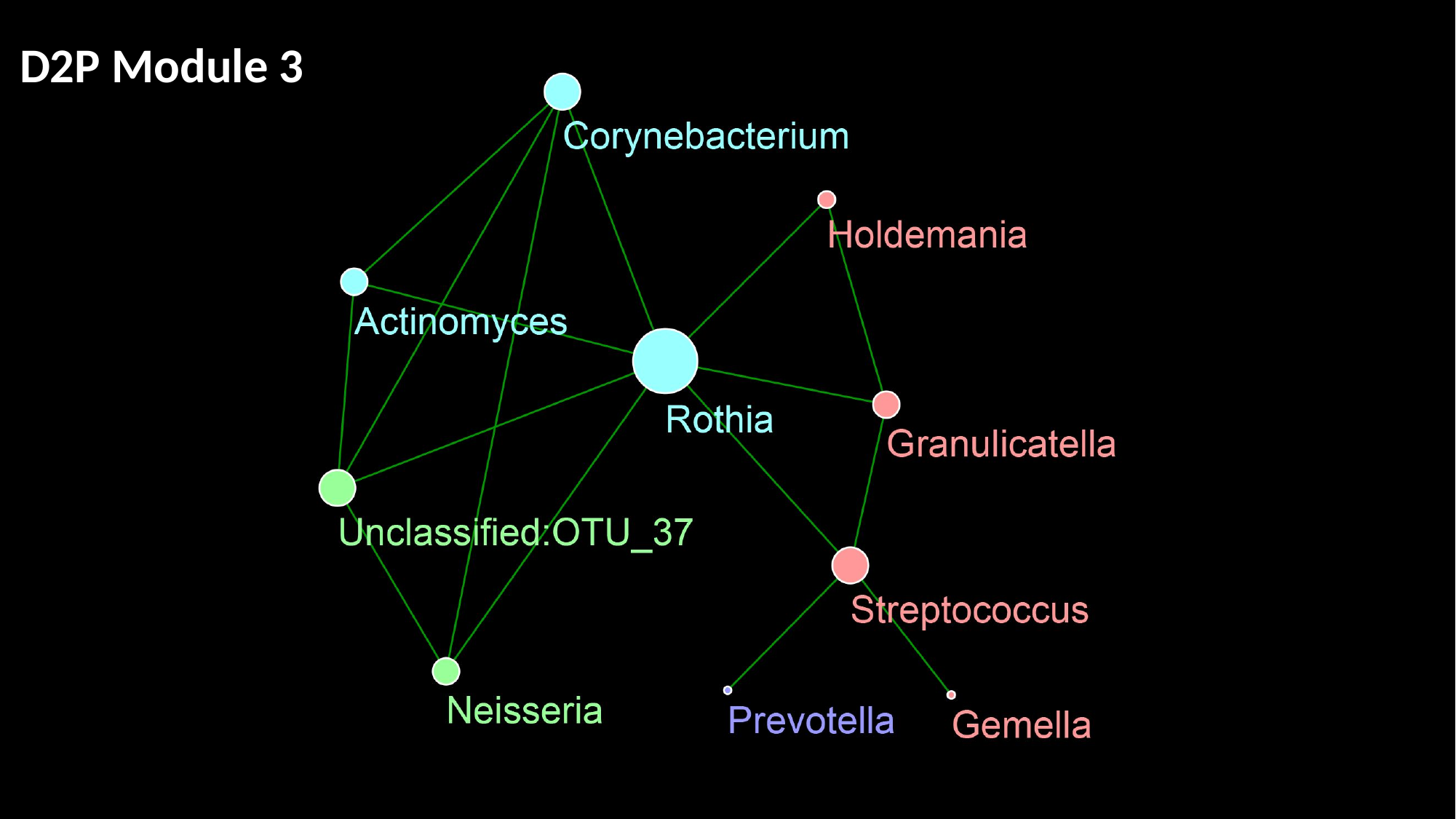

D2P Module 3

## Slide 40
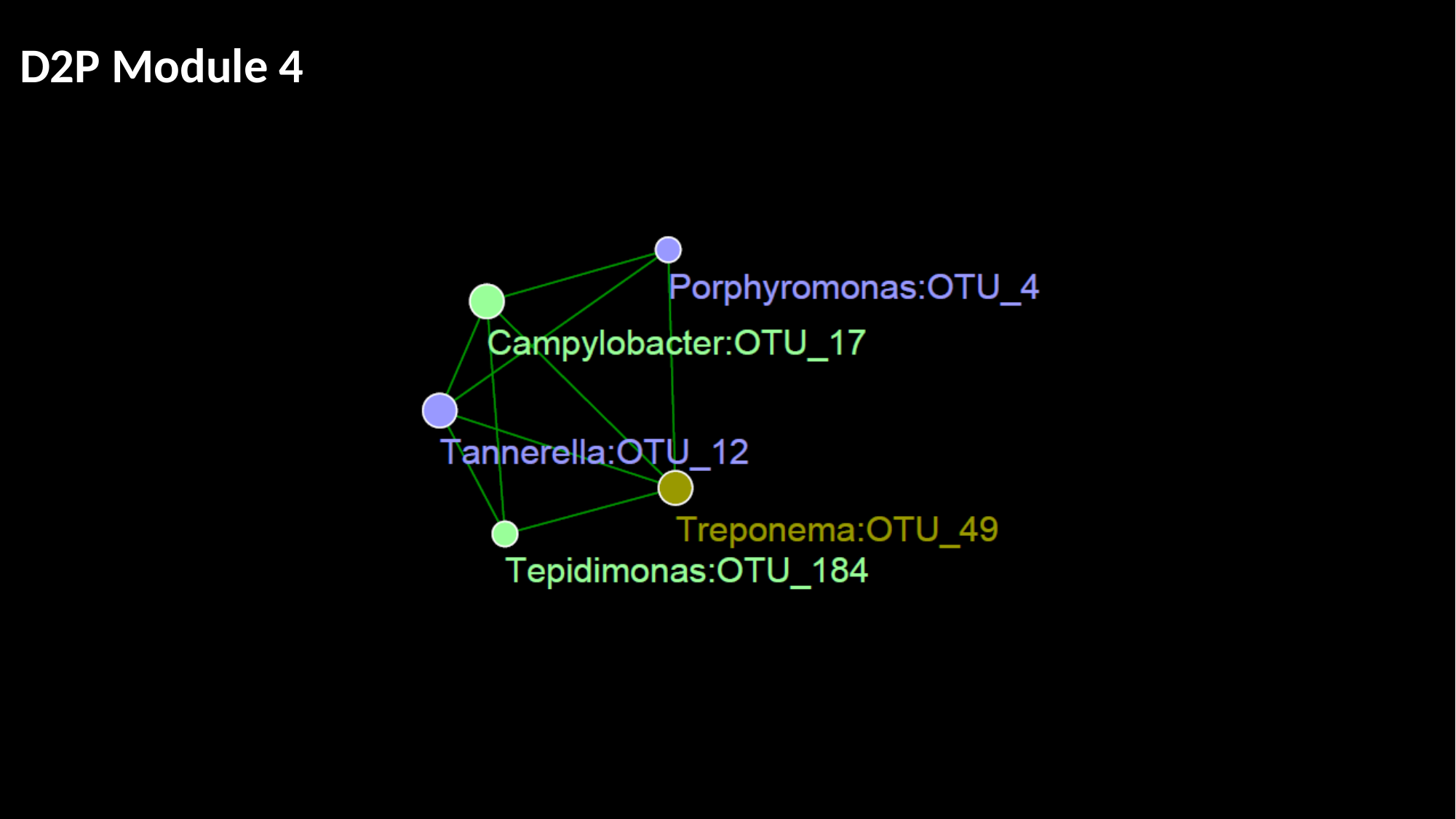

D2P Module 4

## Slide 41
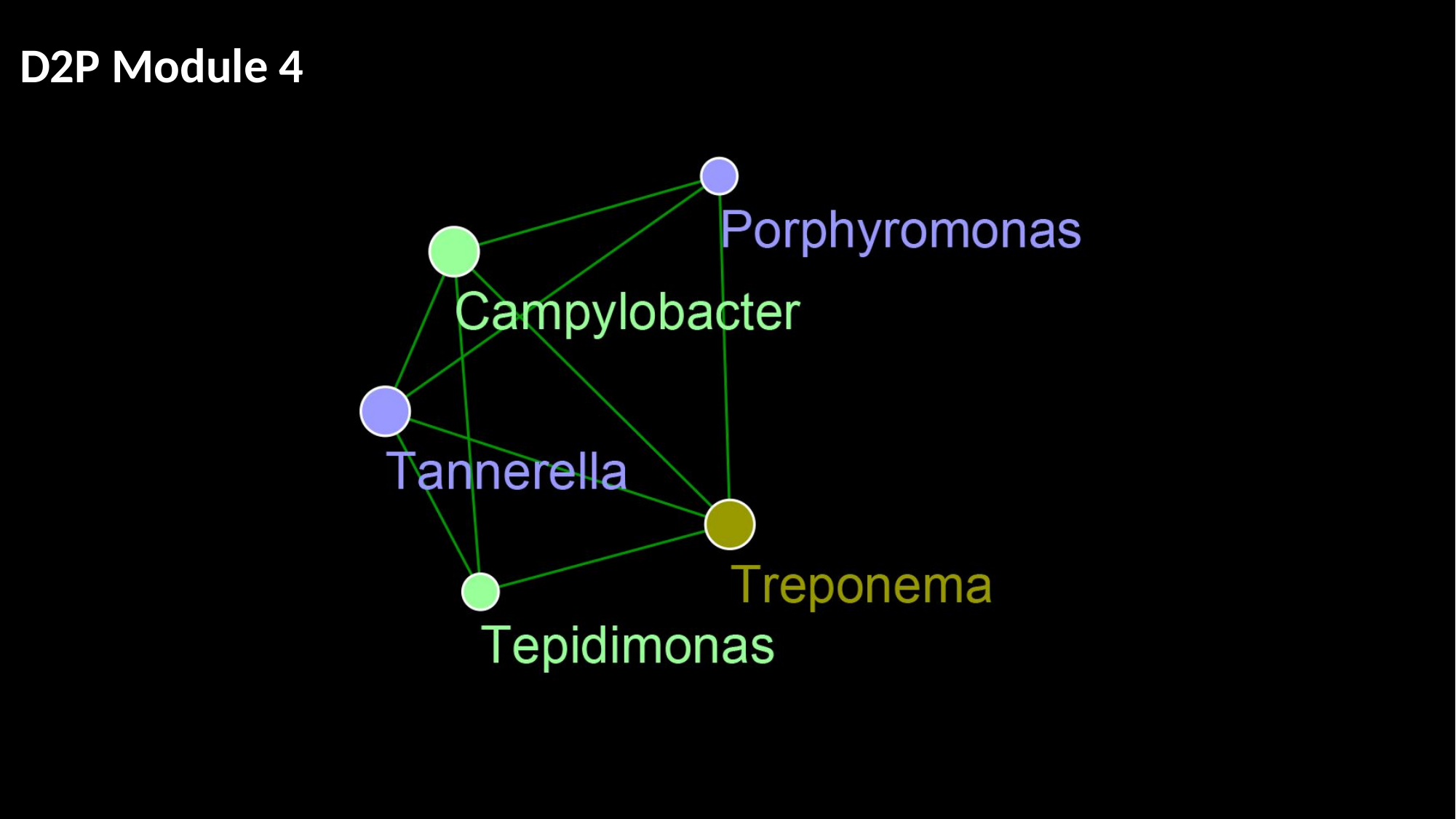

D2P Module 4

## Slide 42
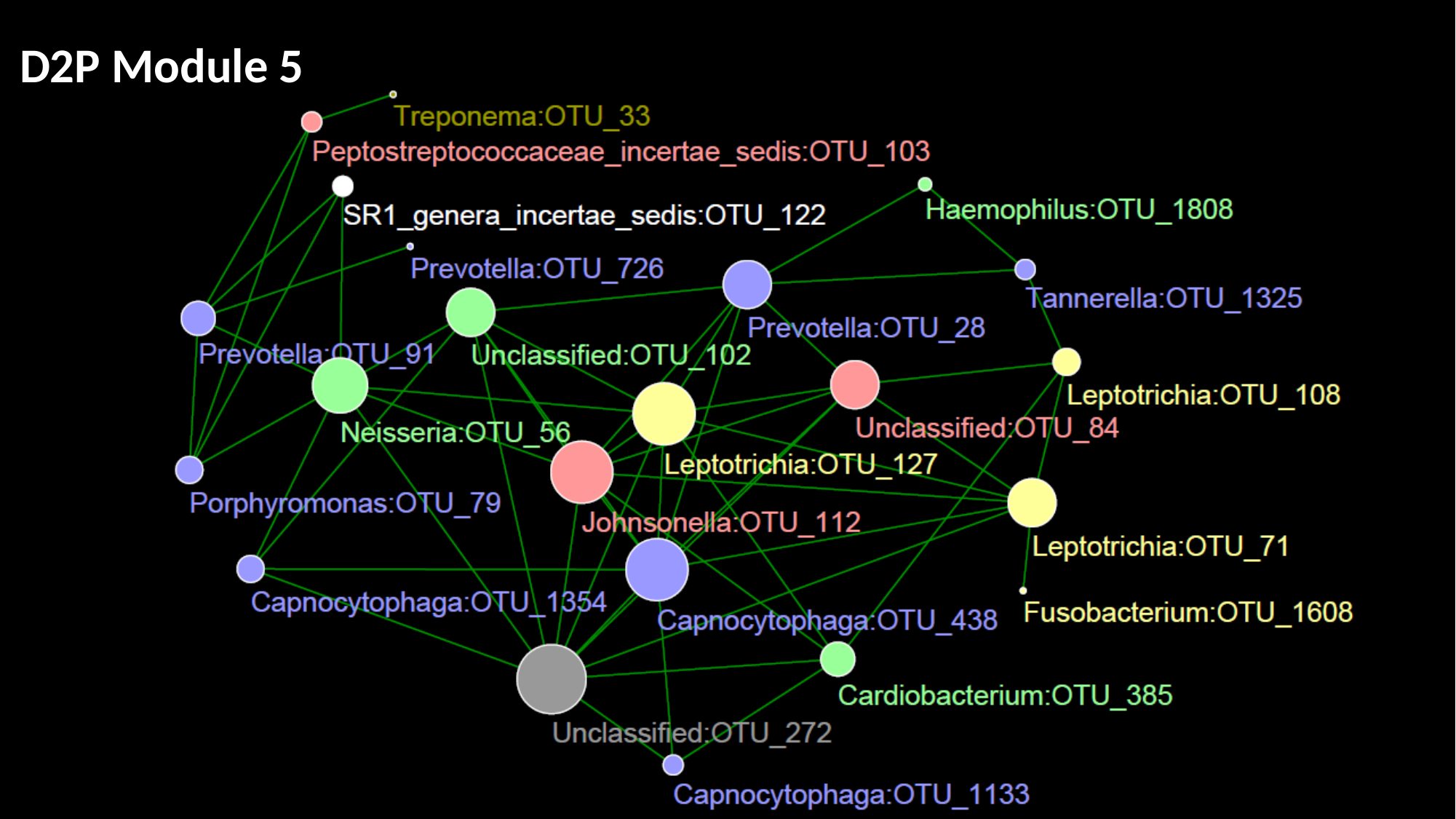

D2P Module 5

## Slide 43
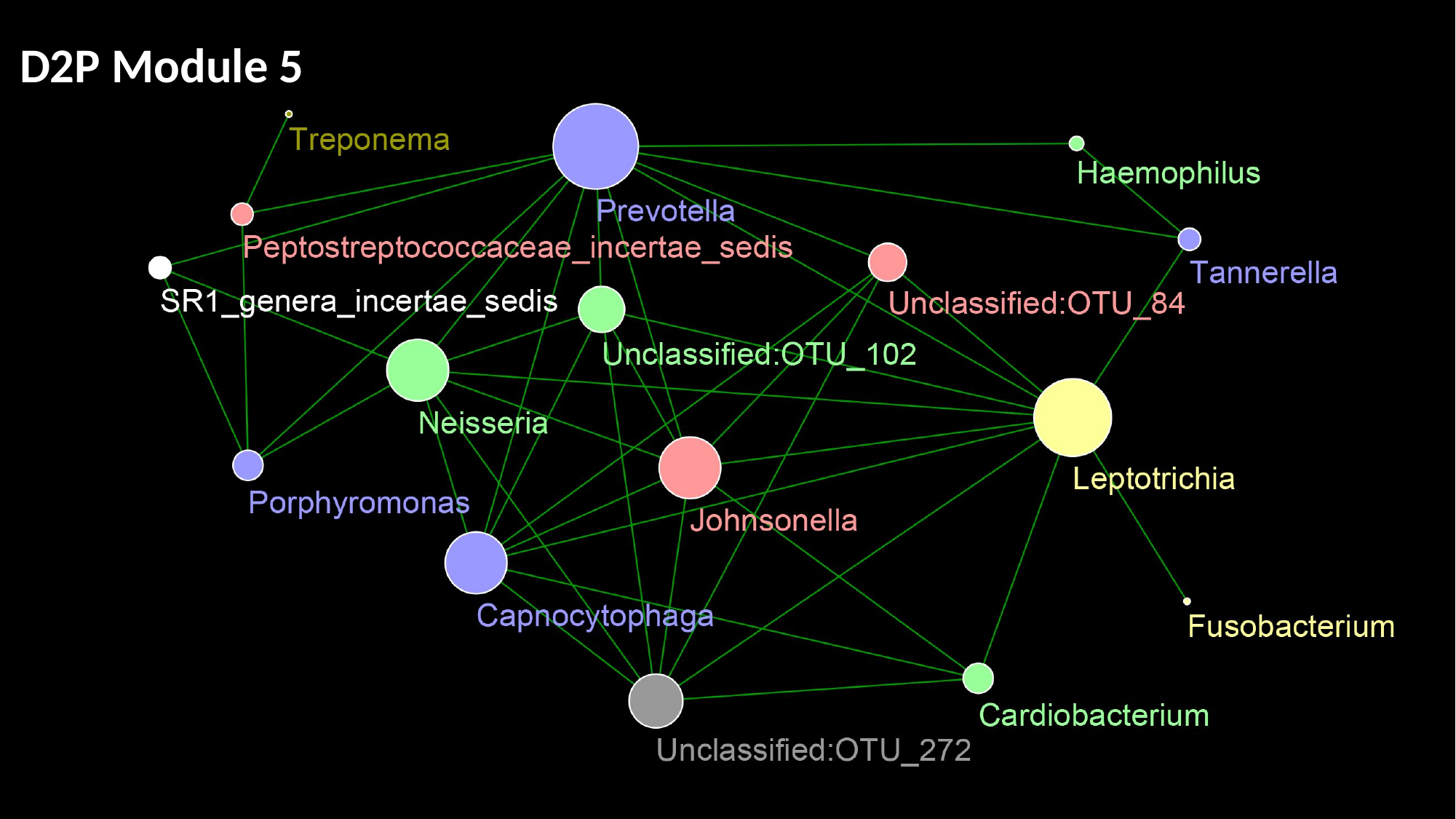

D2P Module 5

## Slide 44
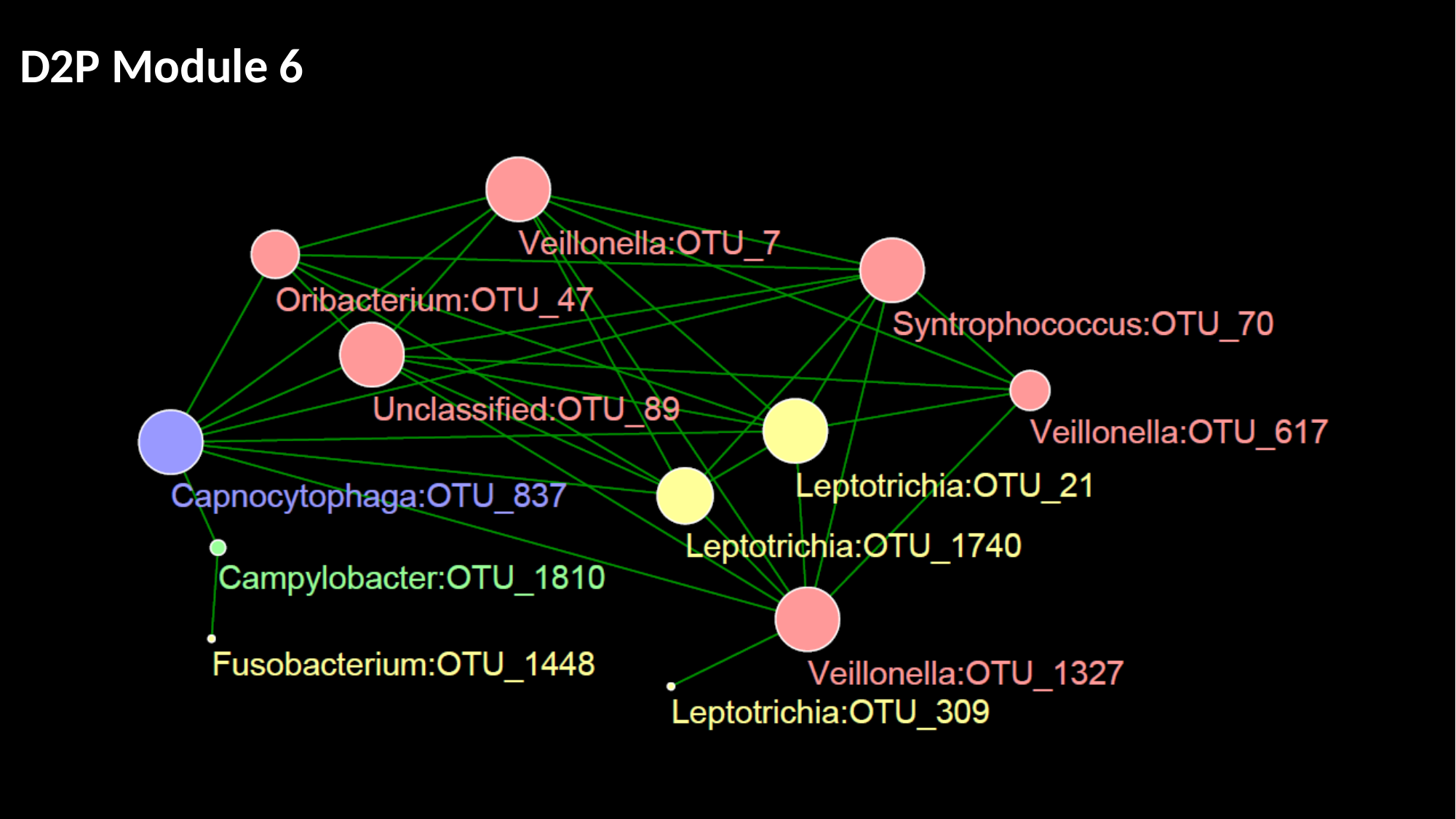

D2P Module 6

## Slide 45
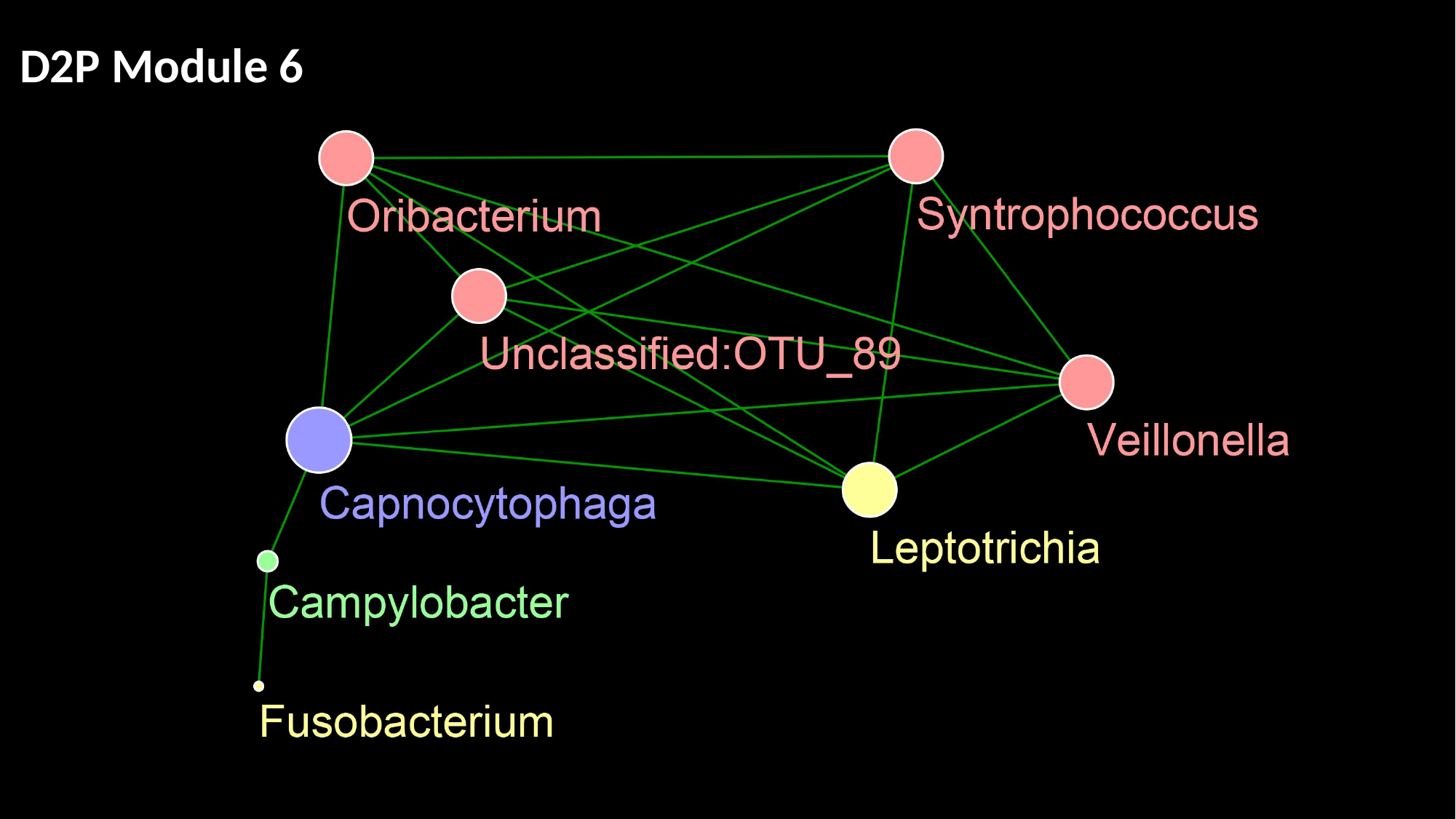

D2P Module 6

## Slide 46
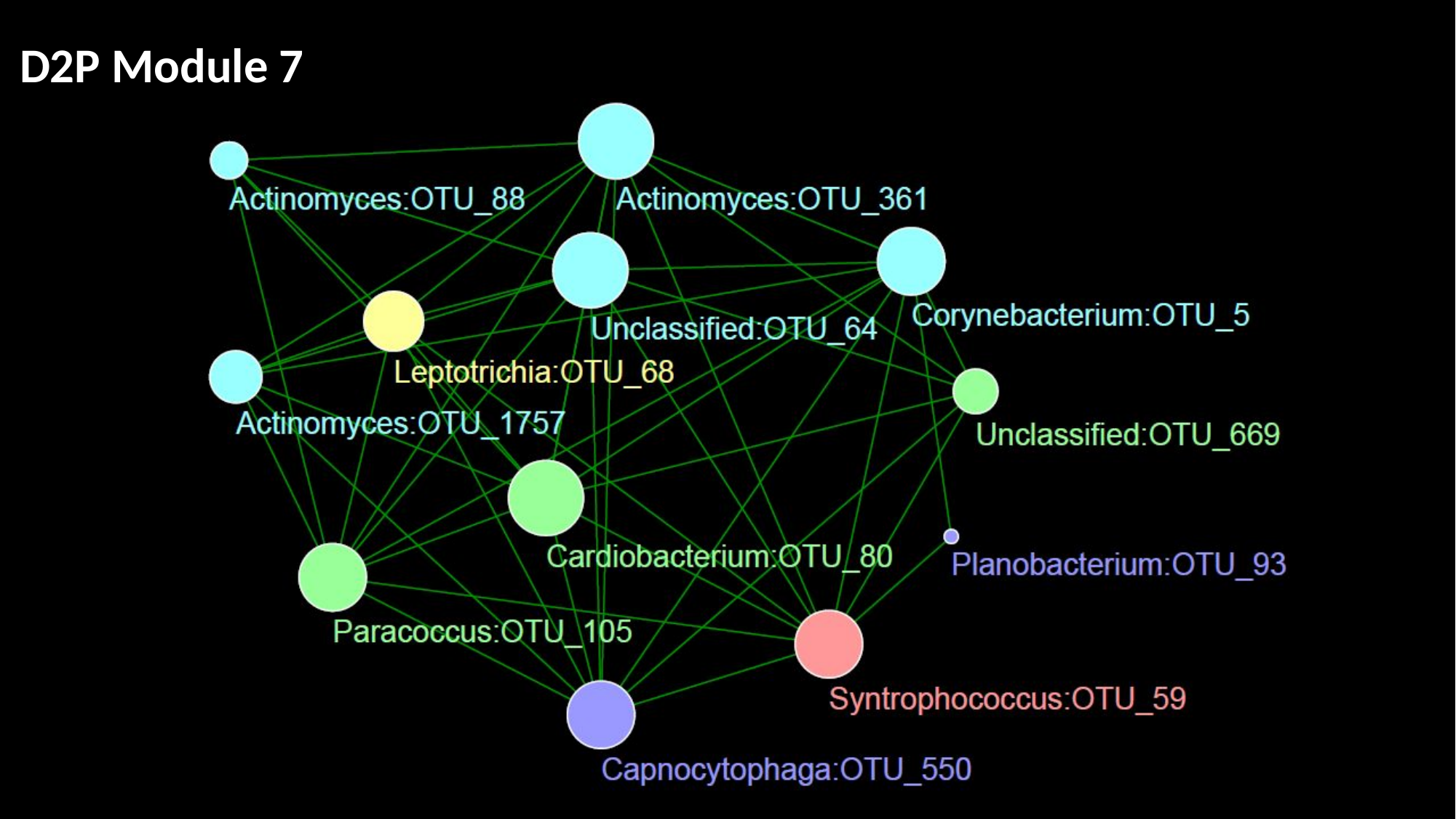

D2P Module 7

## Slide 47
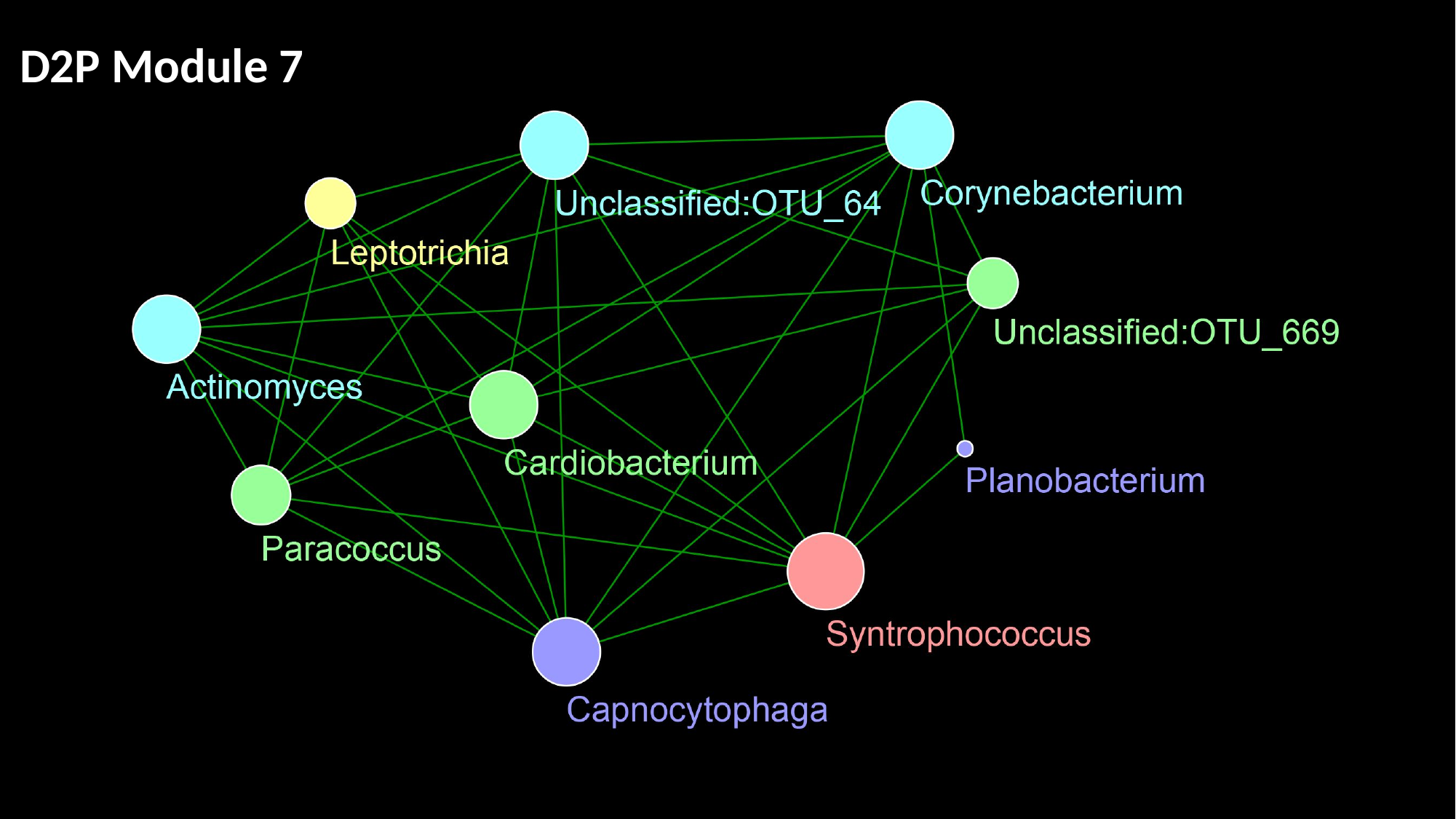

D2P Module 7

## Slide 48
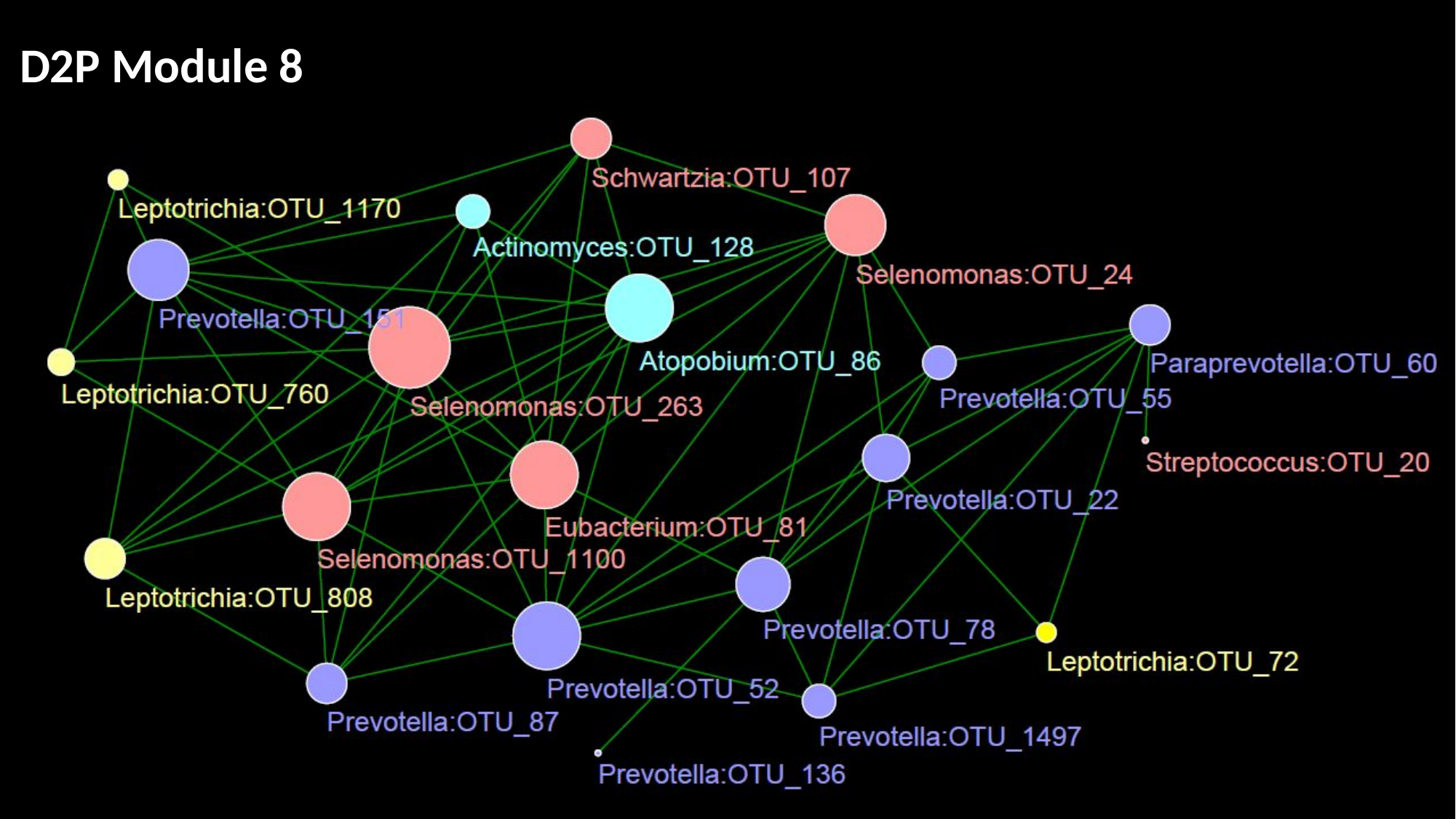

D2P Module 8

## Slide 49
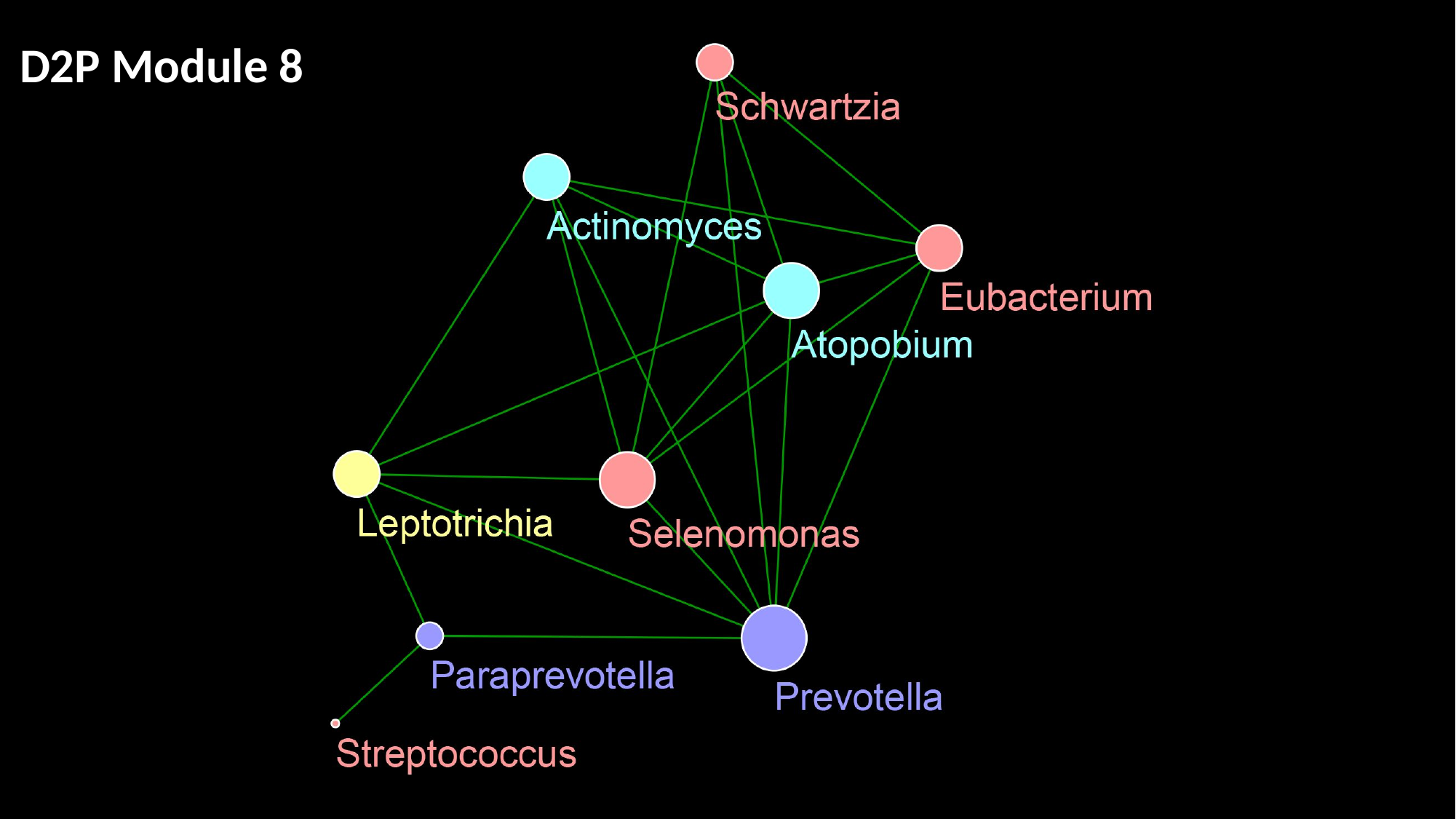

D2P Module 8

## Slide 50
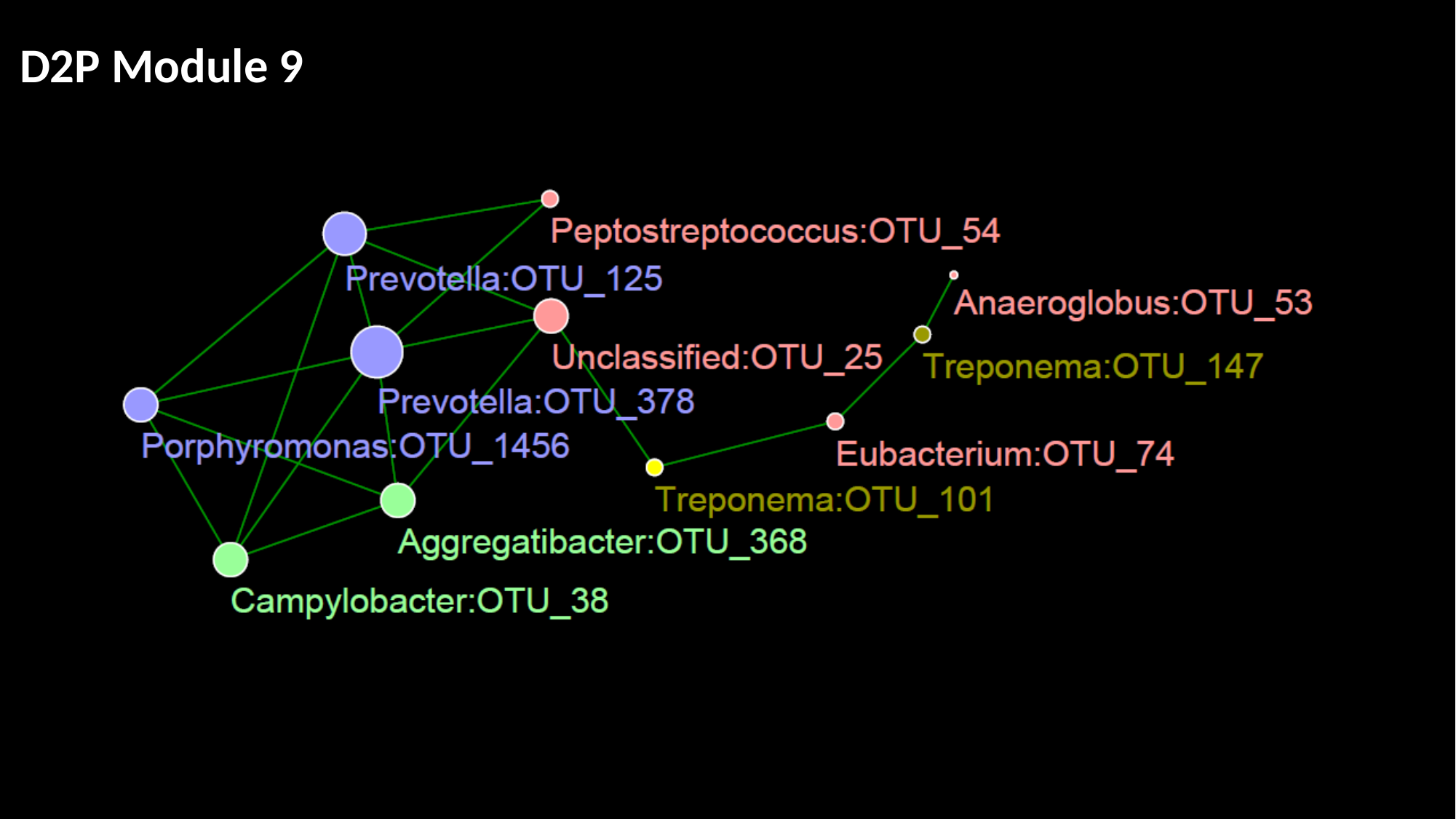

D2P Module 9

## Slide 51
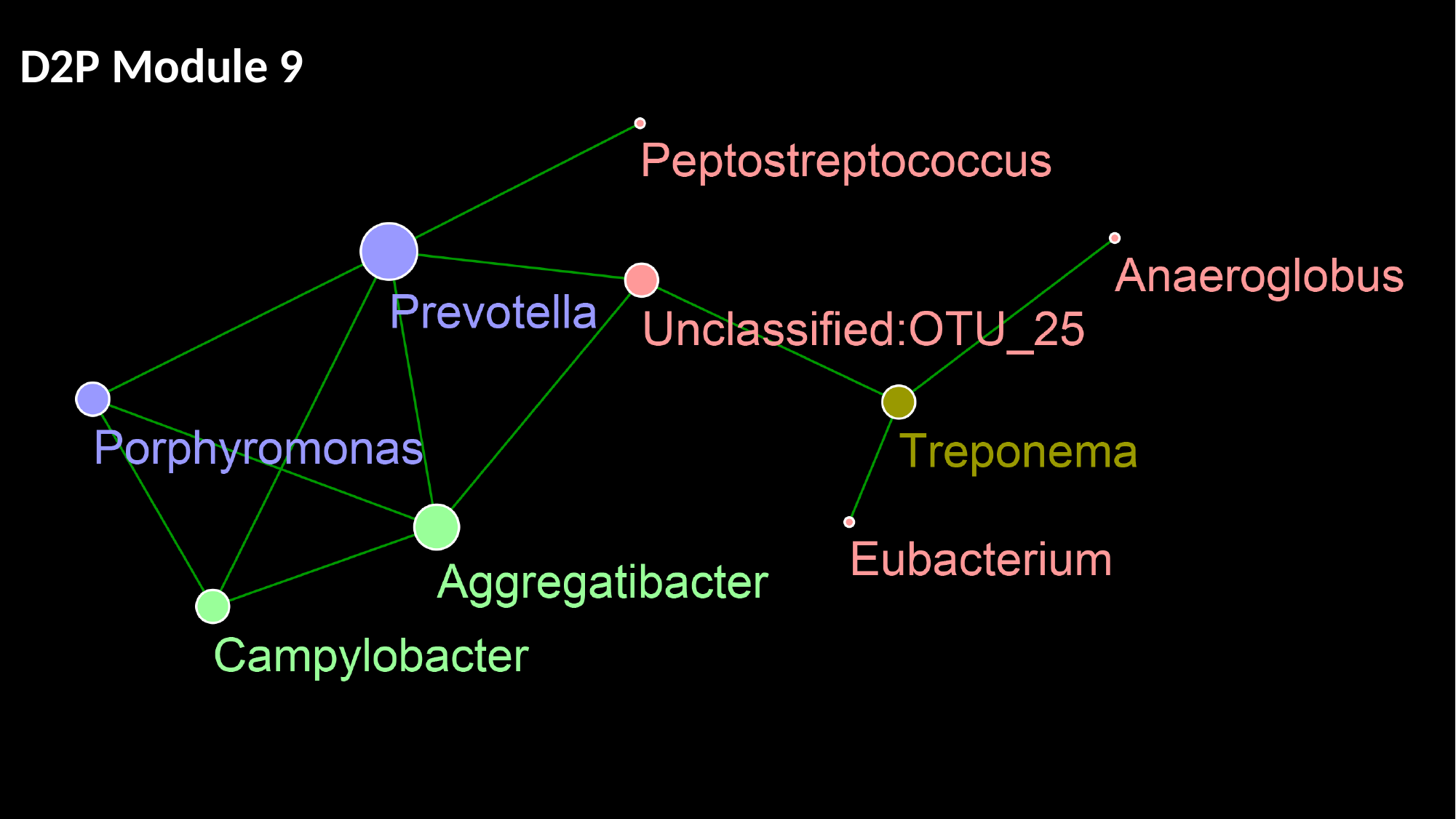

D2P Module 9

## Slide 52
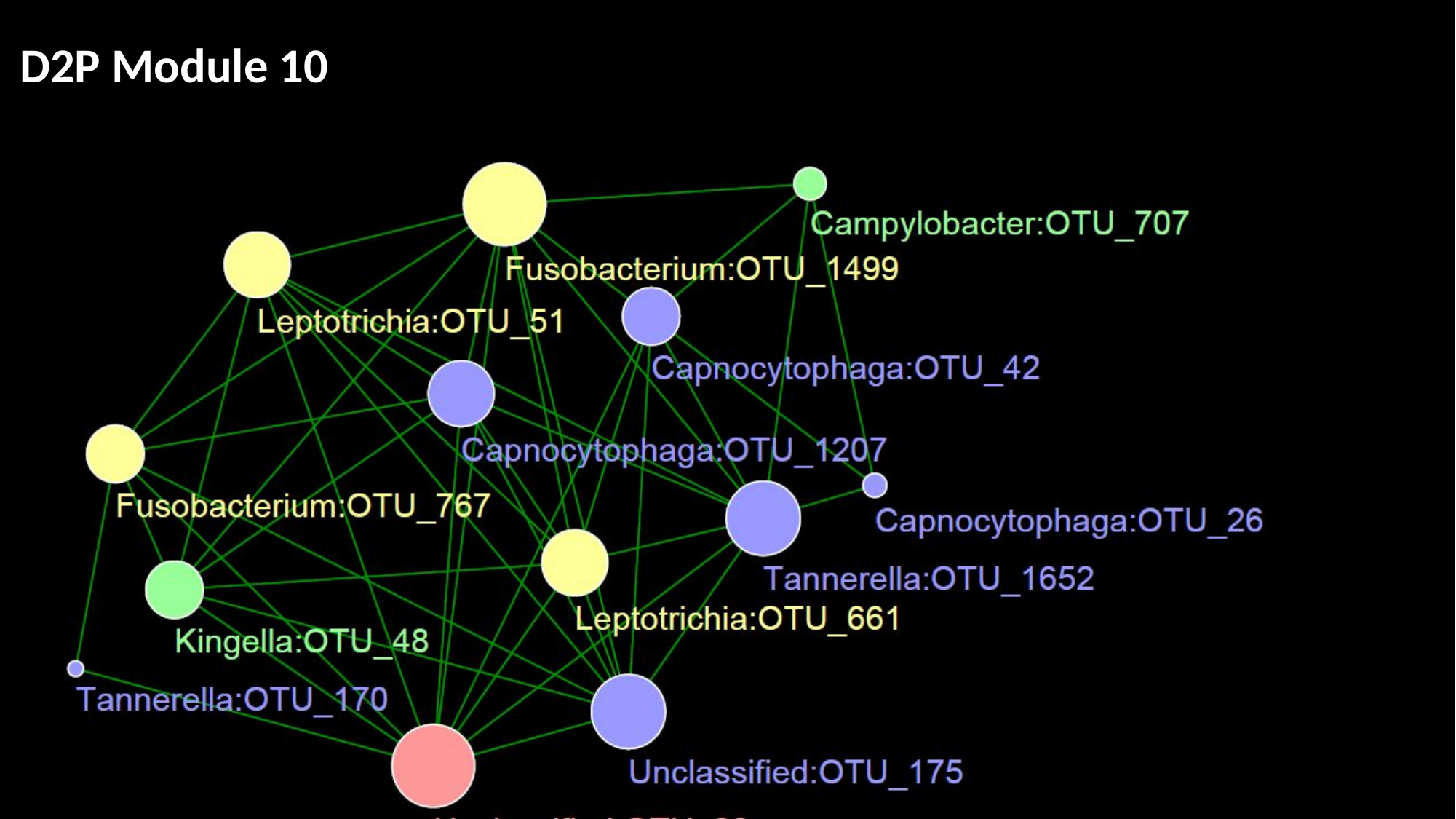

D2P Module 10

## Slide 53
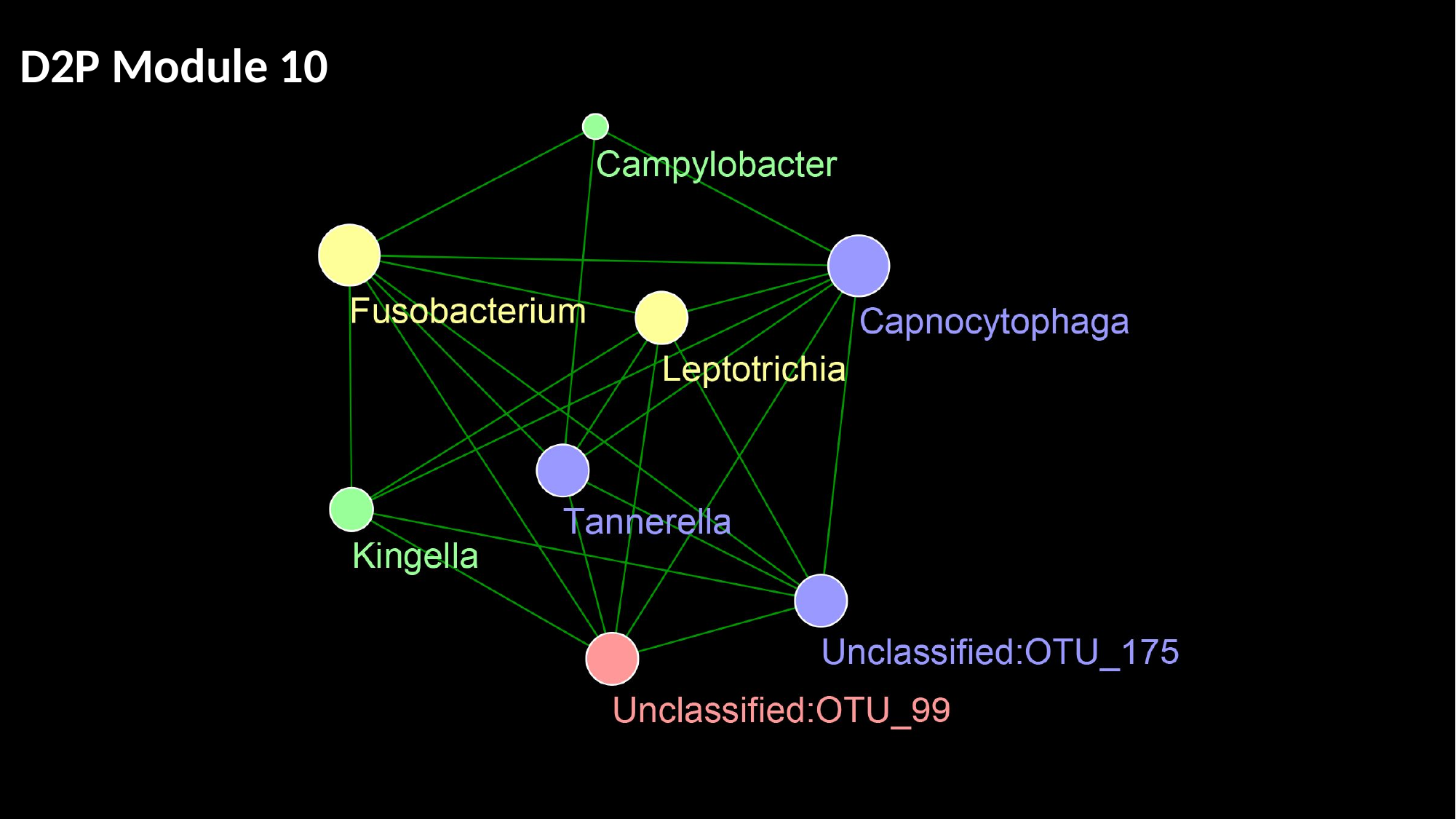

D2P Module 10

## Slide 54
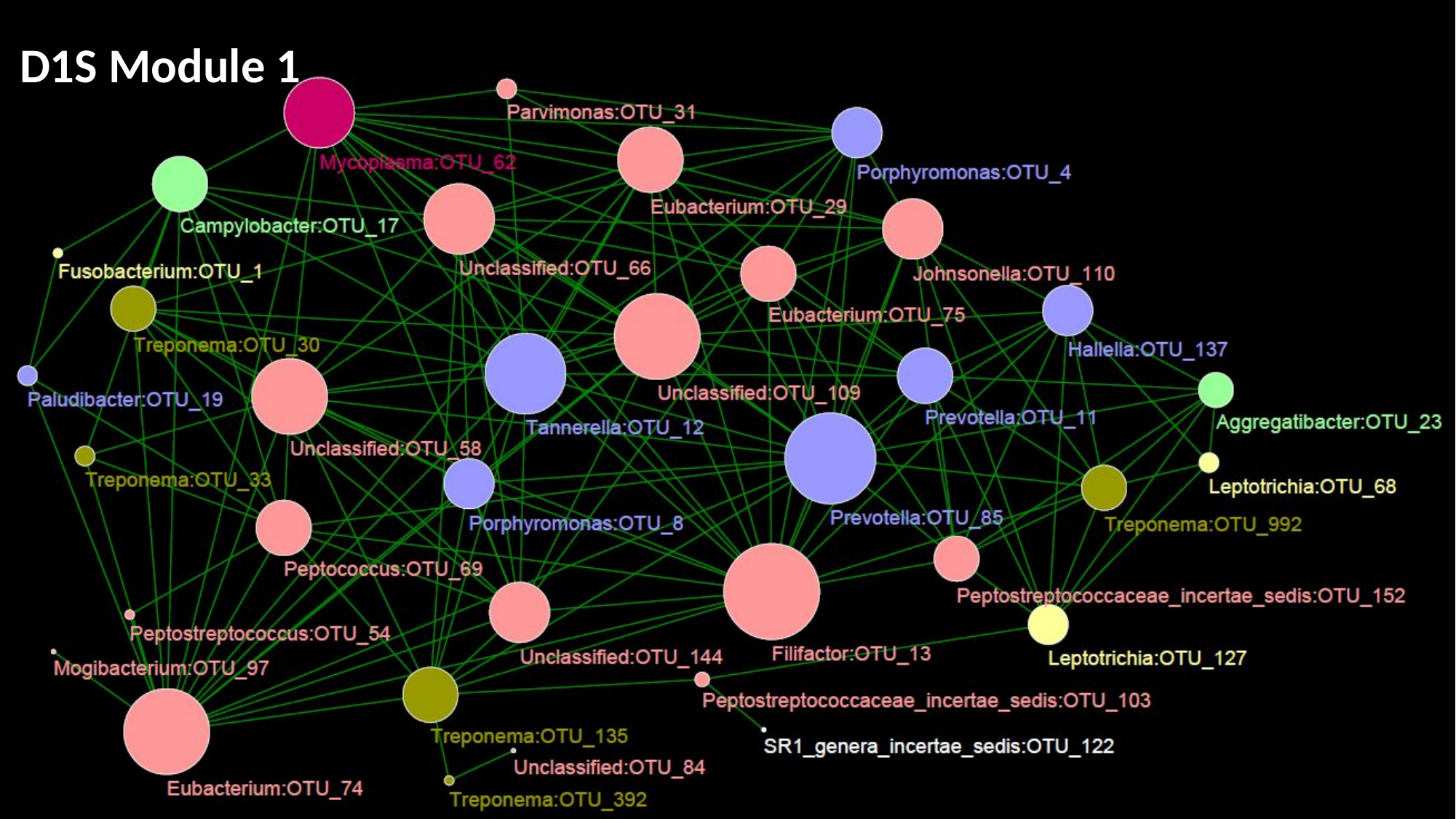

D1S Module 1

## Slide 55
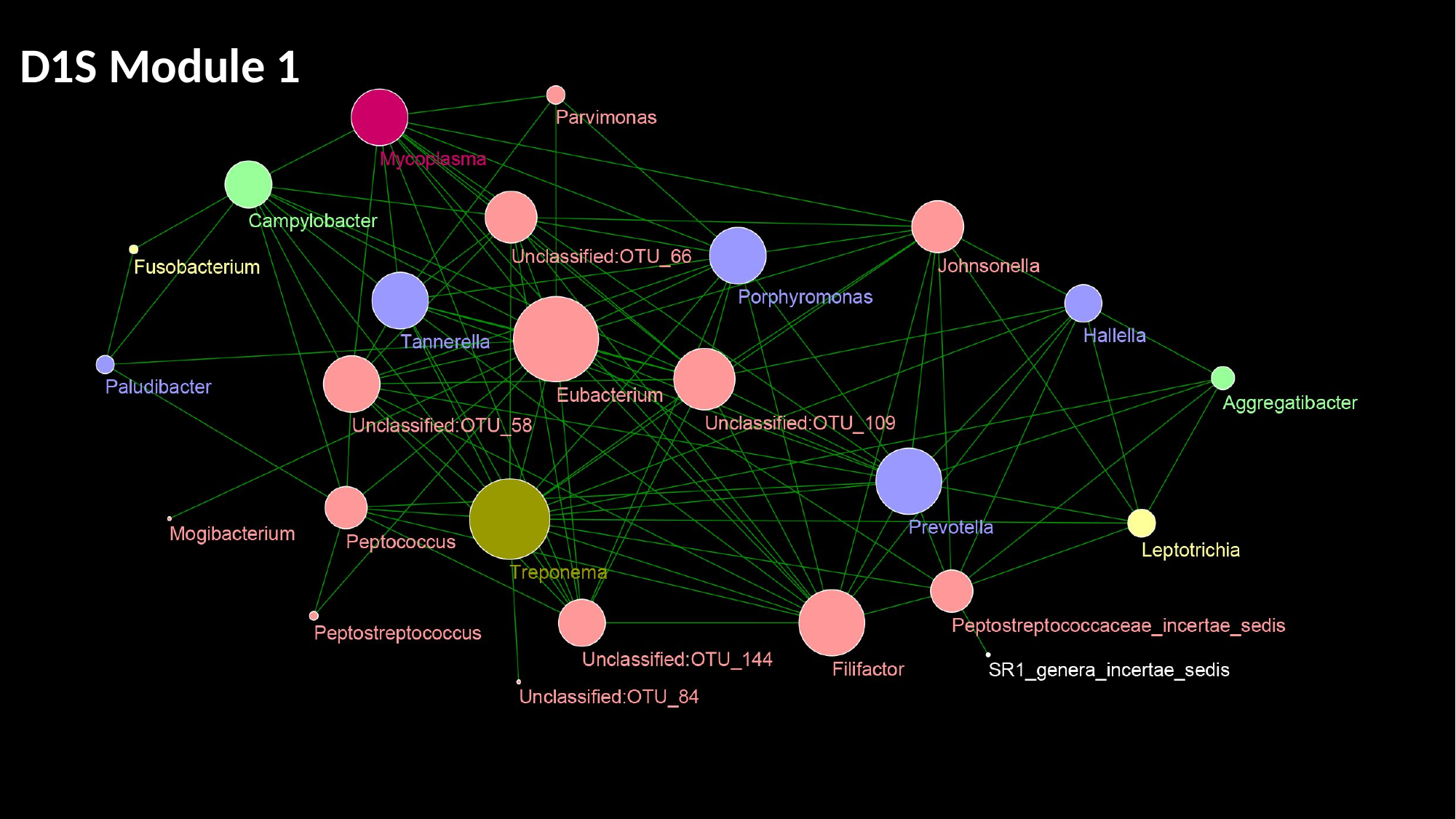

D1S Module 1

## Slide 56
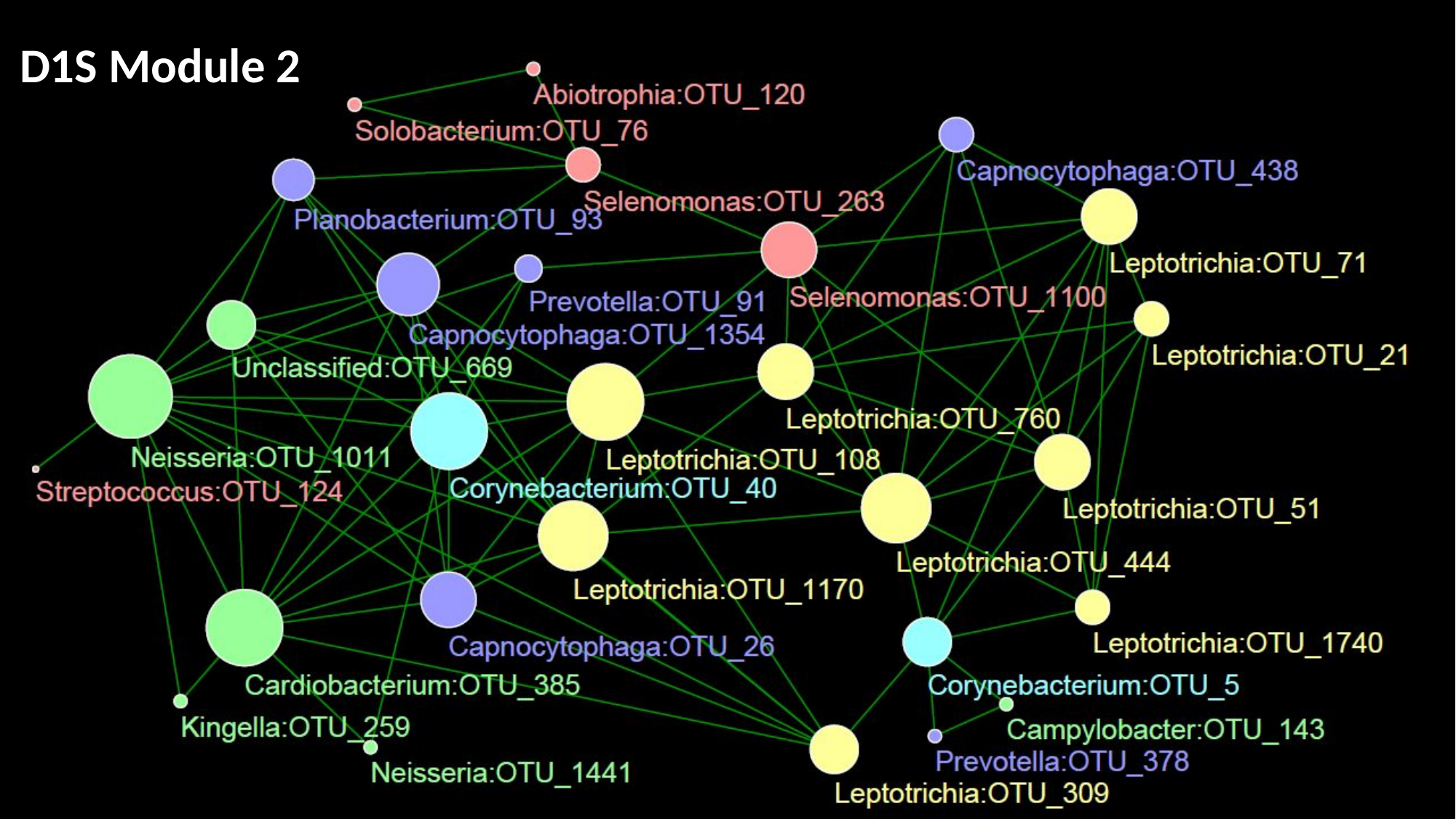

D1S Module 2

## Slide 57
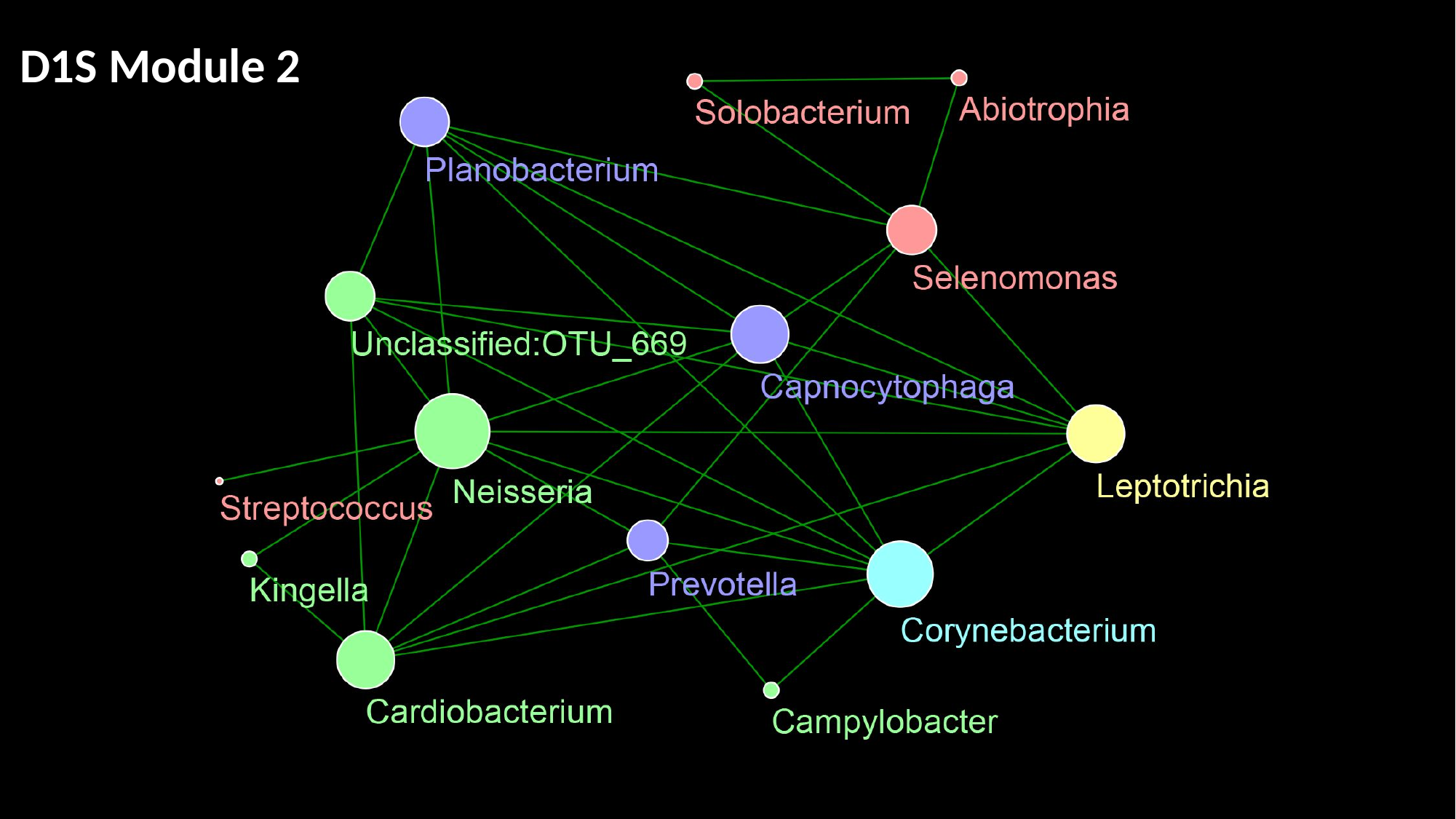

D1S Module 2

## Slide 58
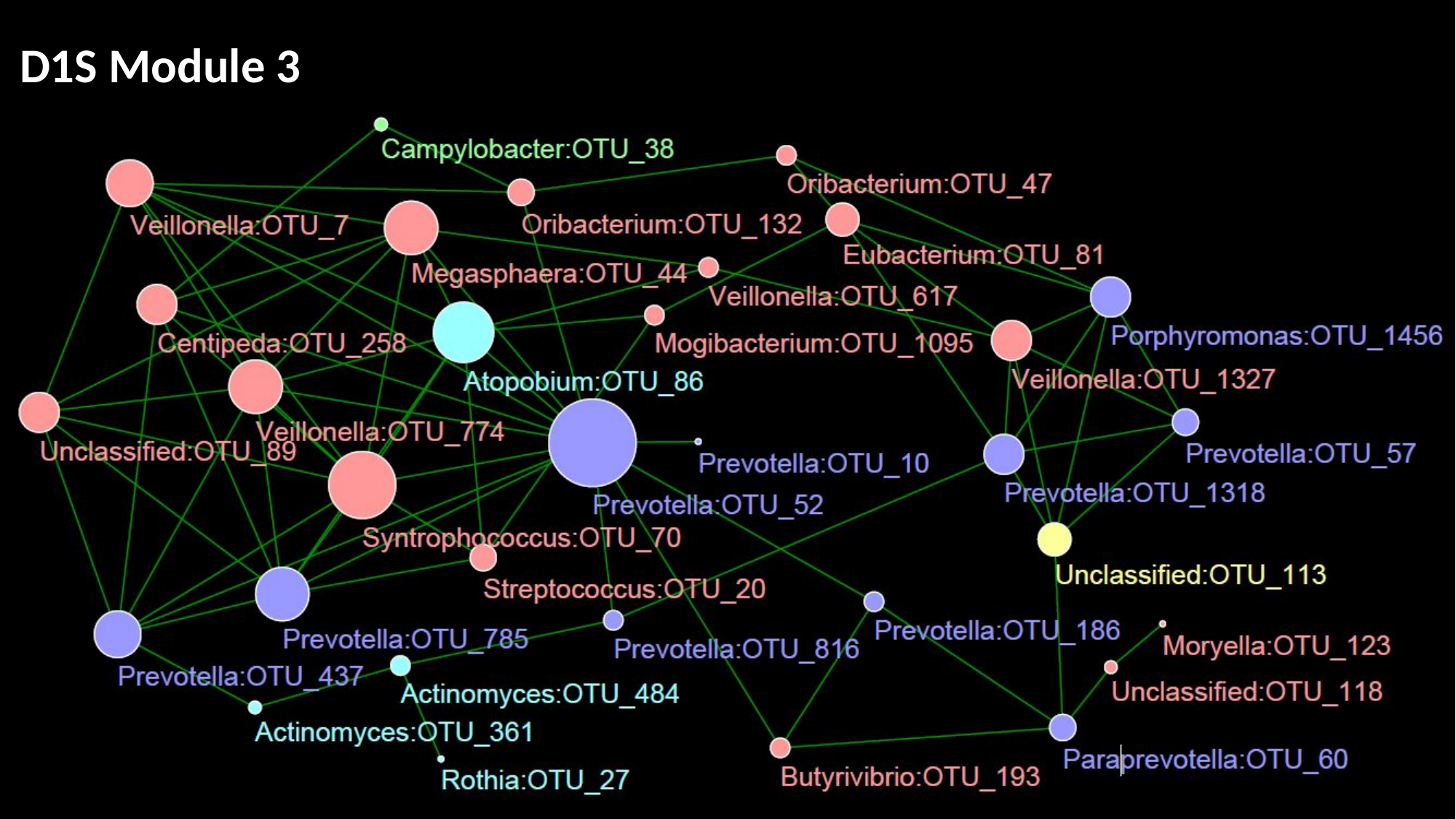

D1S Module 3

## Slide 59
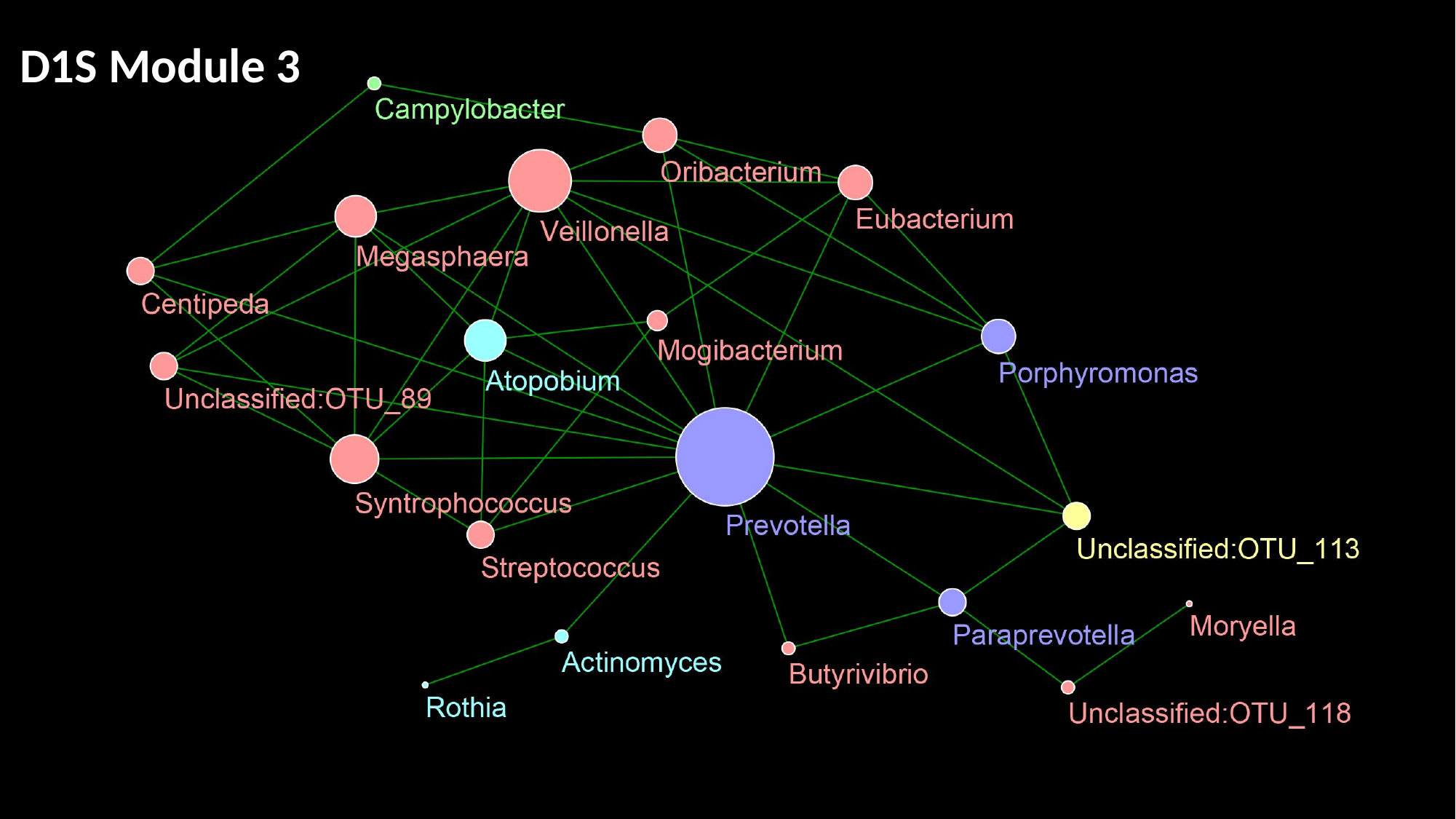

D1S Module 3

## Slide 60
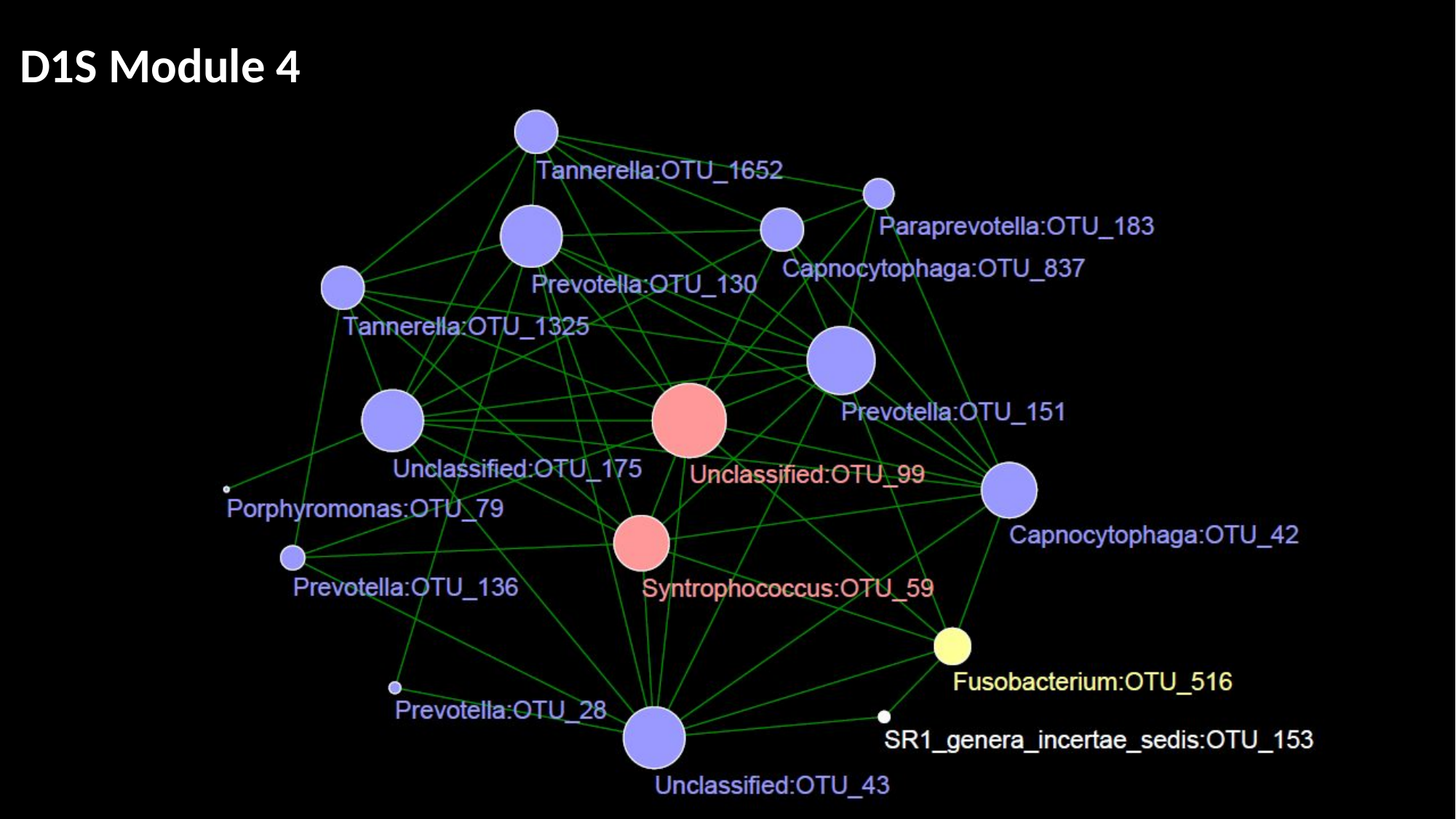

D1S Module 4

## Slide 61
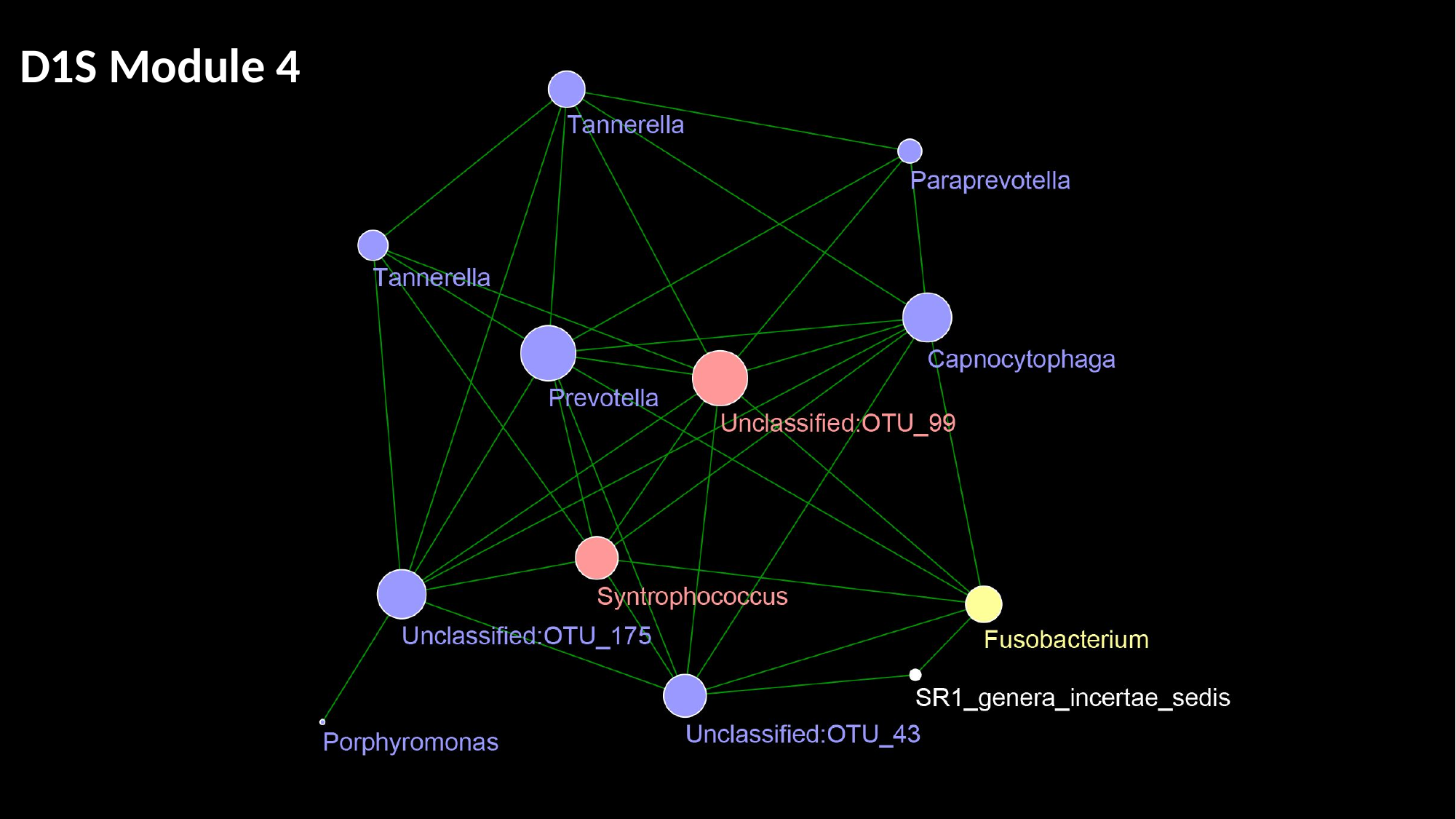

D1S Module 4

## Slide 62
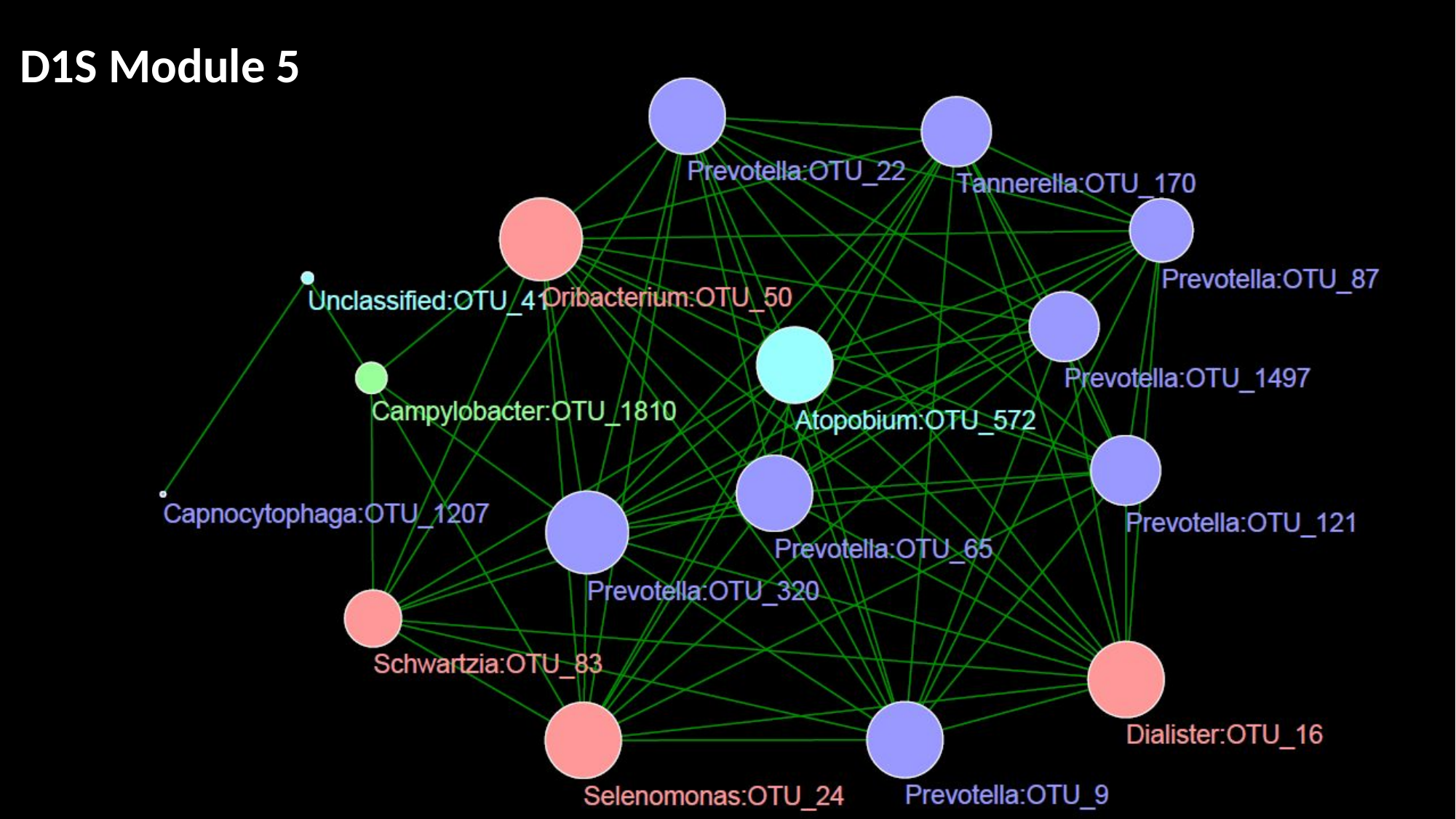

D1S Module 5

## Slide 63
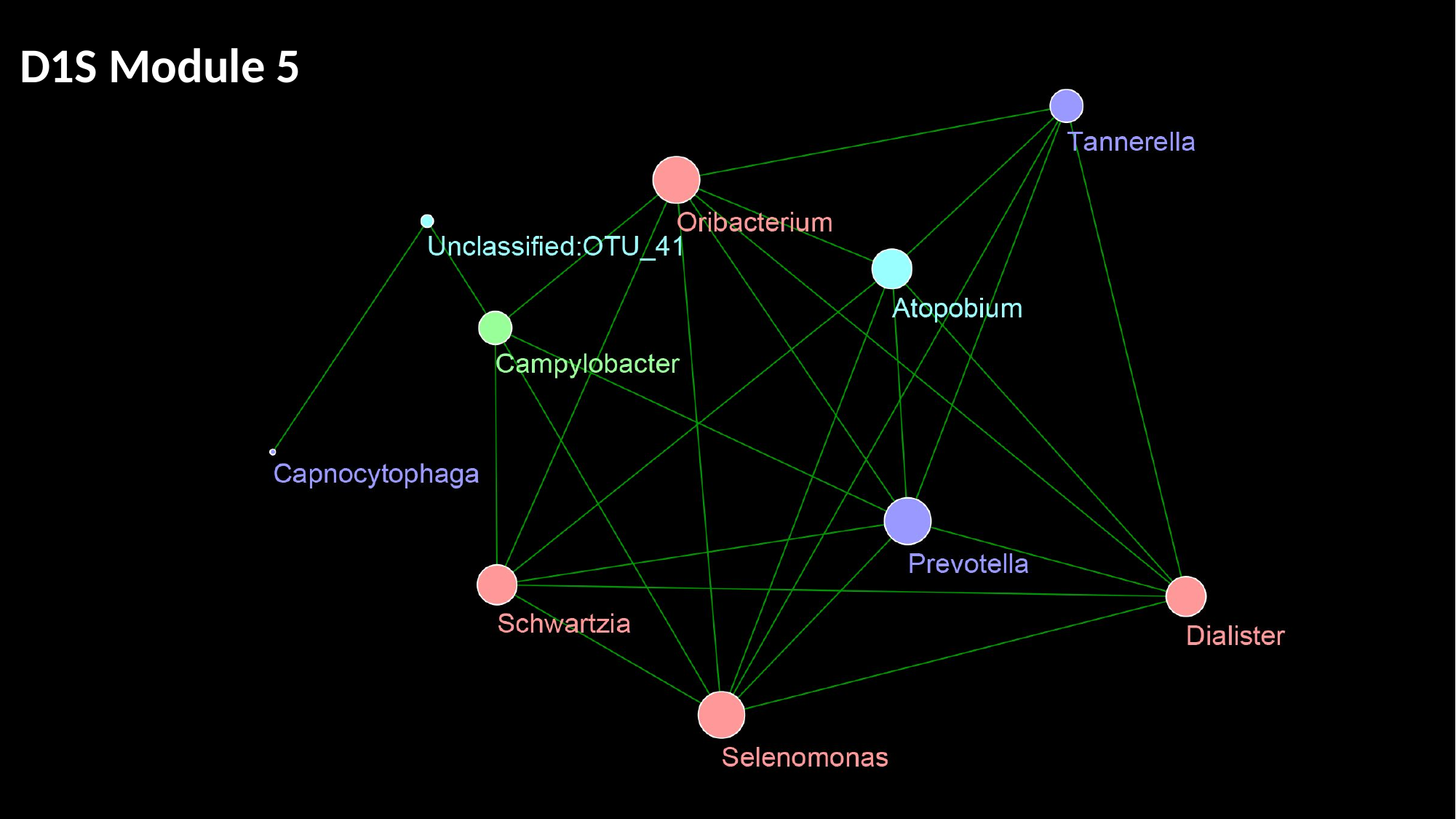

D1S Module 5

## Slide 64
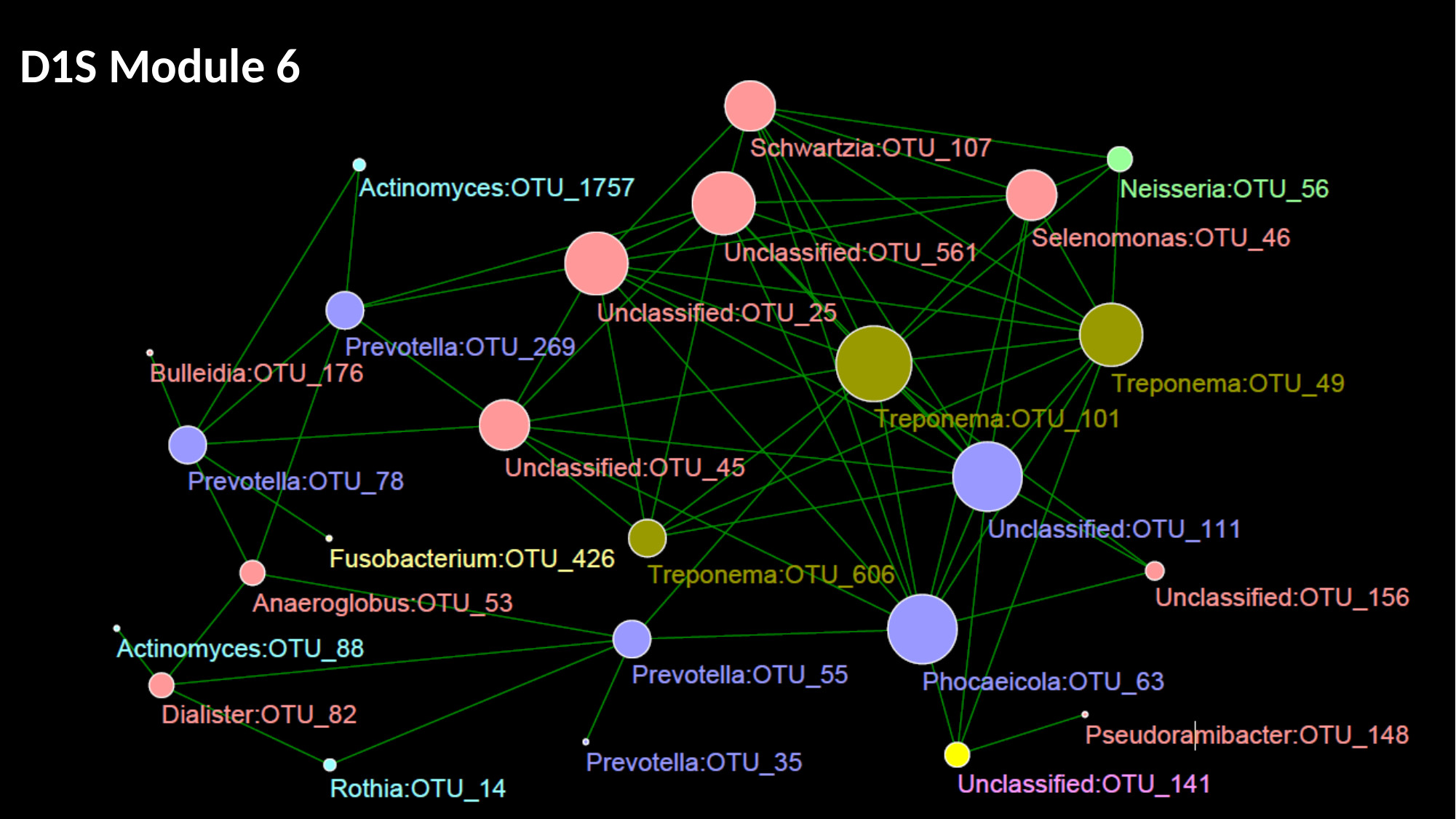

D1S Module 6

## Slide 65
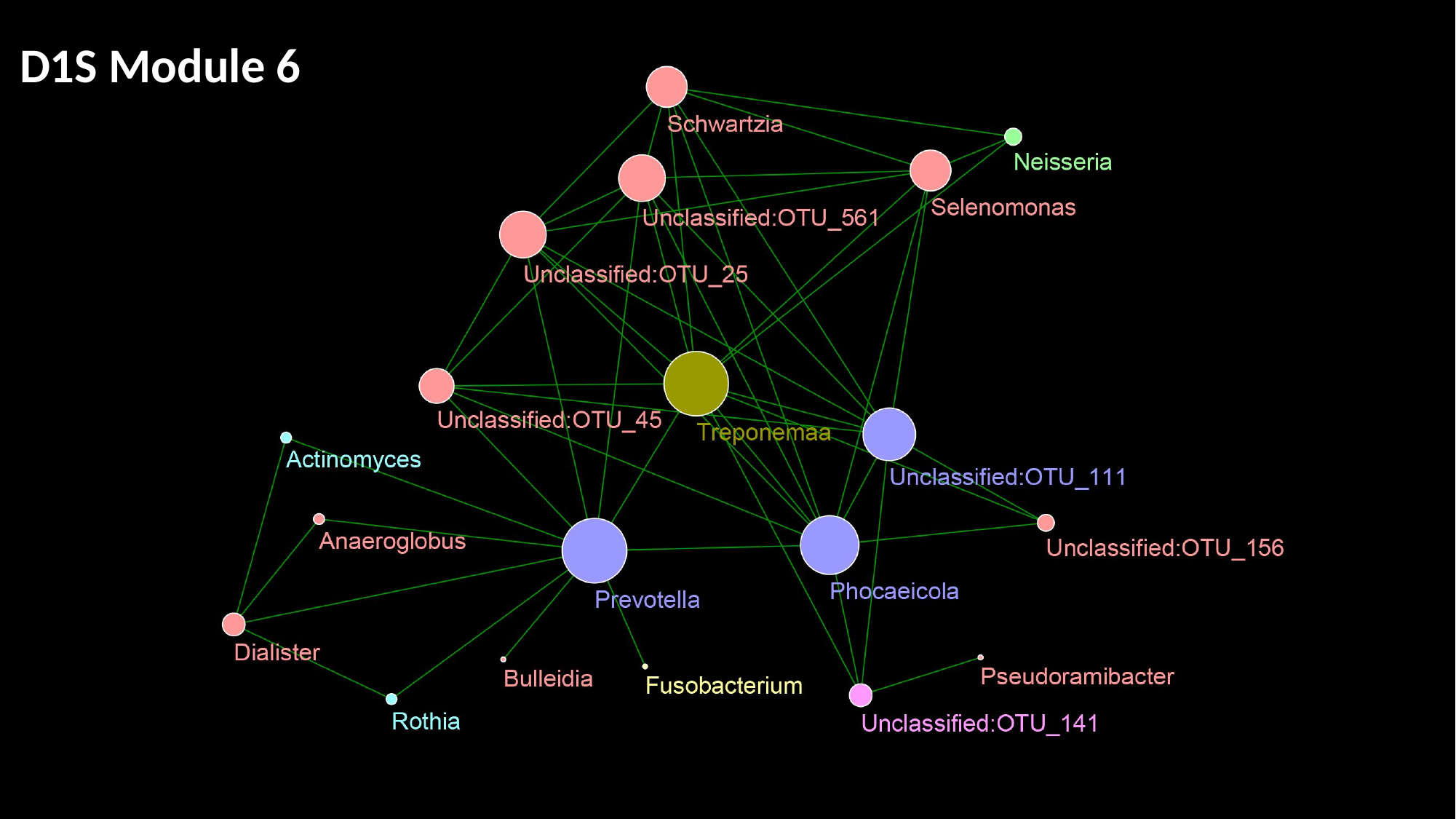

D1S Module 6

## Slide 66
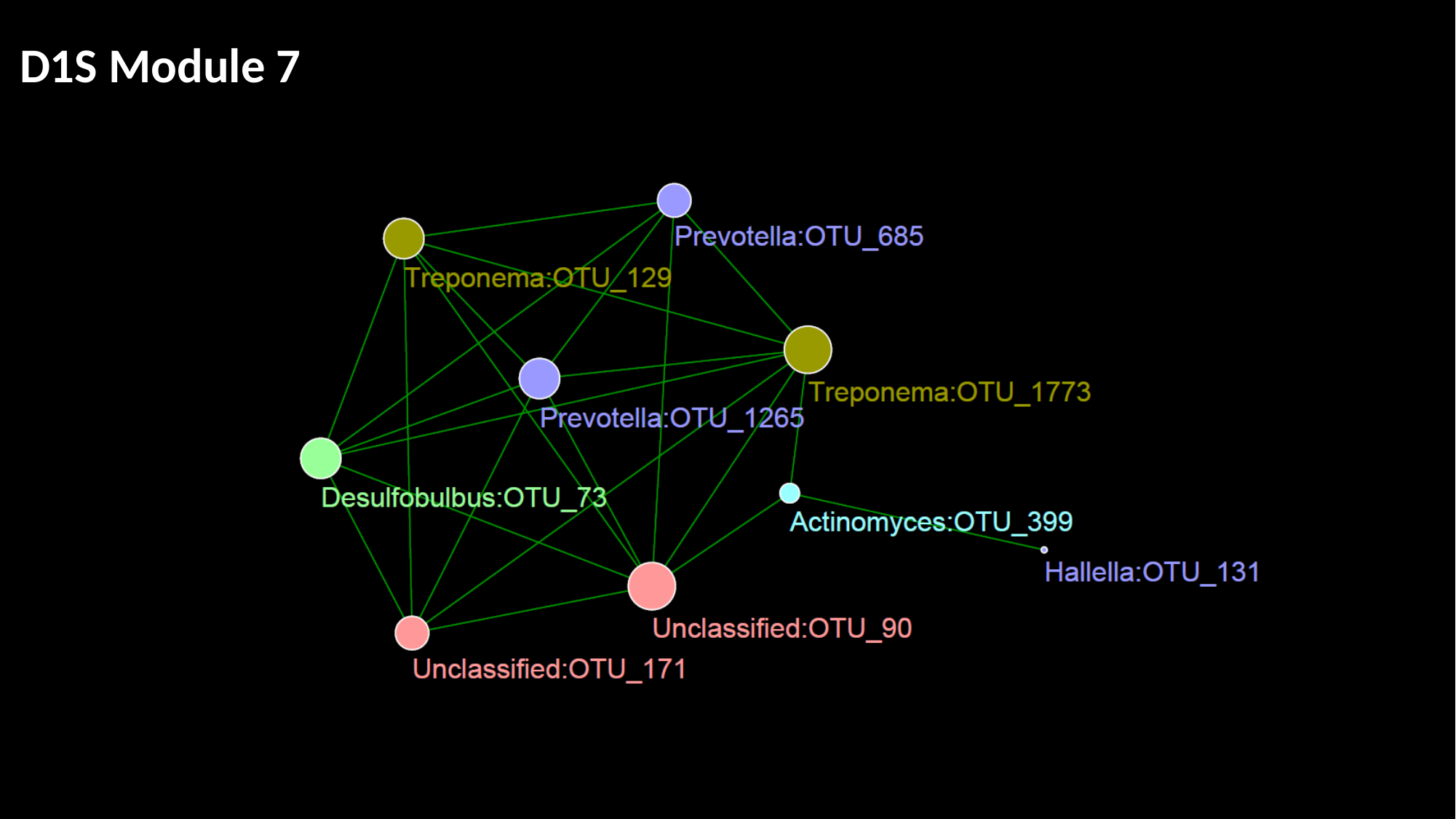

D1S Module 7

## Slide 67
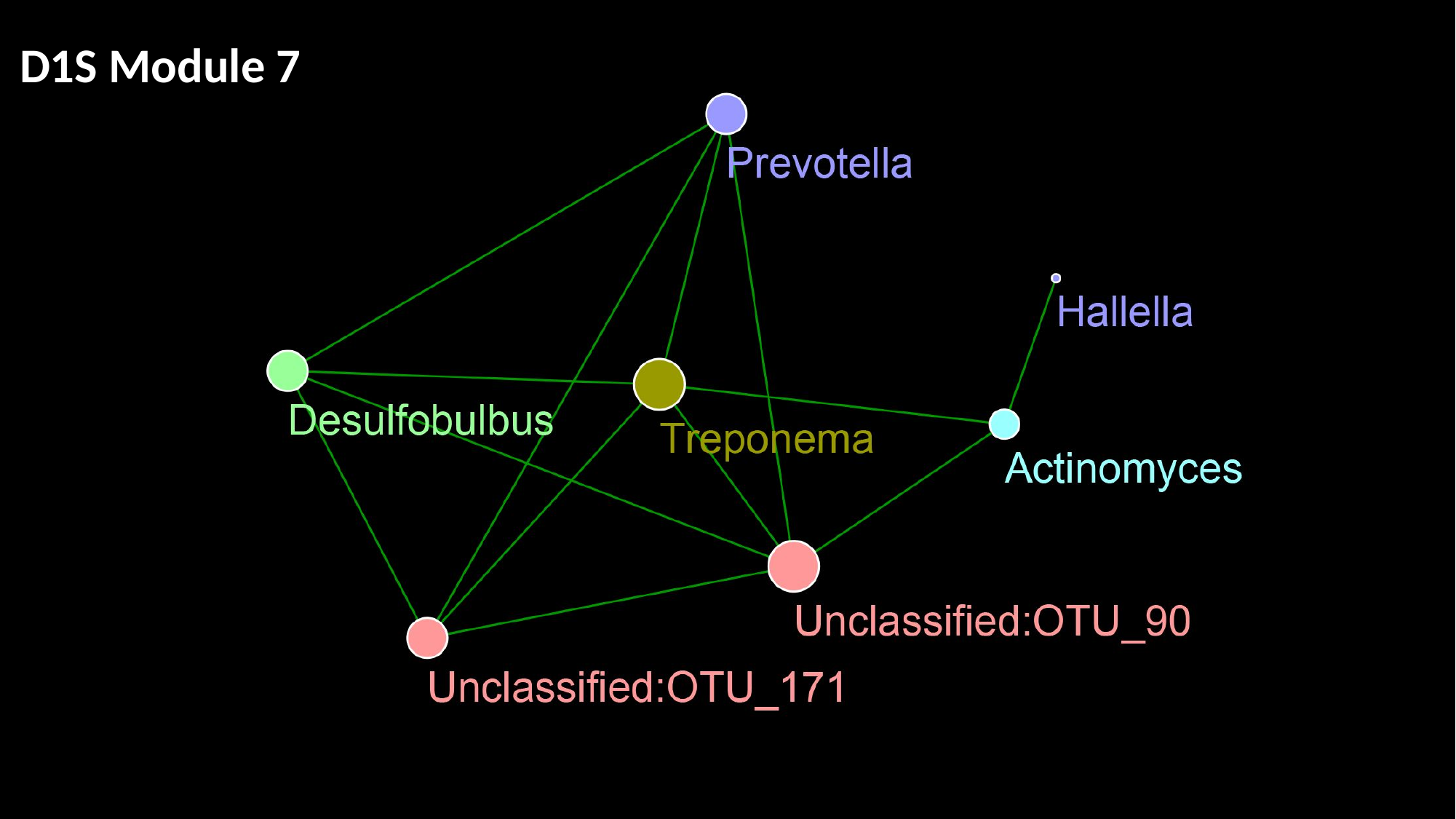

D1S Module 7

## Slide 68
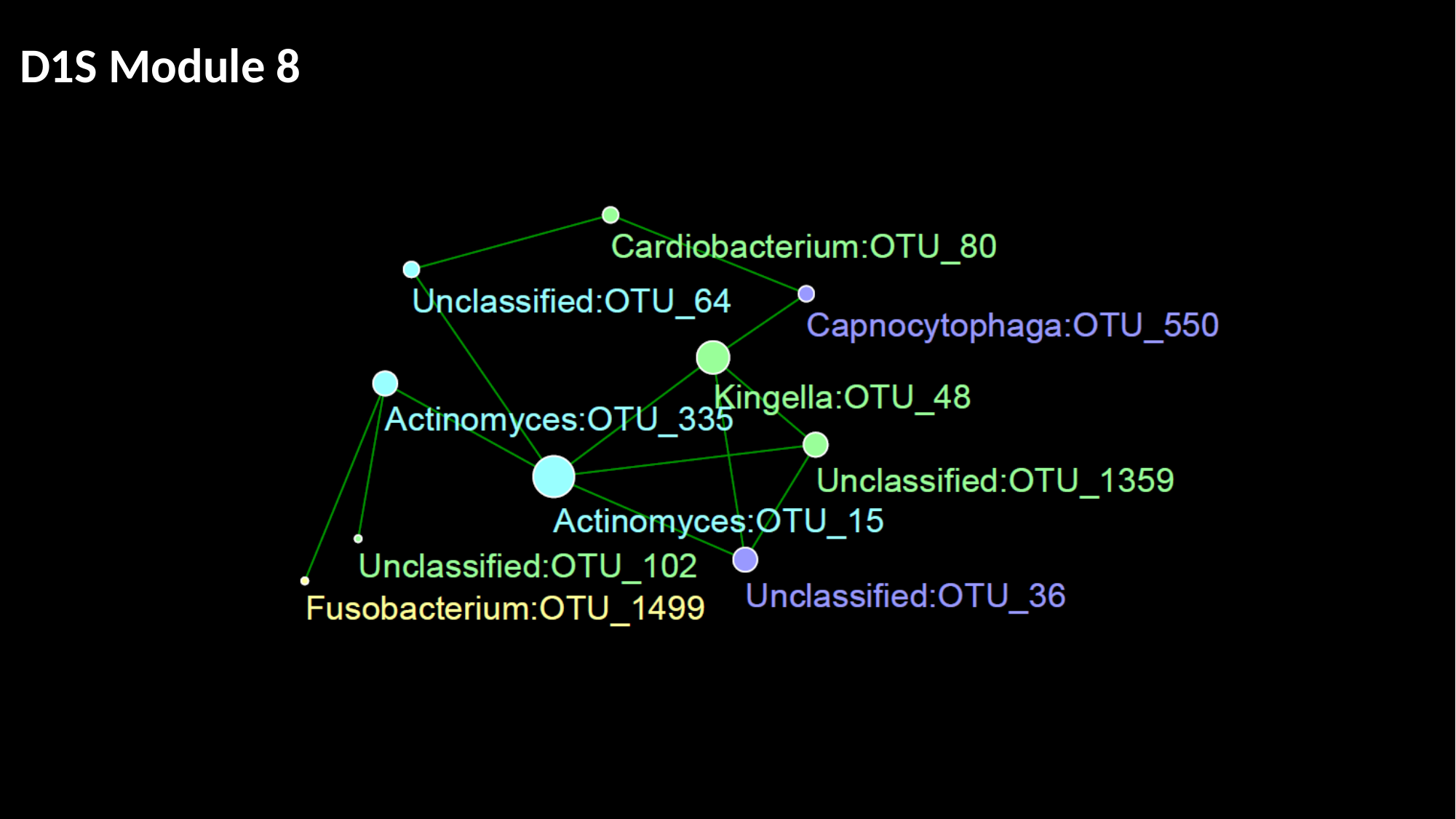

D1S Module 8

## Slide 69
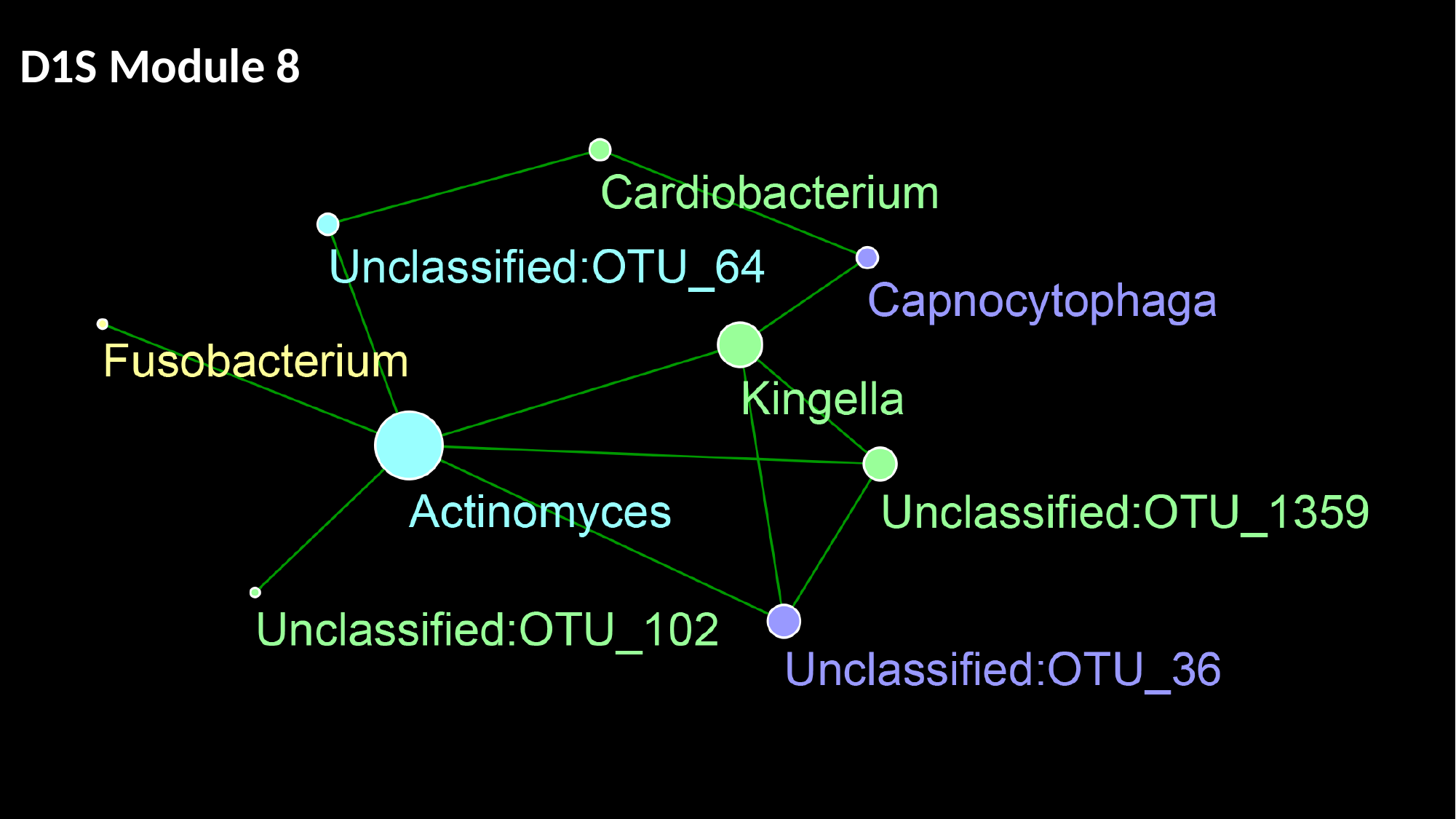

D1S Module 8

## Slide 70
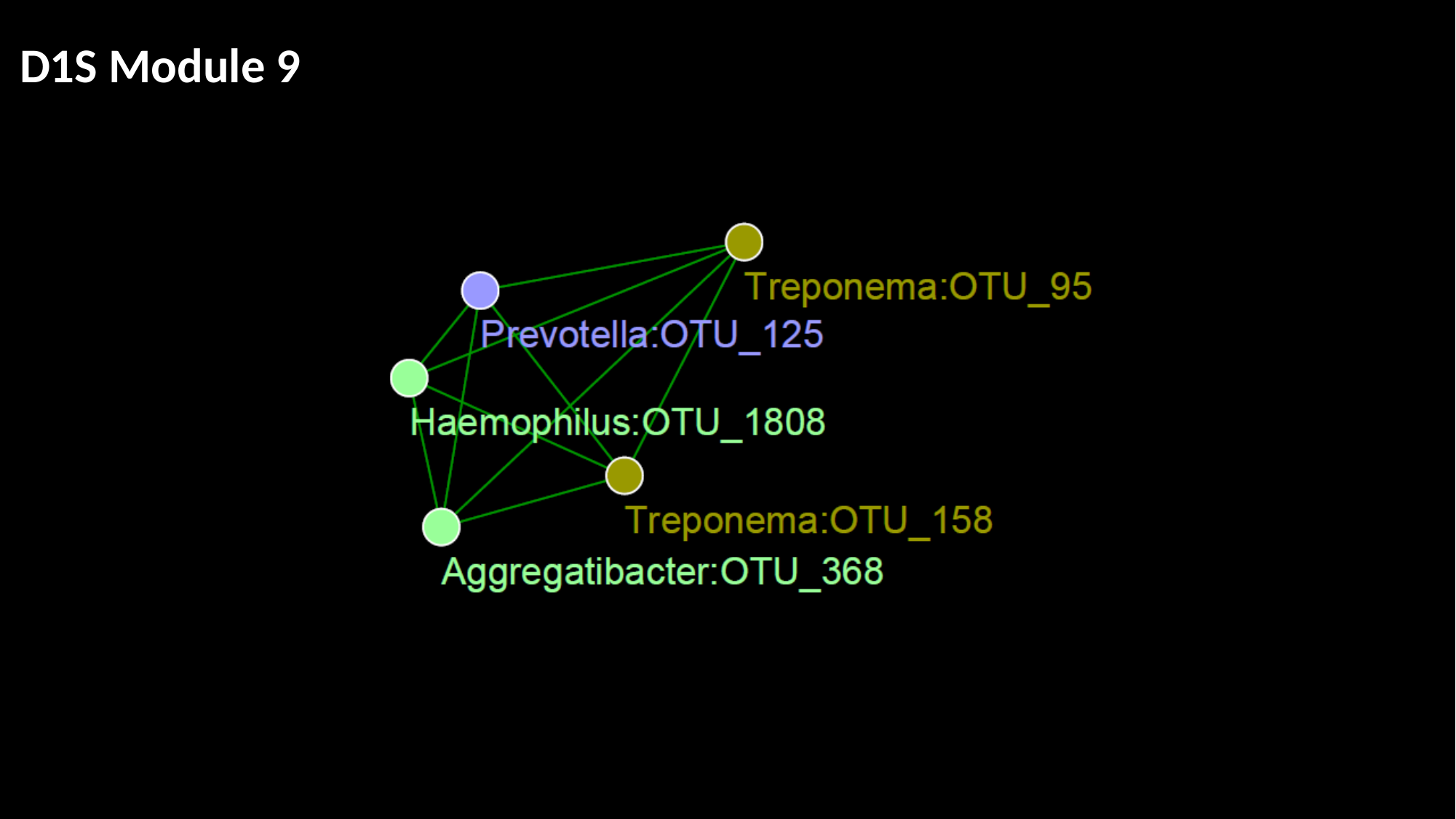

D1S Module 9

## Slide 71
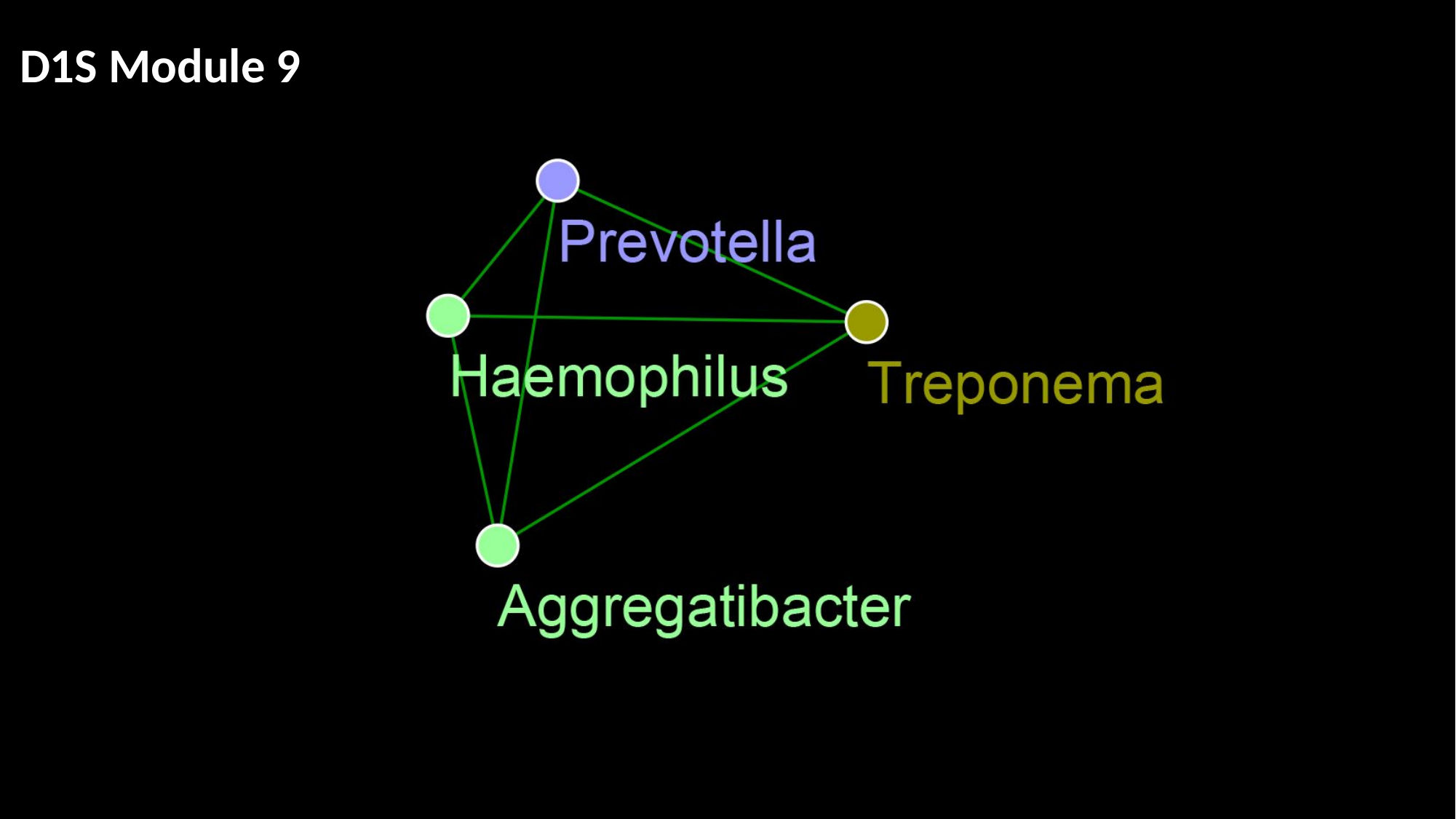

D1S Module 9

## Slide 72
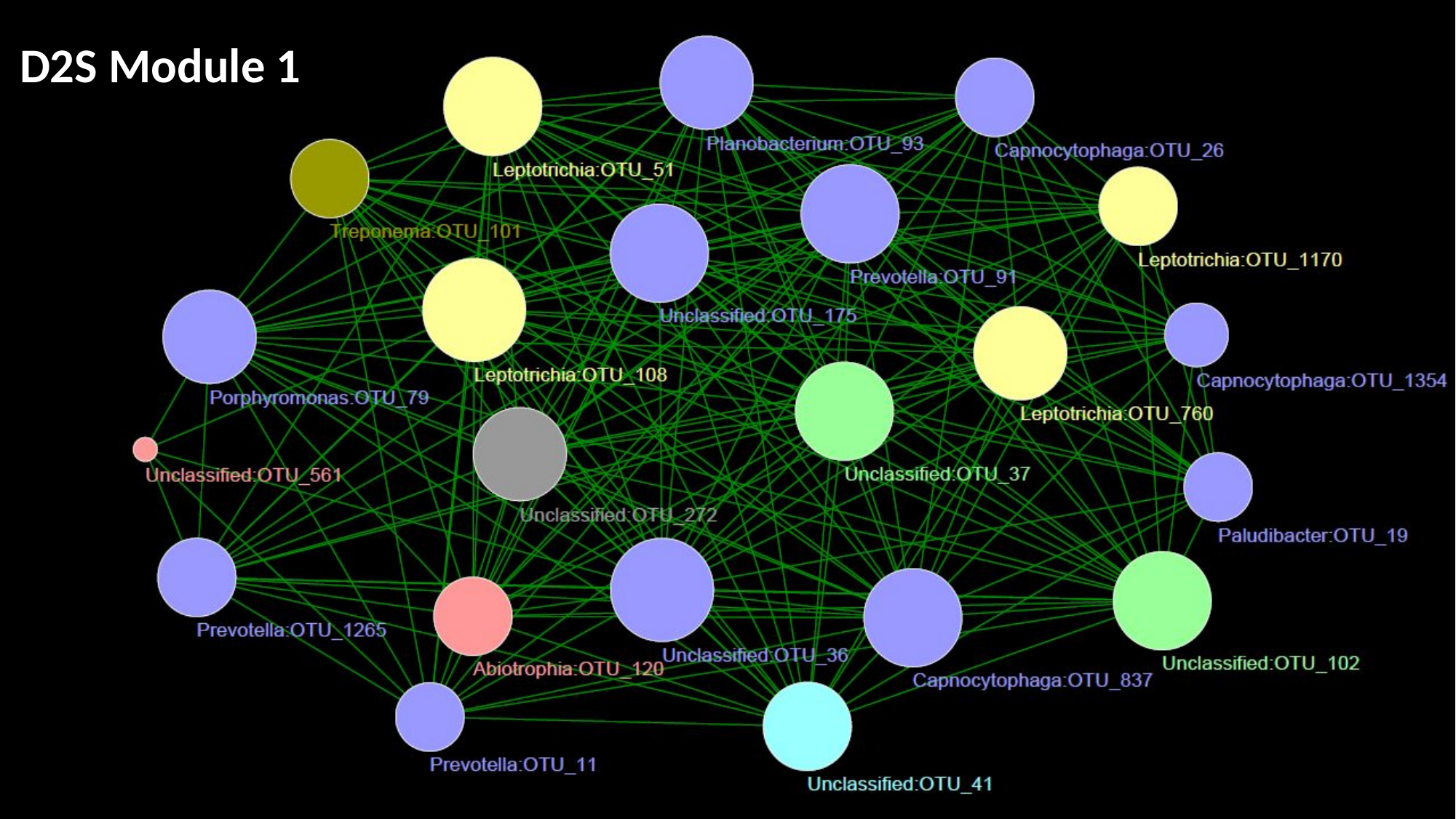

D2S Module 1

## Slide 73
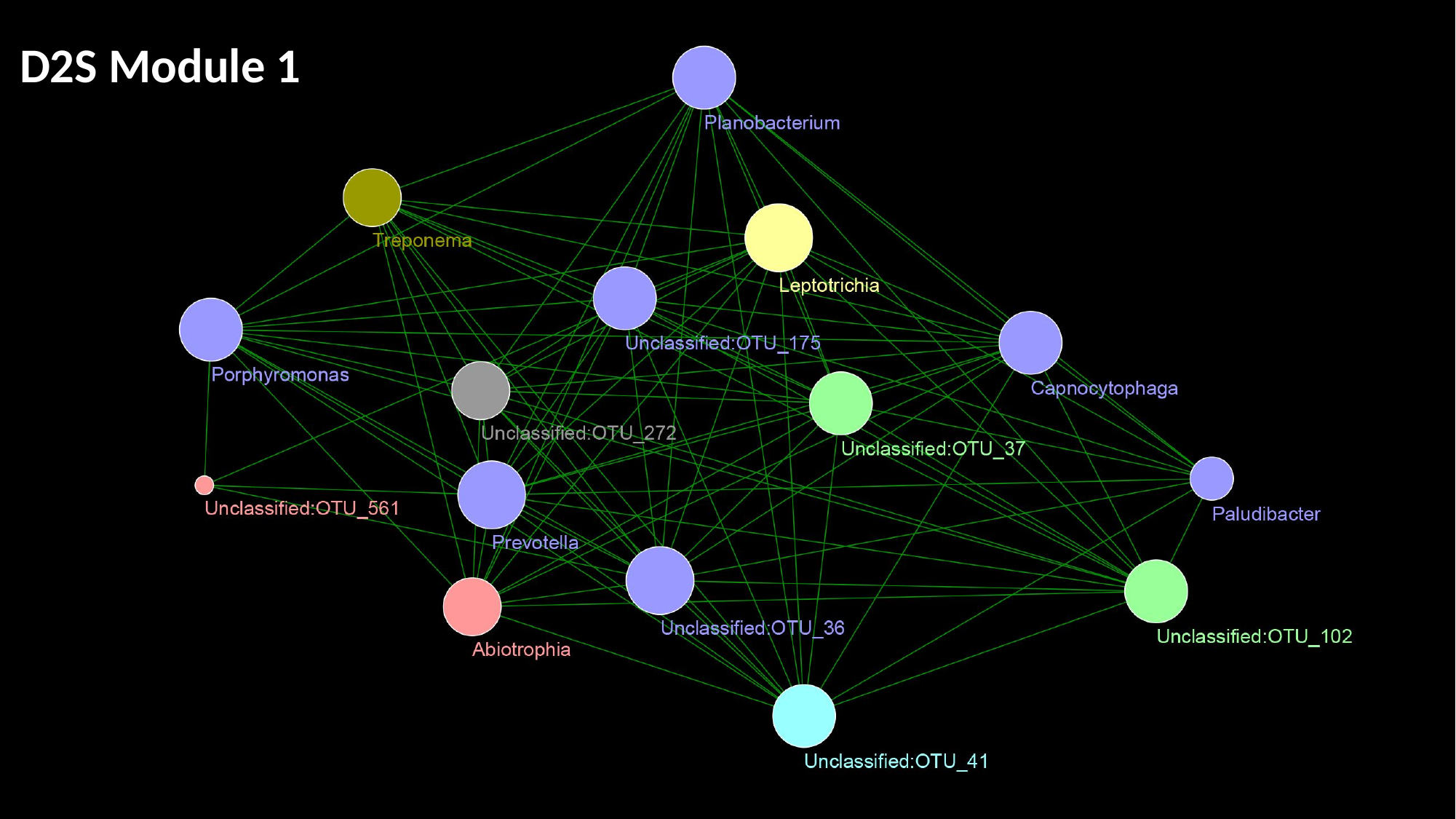

D2S Module 1

## Slide 74
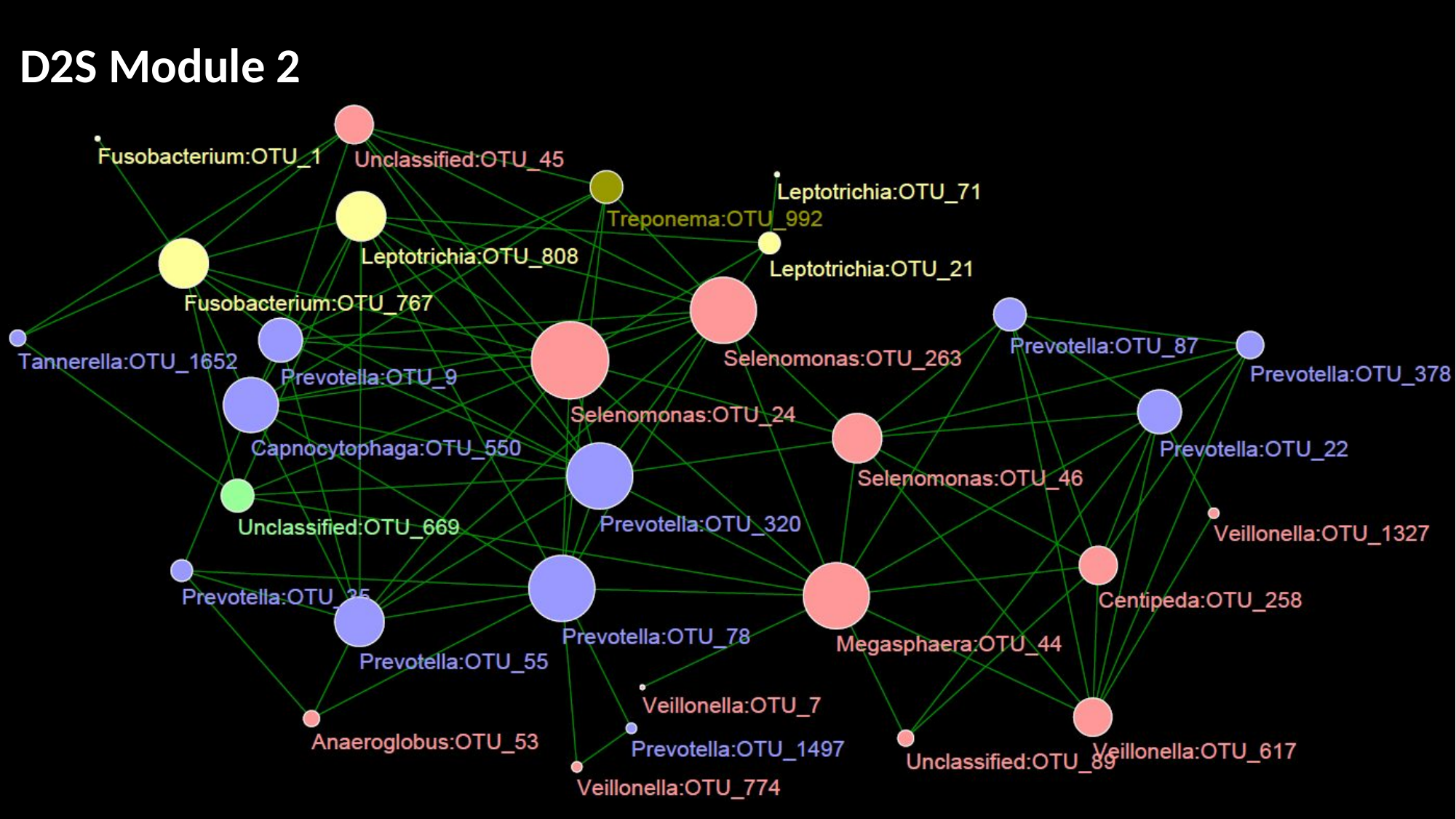

D2S Module 2

## Slide 75
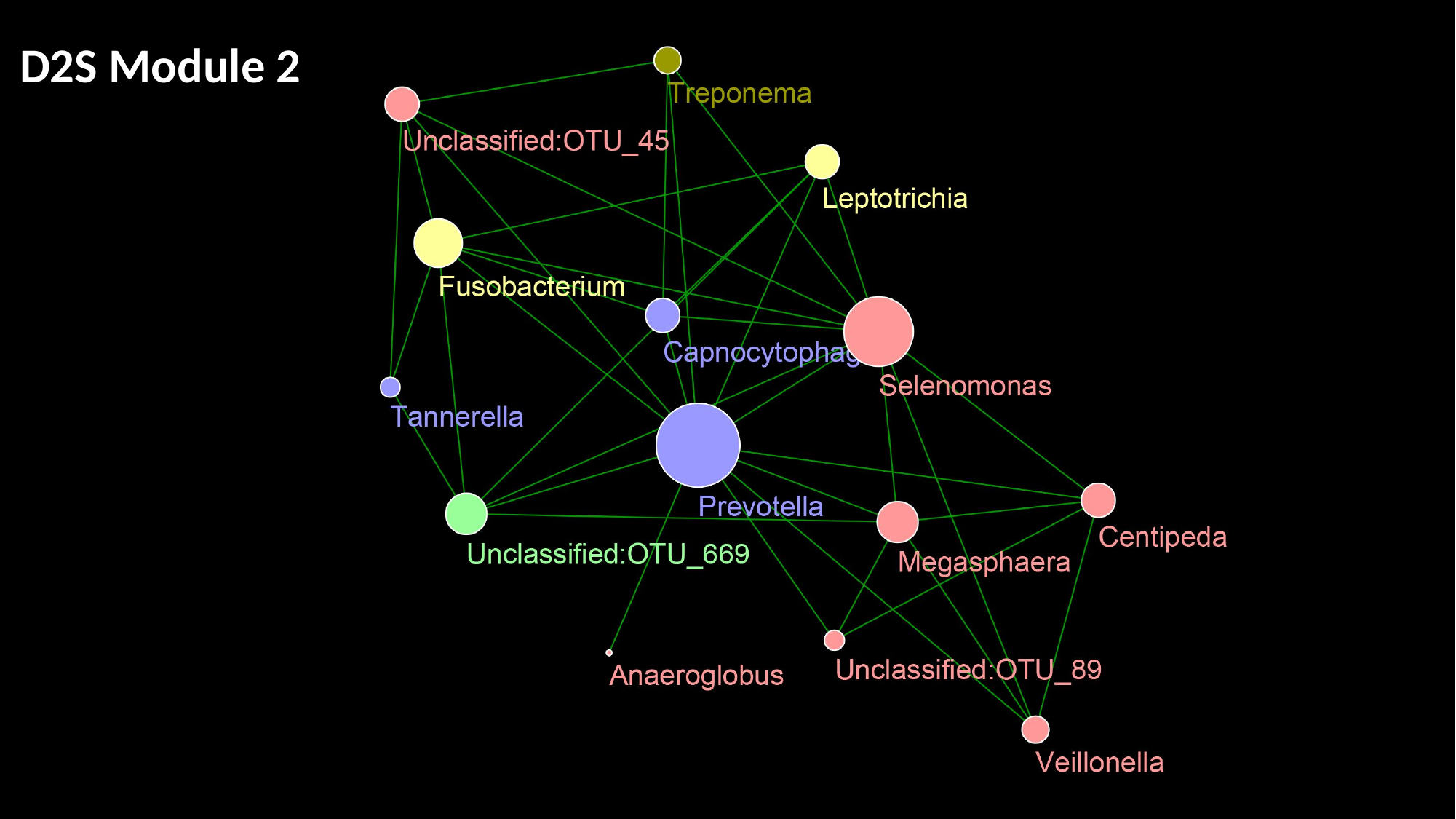

D2S Module 2

## Slide 76
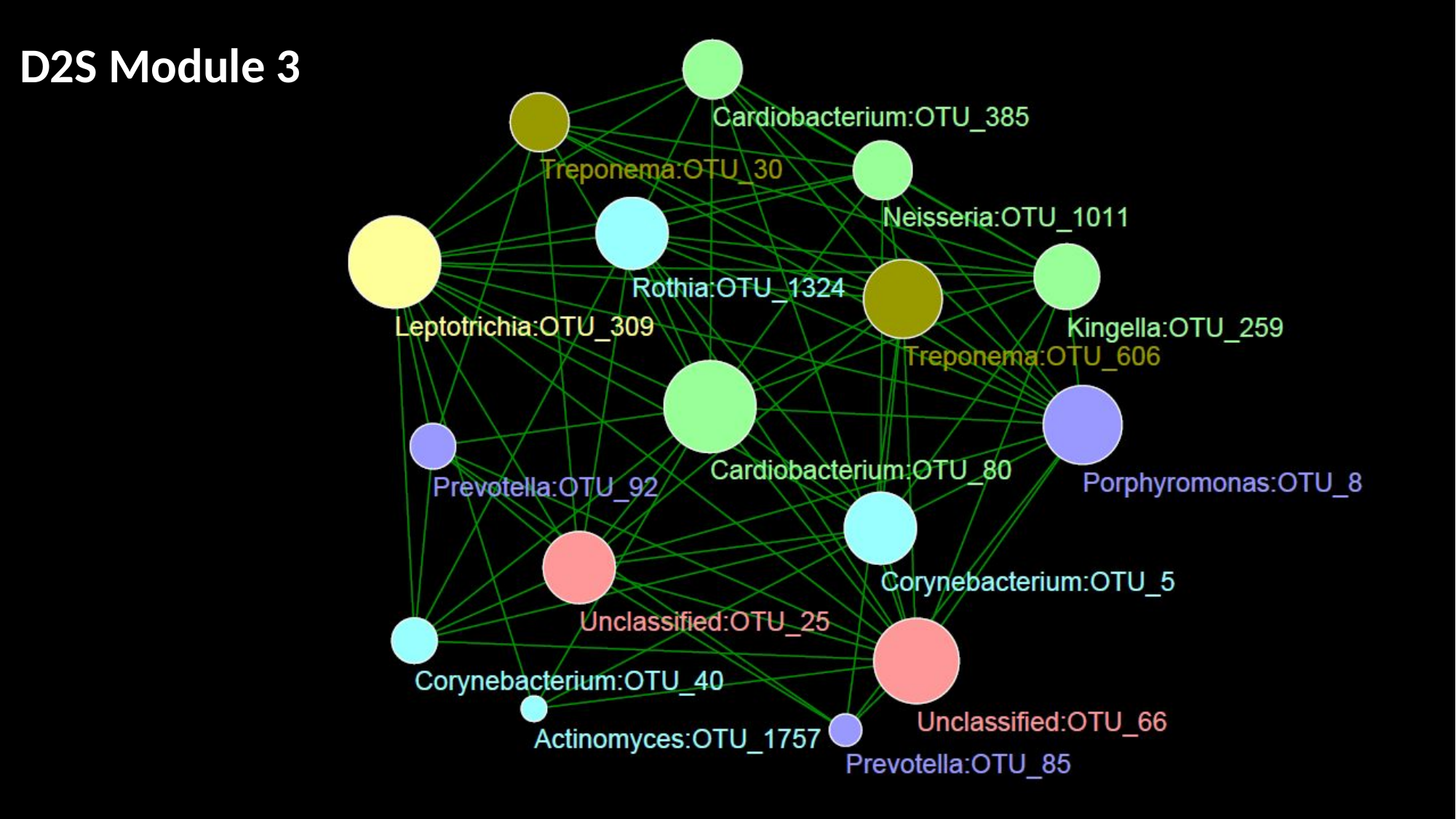

D2S Module 3

## Slide 77
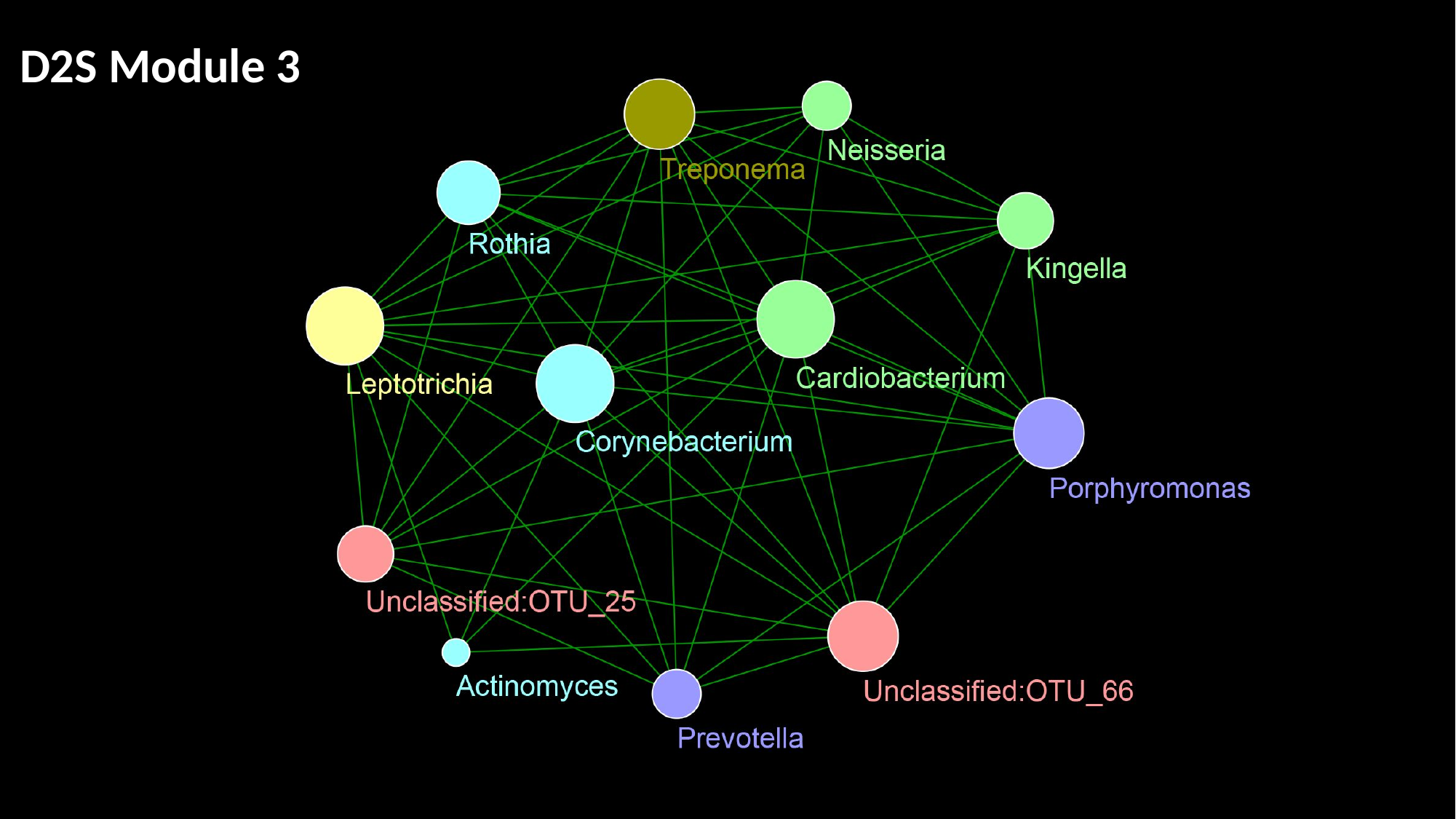

D2S Module 3

## Slide 78
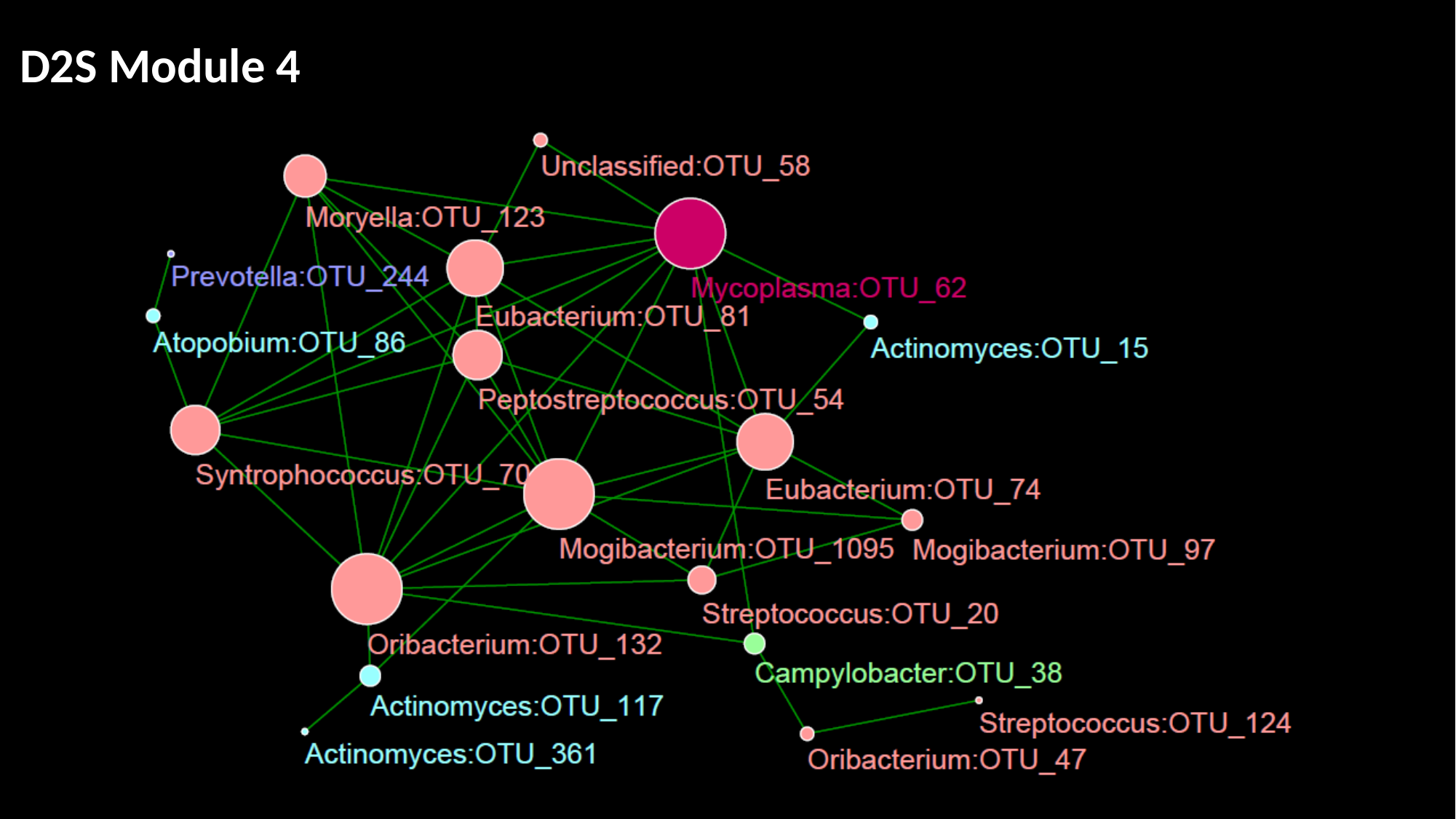

D2S Module 4

## Slide 79
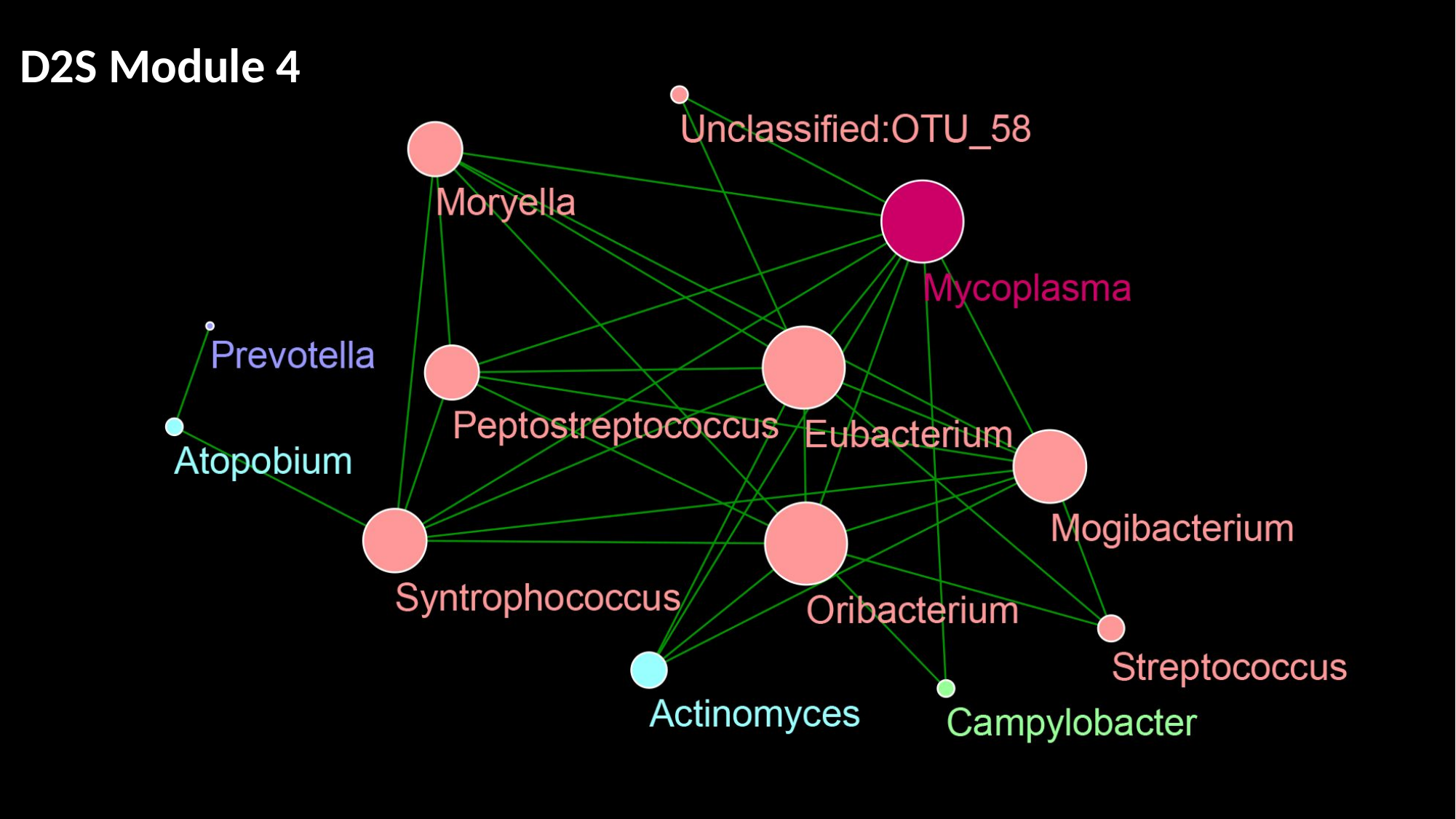

D2S Module 4

## Slide 80
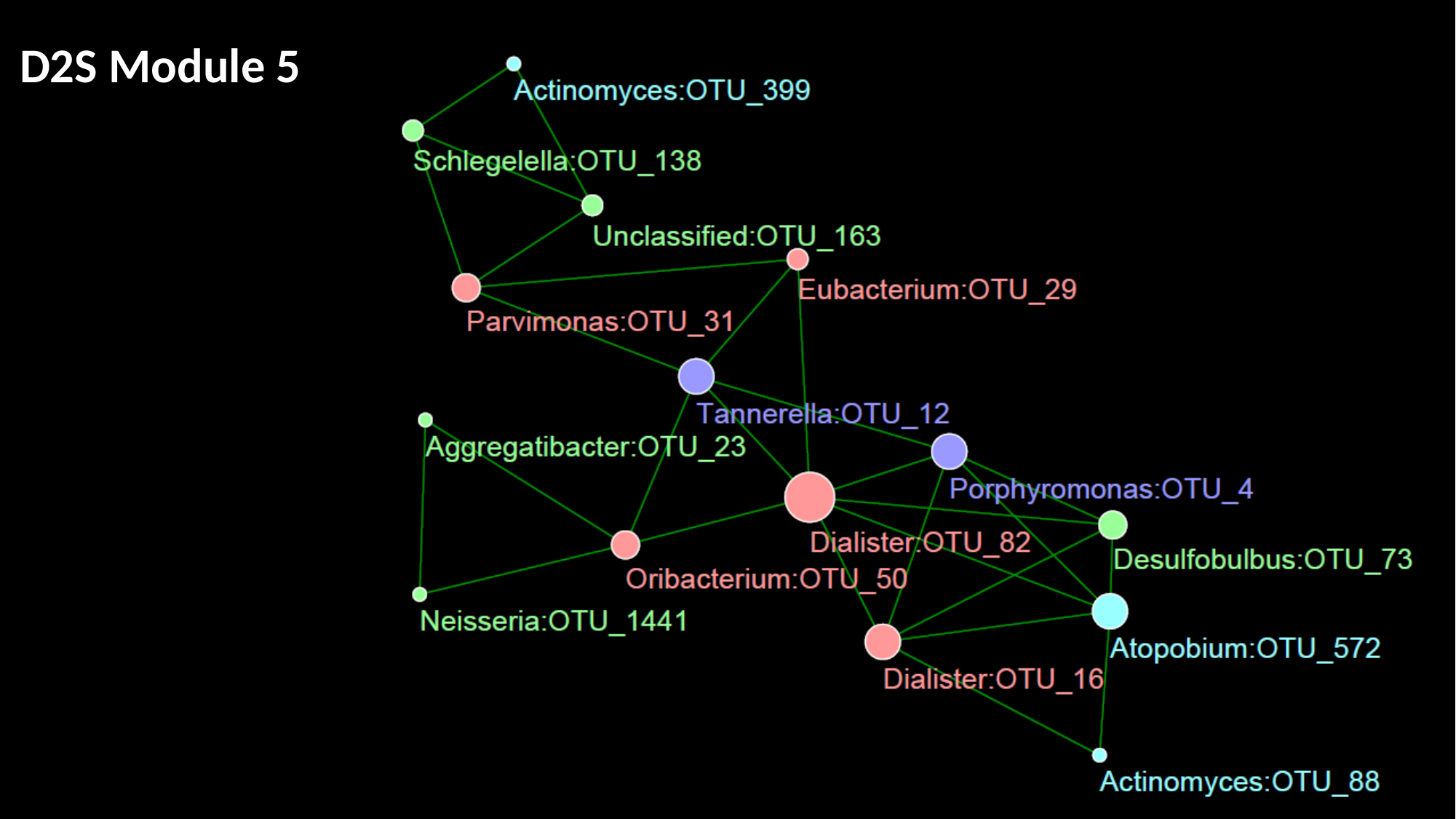

D2S Module 5

## Slide 81
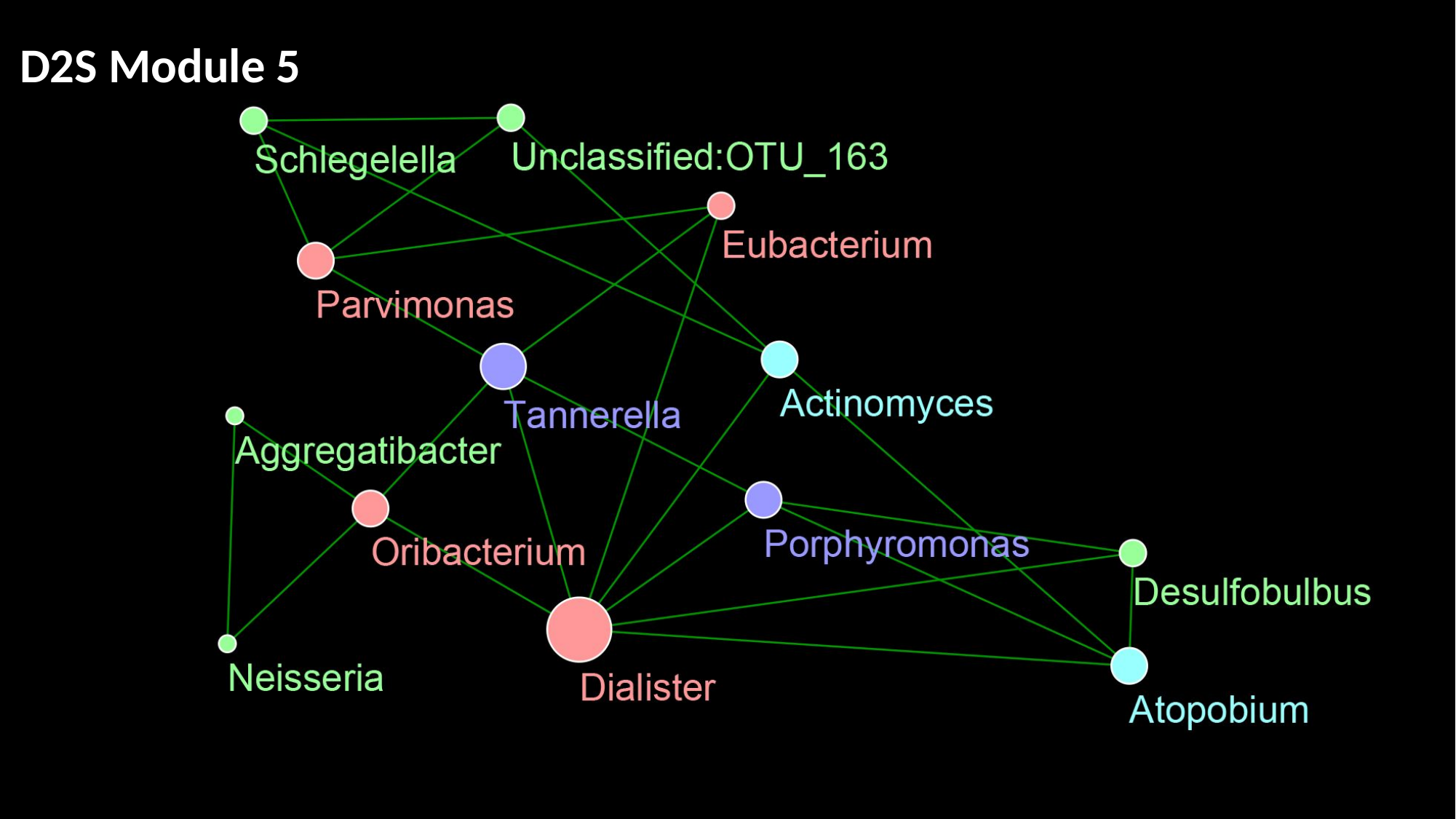

D2S Module 5

## Slide 82
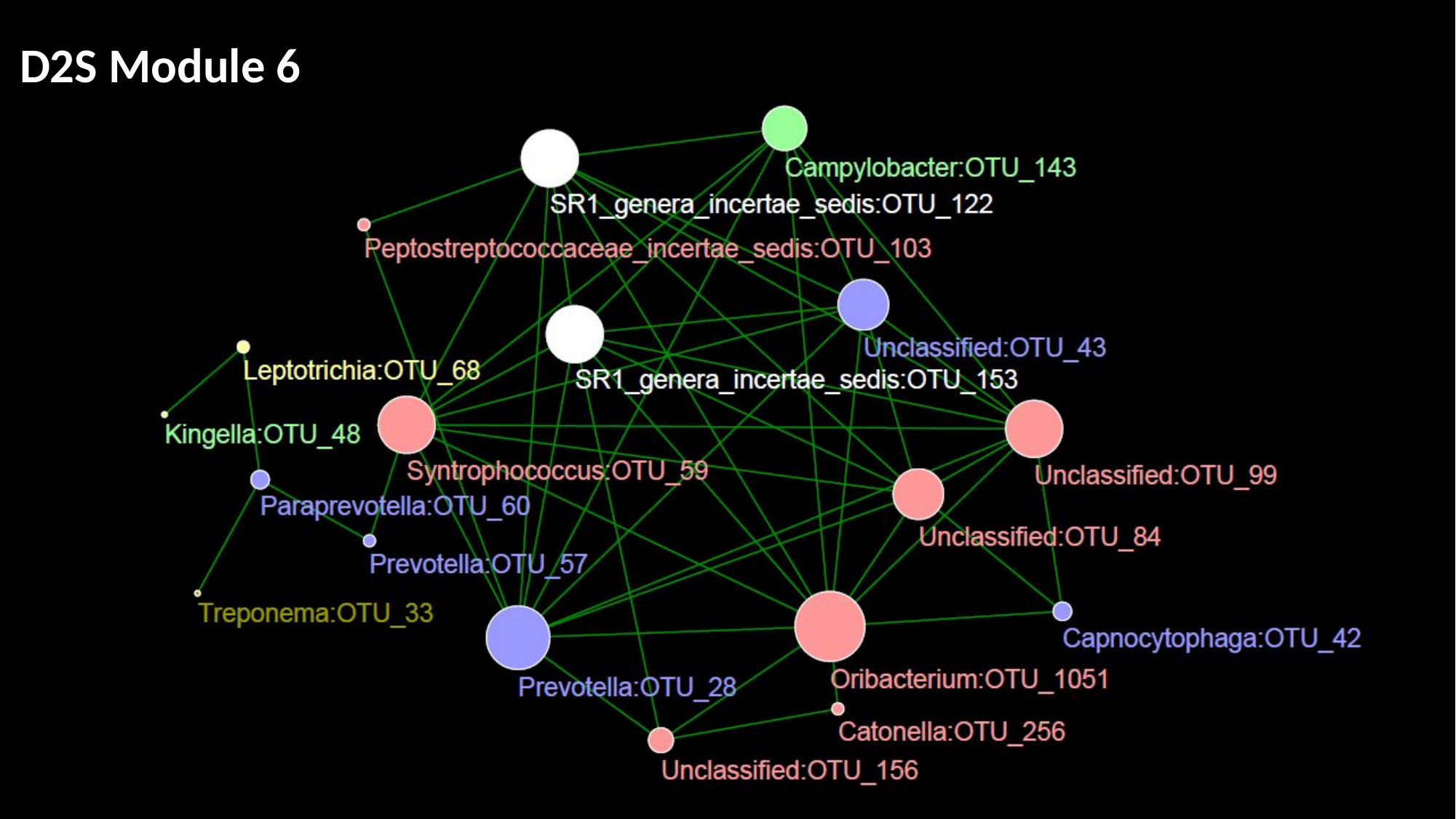

D2S Module 6

## Slide 83
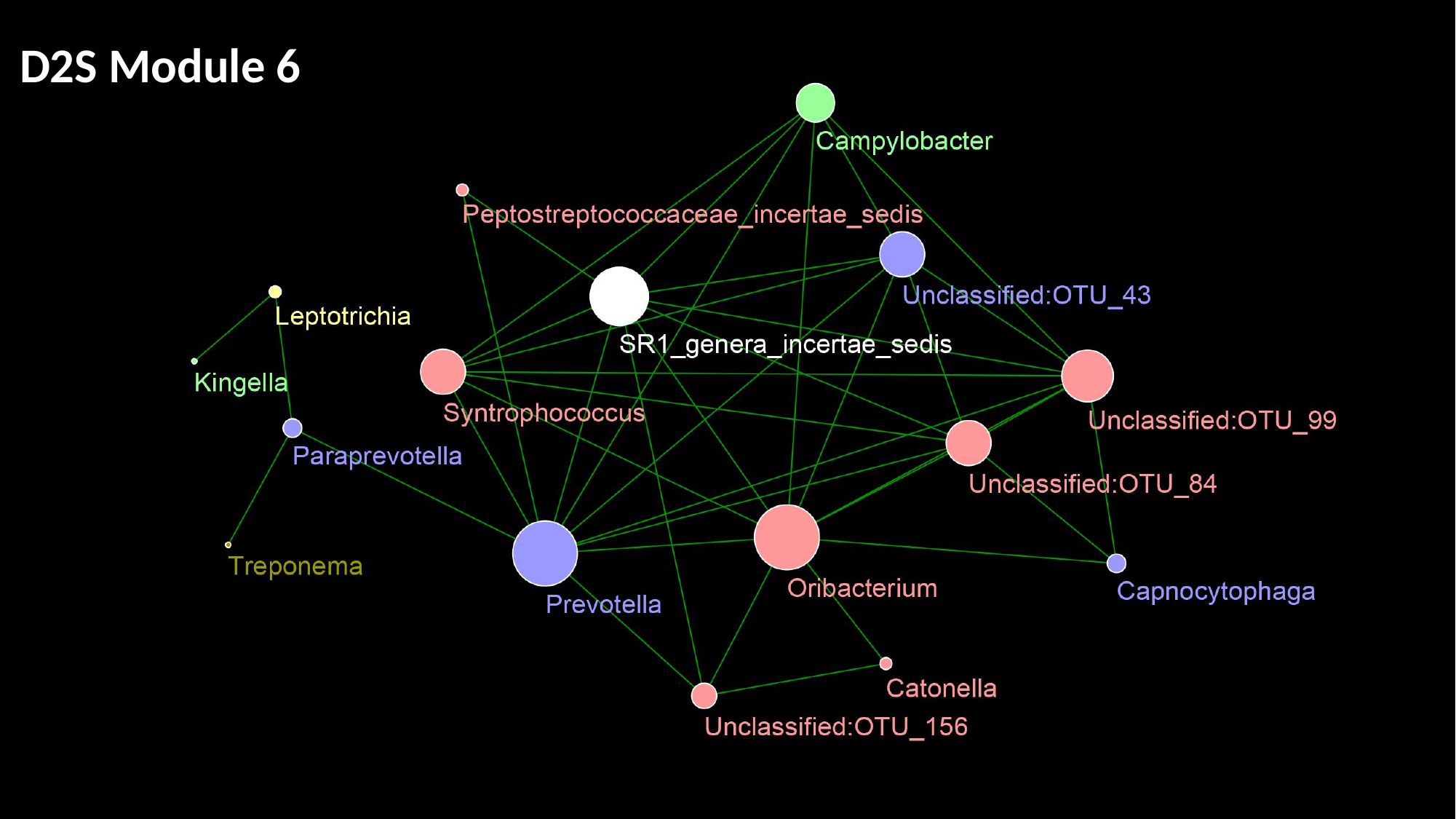

D2S Module 6

## Slide 84
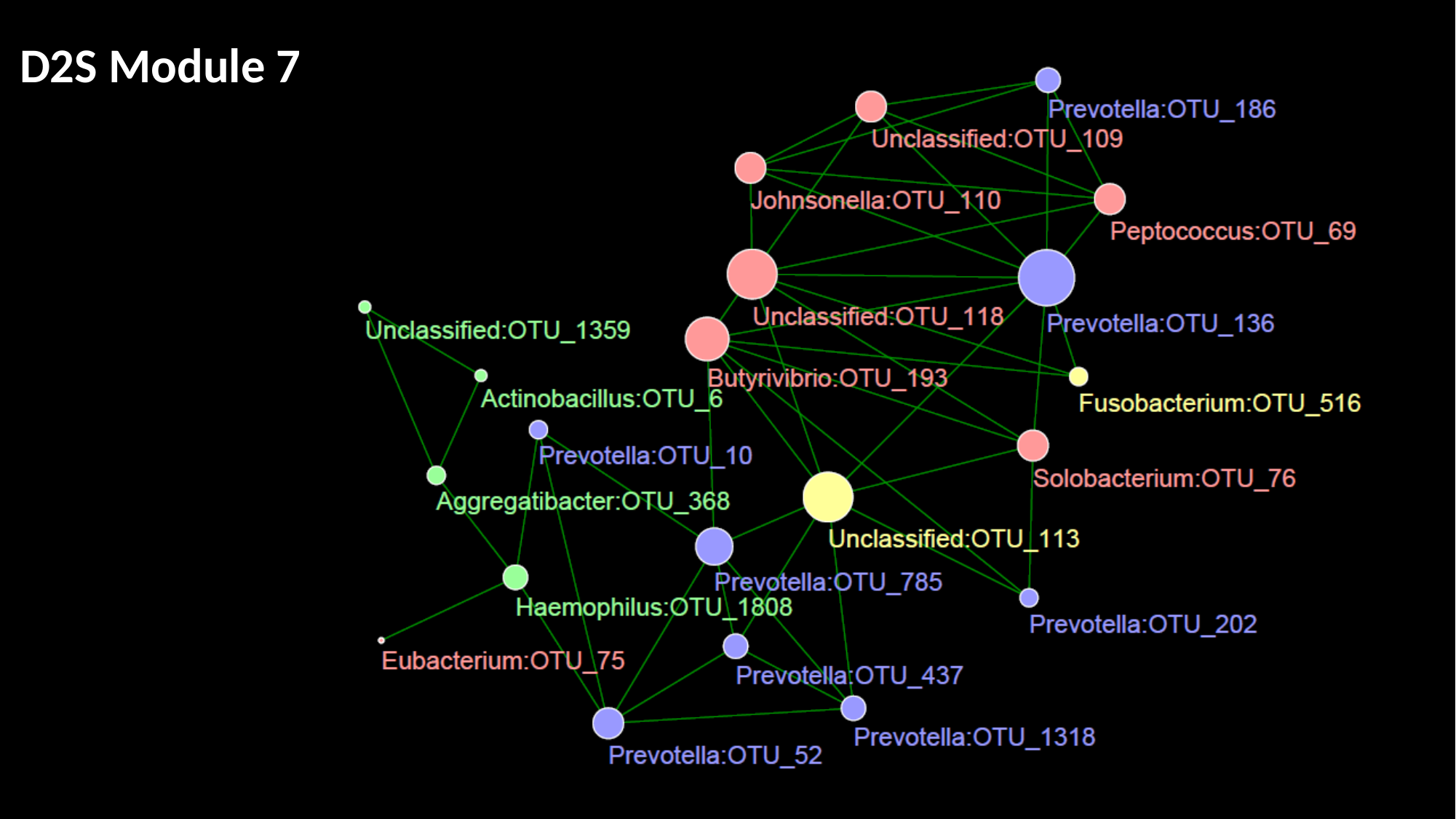

D2S Module 7

## Slide 85
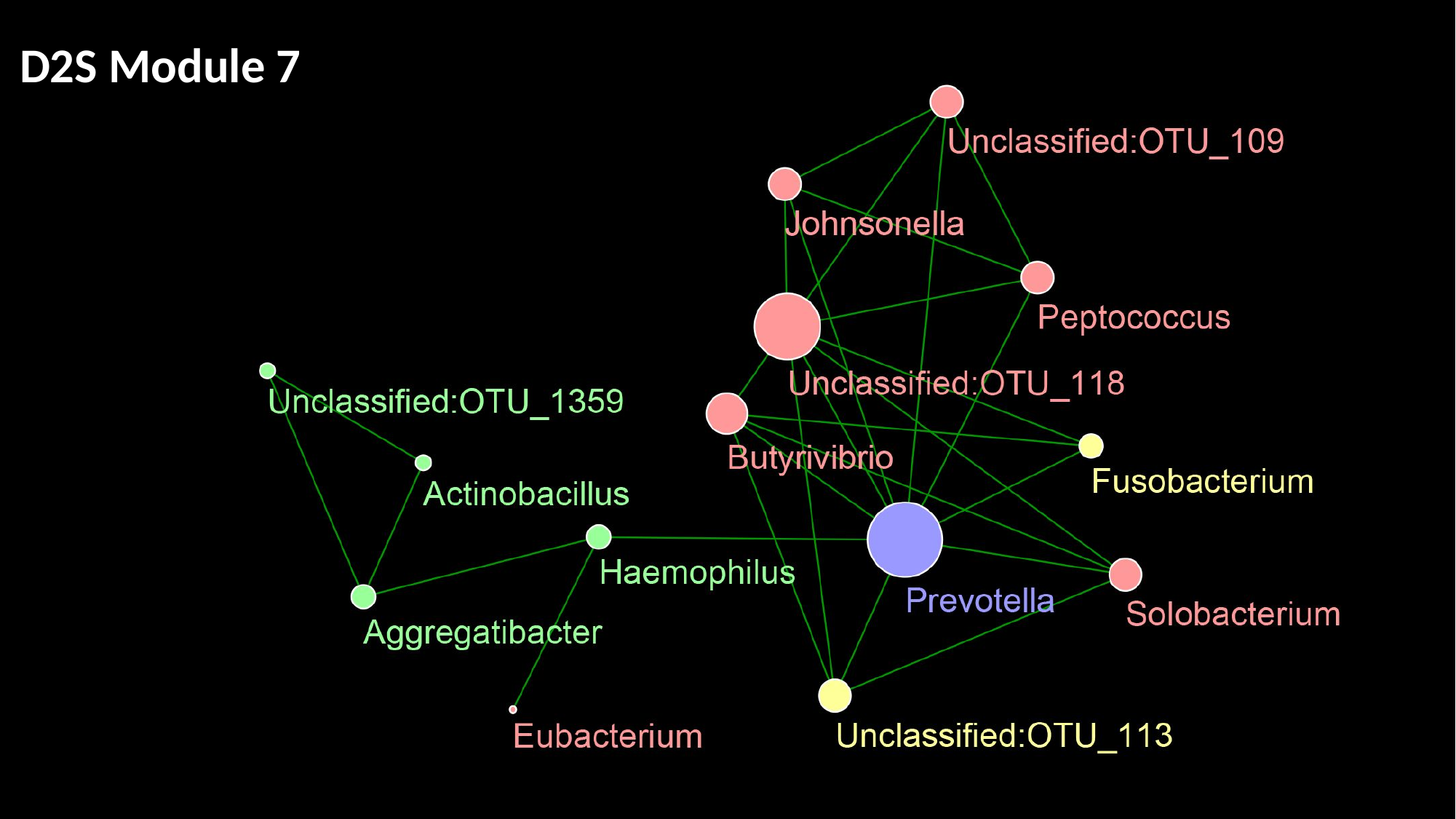

D2S Module 7

## Slide 86
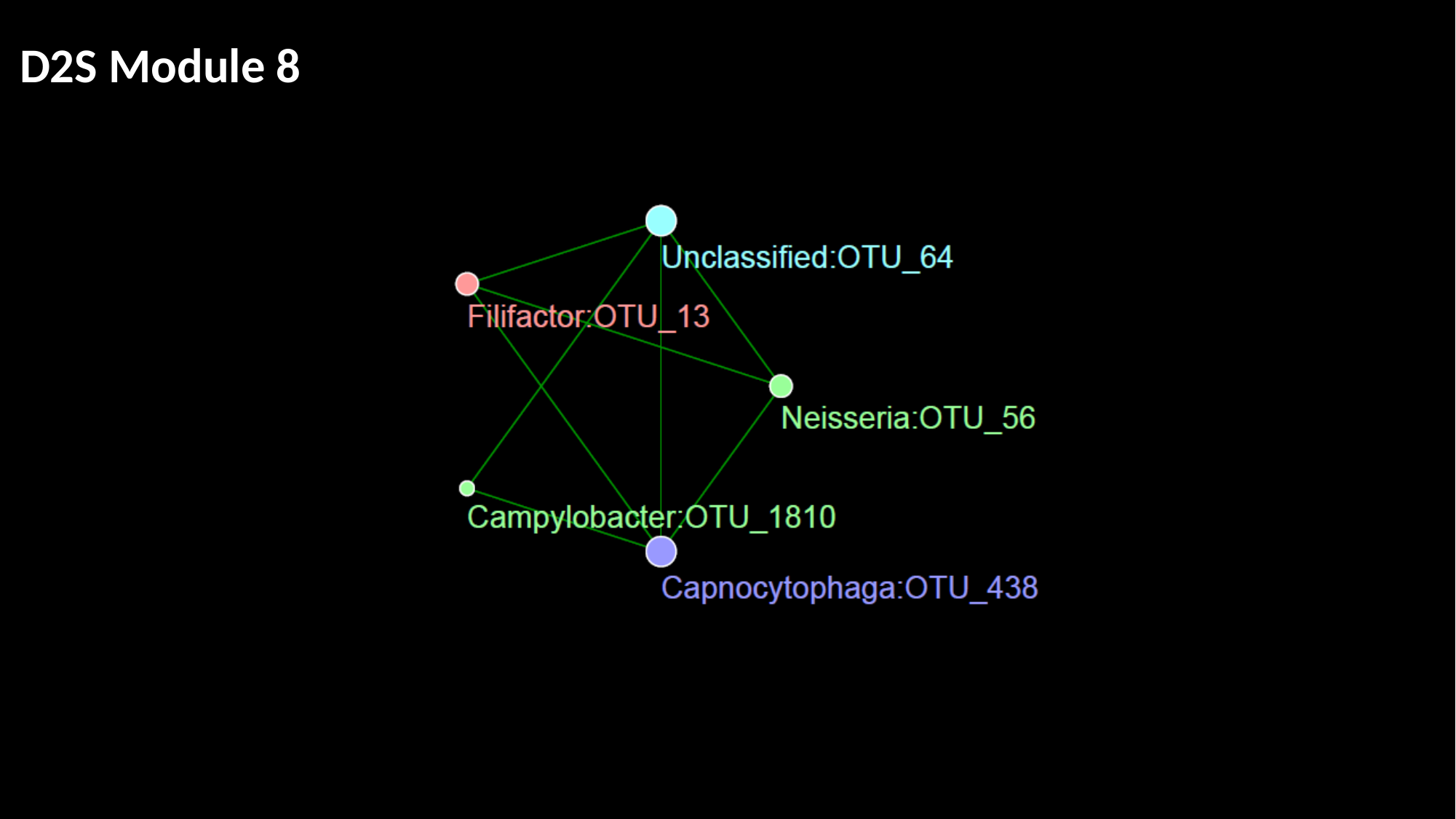

D2S Module 8

## Slide 87
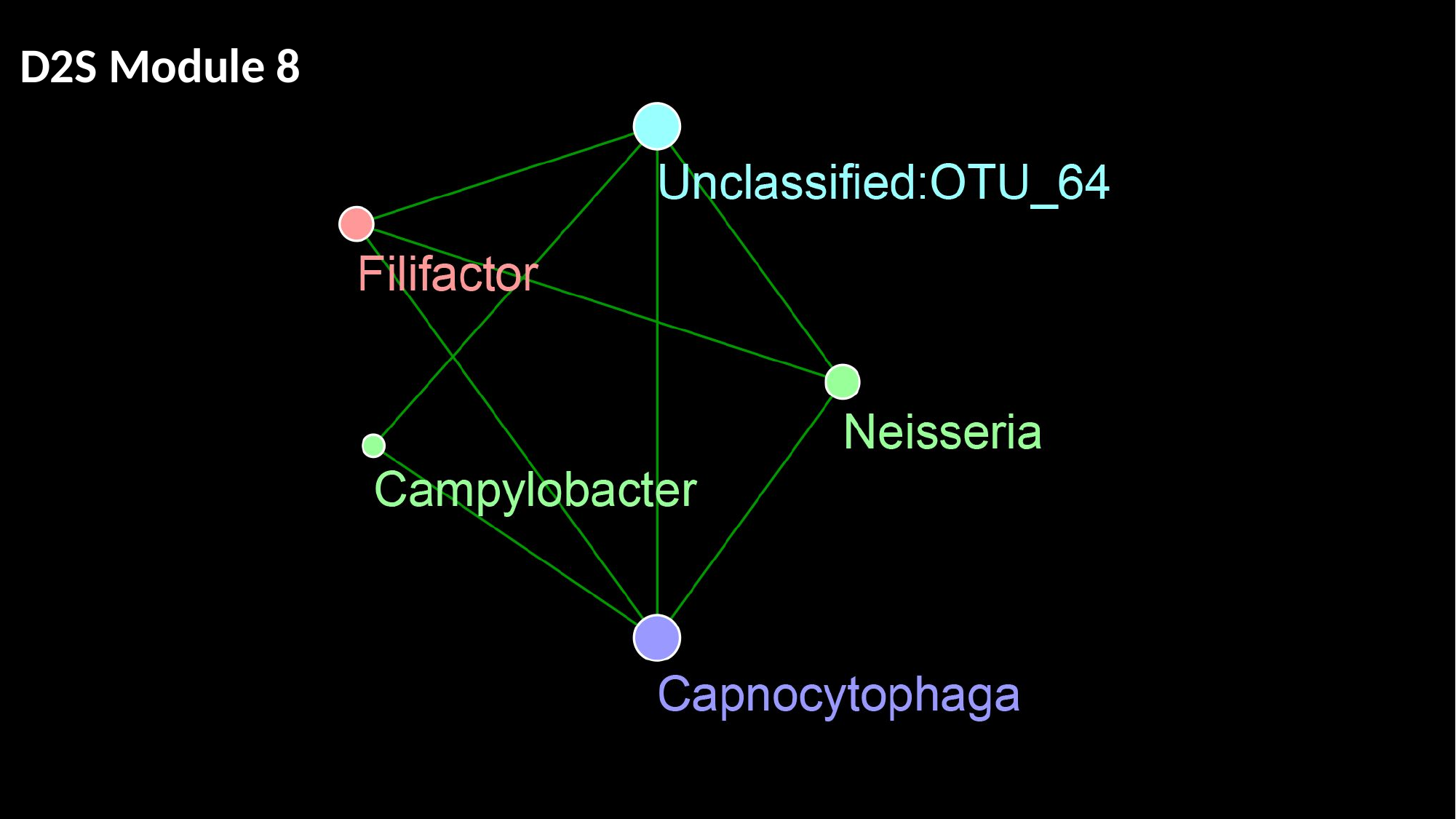

D2S Module 8

## Slide 88
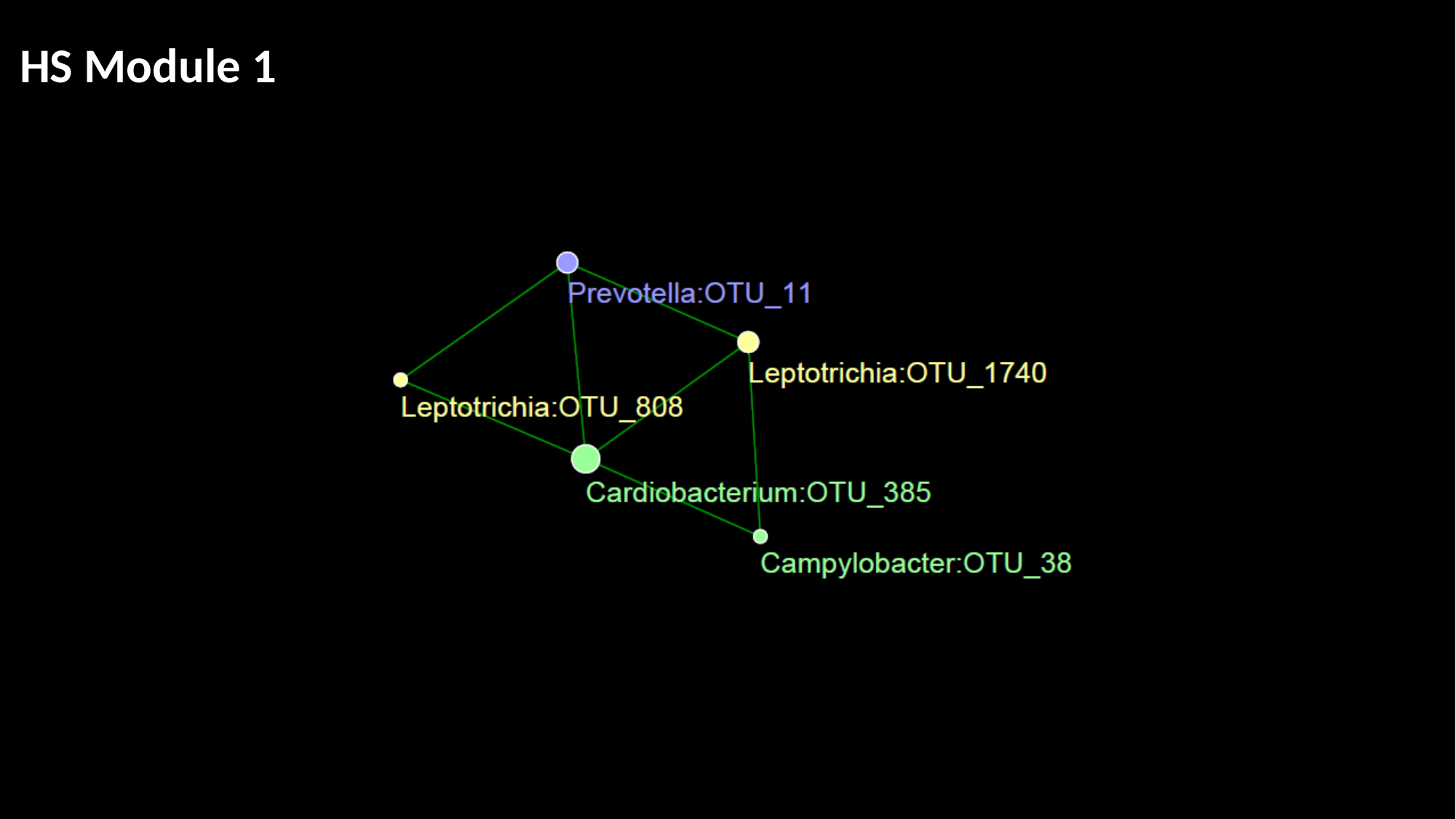

HS Module 1

## Slide 89
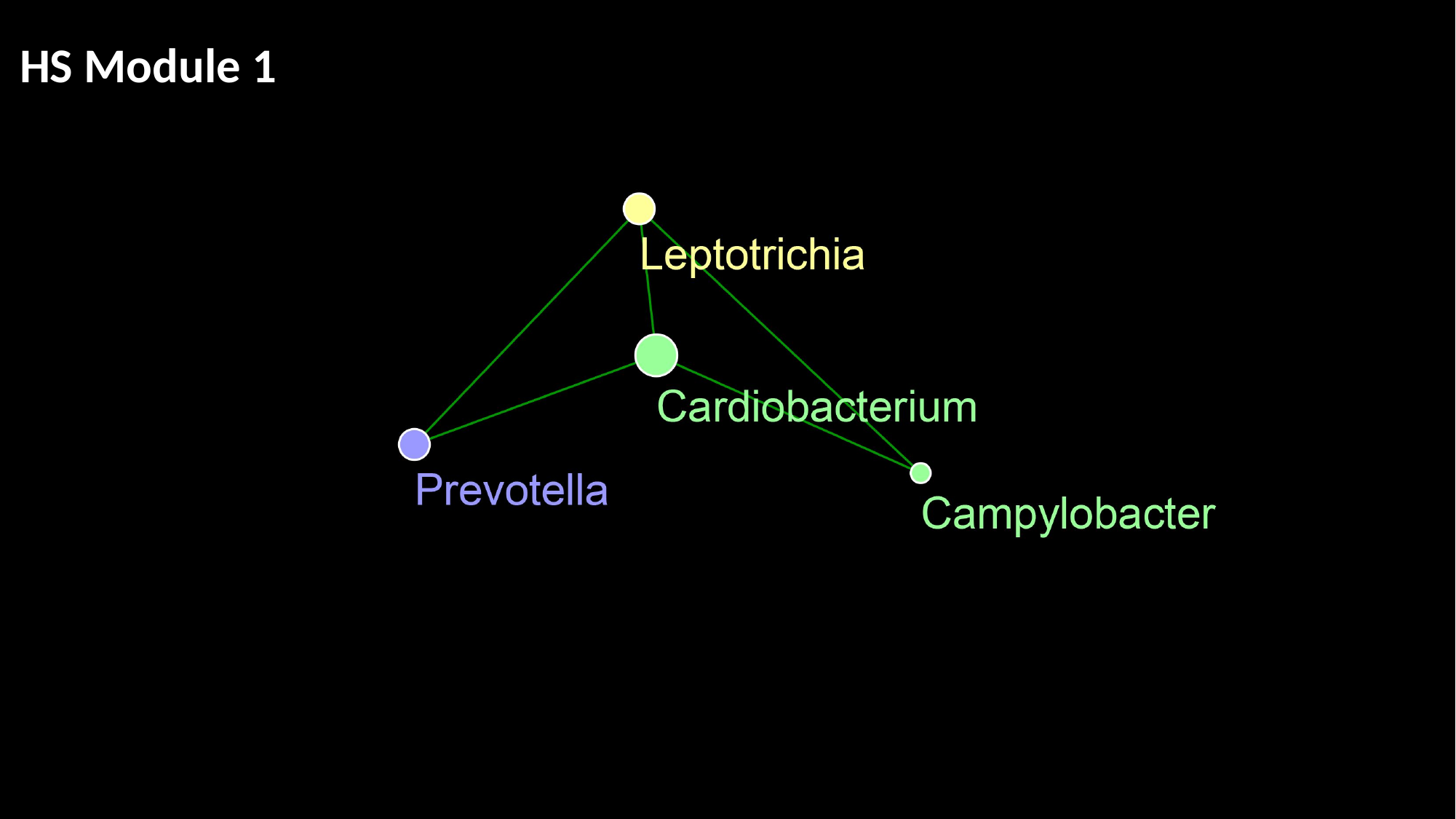

HS Module 1

## Slide 90
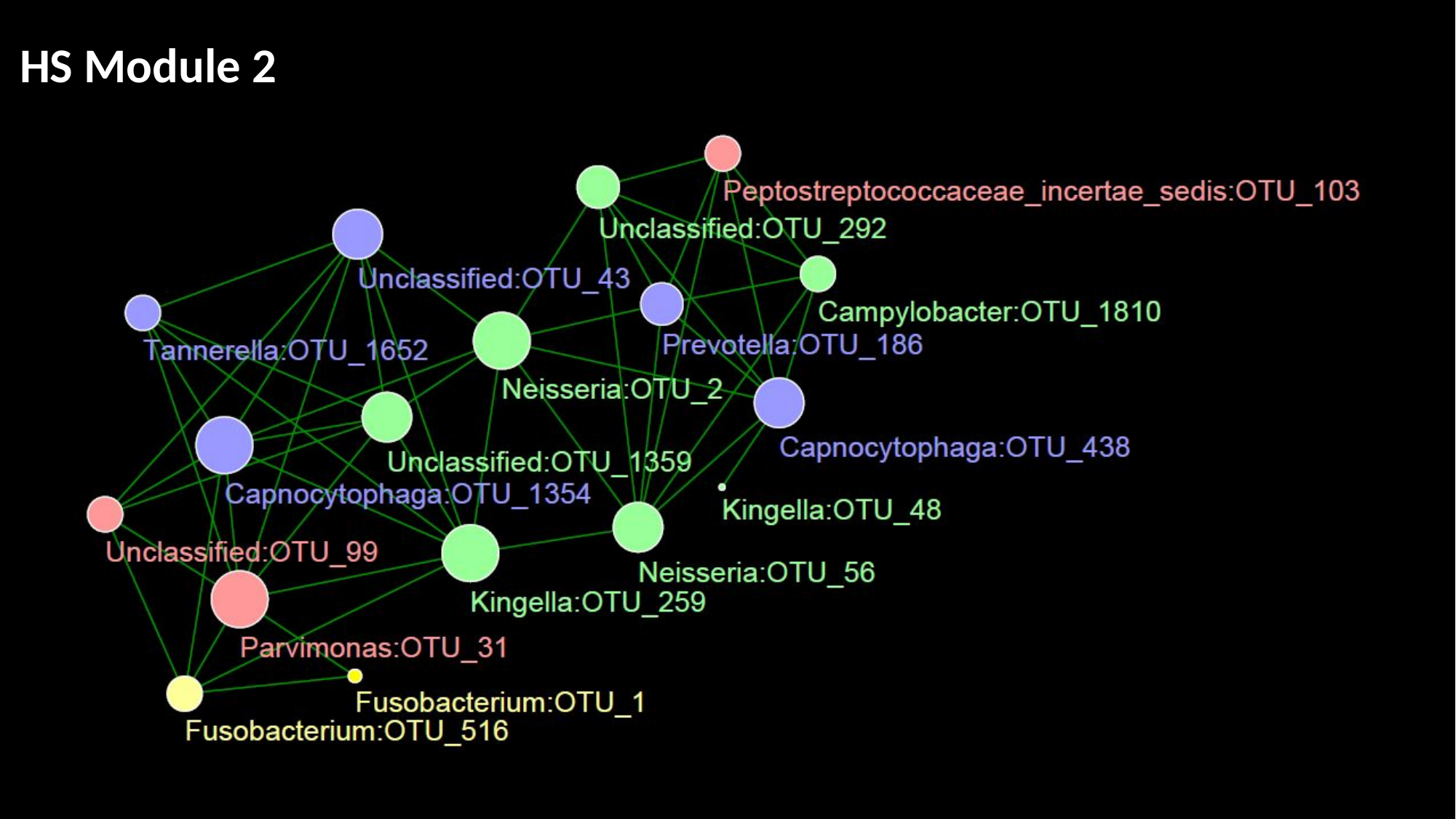

HS Module 2

## Slide 91
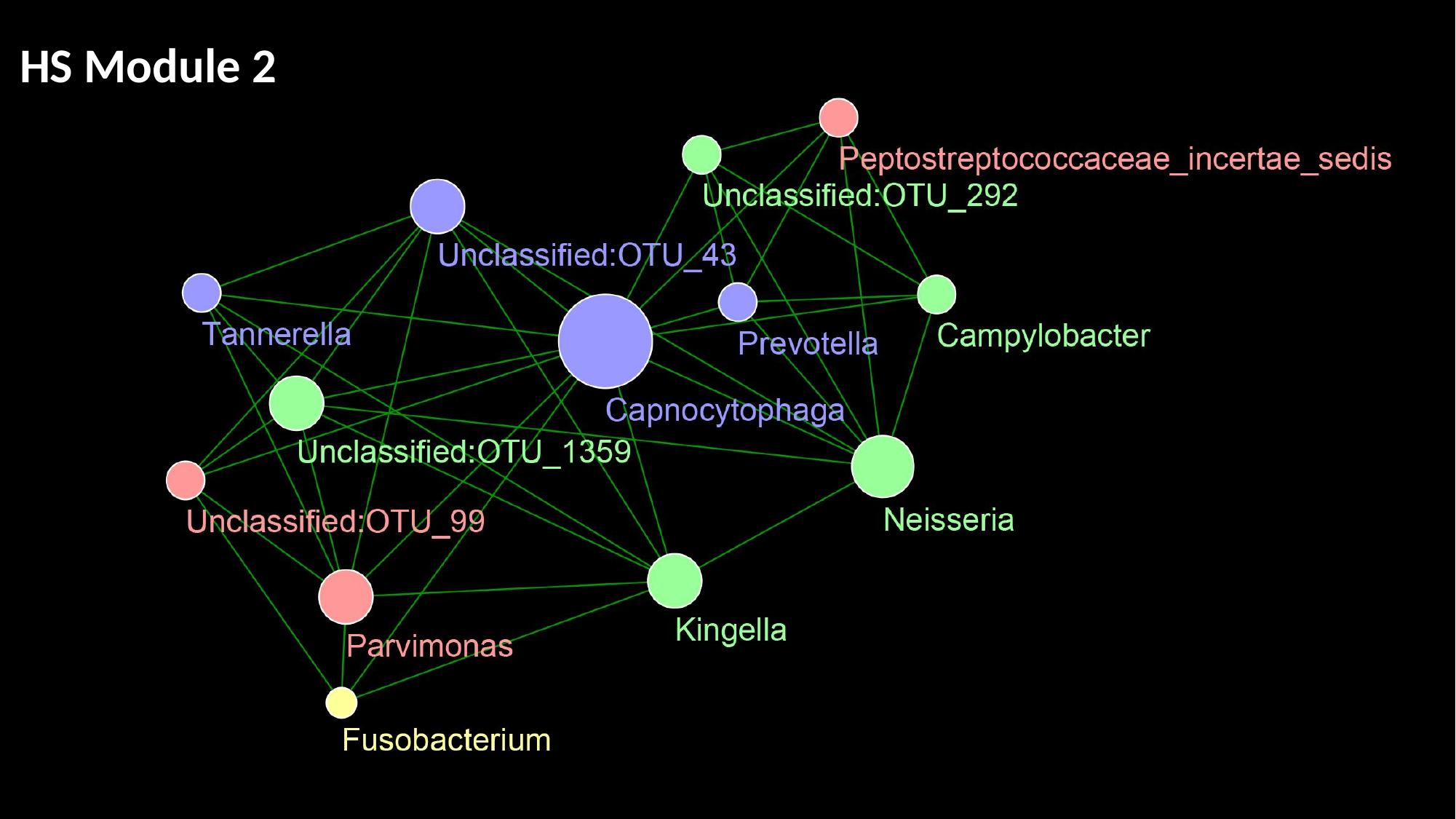

HS Module 2

## Slide 92
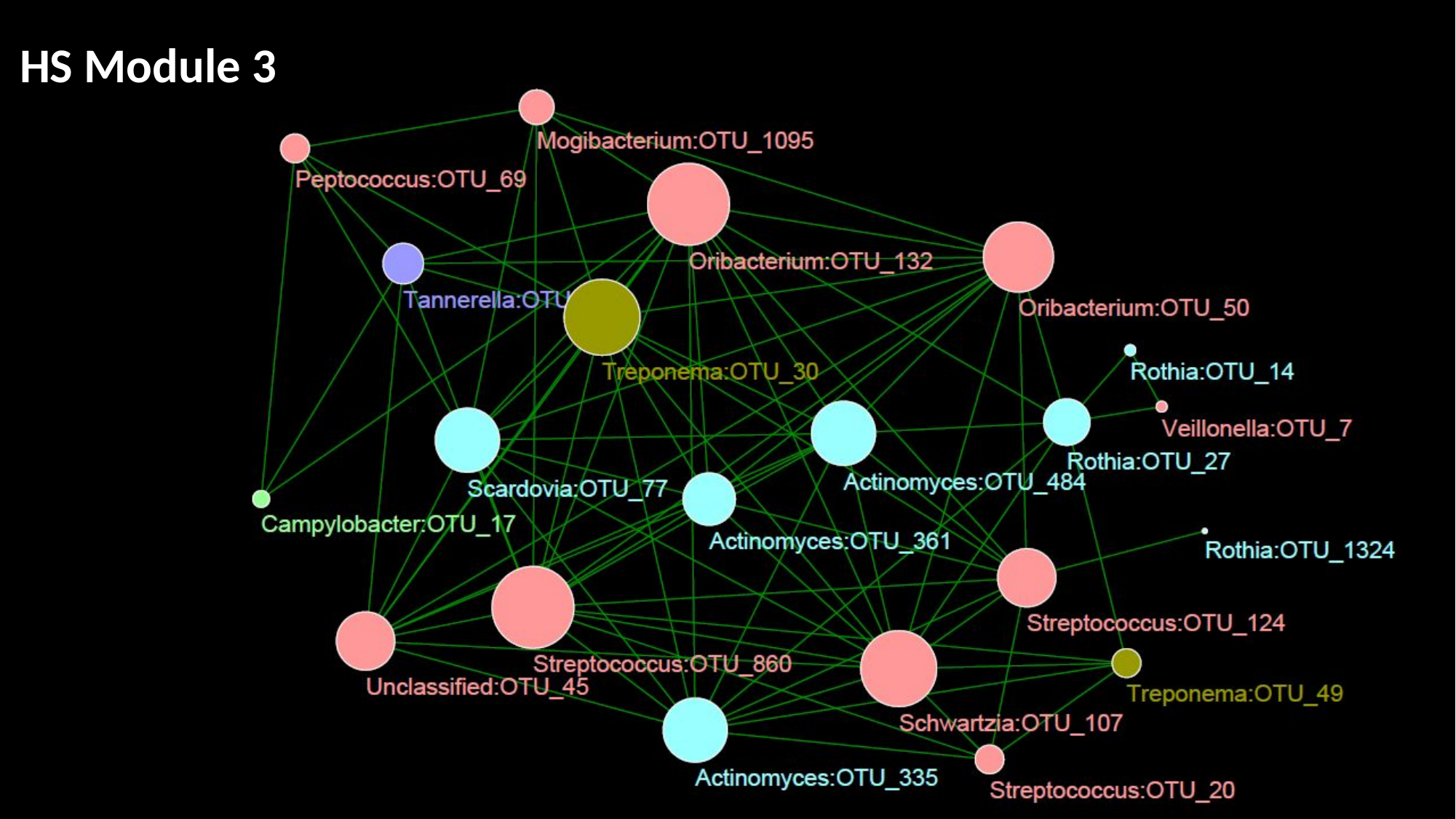

HS Module 3

## Slide 93
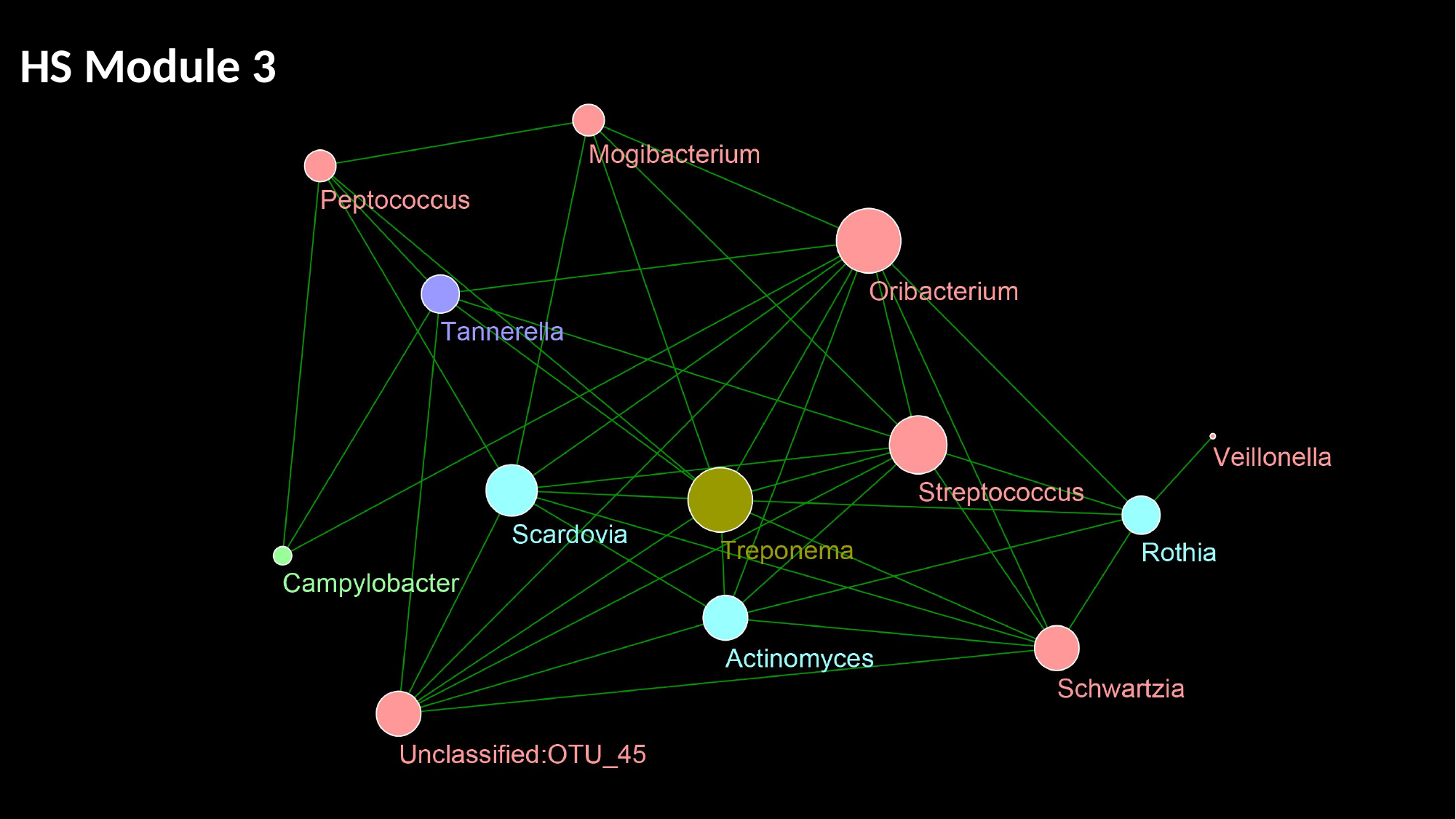

HS Module 3

## Slide 94
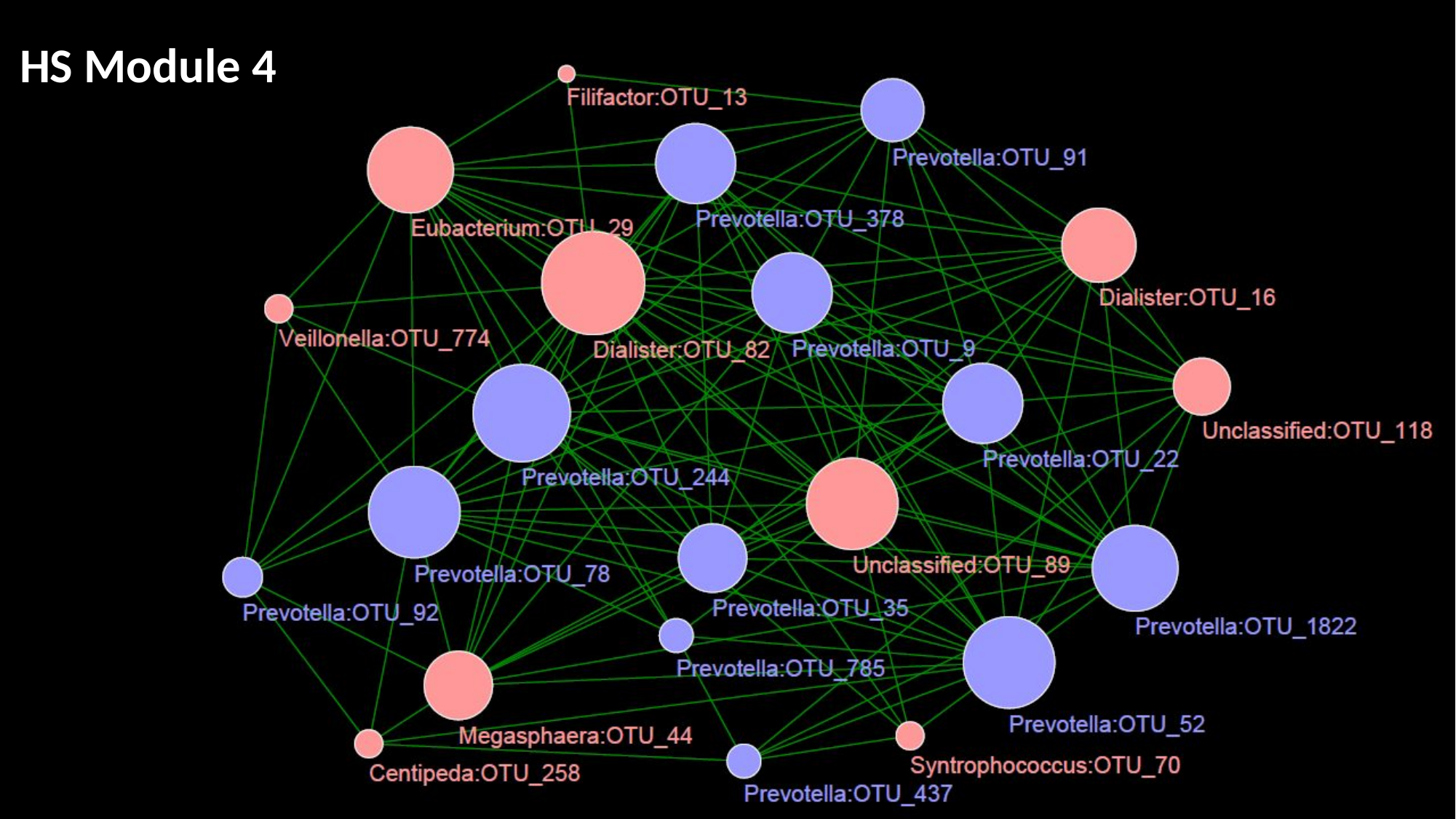

HS Module 4

## Slide 95
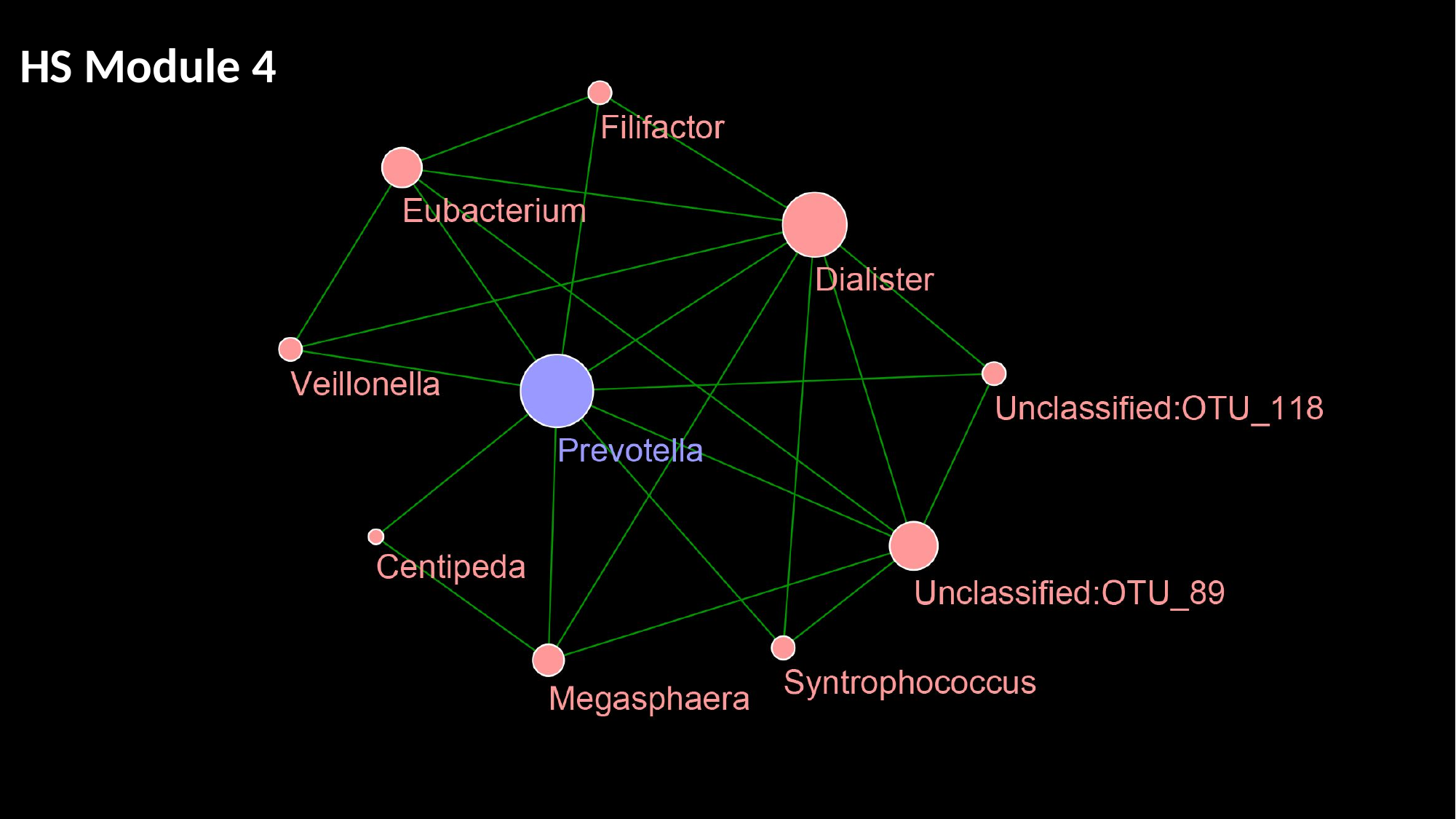

HS Module 4

## Slide 96
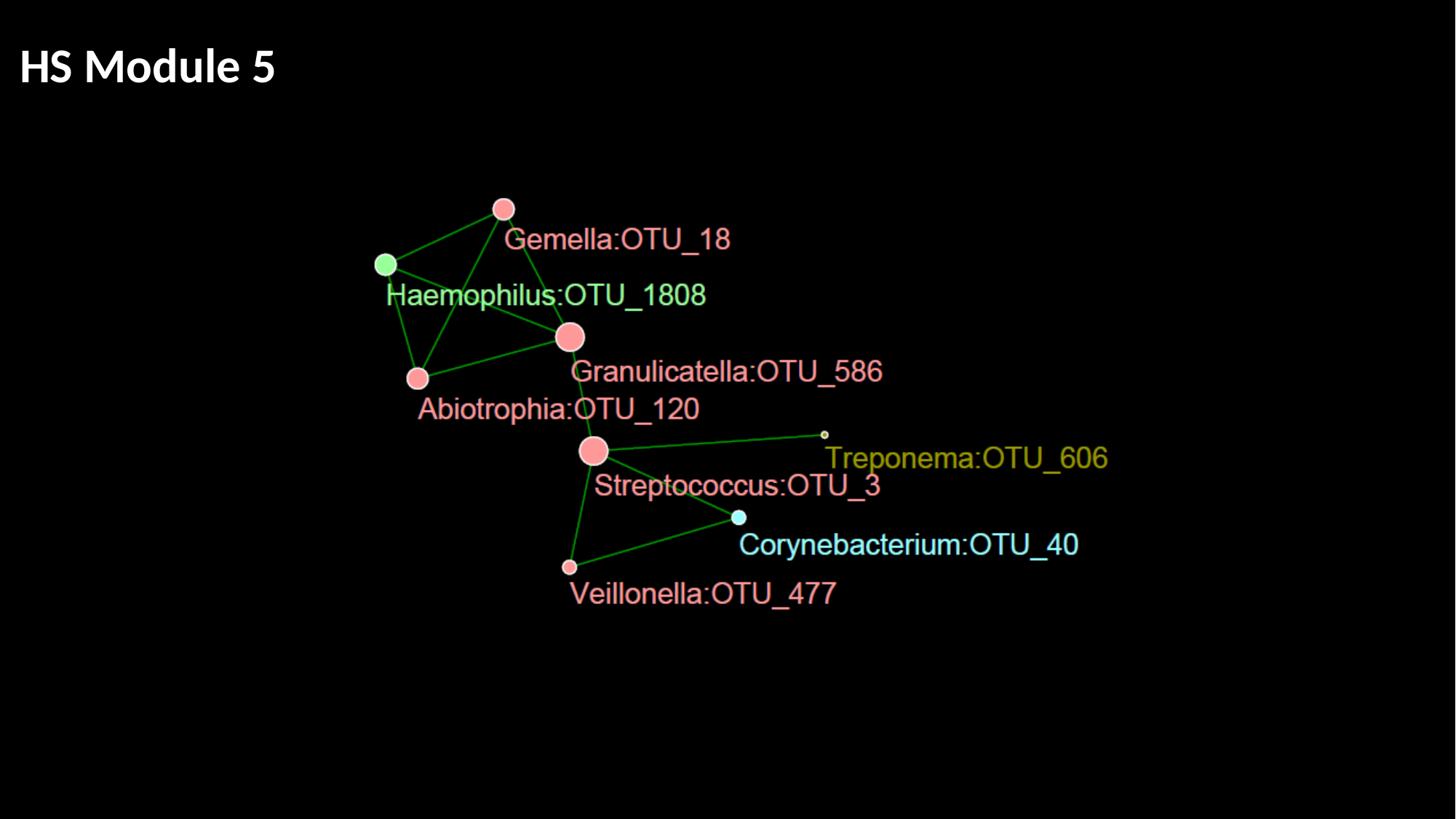

HS Module 5

## Slide 97
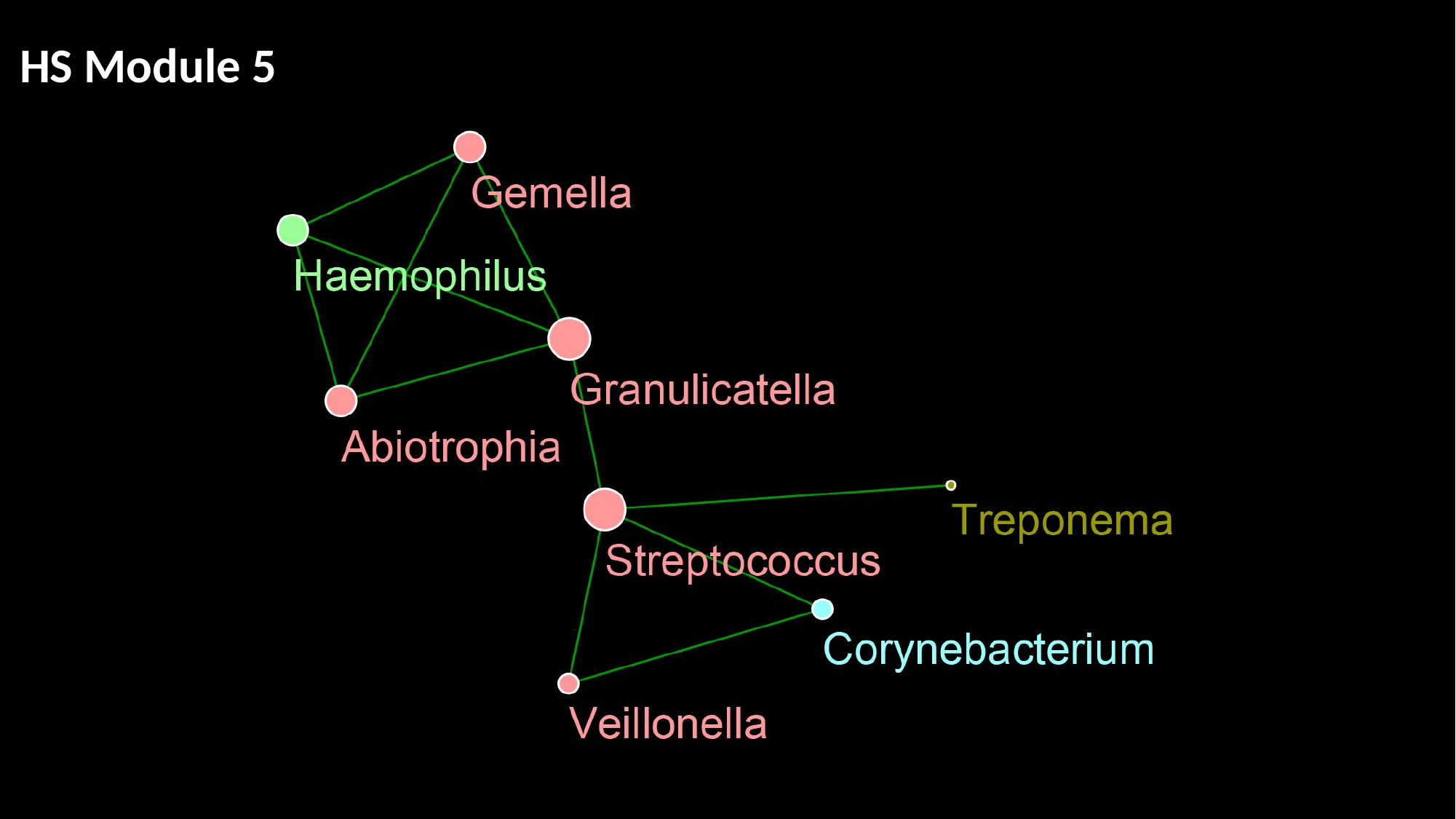

HS Module 5

## Slide 98
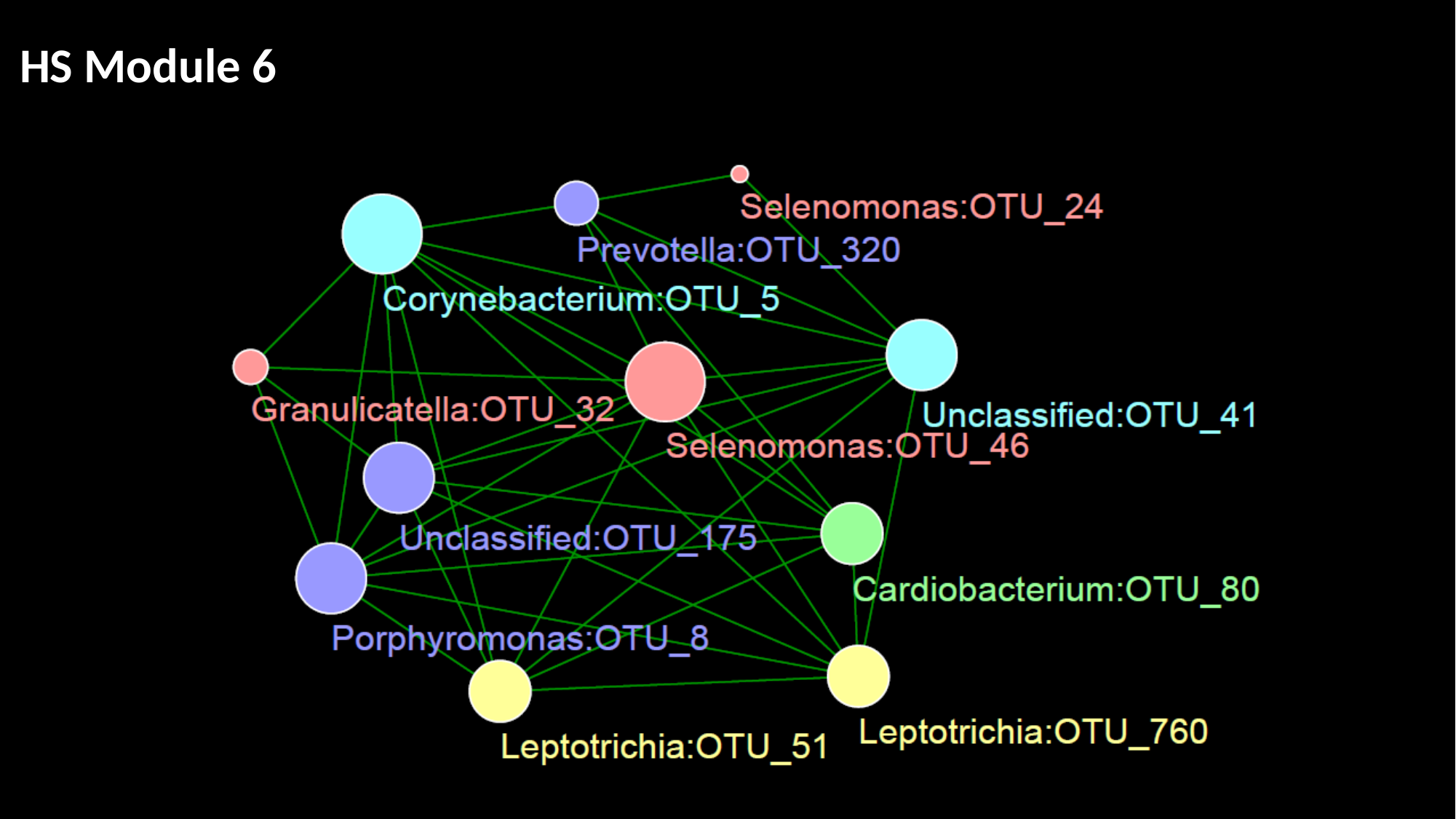

HS Module 6

## Slide 99
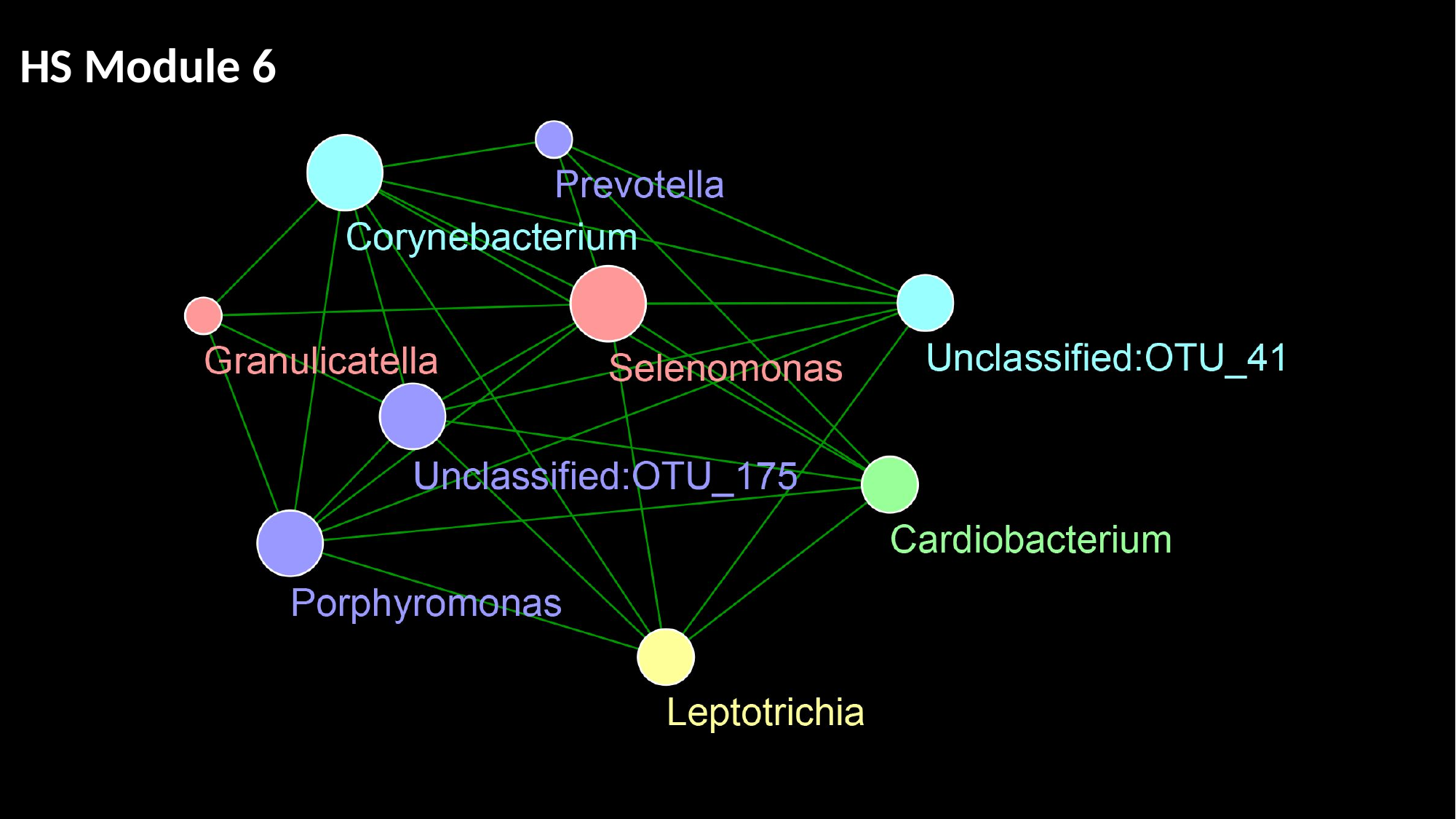

HS Module 6

## Slide 100
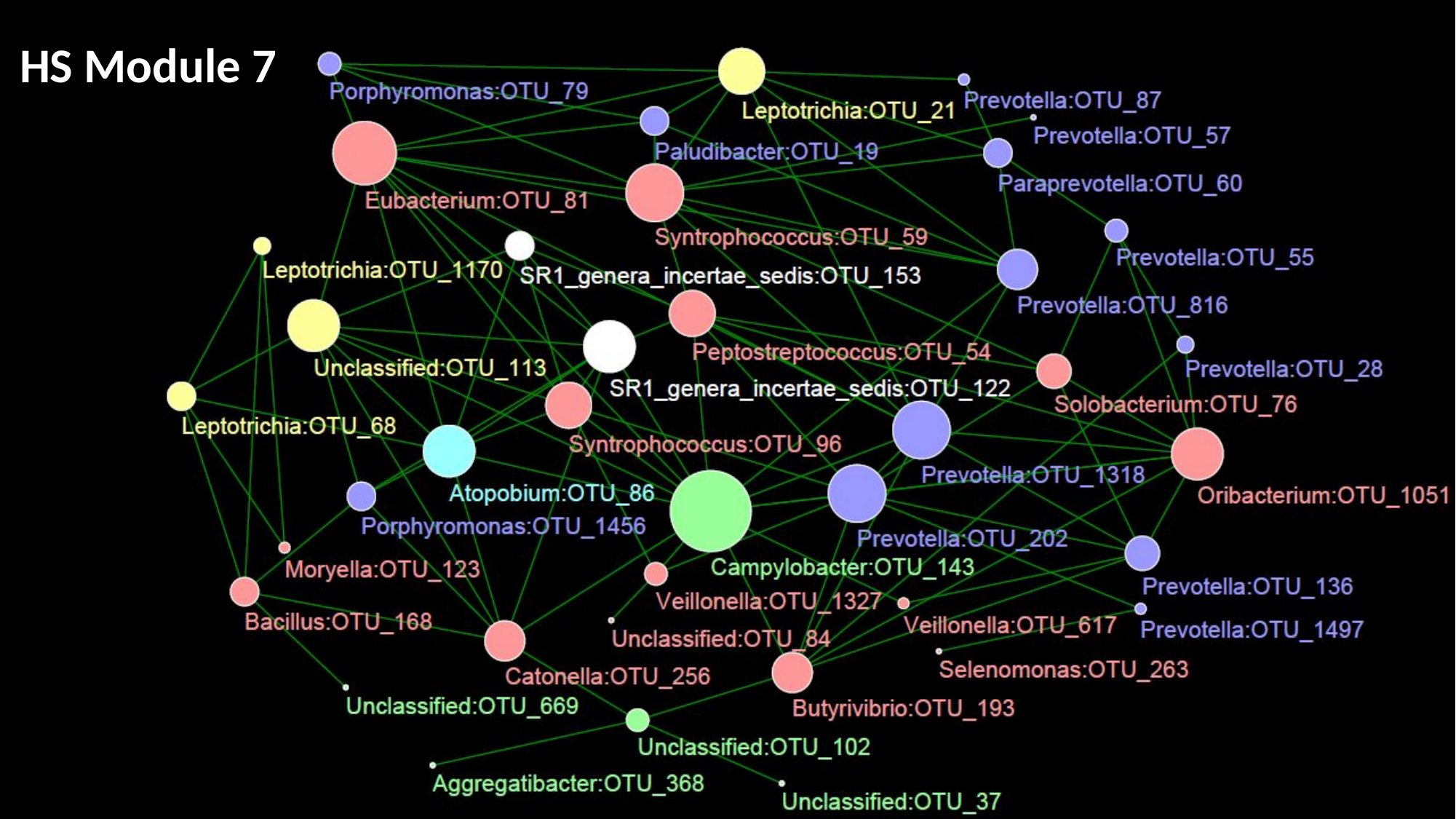

HS Module 7

## Slide 101
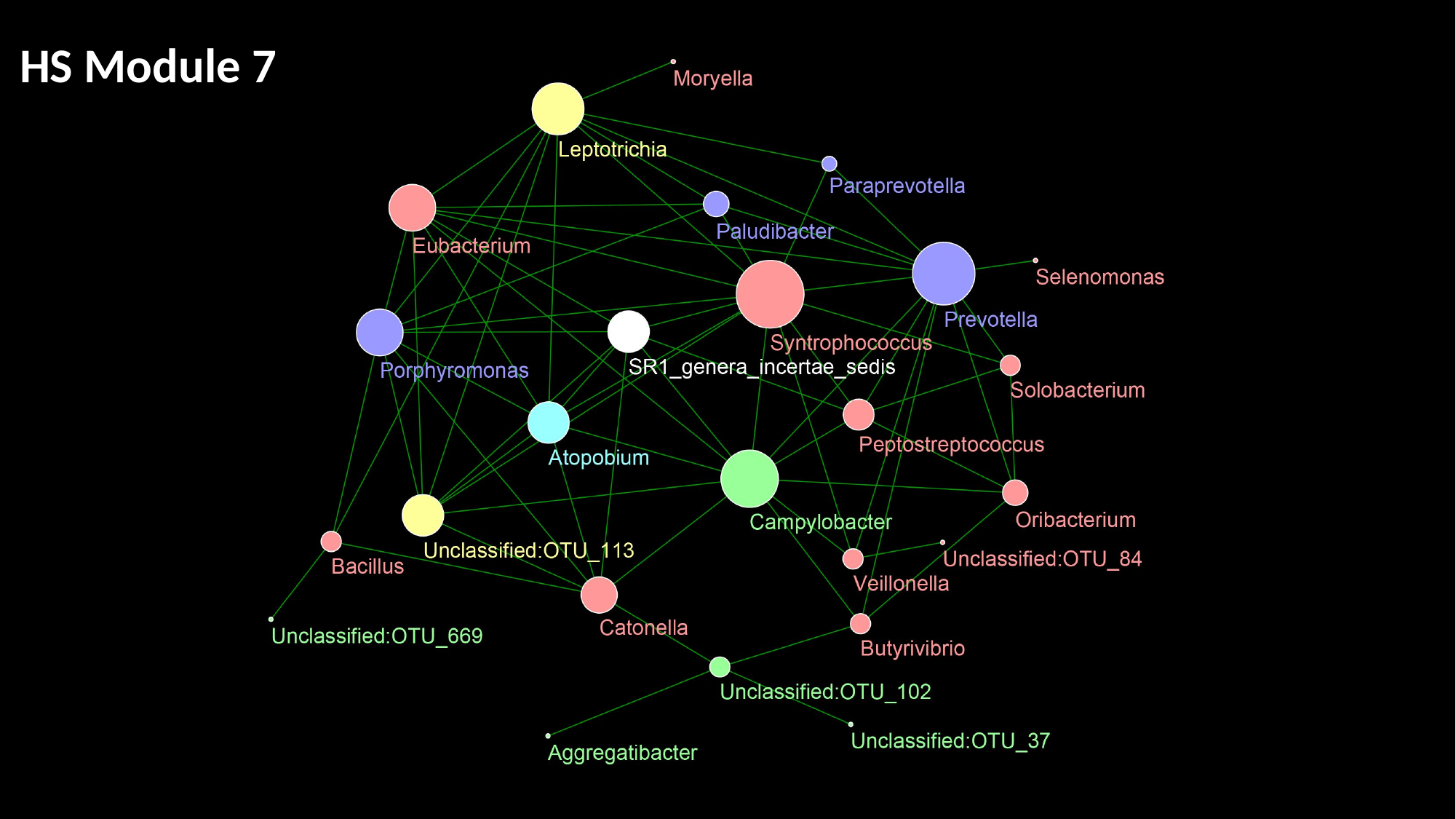

HS Module 7

## Slide 102
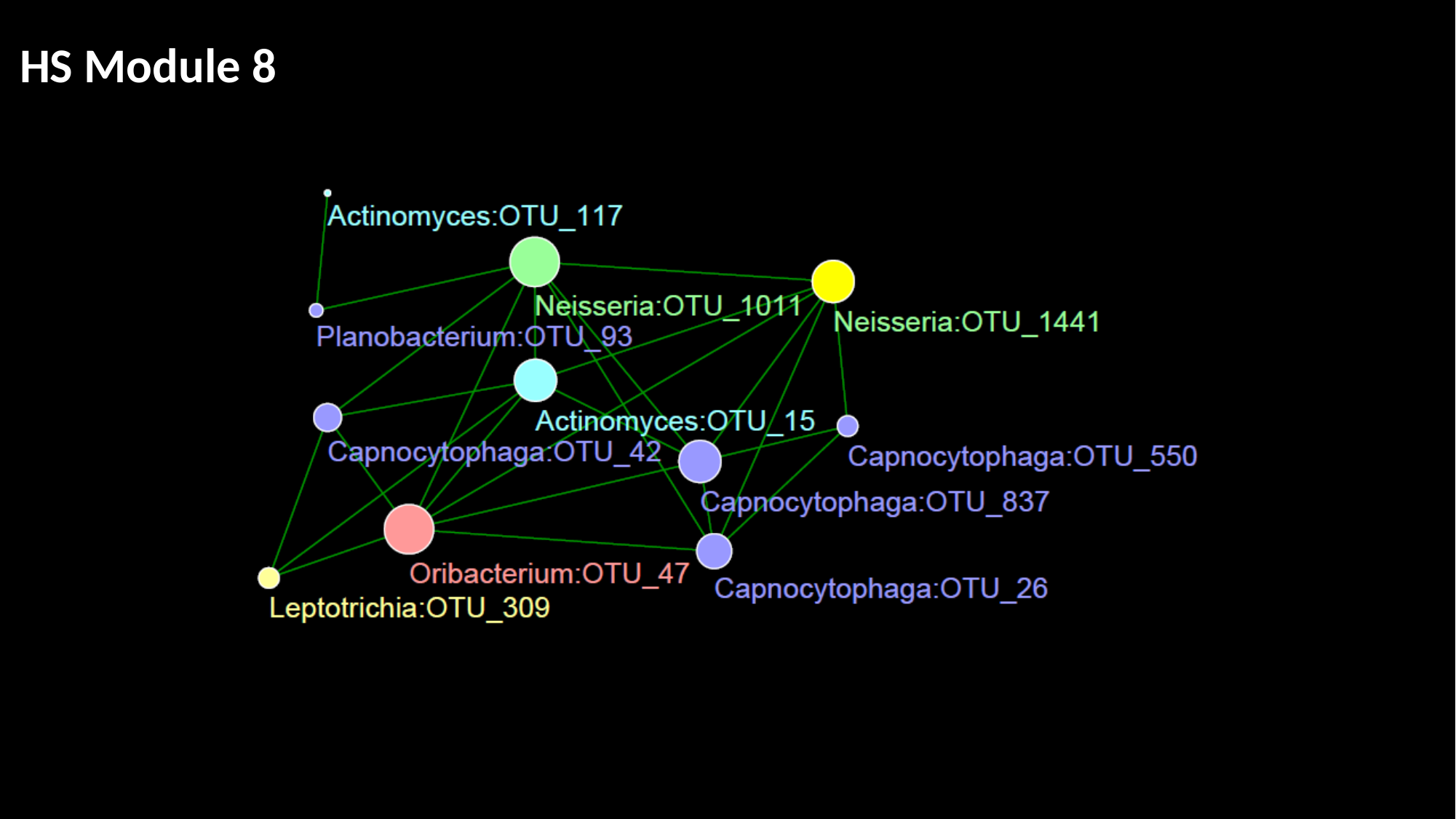

HS Module 8

## Slide 103
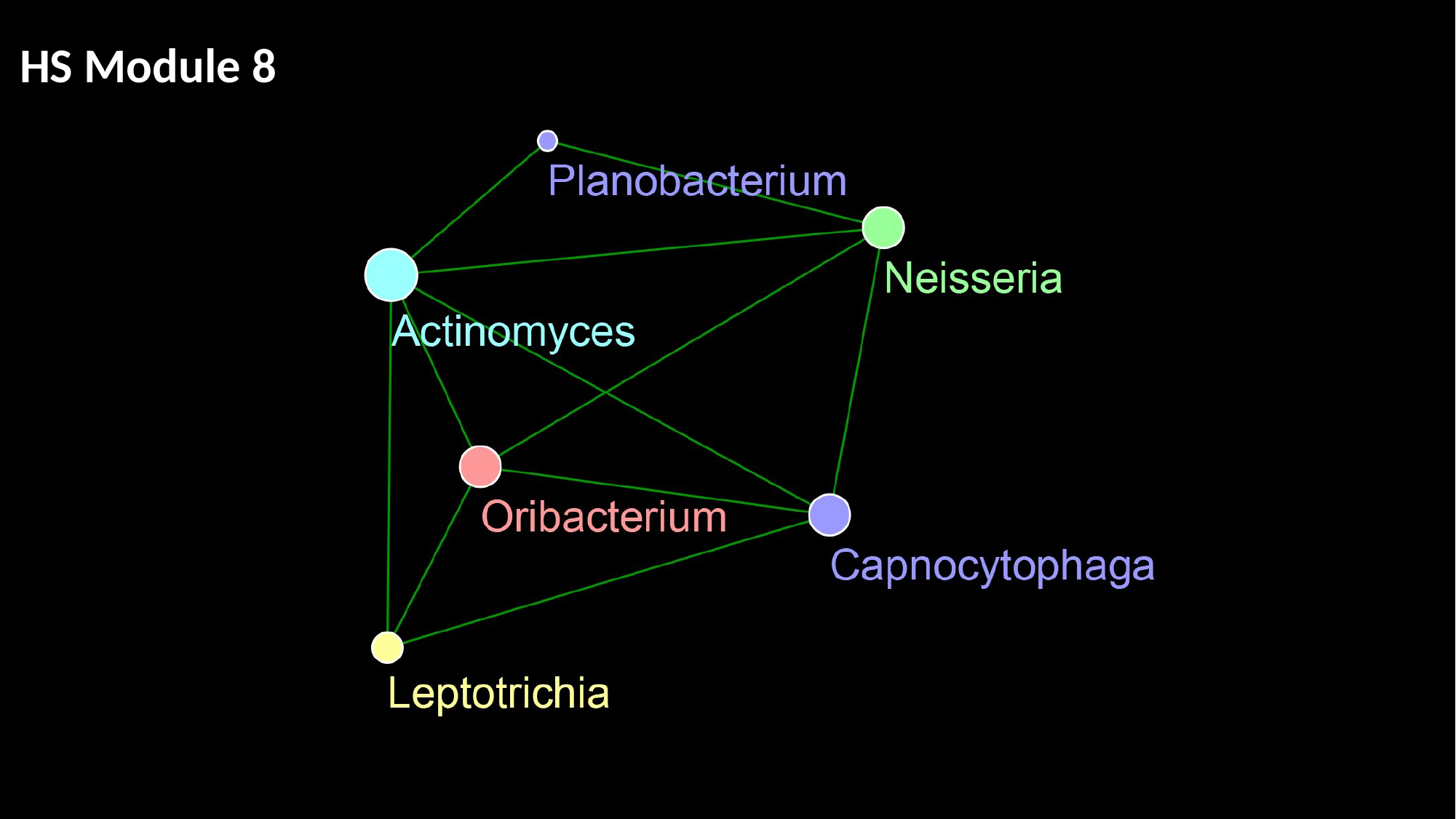

HS Module 8

## Slide 104
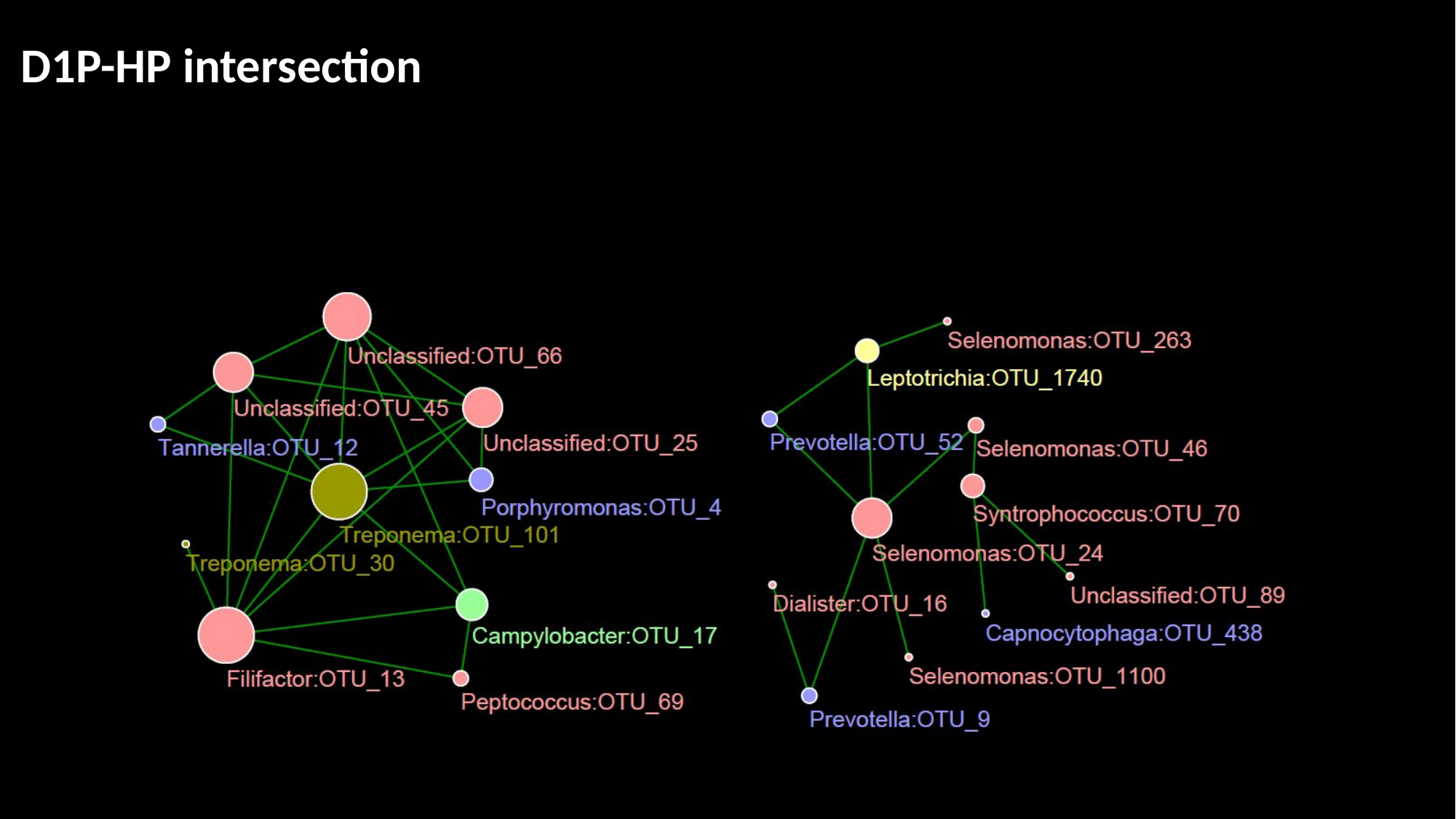

D1P-HP intersection

## Slide 105
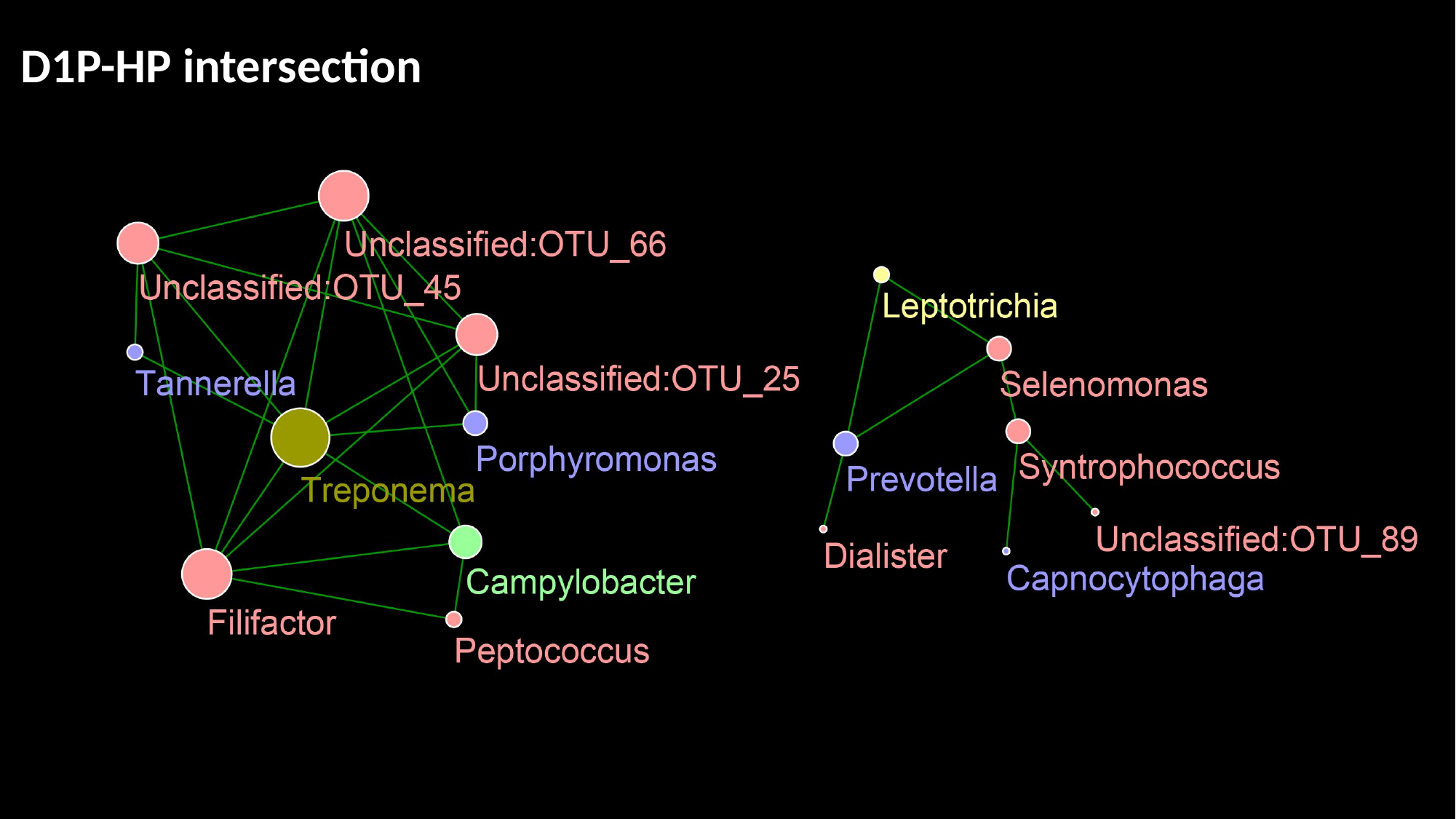

D1P-HP intersection

## Slide 106
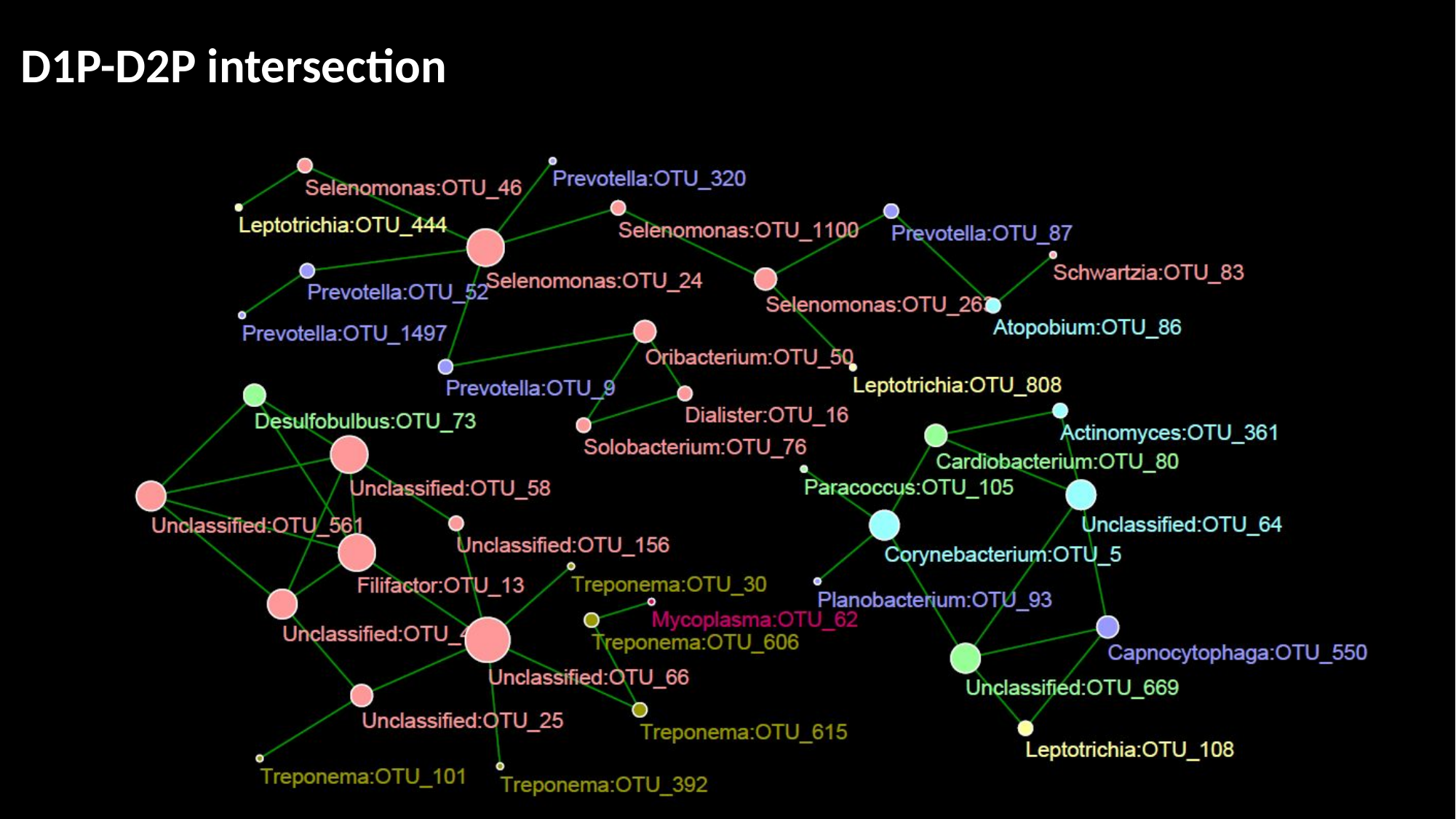

D1P-D2P intersection

## Slide 107
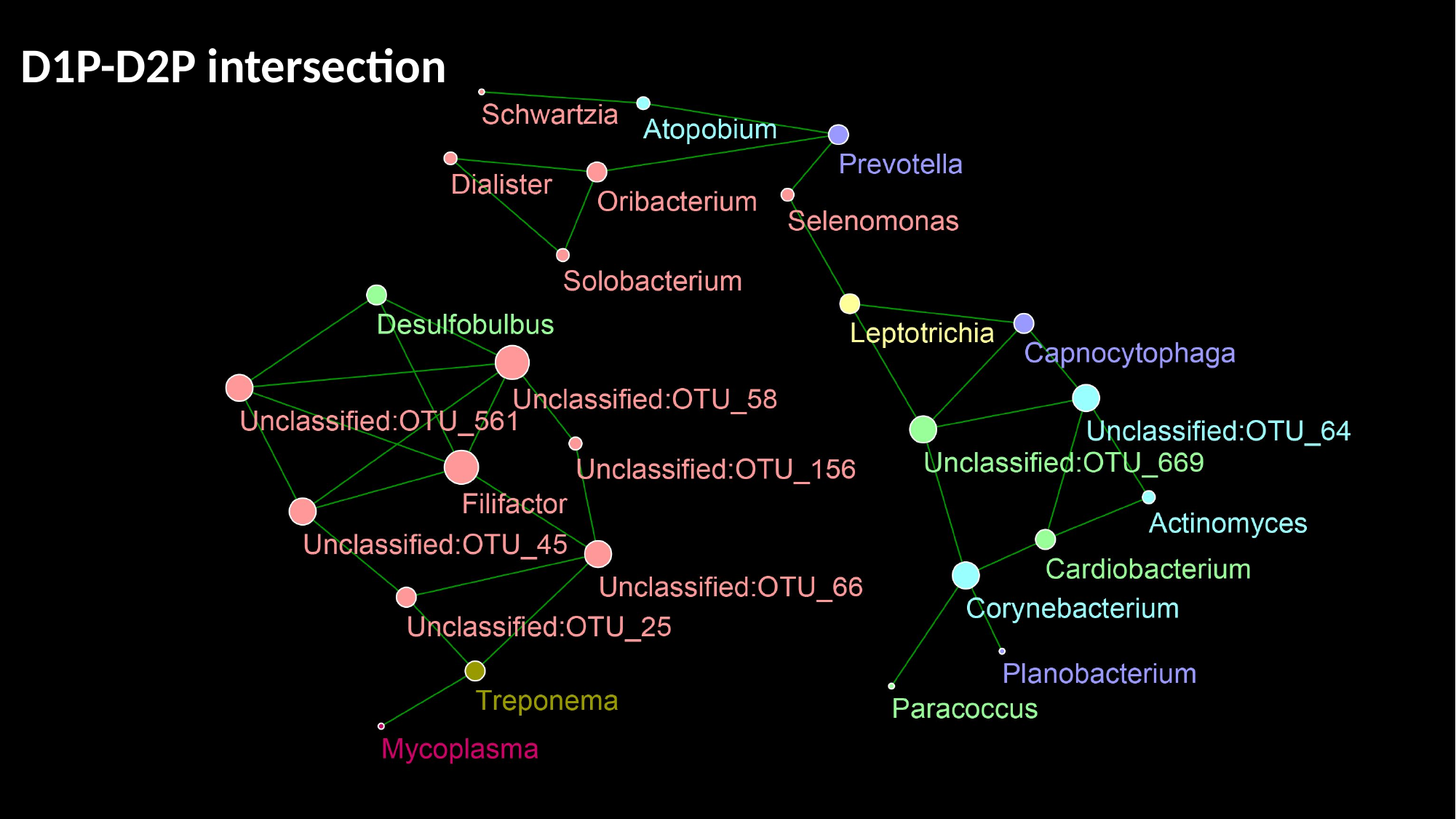

D1P-D2P intersection

## Slide 108
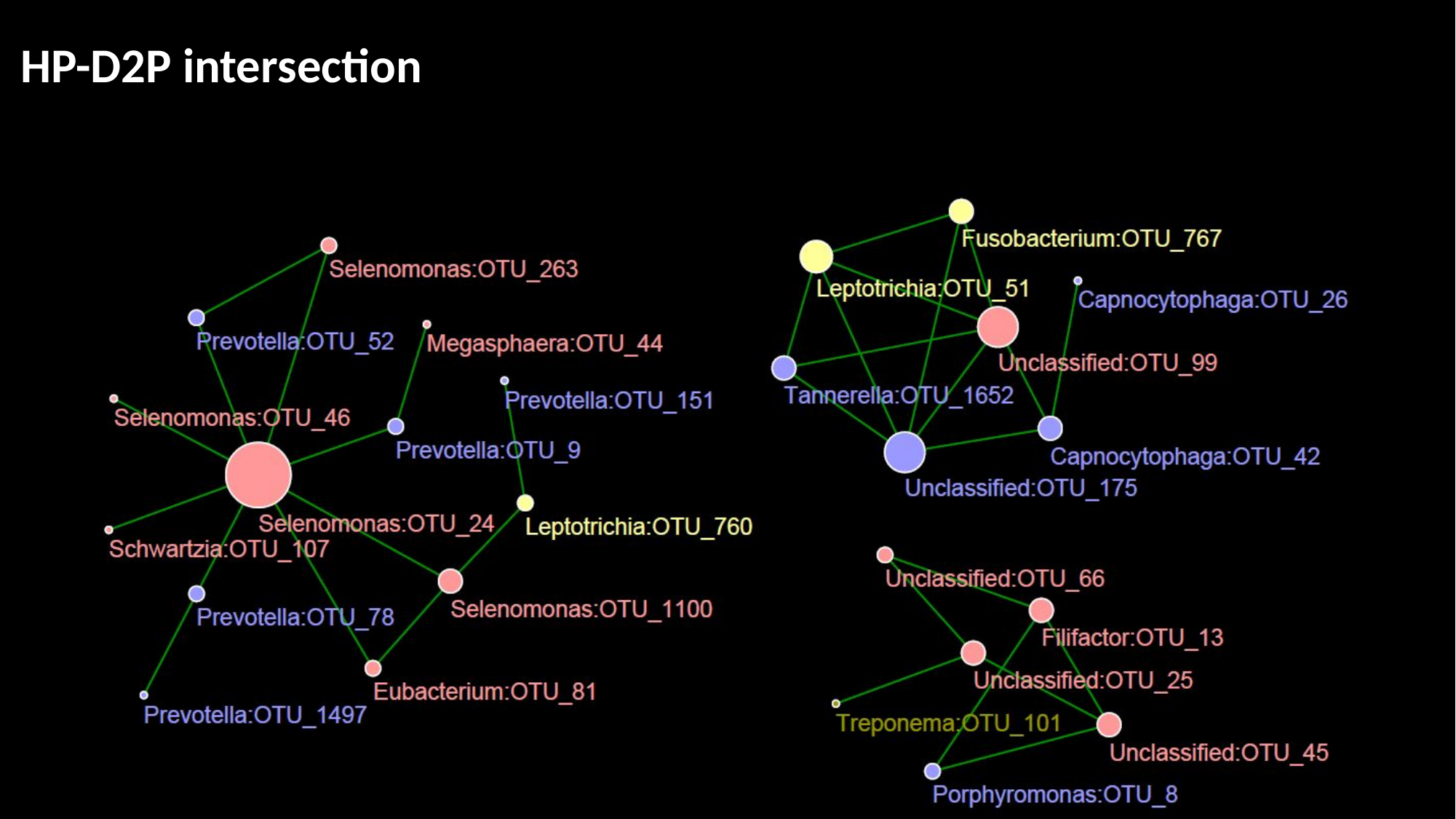

HP-D2P intersection

## Slide 109
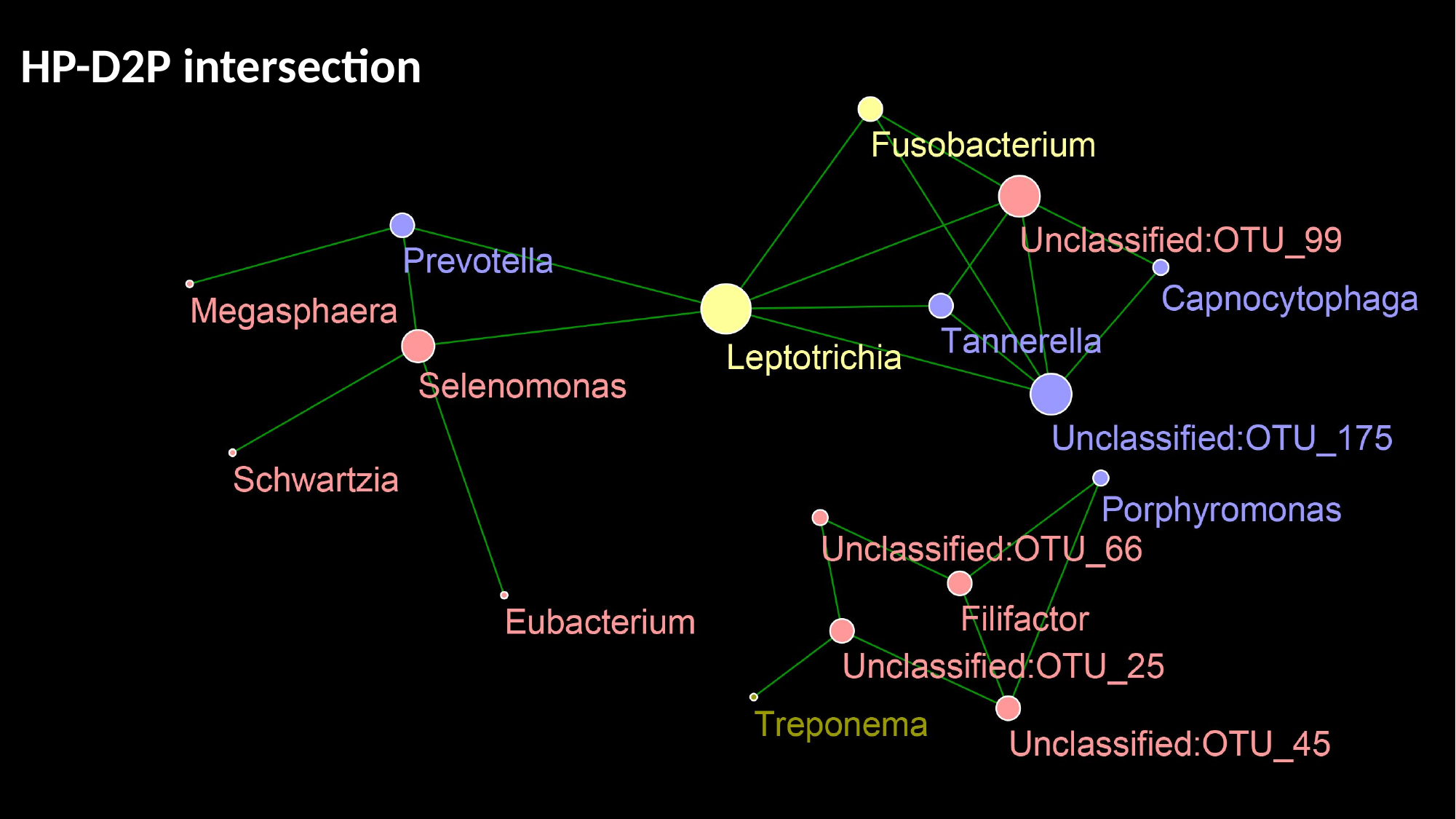

HP-D2P intersection

## Slide 110
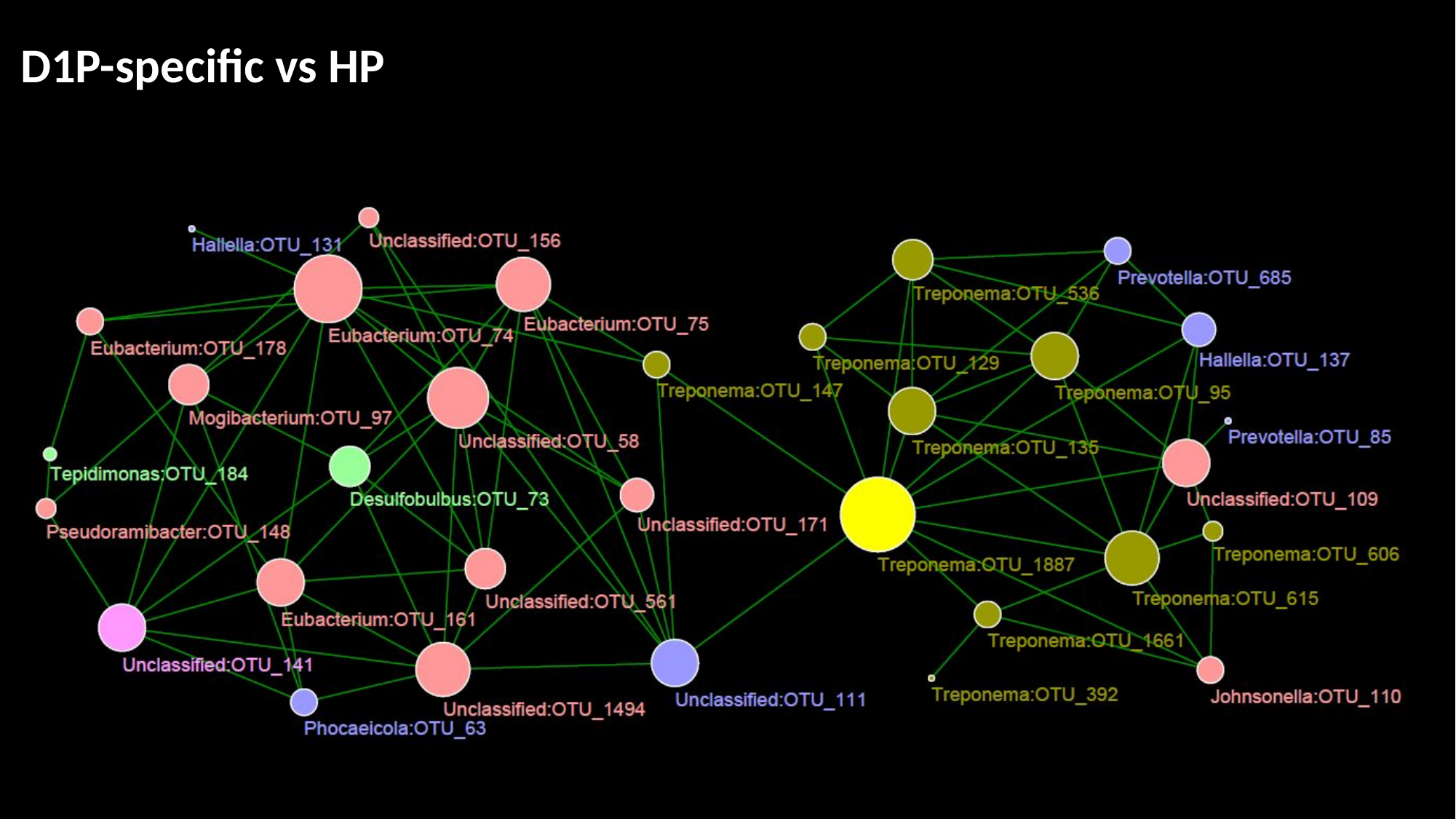

D1P-specific vs HP

## Slide 111
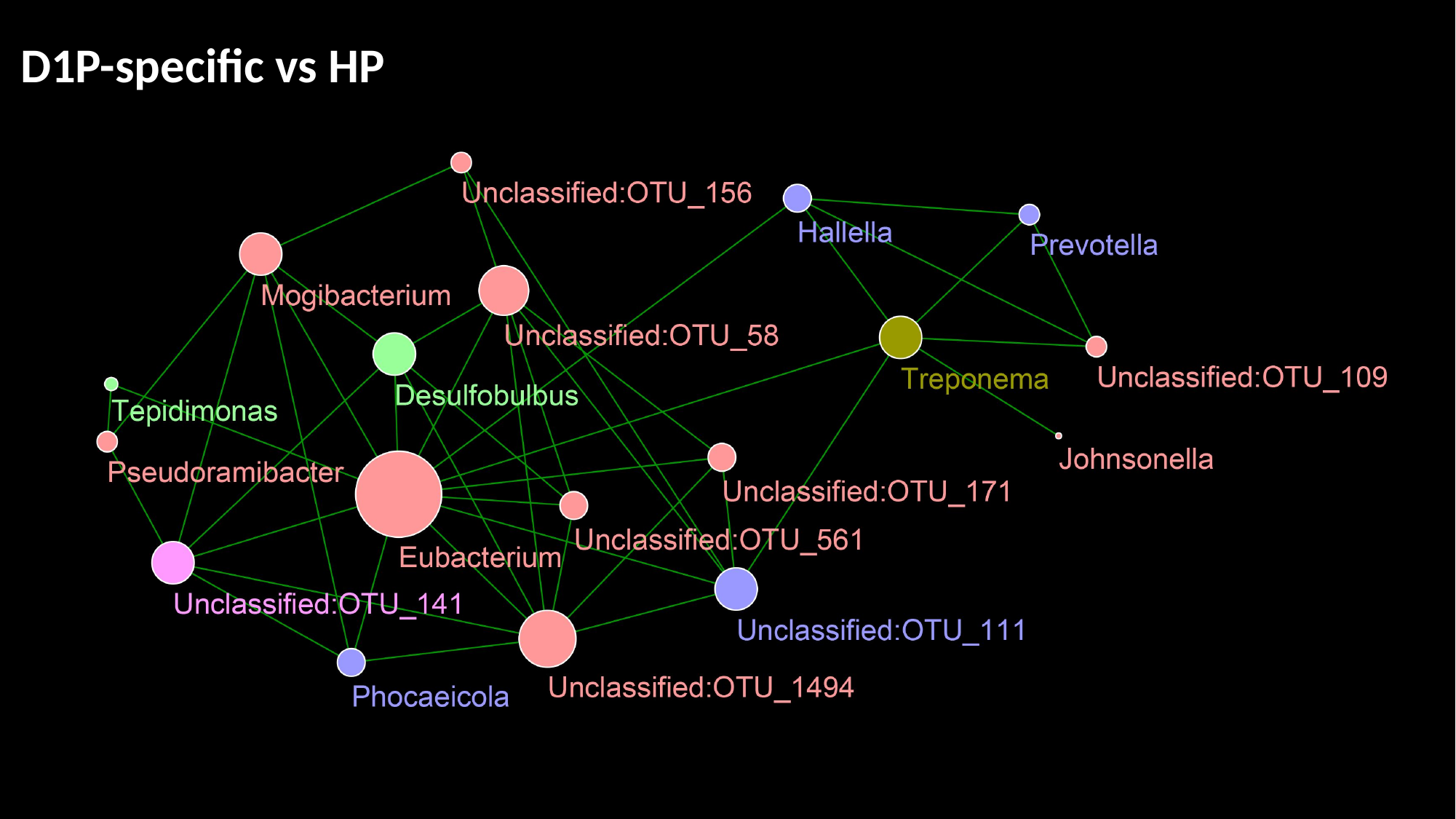

D1P-specific vs HP

## Slide 112
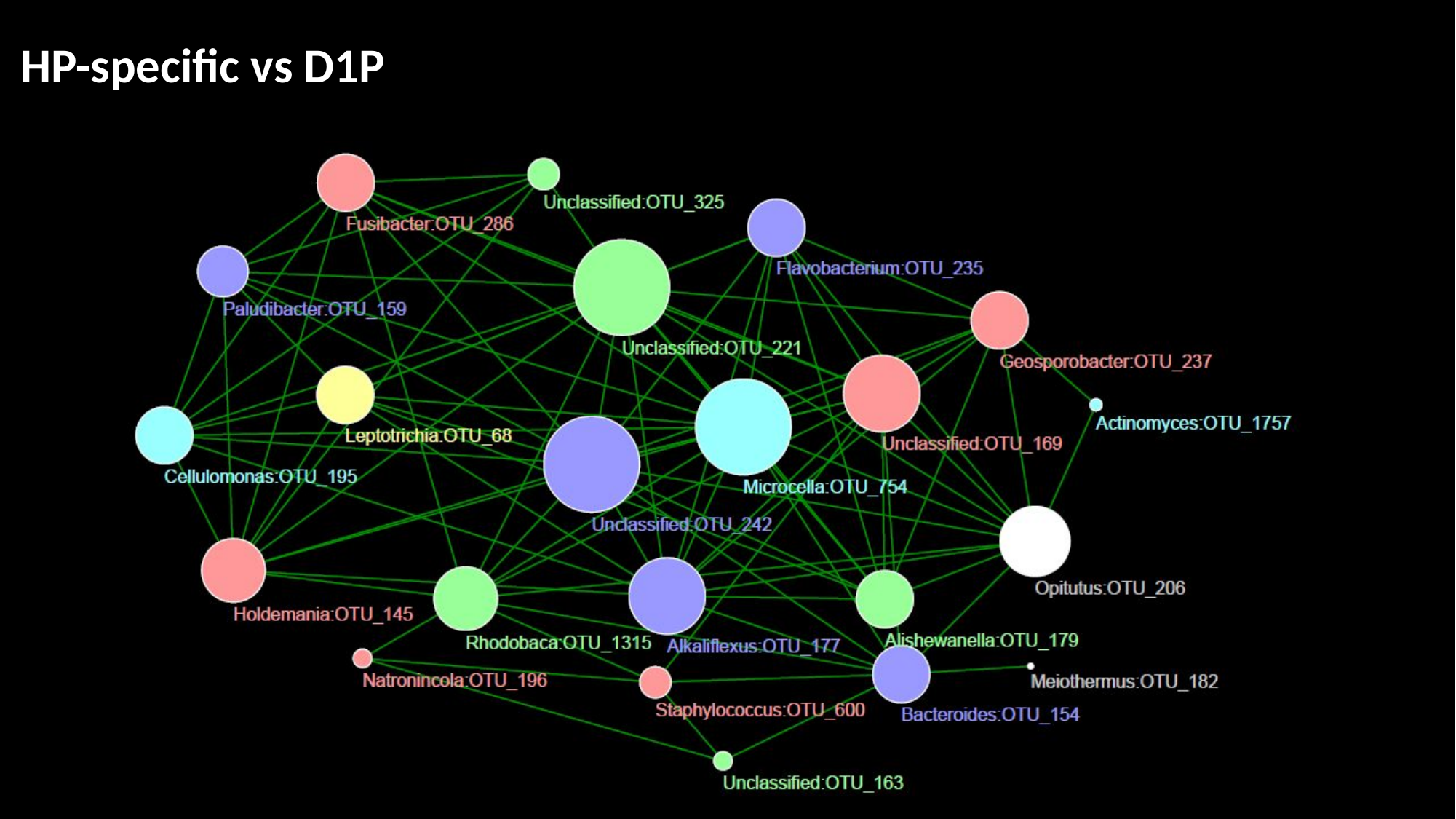

HP-specific vs D1P

## Slide 113
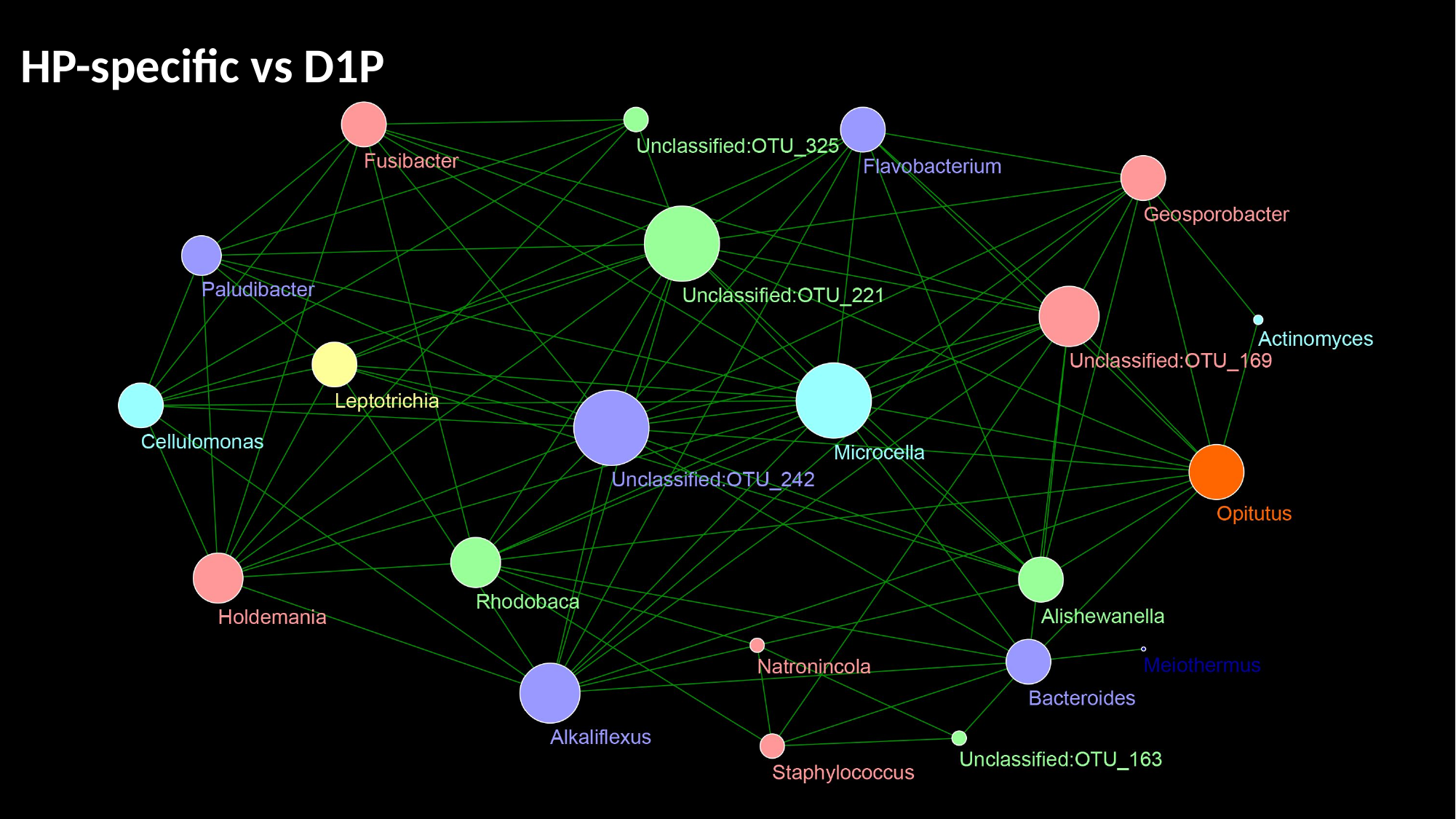

HP-specific vs D1P

## Slide 114
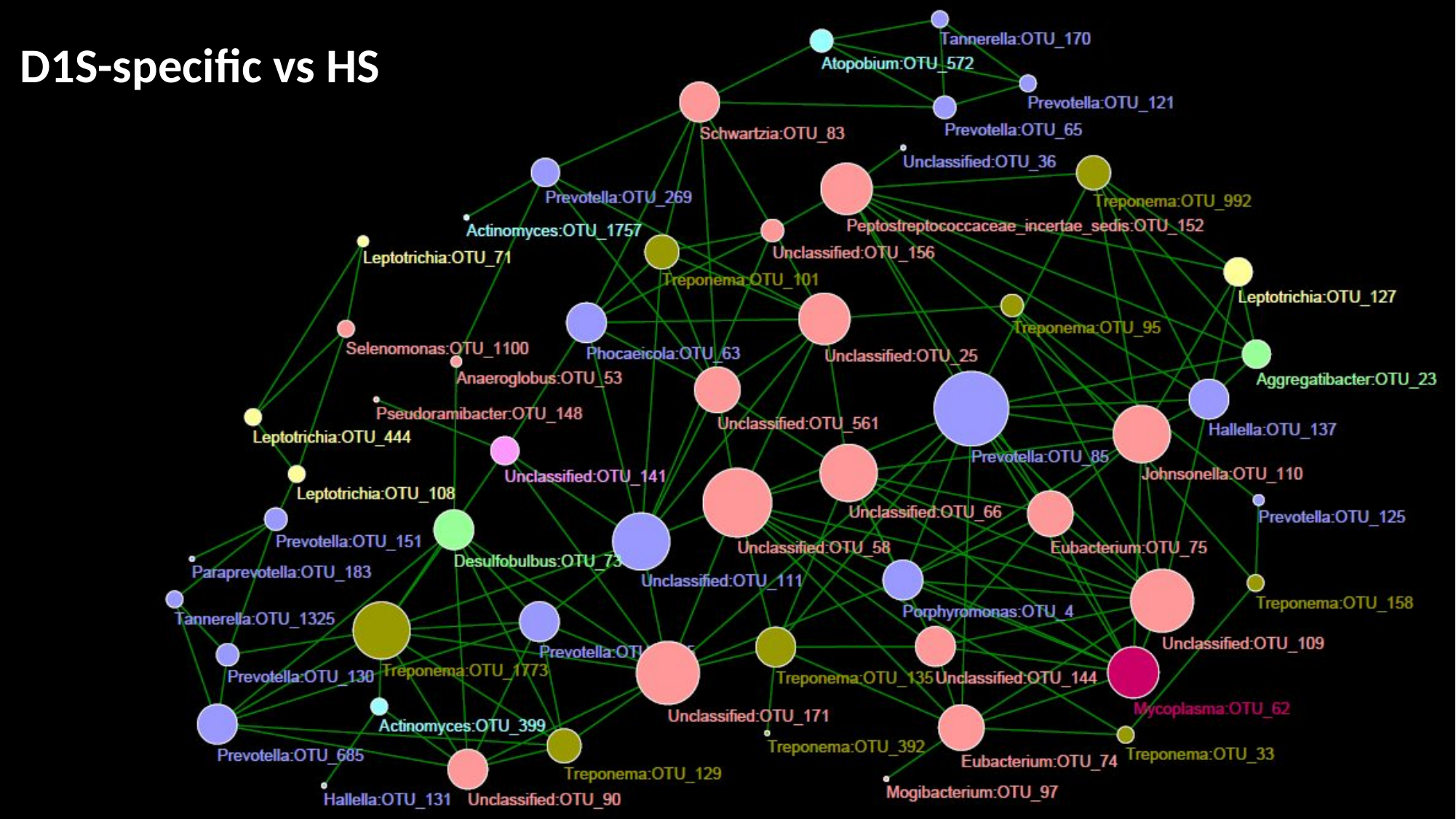

D1S-specific vs HS

## Slide 115
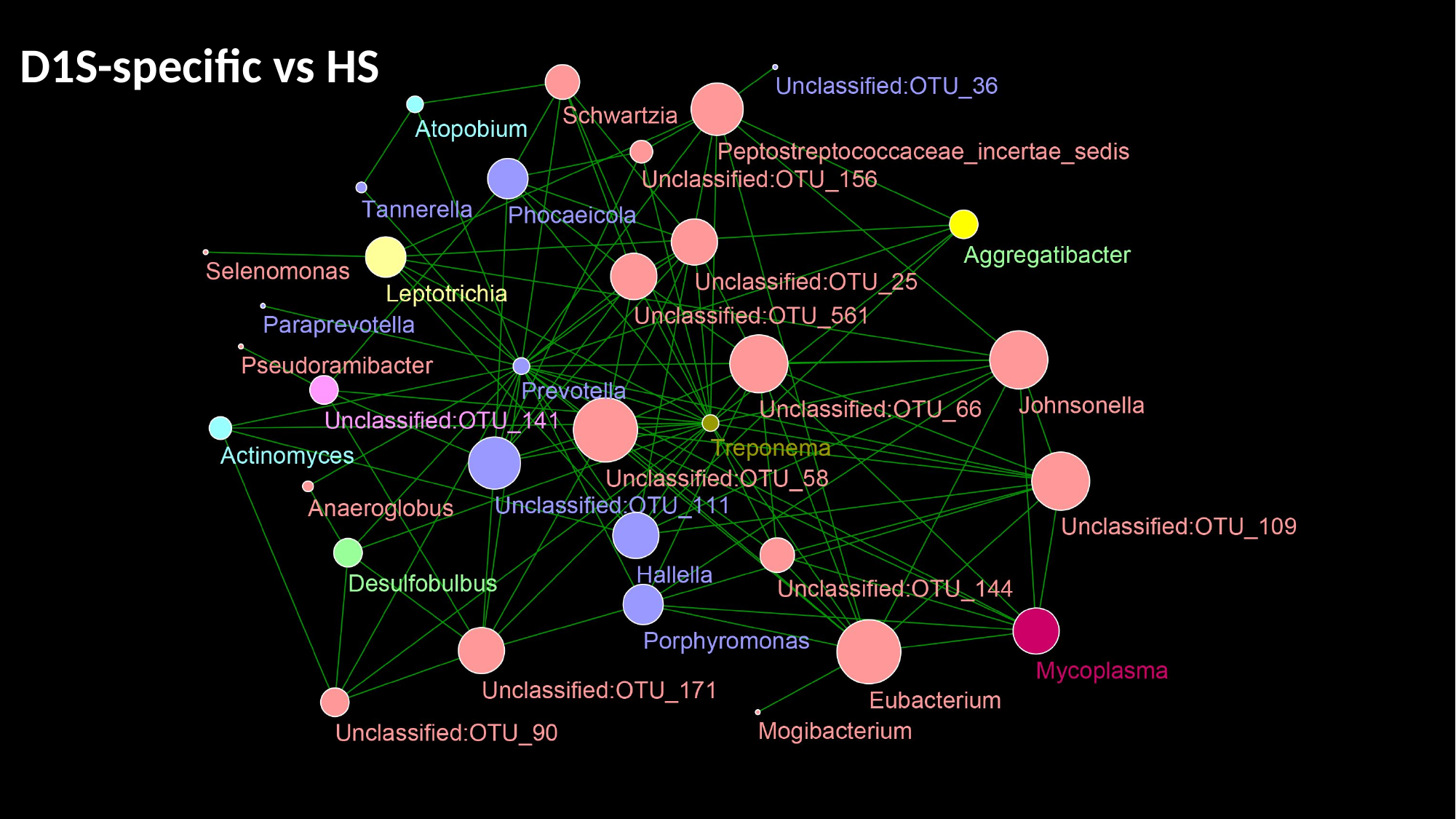

D1S-specific vs HS

## Slide 116
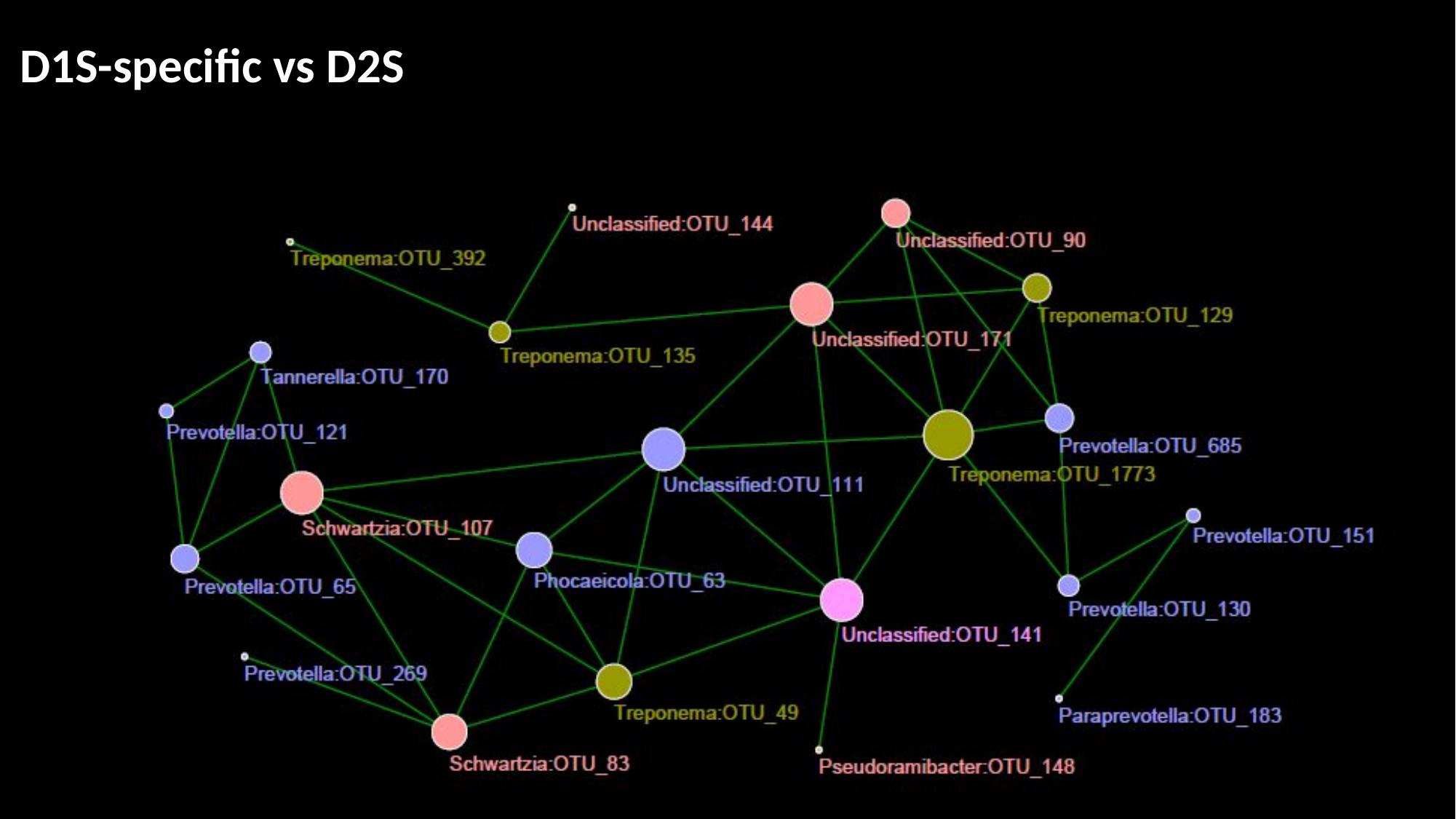

D1S-specific vs D2S

## Slide 117
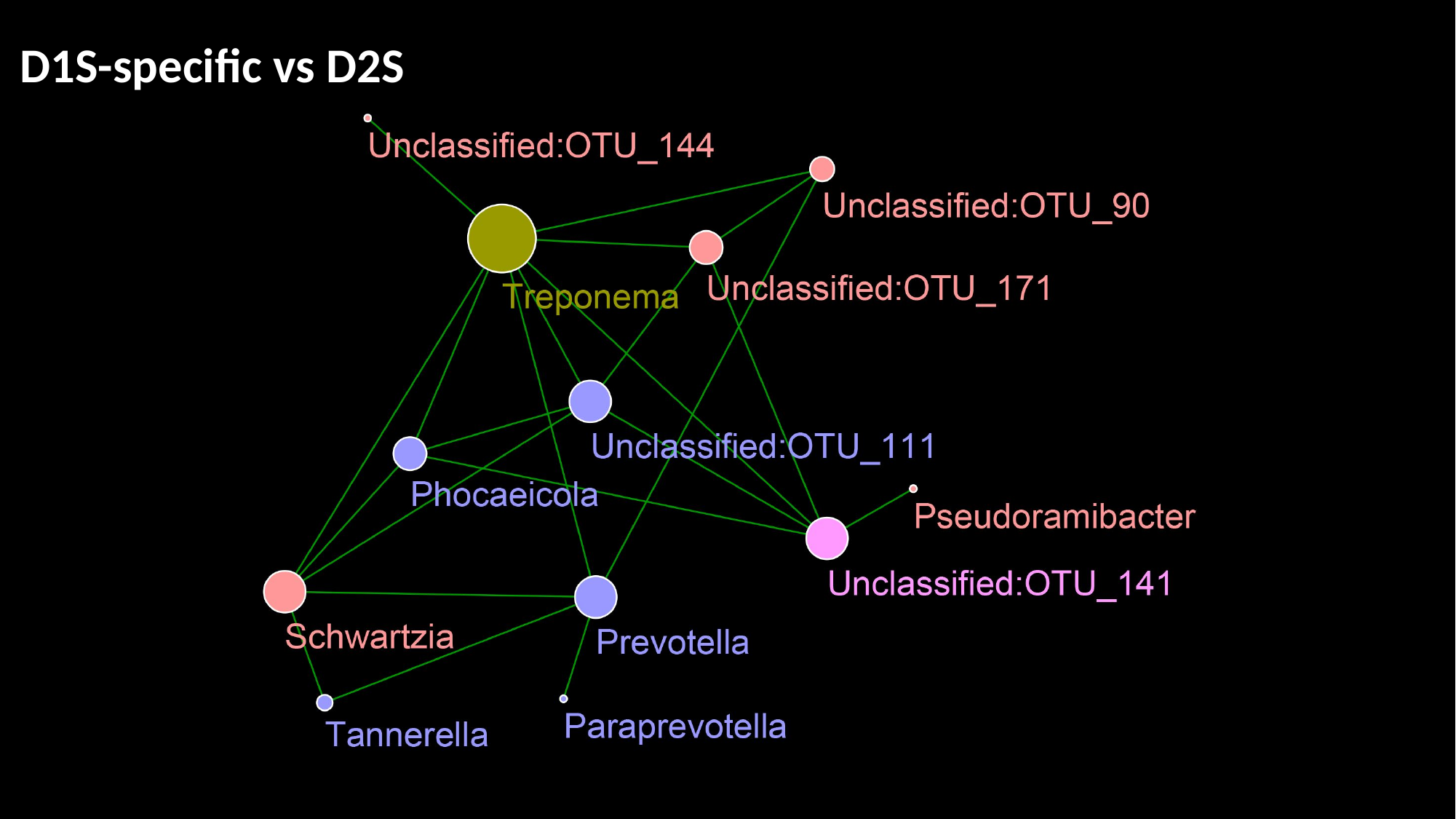

D1S-specific vs D2S

## Slide 118
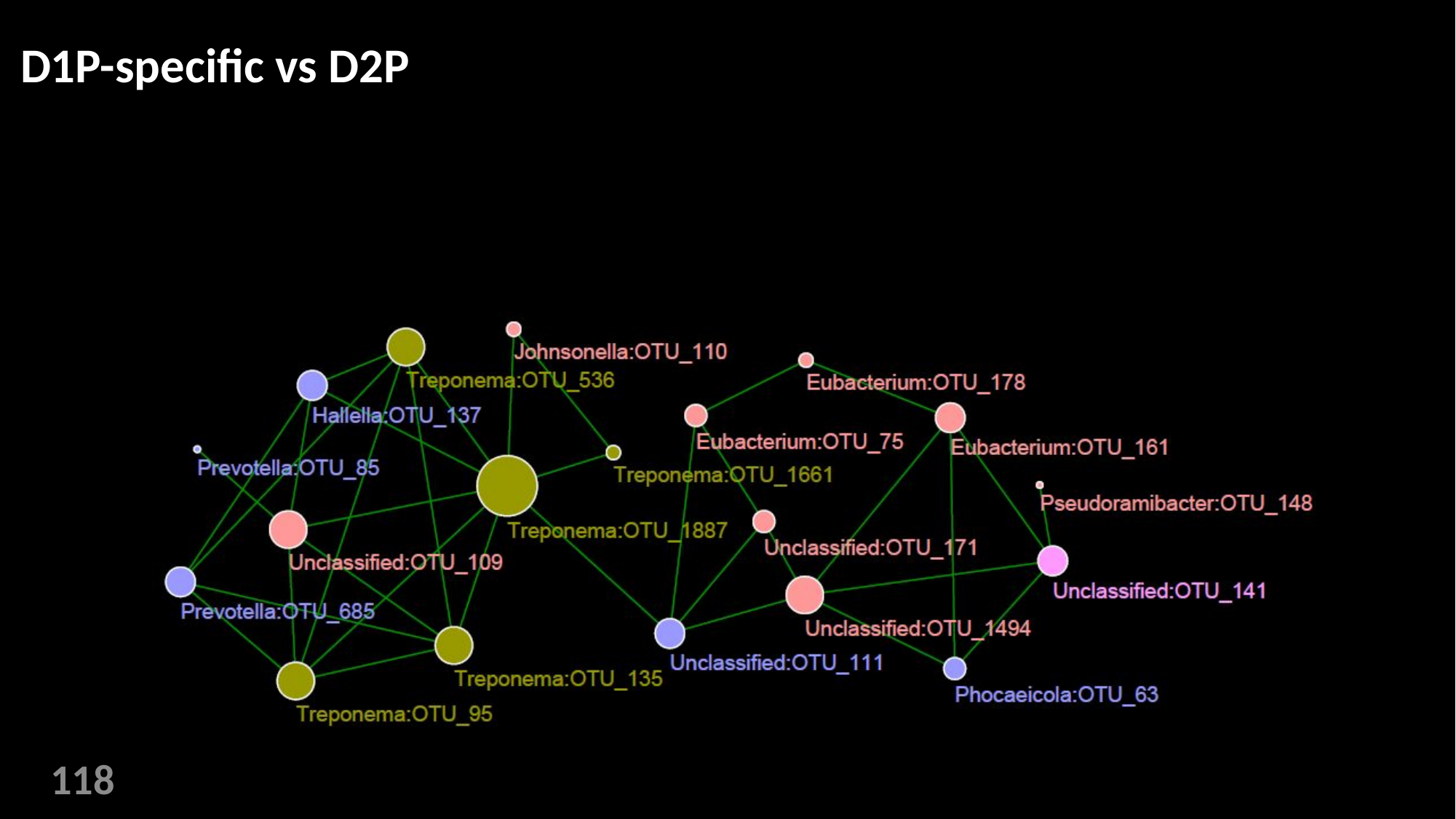

D1P-specific vs D2P
118

## Slide 119
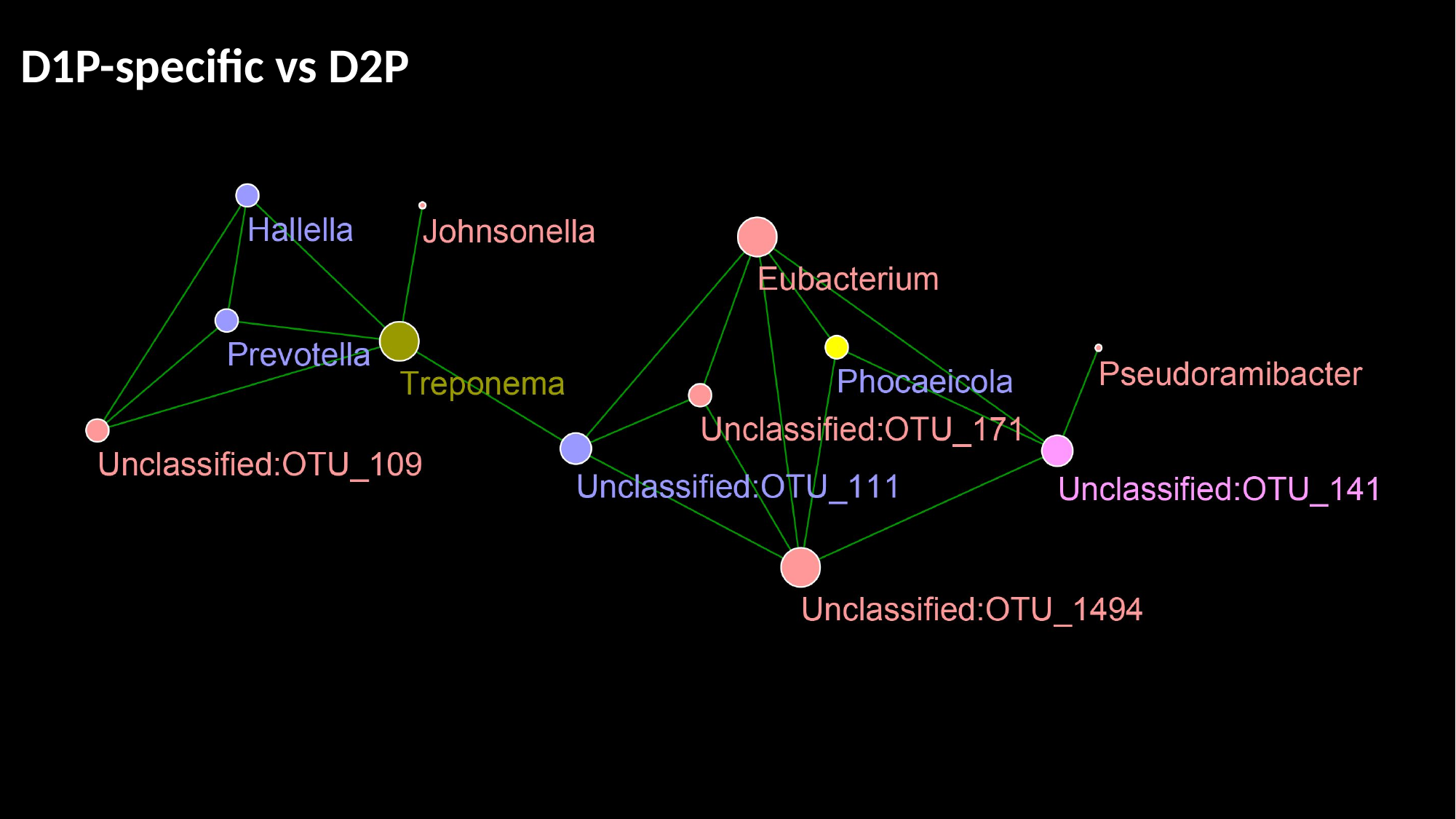

D1P-specific vs D2P
