# Supplementary material for: Oral microbiota of periodontal health and disease and their changes after nonsurgical periodontal therapy
Source: ISME J. 2018 Jan 16;12(5):1210–24. doi: 10.1038/s41396-017-0037-1 (PMC5932080; doi:10.1038/s41396-017-0037-1)
Supplement: Supplementary file 18 — Supplementary Figure S9 [file 41396_2017_37_MOESM18_ESM.pptx]

## Slide 1
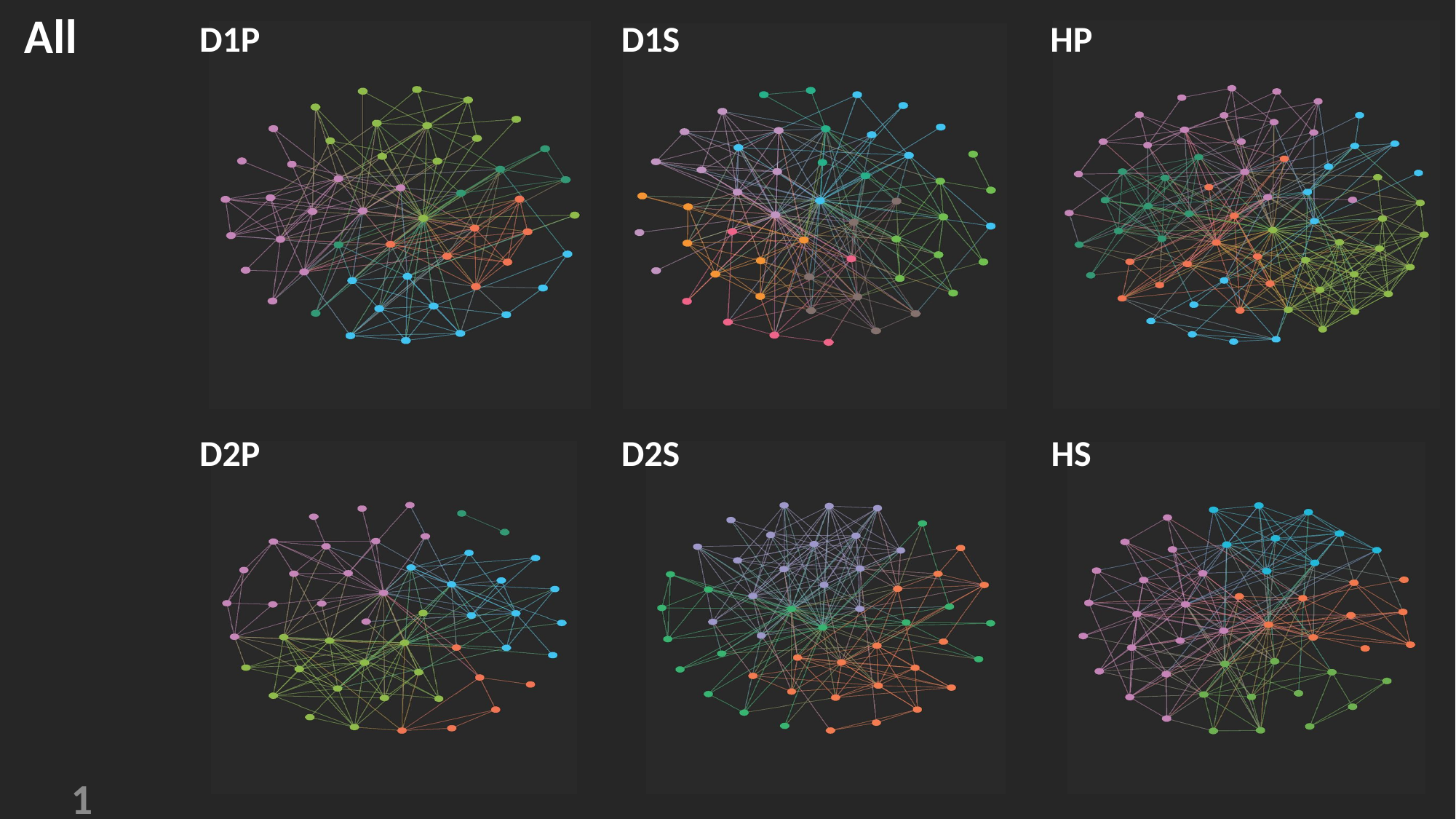

All
D1P
D1S
HP
D2P
D2S
HS
1

## Slide 2
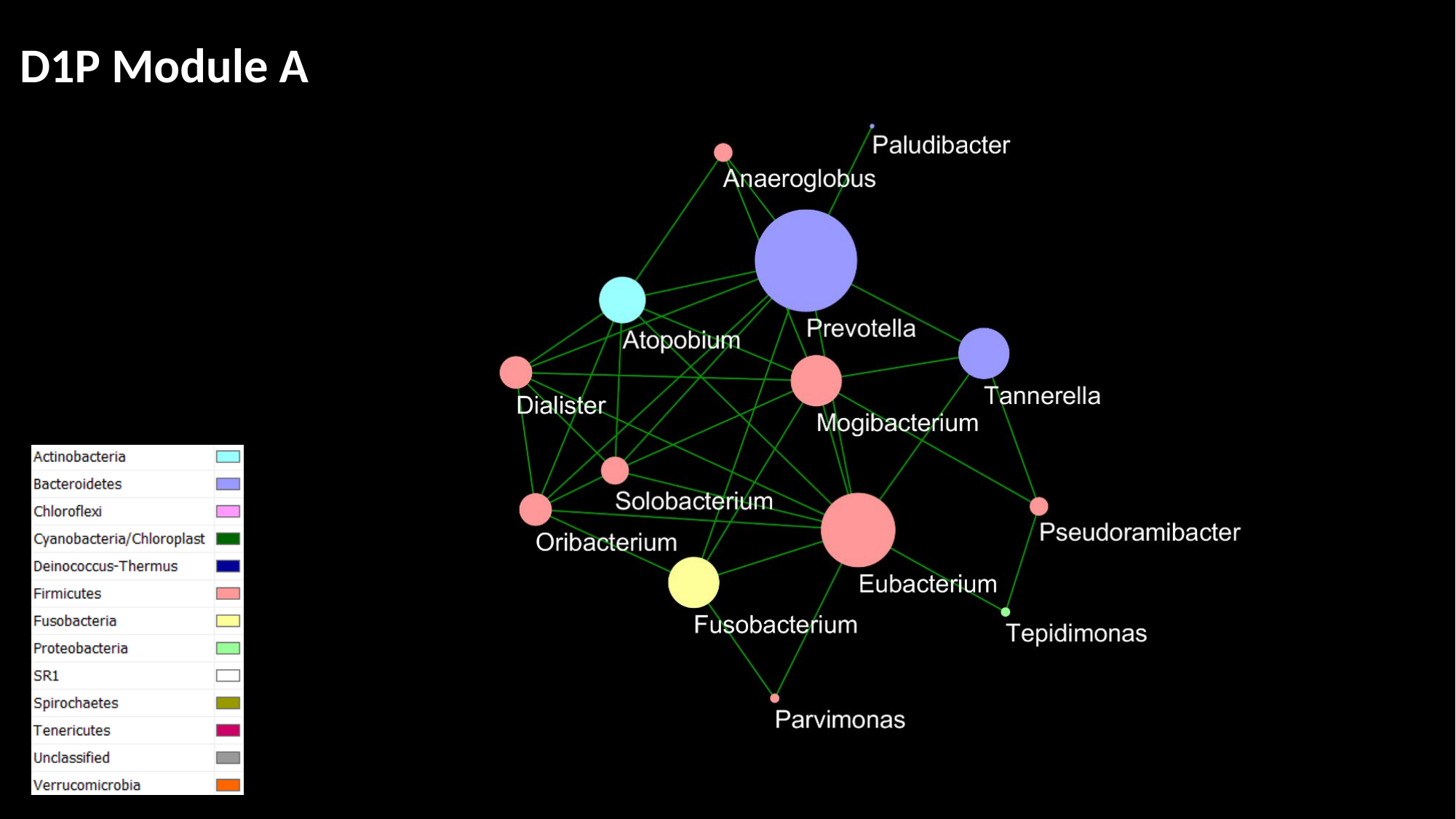

D1P Module A

## Slide 3
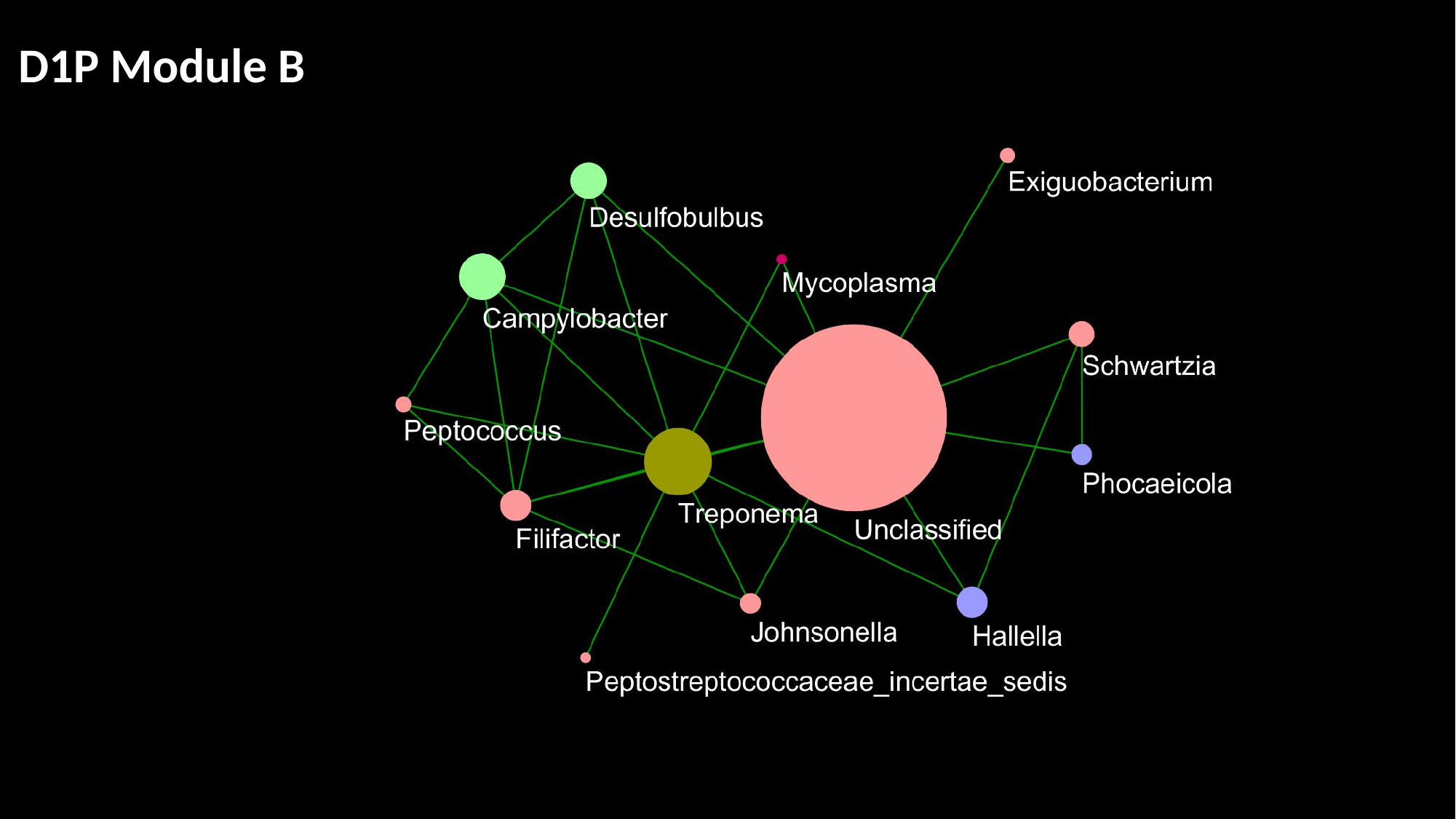

D1P Module B

## Slide 4
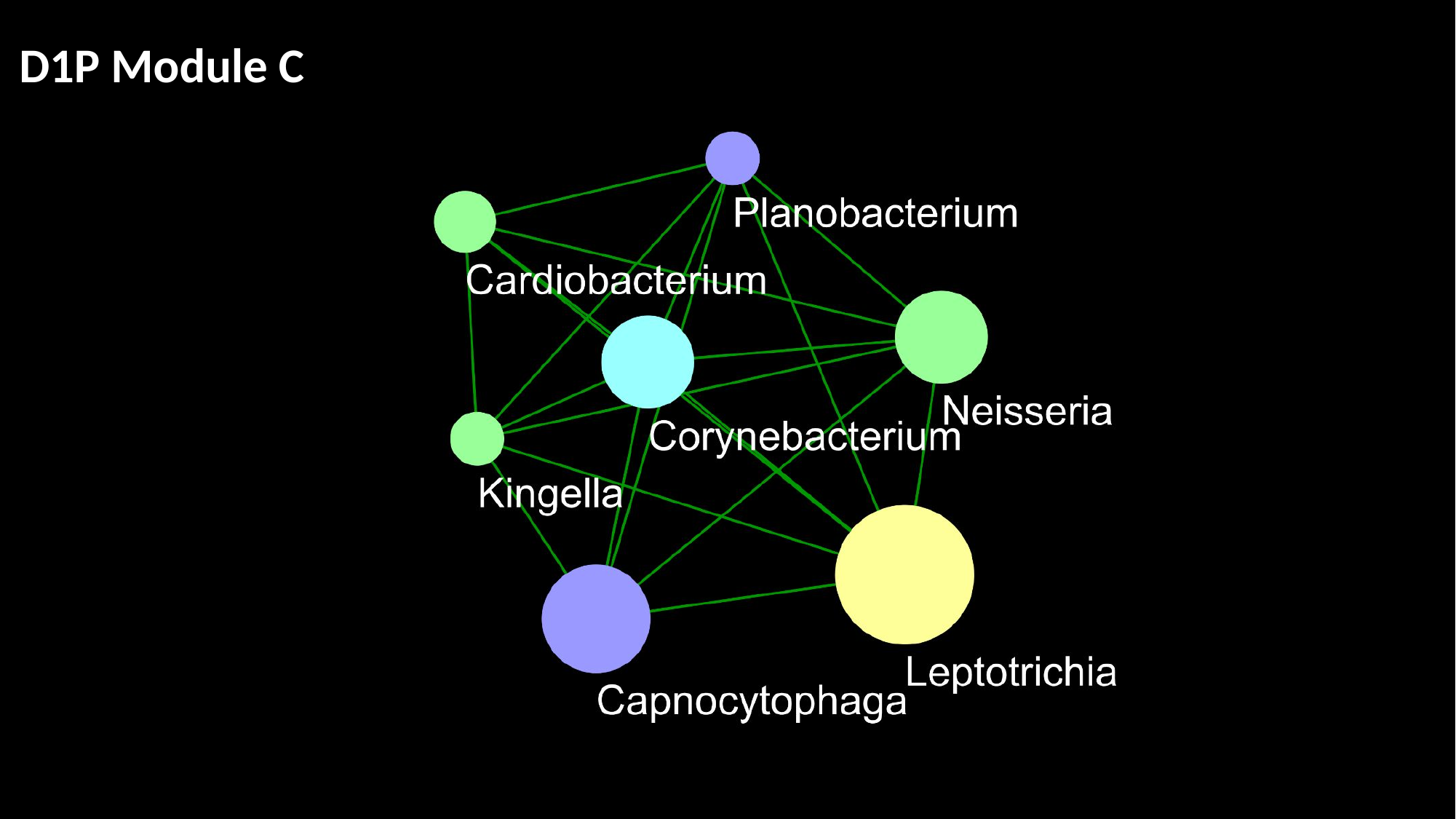

D1P Module C

## Slide 5
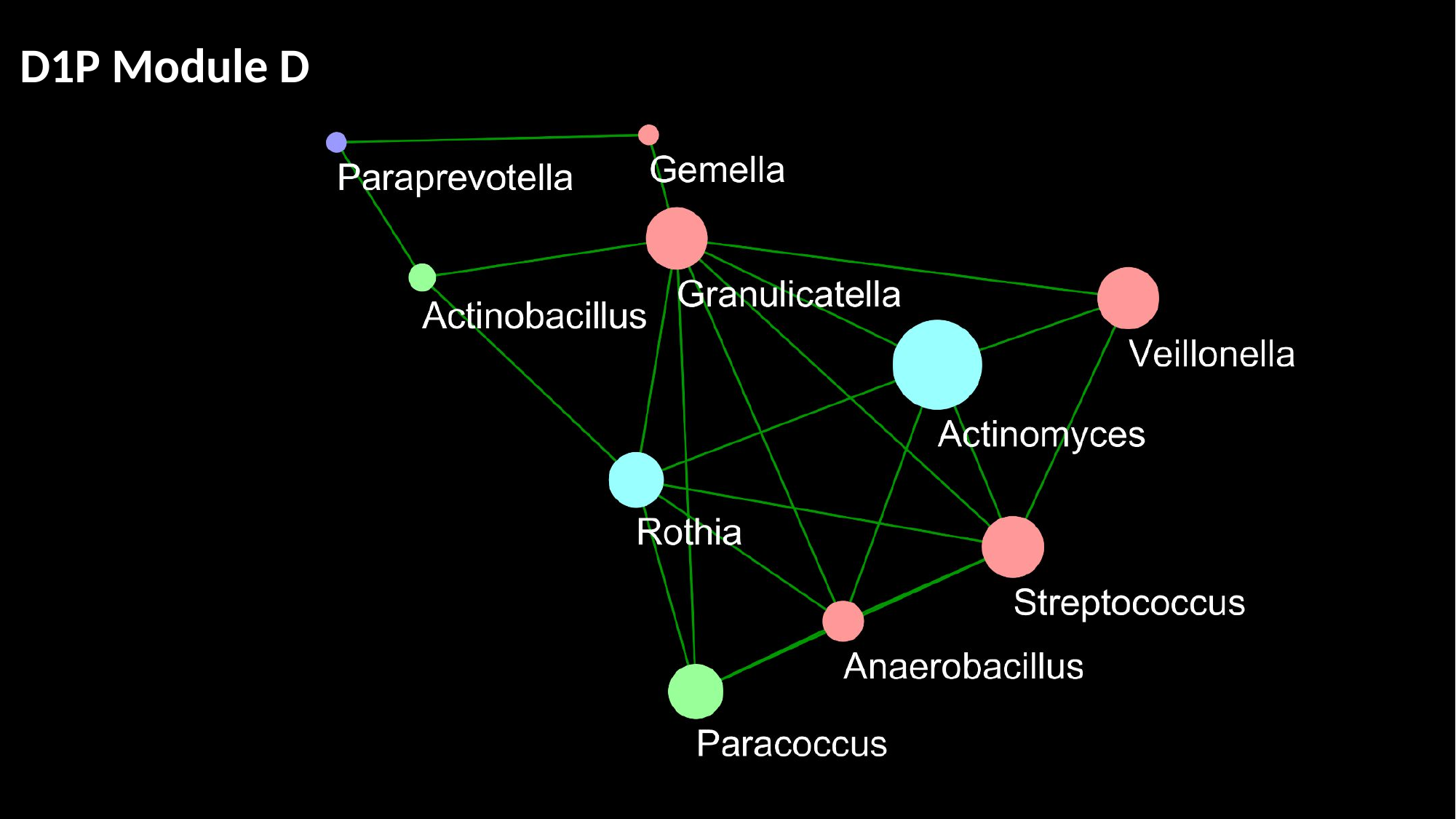

D1P Module D

## Slide 6
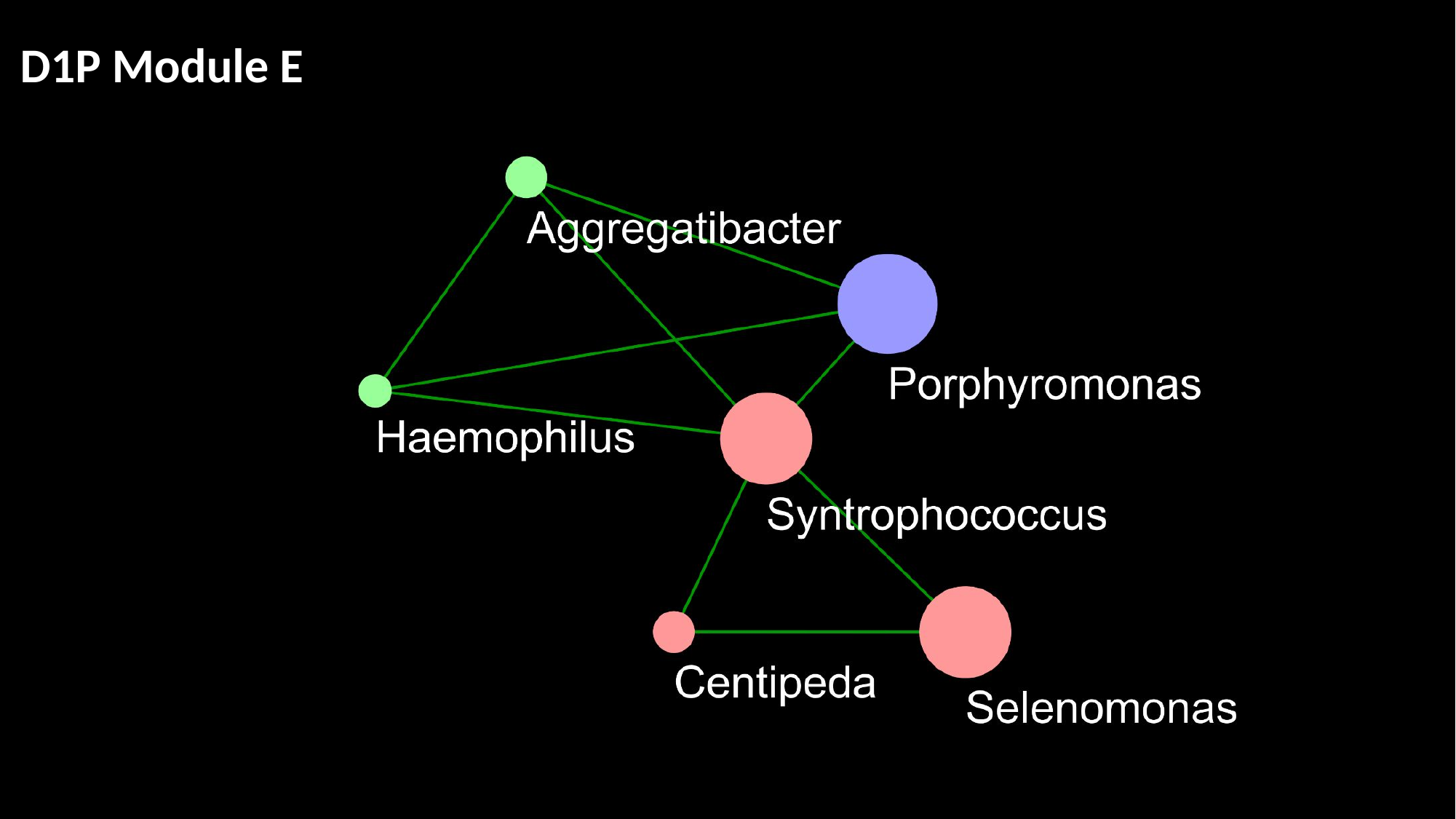

D1P Module E

## Slide 7
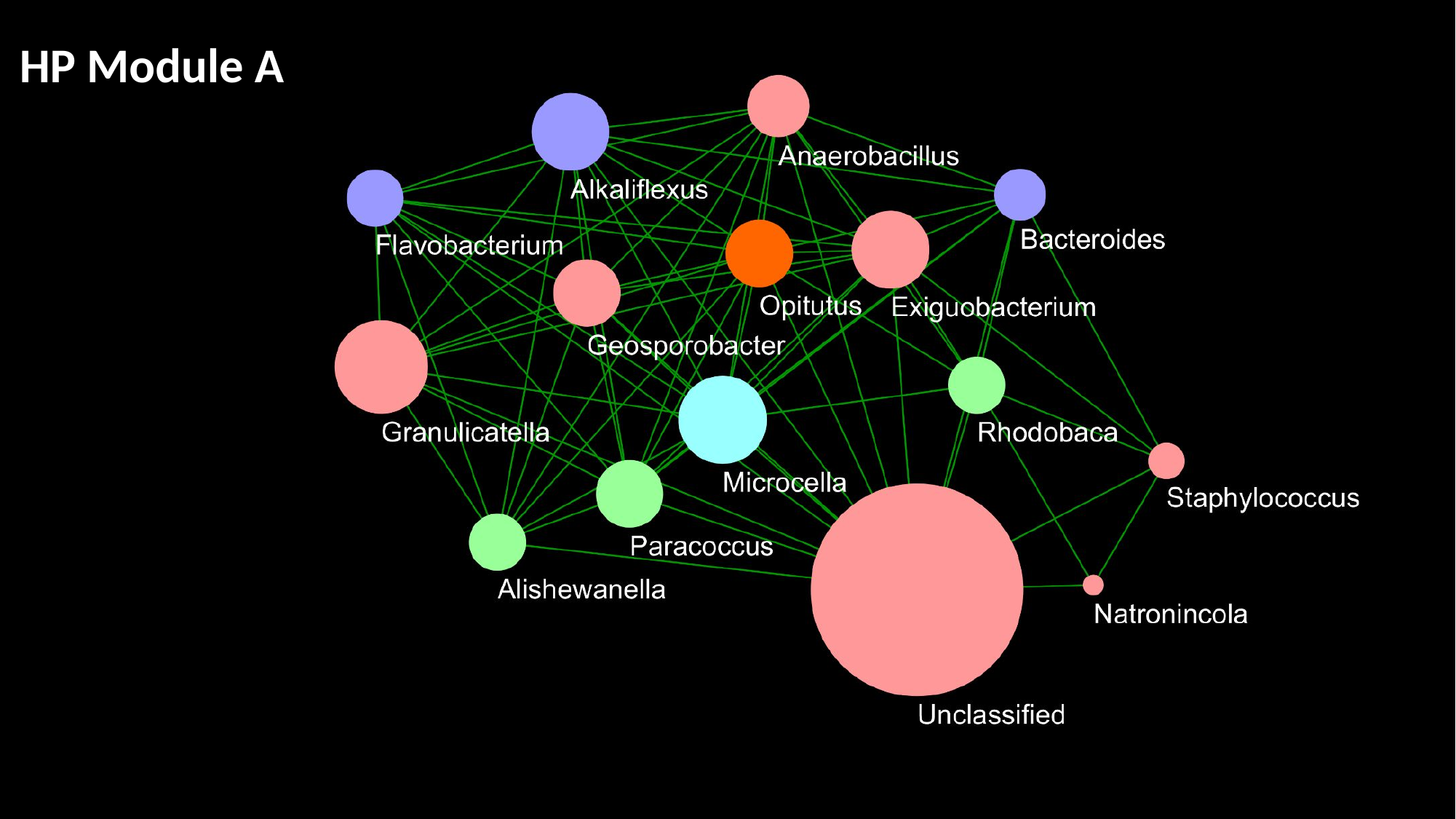

HP Module A

## Slide 8
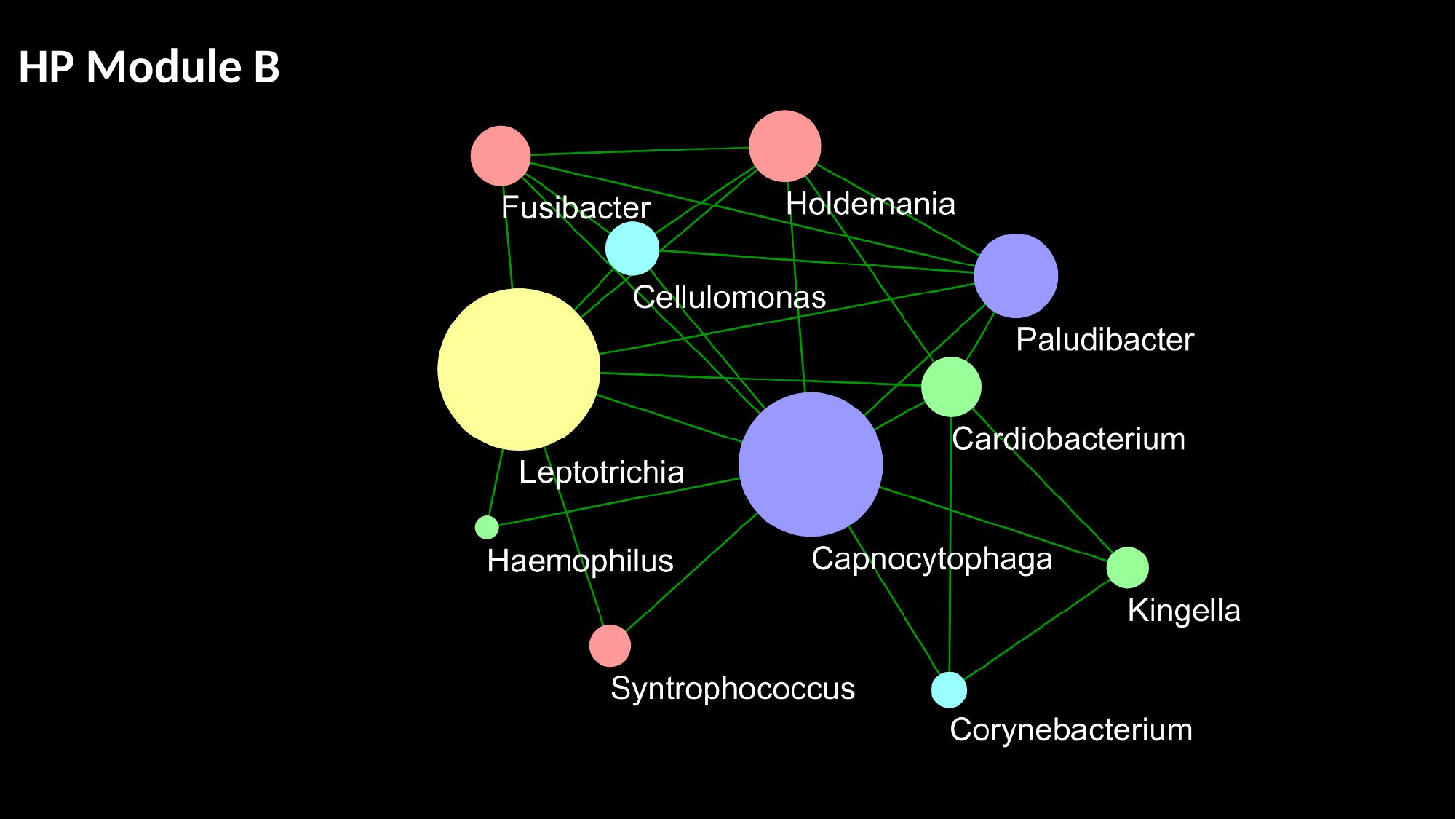

HP Module B

## Slide 9
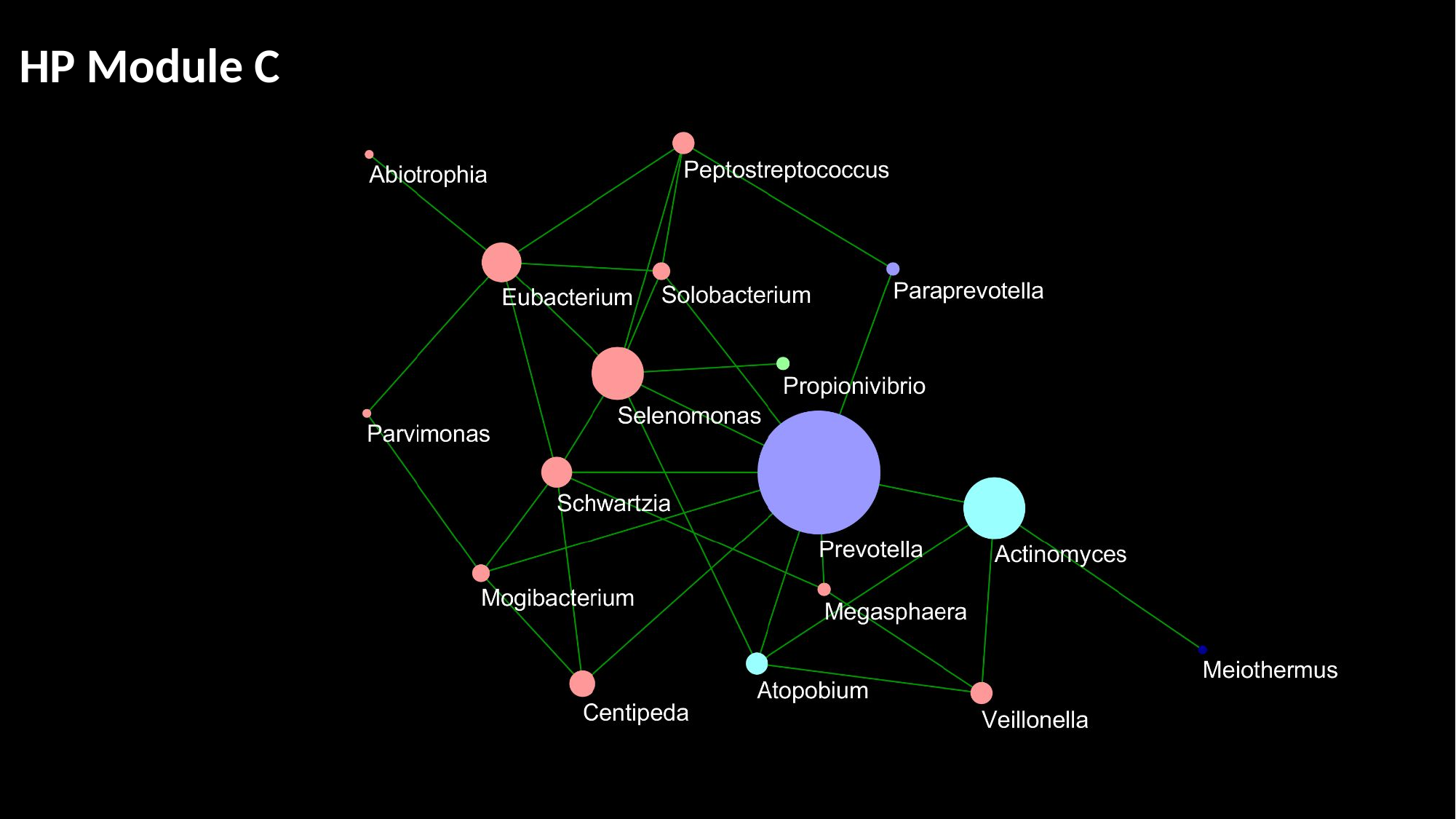

HP Module C

## Slide 10
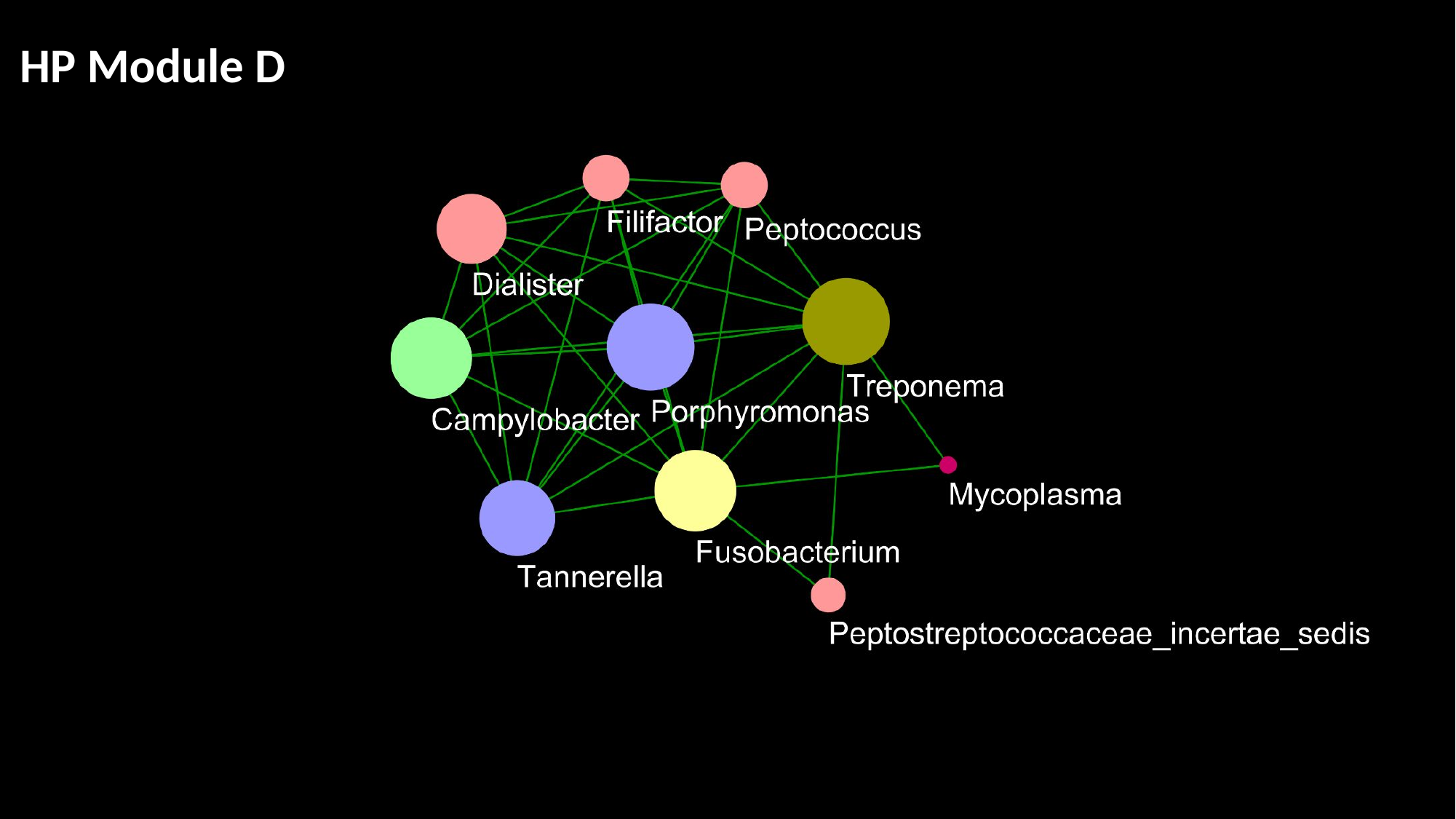

HP Module D

## Slide 11
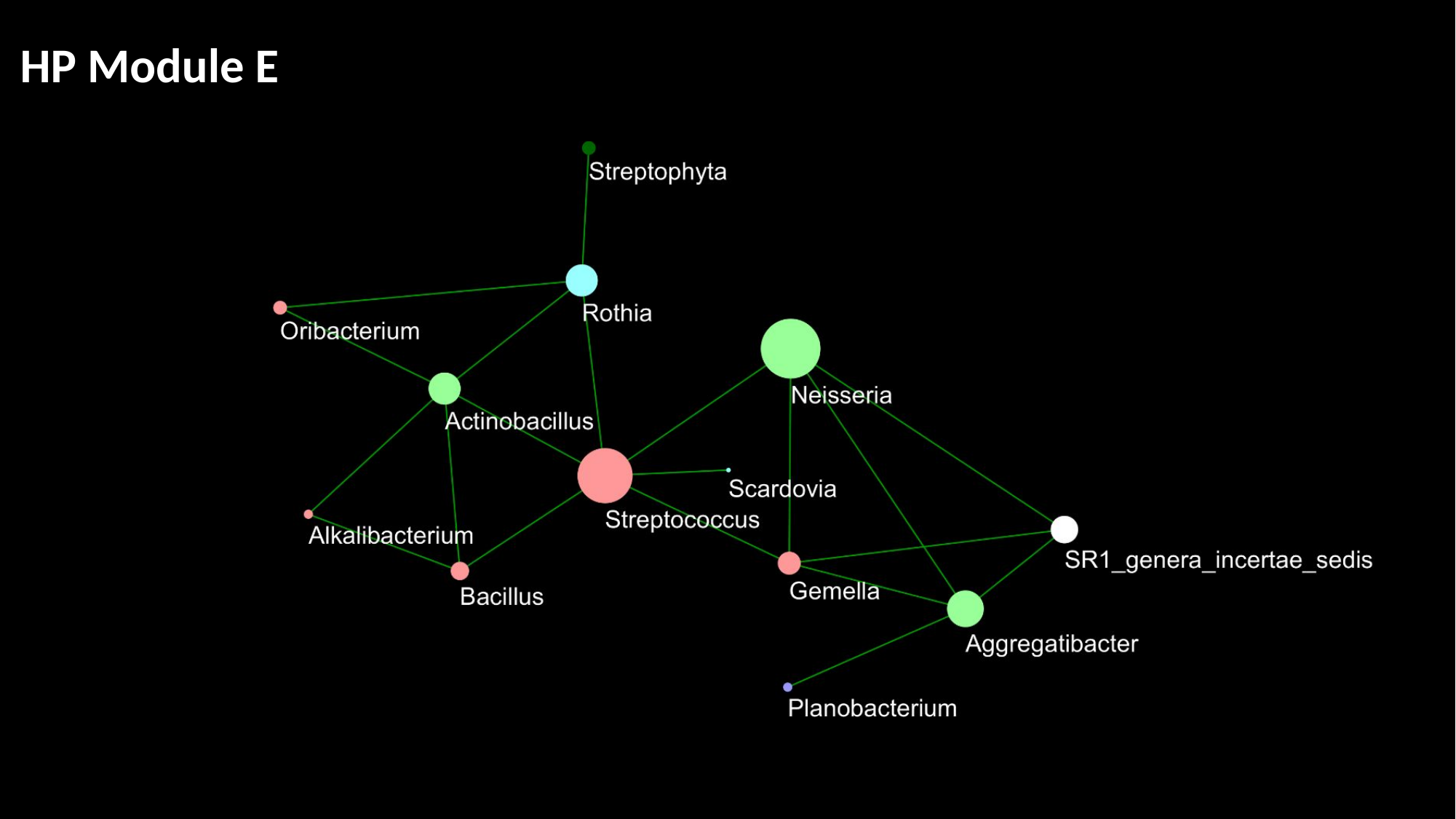

HP Module E

## Slide 12
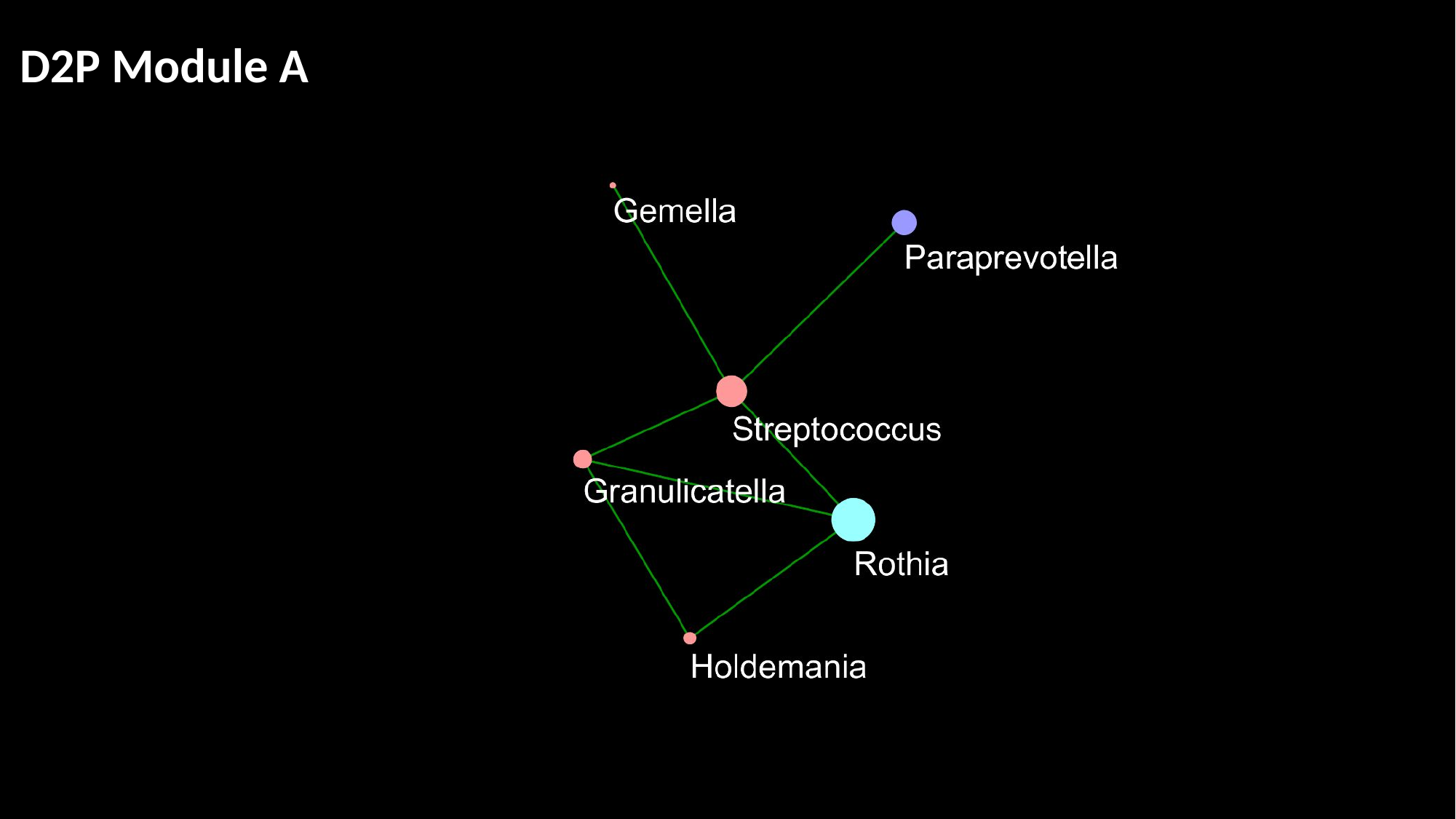

D2P Module A

## Slide 13
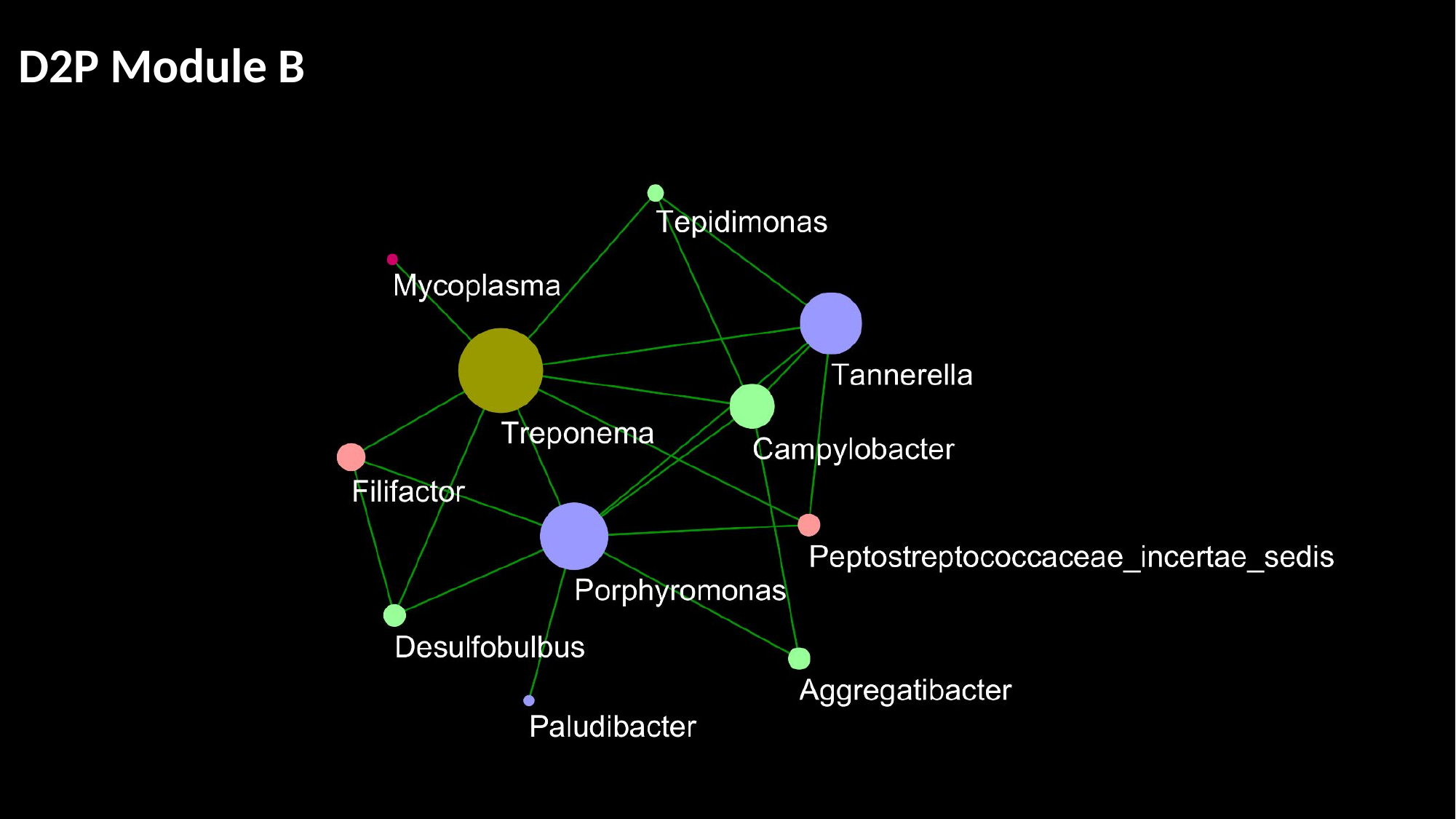

D2P Module B

## Slide 14
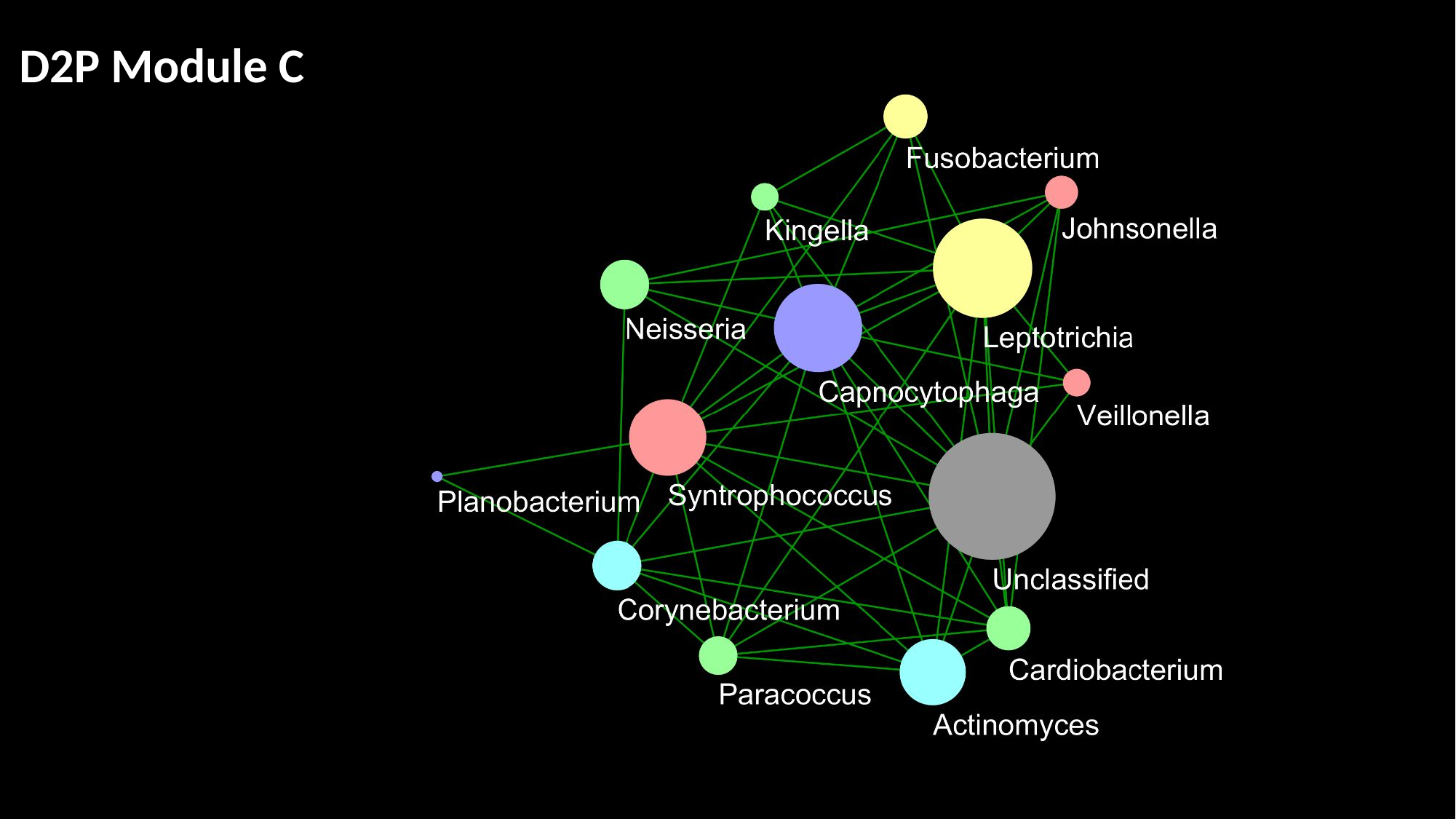

D2P Module C

## Slide 15
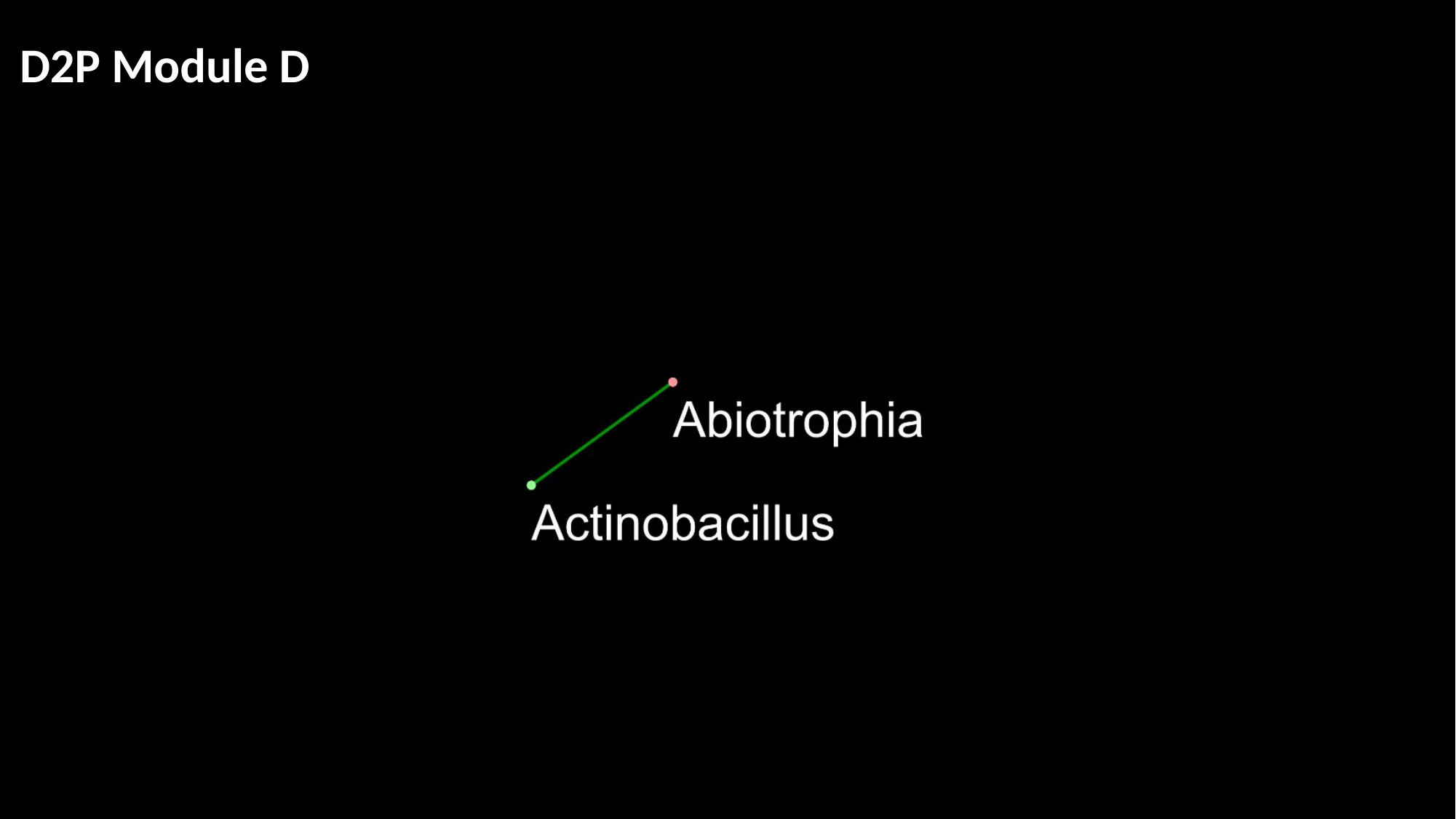

D2P Module D

## Slide 16
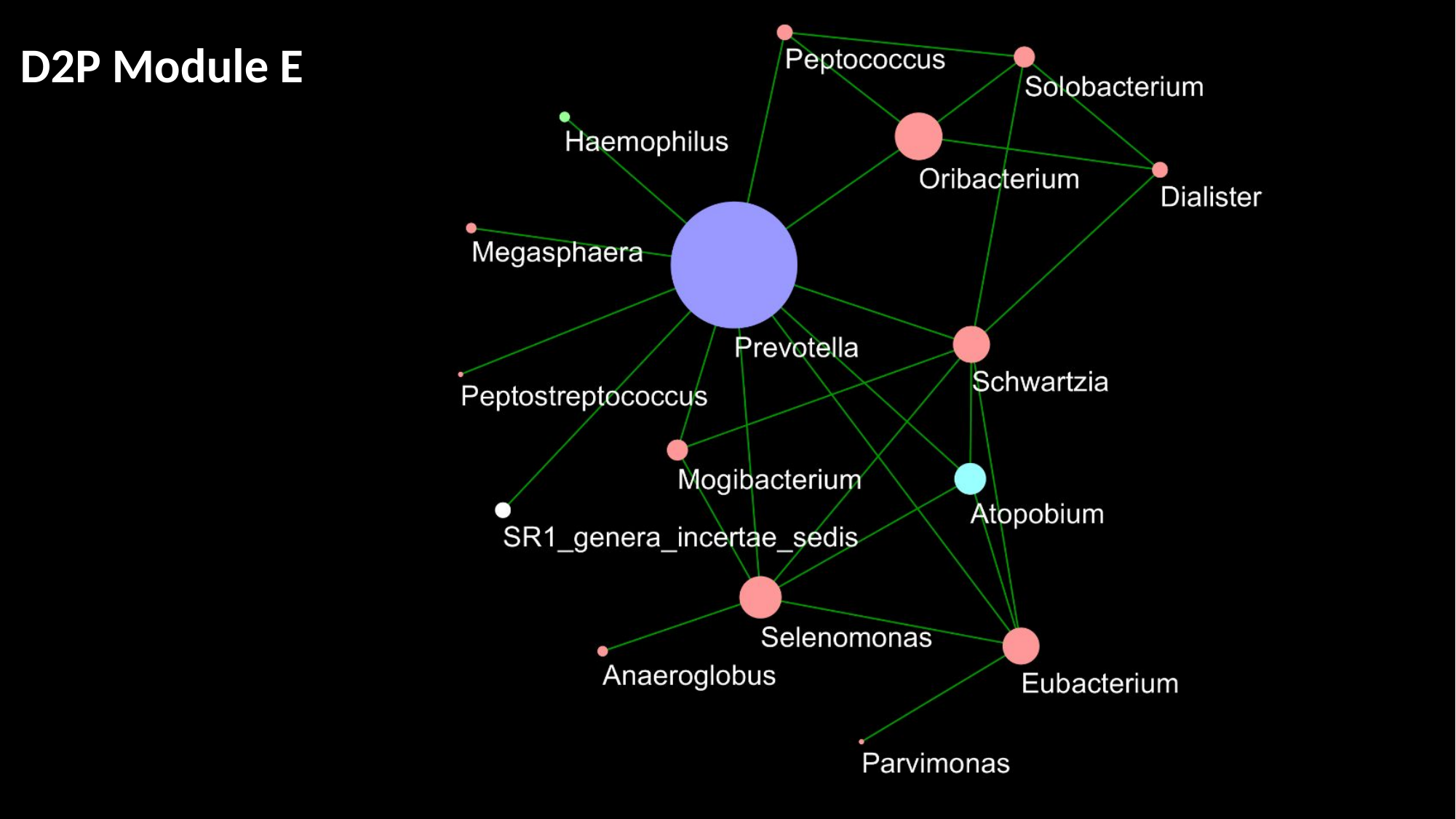

D2P Module E

## Slide 17
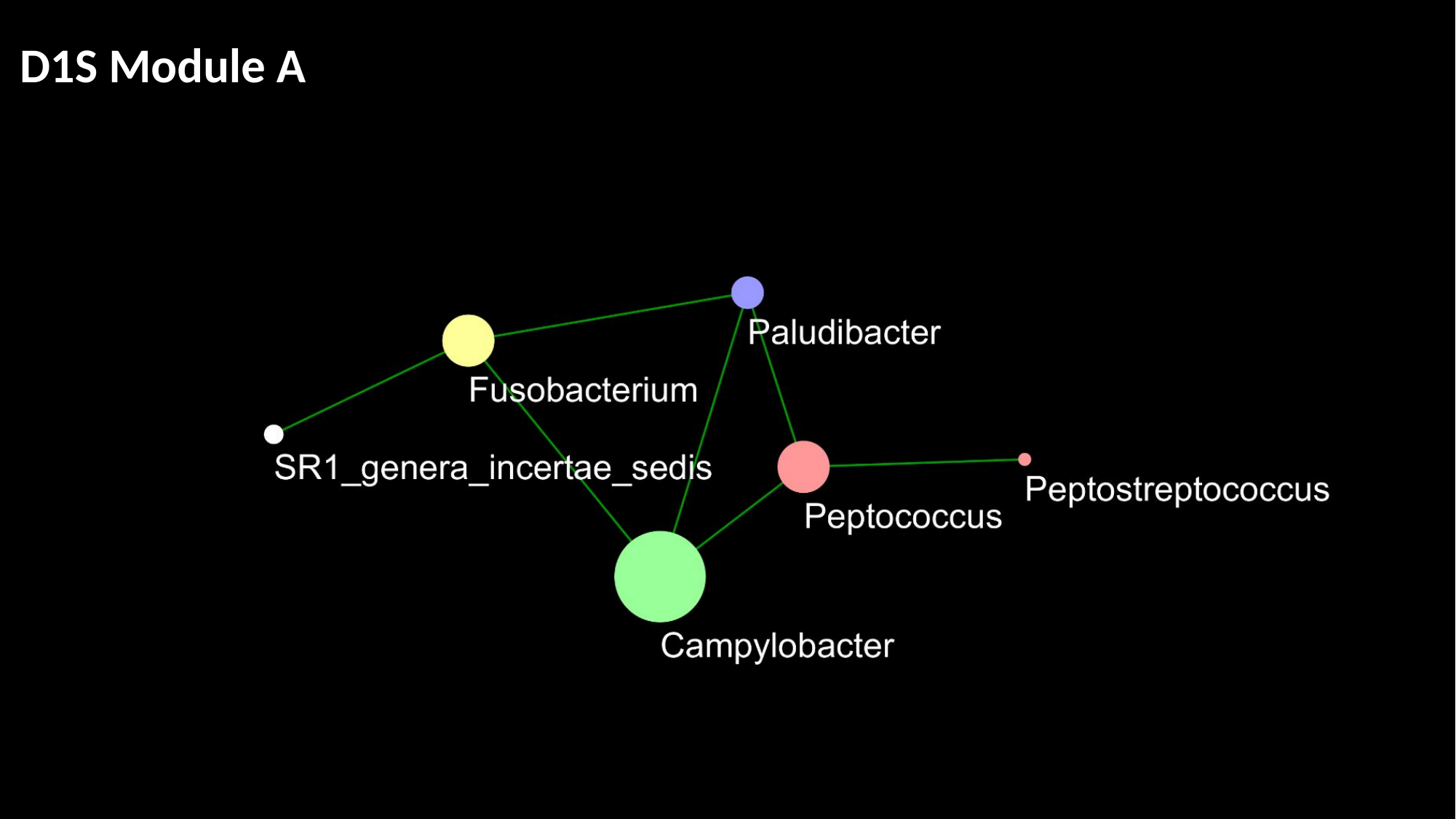

D1S Module A

## Slide 18
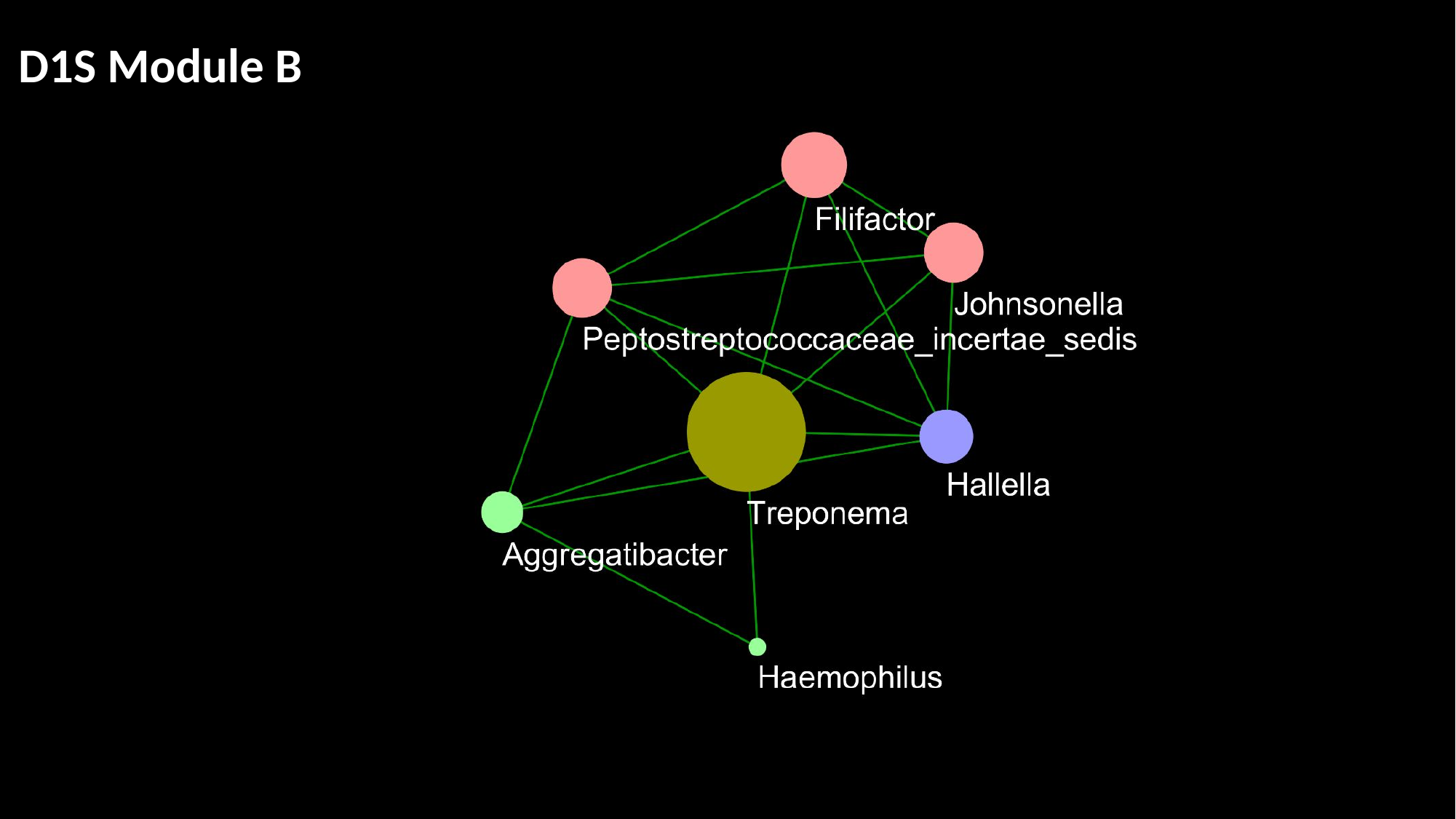

D1S Module B

## Slide 19
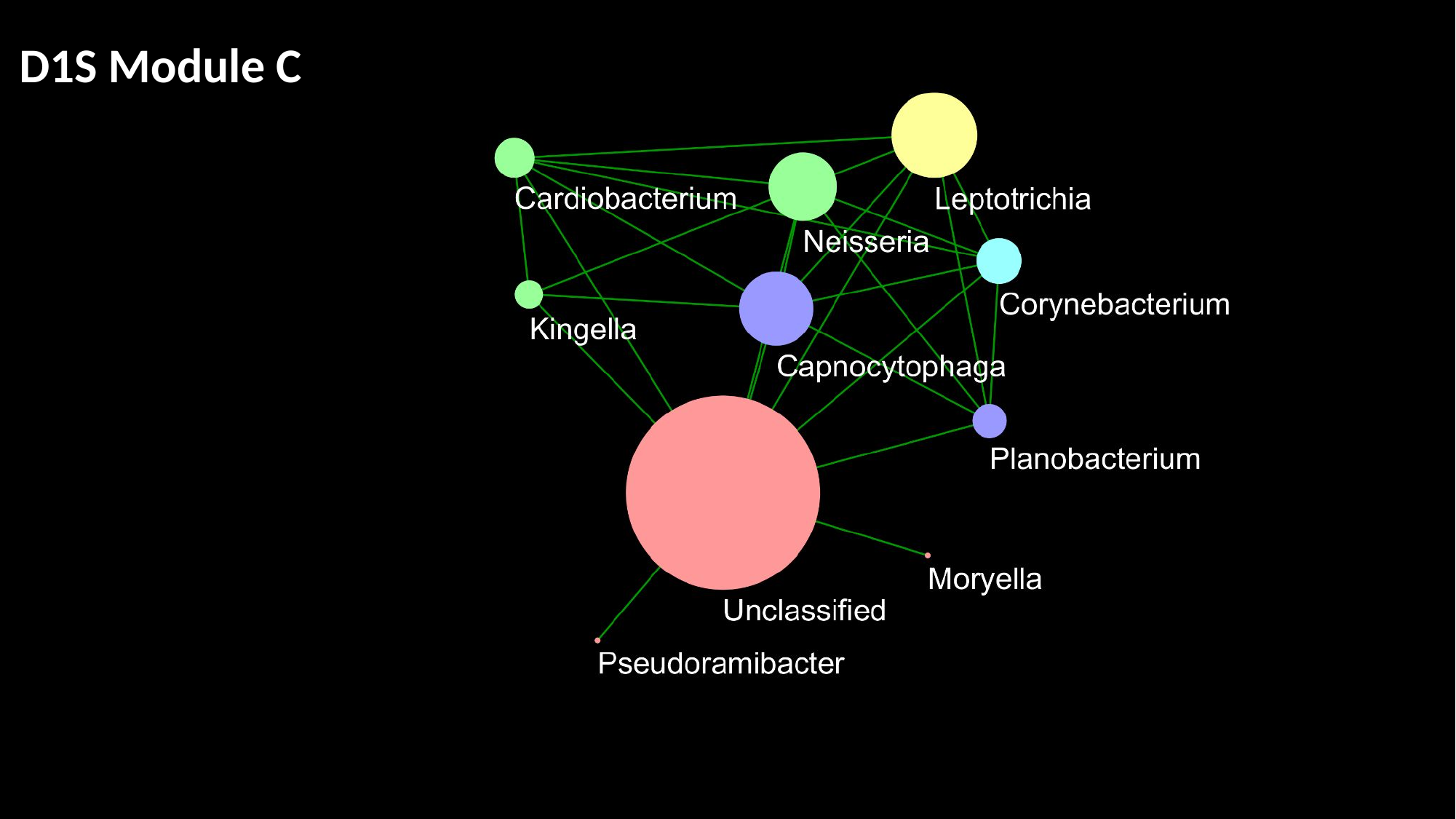

D1S Module C

## Slide 20
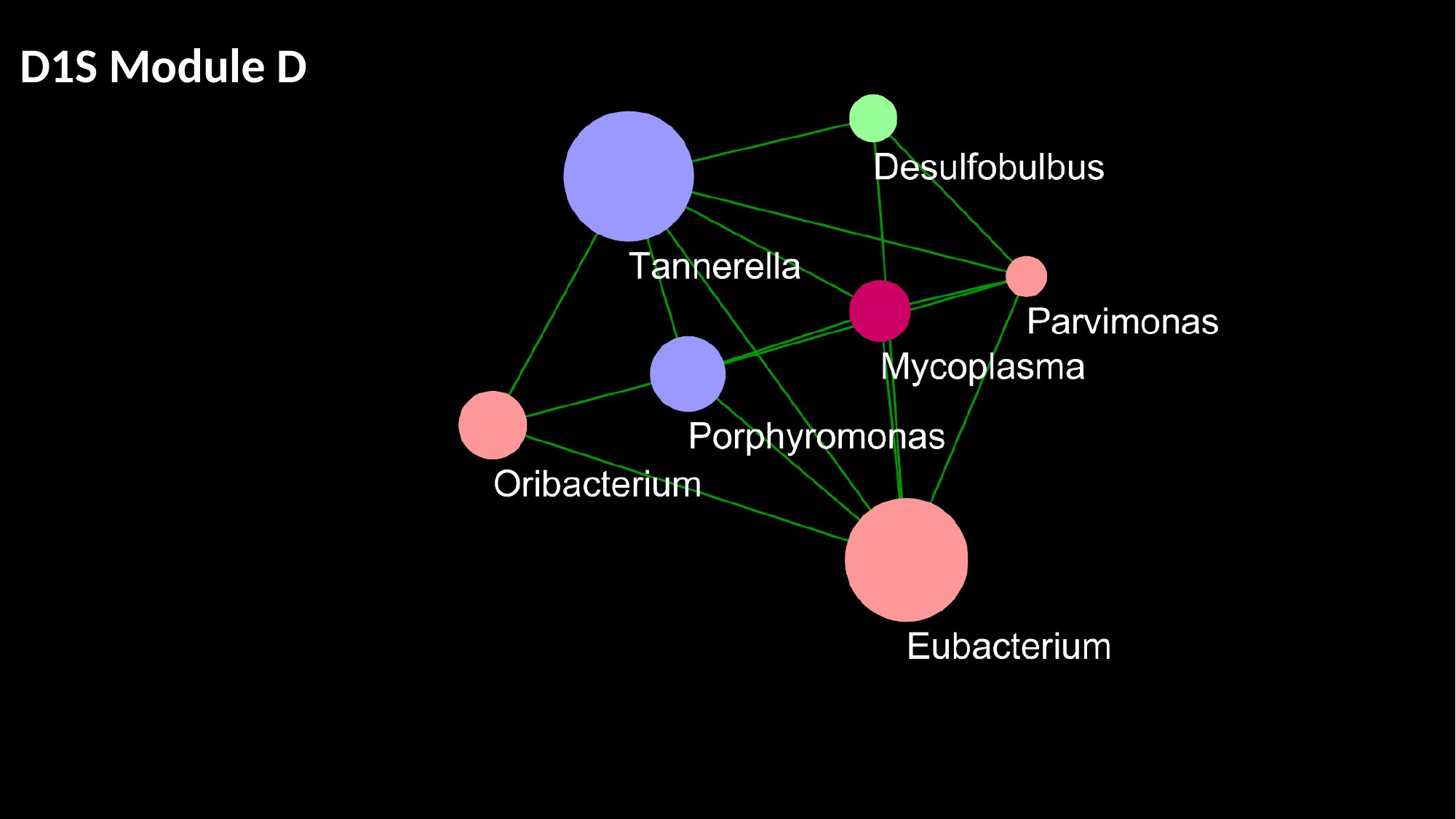

D1S Module D

## Slide 21
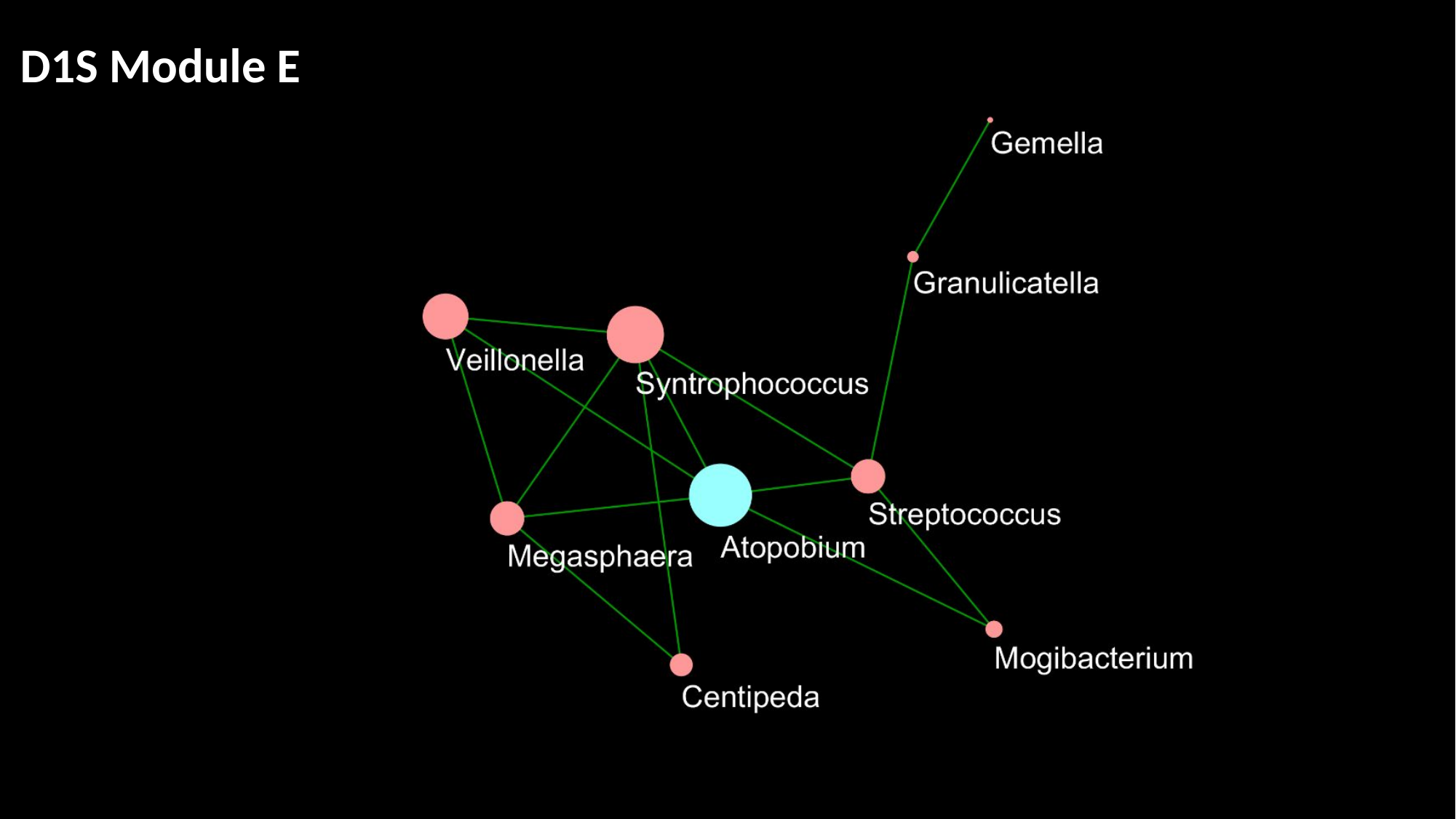

D1S Module E

## Slide 22
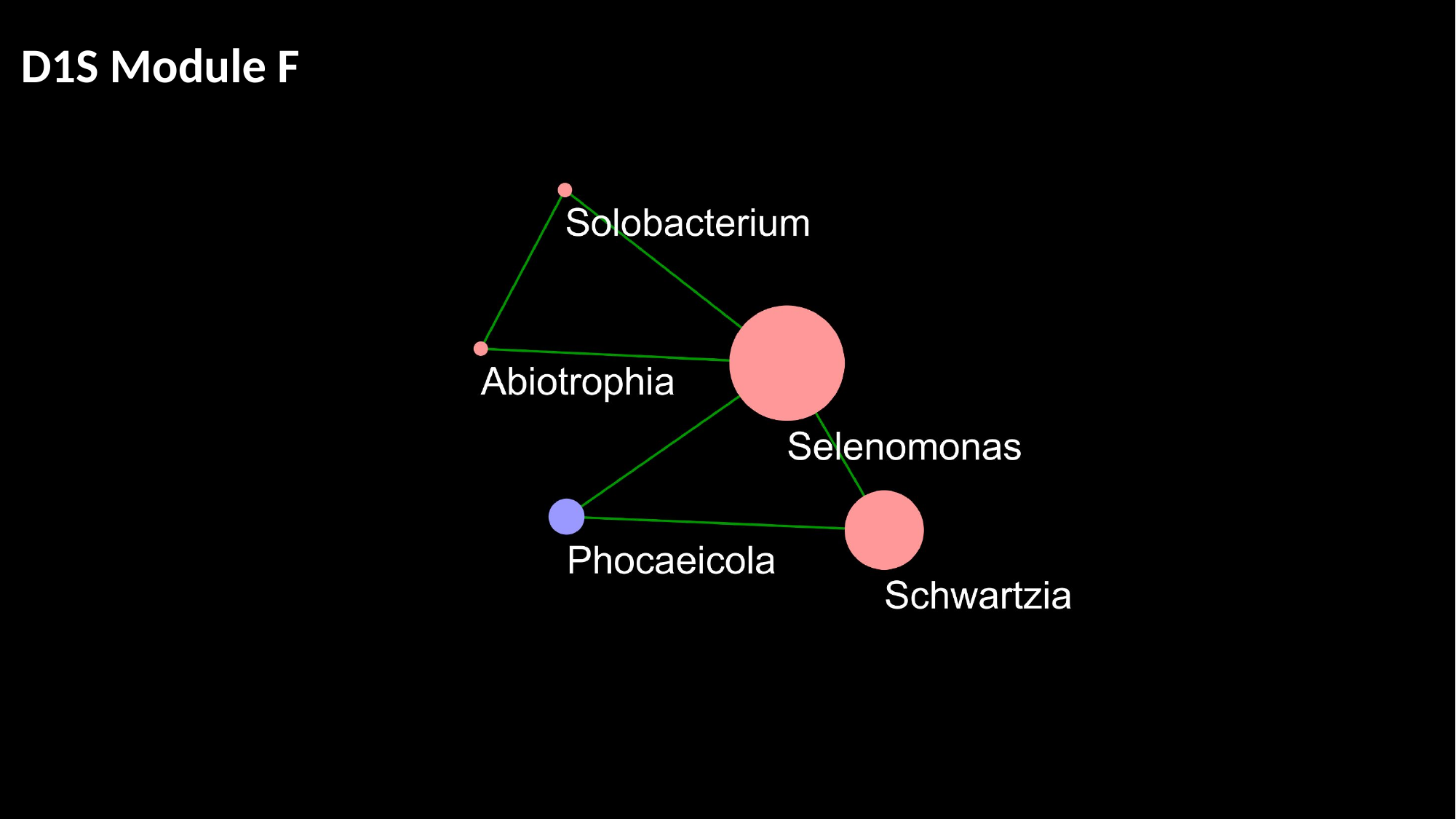

D1S Module F

## Slide 23
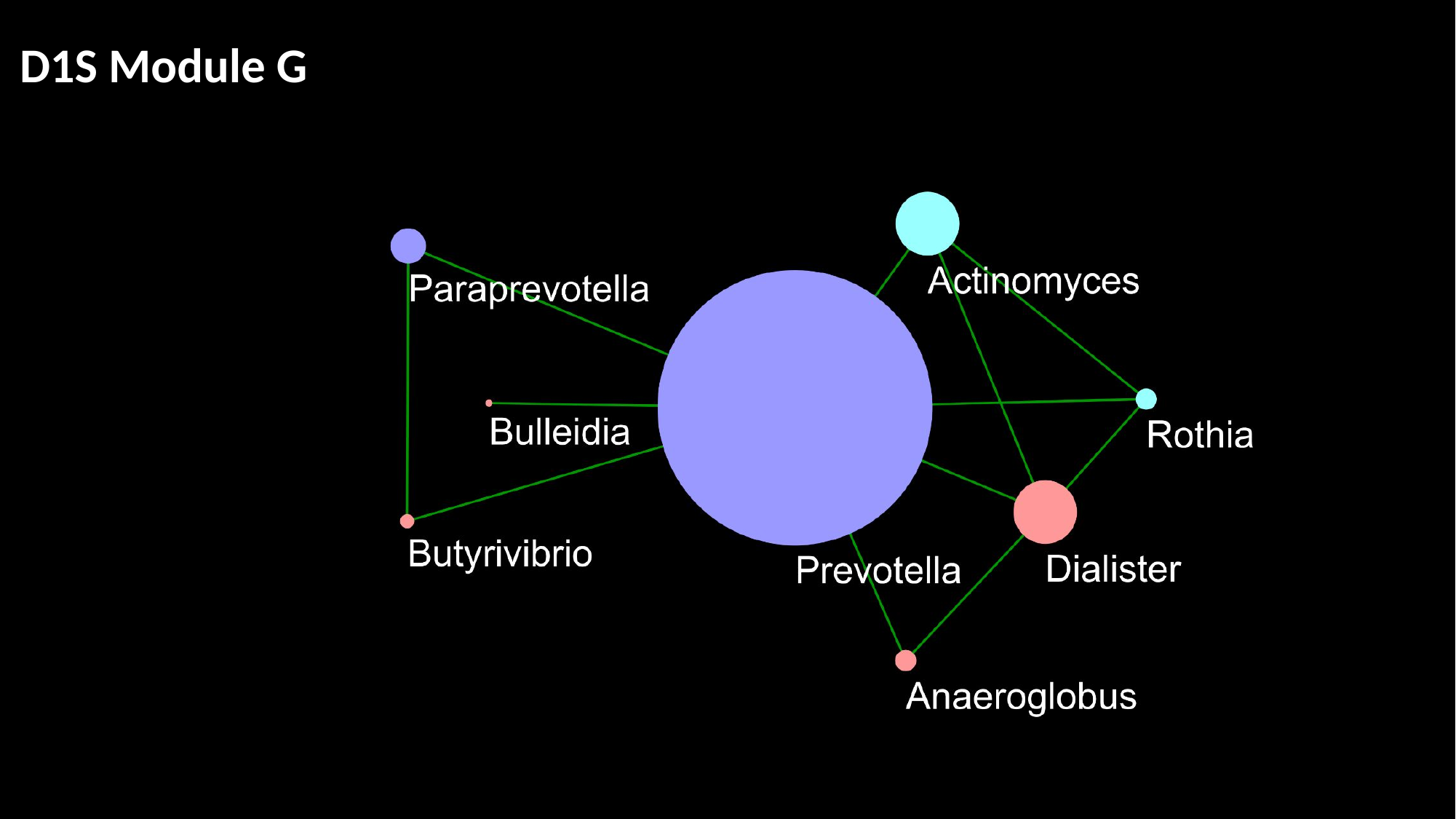

D1S Module G

## Slide 24
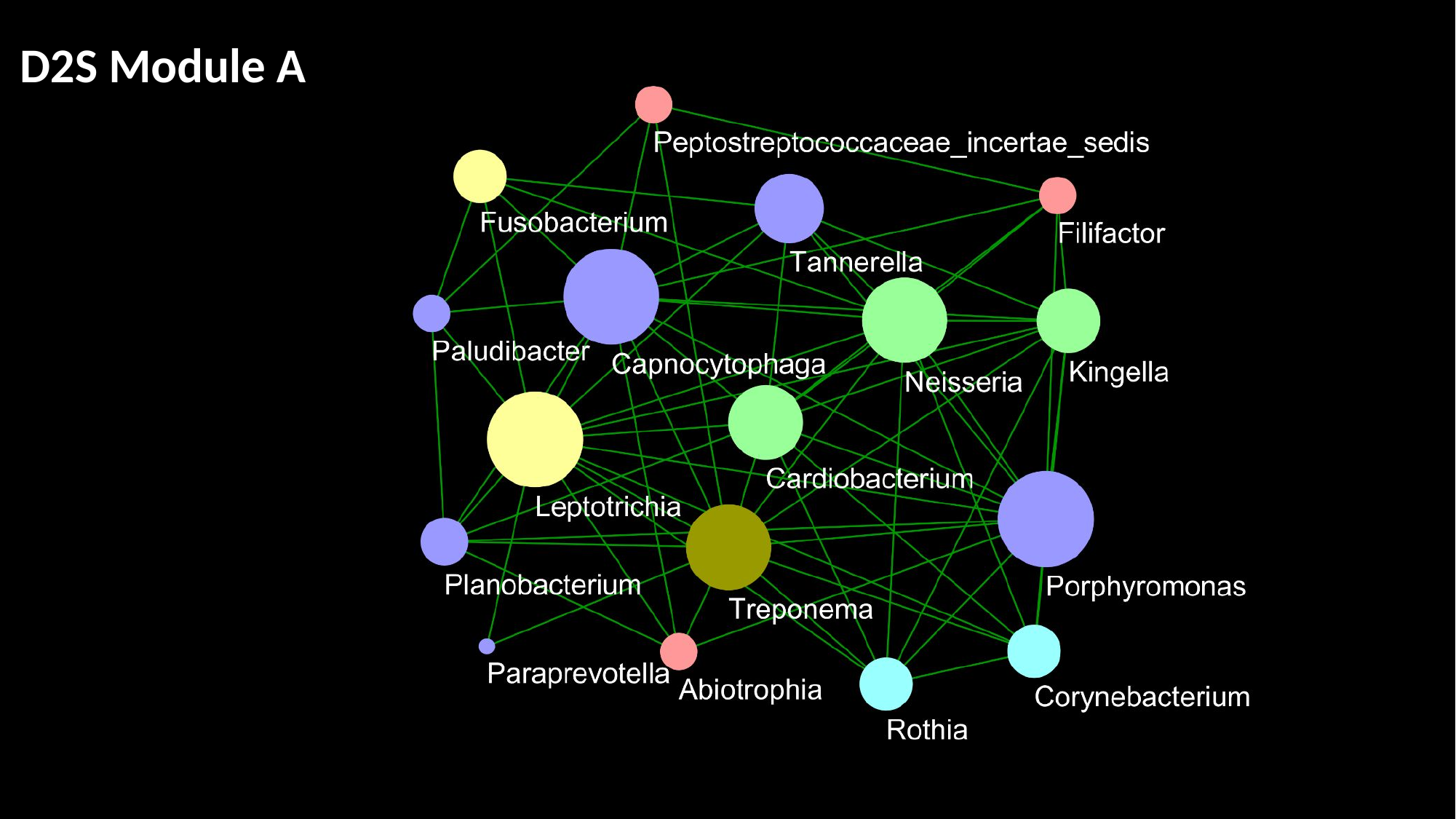

D2S Module A

## Slide 25
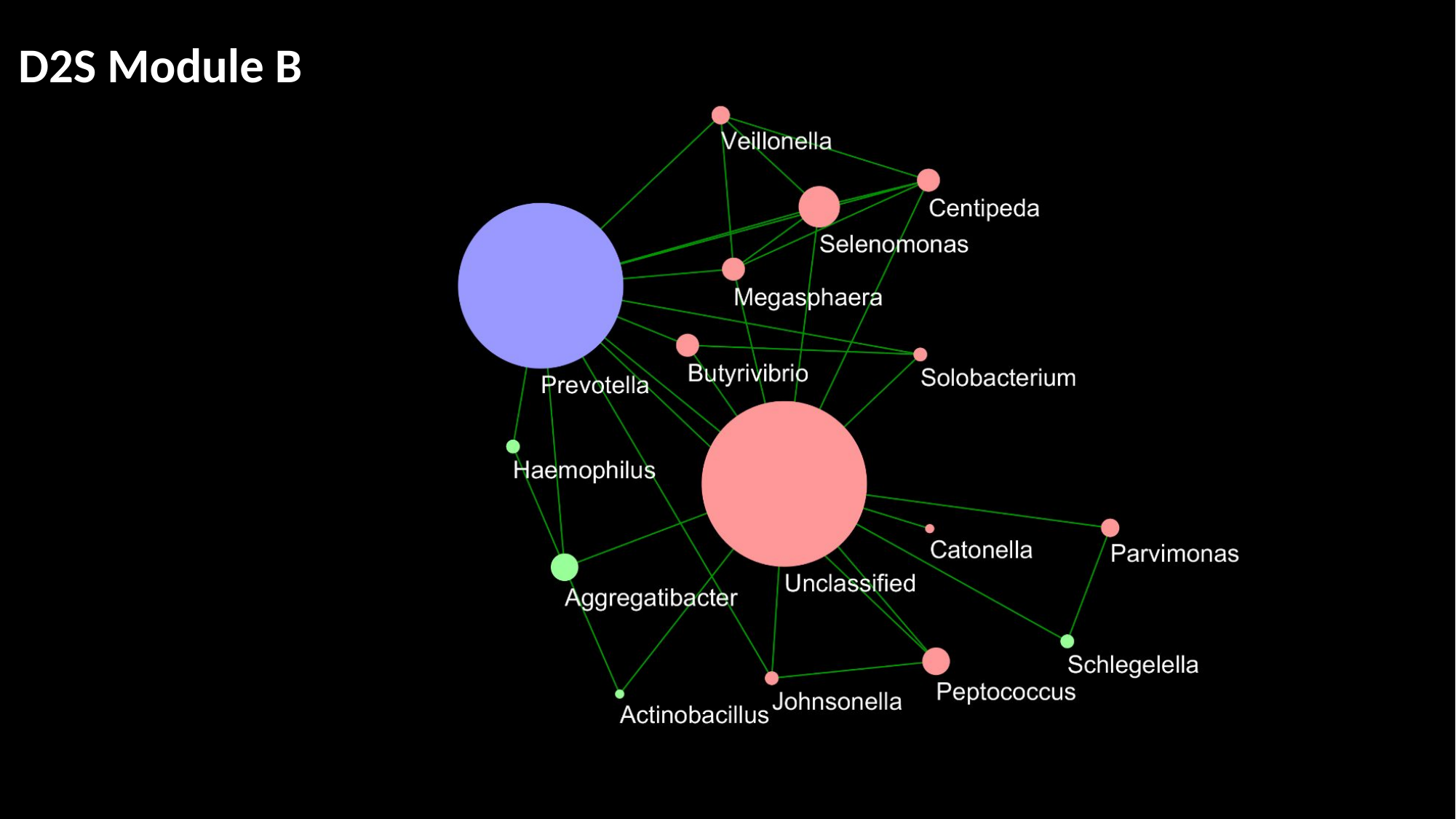

D2S Module B

## Slide 26
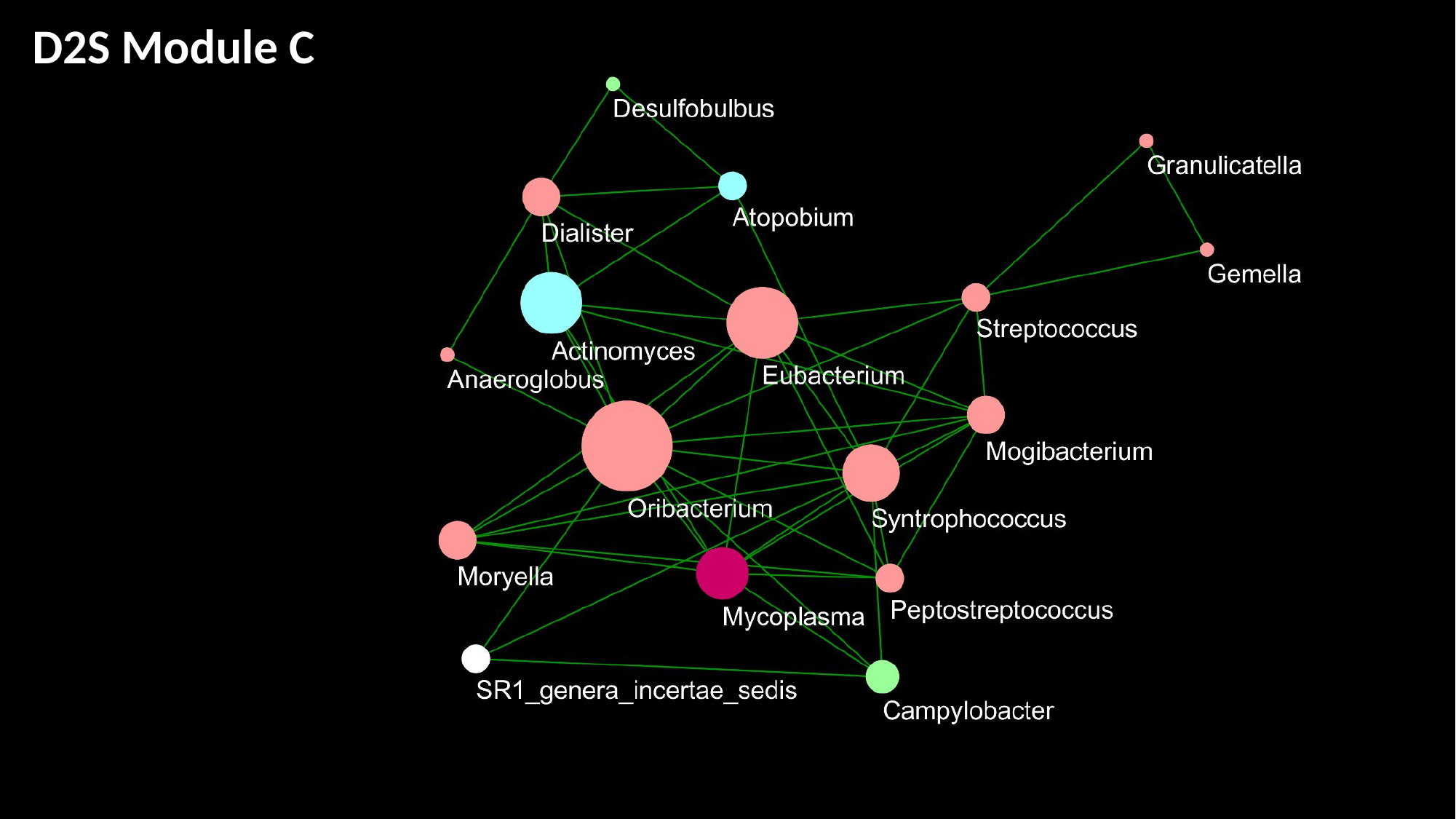

D2S Module C

## Slide 27
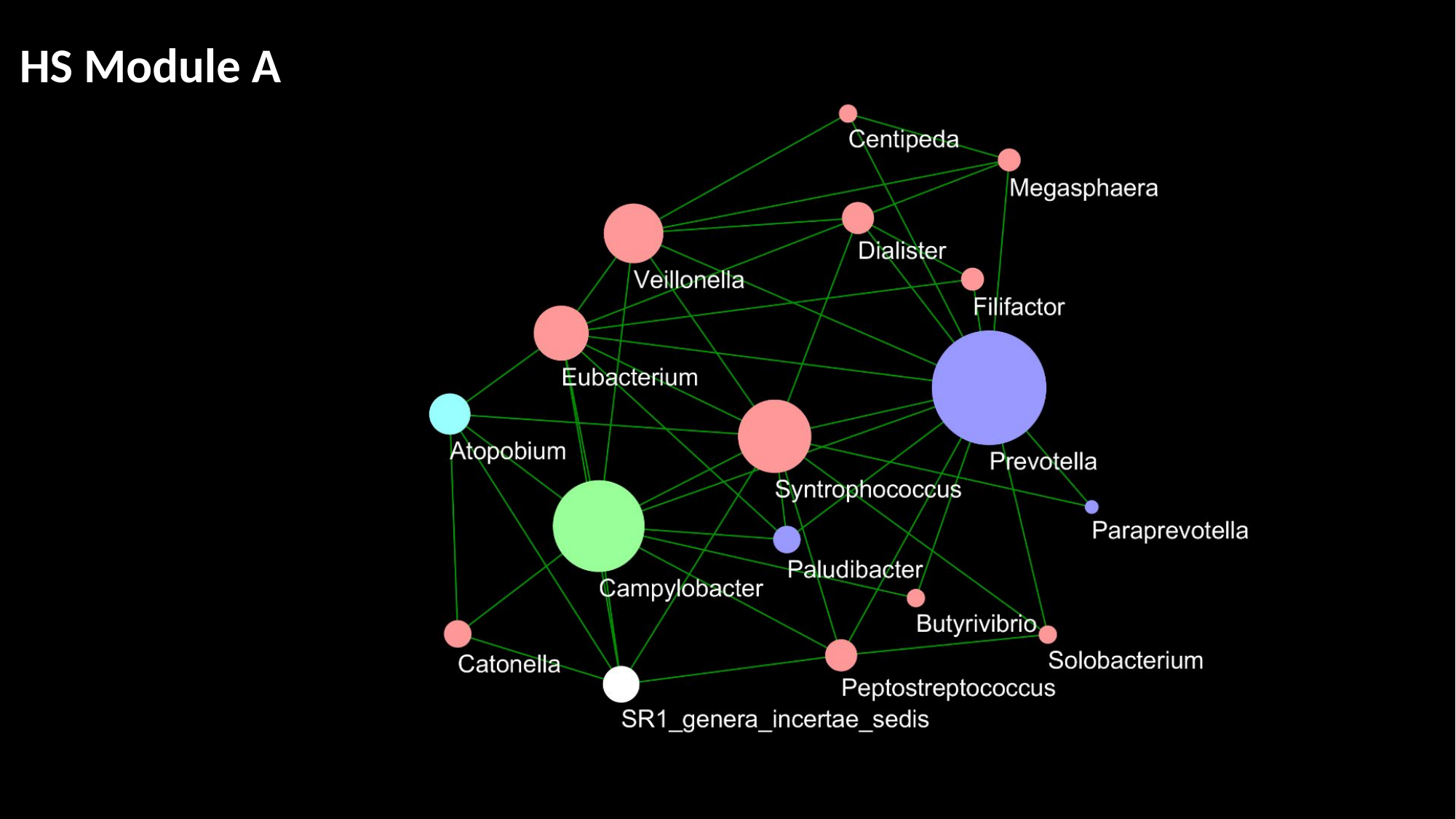

HS Module A

## Slide 28
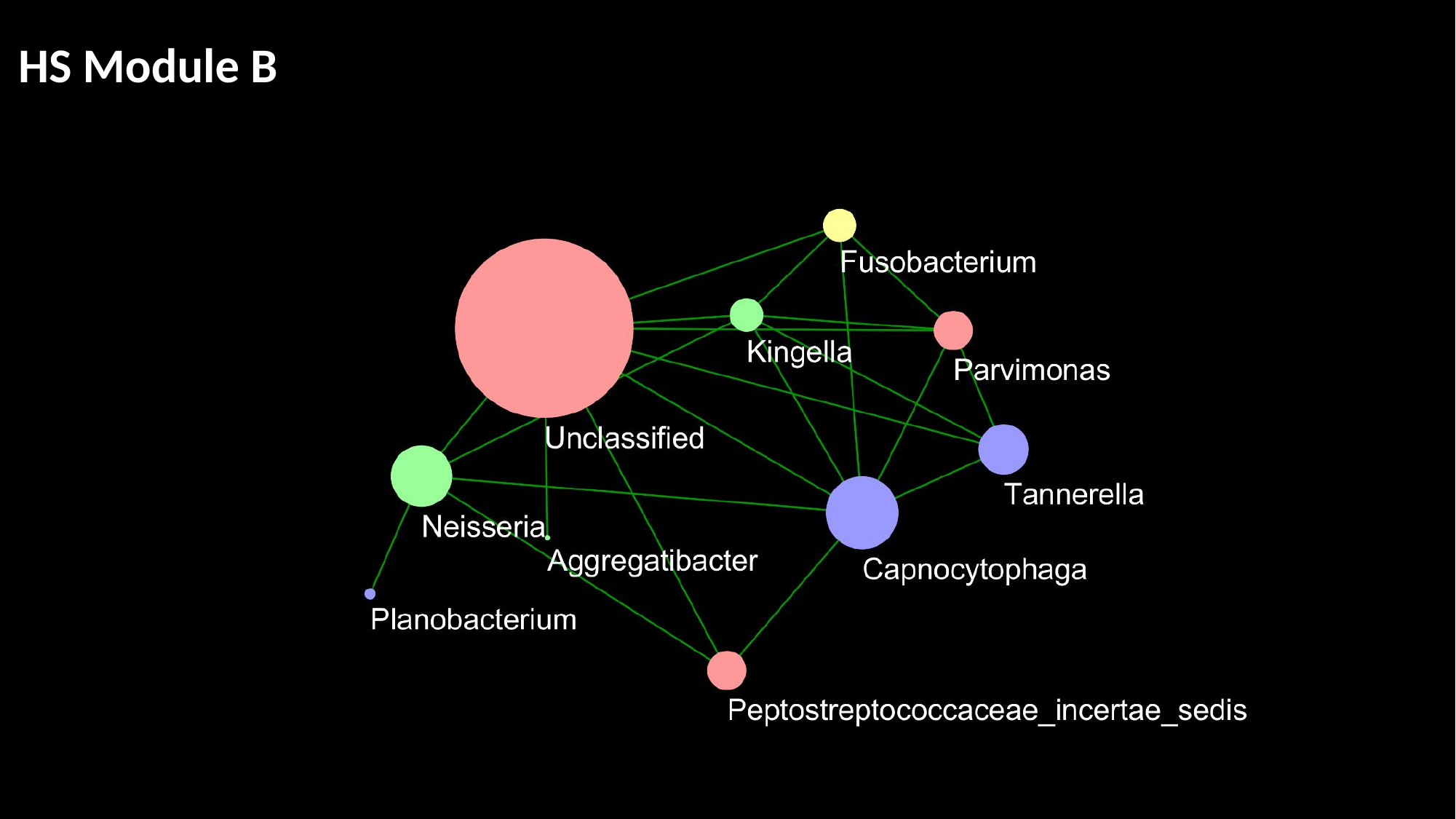

HS Module B

## Slide 29
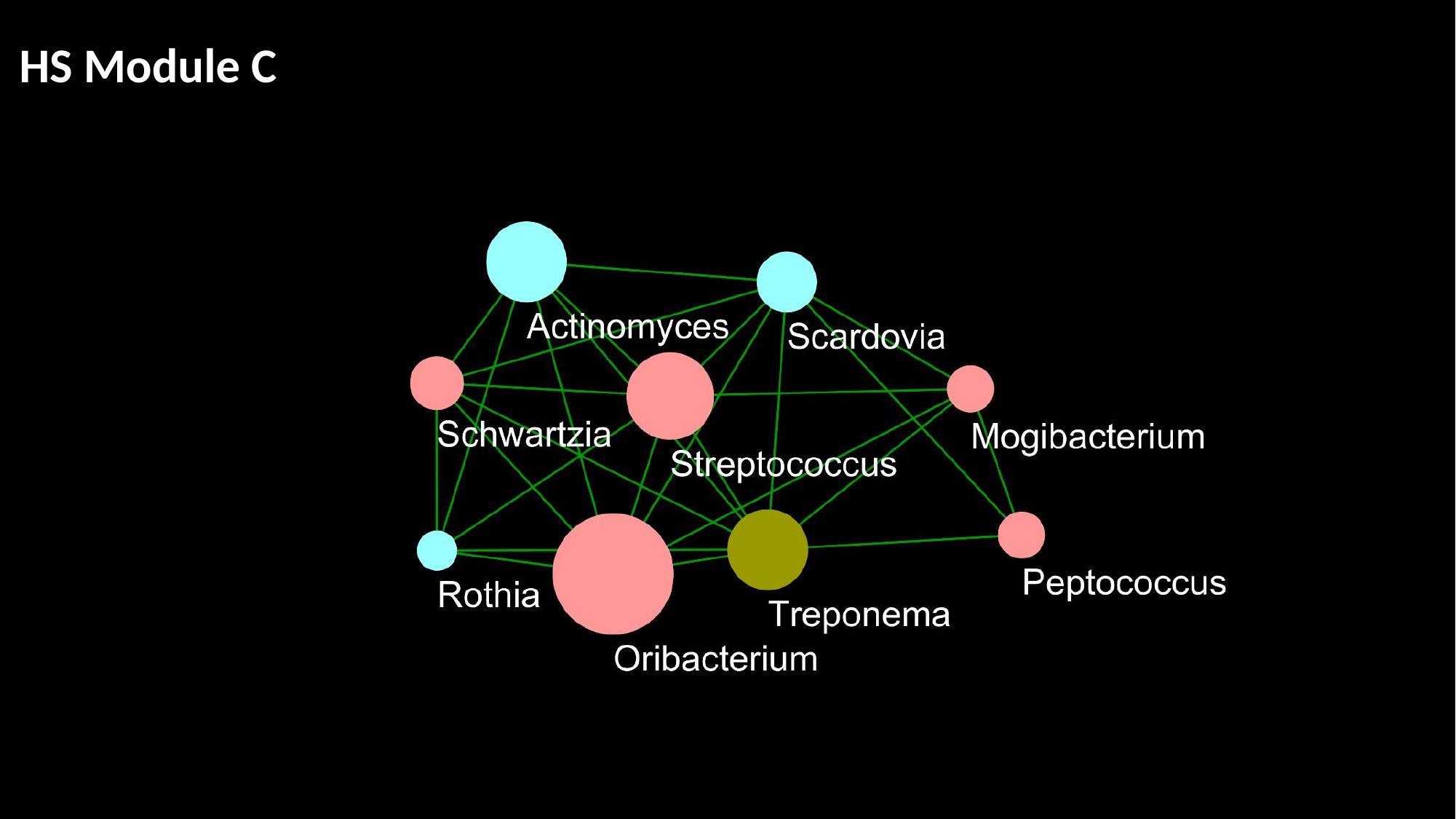

HS Module C

## Slide 30
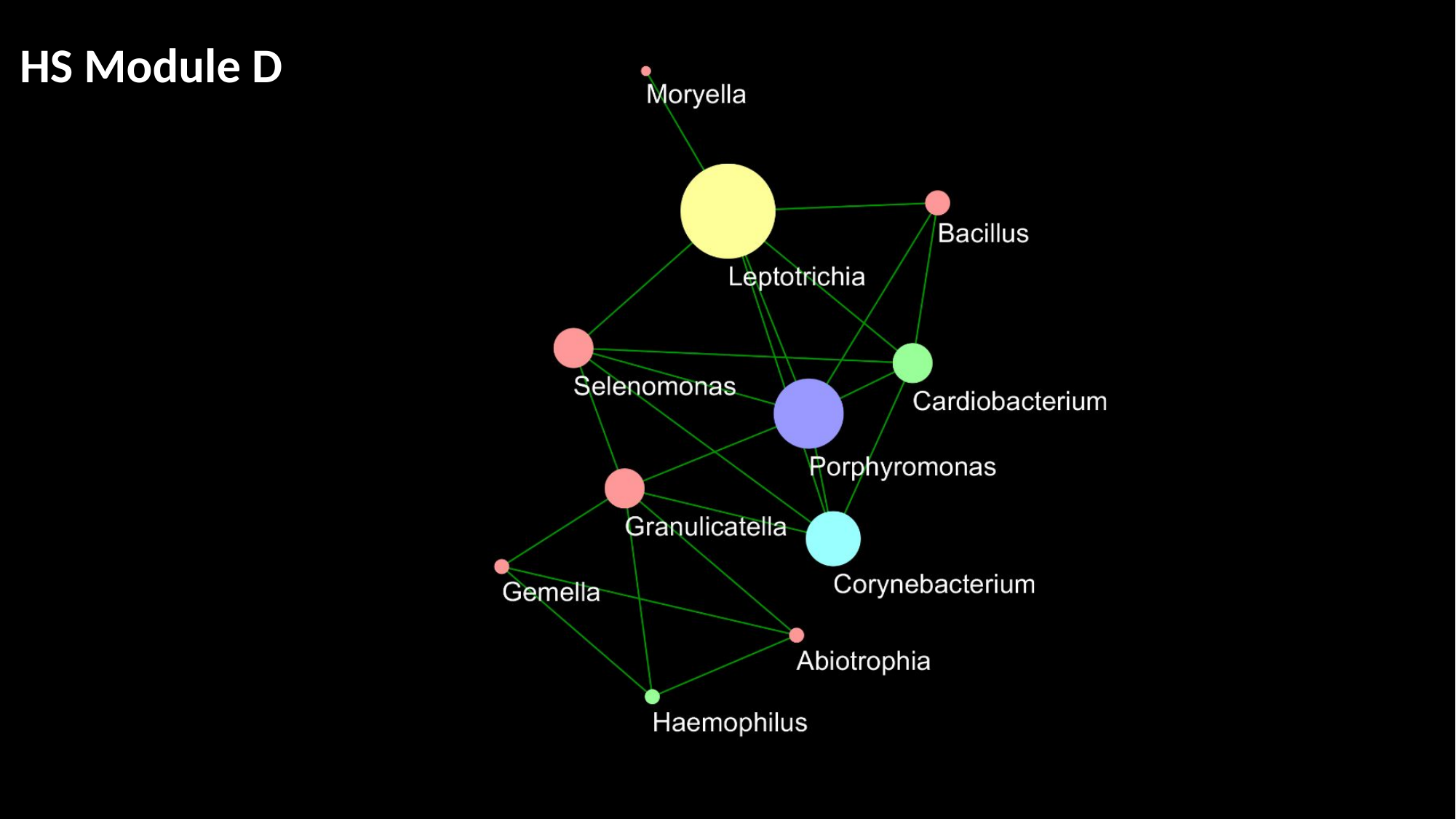

HS Module D
